# Cobalt‐Catalyzed Dehydrogenative C−H Silylation of Alkynylsilanes

**DOI:** 10.1002/chem.202103629

**Published:** 2021-11-08

**Authors:** Hanna Stachowiak, Krzysztof Kuciński, Fabian Kallmeier, Rhett Kempe, Grzegorz Hreczycho

**Affiliations:** ^1^ Department of Chemistry and Technology of Silicon Compounds Faculty of Chemistry Adam Mickiewicz University in Poznań Uniwersytetu Poznańskiego 8 61-614 Poznań Poland; ^2^ Inorganic Chemistry II–Catalyst Design Sustainable Chemistry Centre University of Bayreuth 95440 Bayreuth Germany; ^3^ Centre for Advanced Technologies Adam Mickiewicz University in Poznań Uniwersytetu Poznańskiego 10 61-614 Poznań Poland

**Keywords:** cobalt, dehydrogenative coupling, pincer ligands, silanes, silylation

## Abstract

Herein, we report that a cobalt catalyst permits the general synthesis of substituted alkynylsilanes through dehydrogenative coupling of alkynylsilanes and hydrosilanes. Several silylated alkynes, including di‐ and trisubstituted ones, were prepared in a one‐step procedure. Thirty‐seven compounds were synthesized for the first time by applying our catalyst system. The alkynylsilanes bearing hydrosilyl moieties provide an opportunity for further functionalization (e. g., hydrosilylation). The use of primary silanes as substrates and precatalyst activators permits the use of inexpensive and easily accessible 3d metal precatalysts, and avoids the presence of additional activators.

## Graphical abstract

**Multitasking silanes**: Cobalt‐catalyzed sp C−H silylation by a dehydrogenative coupling between silylacetylenes and hydrosilanes is reported. As a result, a variety of complex organosilicon derivatives was obtained. An inexpensive and easy‐to‐synthesize catalyst can be used due to the dual role of phenylsilane as substrate and activator.

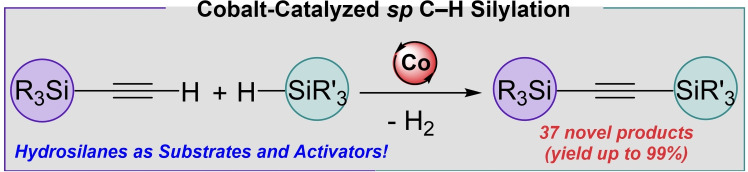

Alkynylsilanes (silylacetylenes) are very important species^[1]^ that can be used in a plethora of relevant transformations (such as protection of reactive groups^[2]^ and carbon–carbon bond formations^[3]^). There are several methods for their synthesis, by using halosilanes,^[4]^ silylamines,^[5]^ silyl triflates,^[6]^ and, more recently, silyl alkynoates.^[7]^ All of them have some benefits and drawbacks, whereby the latter can be reduced to a minimum by using a concept of a dehydrogenative coupling between alkynes and hydrosilanes.^[8]^ This very elegant, atom‐efficient, and environmentally benign cross‐coupling reaction, by definition, liberates only dihydrogen as the single by‐product. Since the first strategy reported by Voronkov,^[9a]^ a number of homo‐ and heterogeneous approaches have been developed,^[9]^ including even main‐group‐mediated pathways.^[9g]^ However, here it should be noted that possible competition between coupling and addition routes (hydrosilylation) can lead to reduced selectivity and efficiency, as well as issues concerning isolation of the desired product.

Sustainable and atom‐efficient synthetic approaches that proceed with 3d‐metal catalysts have gained significant attention recently.^[10]^ They provide prospects for environmentally friendly, cheaper, and less‐toxic processes than has been the case with many commonly used noble metals such as palladium, ruthenium, or rhodium. 3d‐metal catalysts become especially attractive if novel selectivity patterns are observed. Among numerous types of complexes, these stabilized by pincer ligands are known as very robust, chemo‐, and regioselective catalysts.^[11]^ Despite the indisputable progress, cobalt‐catalyzed functionalization within the field of organosilicon chemistry is still largely limited to a few examples (e. g., alkene/alkyne hydrosilylation,^[12]^ dehydrogenative silylation of alkenes/silanols,^[13]^ etc.^[14]^).

Within the program on sustainable organosilicon synthesis (G.H.)^[15]^ combined with an experience in cobalt pincer complex catalysis (R.K.),^[16]^ our groups have joined forces to examine the dehydrogenative silylation of alkynes. In this article, we report on the catalytic silylation of alkynylsilanes with hydrosilanes for the selective formation of several symmetrical and unsymmetrical silylacetylenes, by using cobalt complexes stabilized by PN5P ligands as the (pre)catalysts (Scheme 1). The salient features of our strategy are a) sp C−H silylation through versatile cobalt catalysis, b) small amount of the precatalyst, c) mild reaction conditions, d) efficient transformation of several functionalized acetylenes, and e) an unprecedented dual role of hydrosilanes – as substrates and activators.

**Scheme 1:**
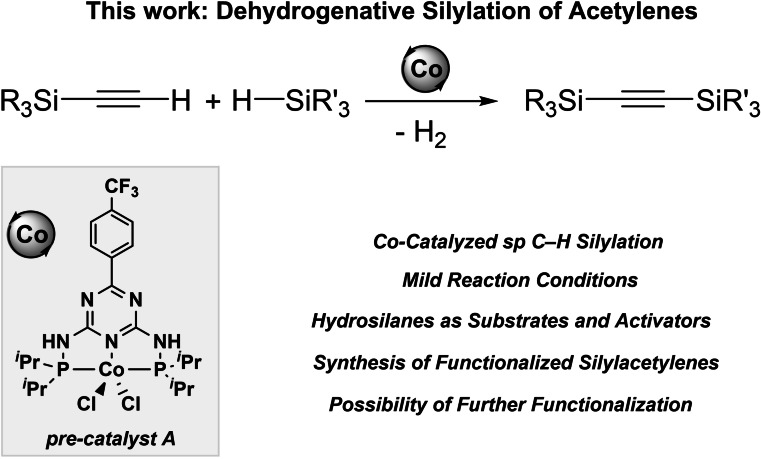
Cobalt‐catalyzed sp C−H silylation of several silylacetylenes.

First, we examined the silylation of trimethylsilylacetylene (**2 a**) with phenylsilane (**1 a**) in the presence of several previously synthesized Co complexes **A**–**D** (Table S1 in the Supporting Information). A variety of readily available bases and commonly used solvents was examined (Tables 1 and S1). Furthermore, we have also recorded some tests in the absence of Co‐precatalyst (Table 1, entry 2), and with Co‐starting material (Table 1, entry 3).

**Table 1:** Optimization of cobalt‐catalyzed sp C−H silylation.^[a]^

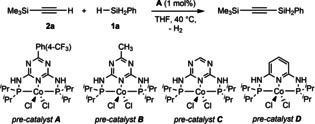			
	Variation from standard conditions	Conversion of **2 a** [%]^[b]^	Yield of **3 aa** [%]^[c]^
1	no change	99	94
2	no catalyst	0	–
3	CoCl_2_ instead of **A**	0	–
4	0.5 mol% of **A**	94	90
5	**B** instead of **A**	55	–
6	**C** instead of **A**	0	–
7	**D** instead of **A**	62	–
8	with 1.0 equiv. of **1 a**	99	70^[d]^
9	with 2 mol% of LiHBEt_3_	83	–
10	with 2 mol% of NaHBEt_3_	78	–
11	with 2 mol% of KHBEt_3_	71	–
12	with 2 mol% of KOtBu	88	–
13	at room temperature	97	91
14	in toluene	0	–
15	in 1,4‐dioxane	21	–
16	in diglyme	44	–
17	with 1.5 equiv. of **1 b** ^[e]^ instead of **1 a**	98	90

[a] General reaction conditions: **1 a** (1.3 equiv.), **2 a** (1 equiv.), **A** (1.0 mol%), under argon, 40 °C, 22 h. [b] Conversion of **2 a** determined by GC with *n*‐dodecane as the internal standard. [c] Isolated yield. [d] 23 % of trisilyl‐bis(acetylene). [e] *n*‐Hexylsilane.

Control experiments showed the essential role of the cobalt catalyst (Table 1, entry 2), whereas simple cobalt chloride was not active in this process (Table 1, entry 3). Moreover, it turned out, that in the presence of 0.5 mol% of **A**, the observed conversion was slightly lower (Table 1, entries 4). In general, other complexes **B**–**D** were significantly less active in the sp C−H silylation (Table 1, entry 5–7). Notably, when 1.0 equiv. of **1 a** was used, the reaction was less selective (it gives 23 % of trisilyl‐bis(acetylene), Table 1, entry 8). The catalytic activity of the most promising cobalt complex **A** was subsequently checked in the presence of alkali metal‐based activators (Table 1, entries 9–12), whereby better conversion was observed without any additive. This suggested that one of the substrates plays a dual role in the presented catalytic system and further studies have confirmed that hydrosilane was responsible for this effect. Last but not least, THF turned out to be the optimal solvent for further experimentation. At this point, it is also worth to mention, that only silyl‐substituted alkynes gave satisfactory results in terms of chemoselectivity. The non‐silylated acetylenes (e. g., phenylacetylene, 4‐ethynyltoluene, 4‐ethynylanisole, 4‐ethynylbenzonitrile, 1‐chloro‐4‐ethynylbenzene, 1‐ethynyl‐4‐fluorobenzene, and 4‐ethynyl‐α,α,α‐trifluorotoluene) or silylated unsaturated alcohols (e. g., 3‐(trimethylsilyloxy)but‐1‐yne, 3‐(triethylsilyloxy)but‐1‐yne, and *tert*‐butyldimethyl(2‐propynyloxy)‐silane) led to the mixture of products and lower conversion rates (even under harsh conditions).

With the optimized conditions in hand, we investigated the scope of the sp C−H silylation with **1 a** or *n*‐hexylsilane (**1 b**), as well as commercially inaccessible *p*‐tolylsilane (**1 c**; Scheme 2, top). Thus, a variety of unsymmetrical bis(silyl)acetylenes was prepared with excellent isolated yields, under mild reaction conditions, and at low loading of the precatalyst **A** (Scheme 2, **3 aa**–**ak**, **3 ba**–**bk** and **3 cc**). We also probed the robustness of this Co‐mediated approach by employing more challenging vinyl‐substituted silylacetylene **2 j**. Thus, the C=C double bond remained untouched (**3 aj** and **3 bj**), showing the high chemoselectivity of this protocol. Additionally, this fact provides the possibility of subsequent functionalization, by using the alkene function. Given the success of the cobalt‐catalyzed mono‐dehydrogenative coupling, we wondered whether a second de‐hydrocoupling would be achieved (keeping in mind our previous observation, Table 1, entry 8). As the result (Table S2), we discovered that only hydrosilane **1 a** can selectively lead to products with three silyl substituents. Notably, larger excess of silylacetylene (>3.5 equiv.) caused inferior results (Table S2). However, when bulkier acetylenes were employed as the coupling partners, the desired bis(silyl)acetylenes were selectively obtained, without any traces of trisubstituted **1 a**. All these results are summarized in Scheme 2 (bottom, Table S3). Next, we set out to investigate the scope for secondary hydrosilanes. Due to the beneficial effect of using a greater amount of hydrosilanes, together with elevated temperatures, we wondered whether Co‐catalyzed C−H silylation would be viable (for detailed information, see Table S4).

**Scheme 2:**
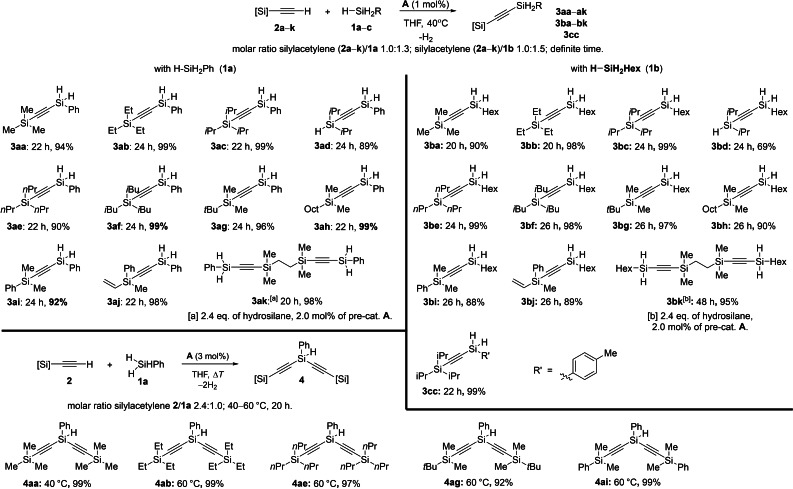
Scope for cobalt‐catalyzed sp C−H mono‐ (top) and double‐silylation (bottom) of silylacetylenes with hydrosilanes.

To our delight, under forcing conditions (at 100 °C, which also forced a change in the type of the solvent), a reaction of **2 c** with diphenylsilane (**1 d**) took place in the presence of 10 mol% of **A**. Unfortunately, besides the desired product, we have also detected the redistribution of dihydrosilane in moderate quantity. Considering that at a lower temperature (RT–60 °C) the redistribution was not detected, we assumed that probably primary silanes (formed at elevated temperature)^[17]^ are still true activators of the cobalt precatalysts.

With this in mind, we examined the use of phenylsilane (**1 a**) as the precatalyst activator (molar ratio **1 a**/**A** as 2.0 : 1.0) in a dehydrogenative coupling of silylacetylenes with secondary silanes under slightly milder reaction conditions. After considerable experimentation (Table S3), we found that secondary silanes **1 d**–**g** may provide satisfactory results in reaction with **2 c**, leading to the corresponding silylated acetylenes in good yields (Scheme 3).

**Scheme 3:**
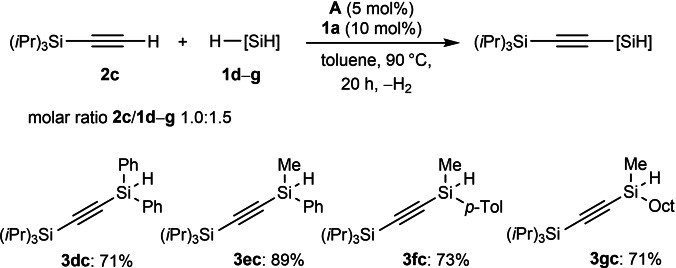
Scope for cobalt‐catalyzed sp C−H silylation of silylacetylenes with dihydrosilanes.

Next, we turned our attention to previously obtained alkynes bearing both alkynyl and hydro substituents. Given the success of H_3_SiPh (**1 a**) as the activator, we wondered whether a dehydrocoupling between sterically hindered bis(silyl)acetylenes with another silylacetylenes would be viable. For this purpose, **2 a** and **3 ac** were chosen as model substrates (the optimization findings are summarized in Table S5). As we could observe, the temperature value is crucial for this transformation. Thereby, we have established individual reaction conditions for each substrate, and consequently, another six examples of unsymmetrical derivatives were obtained (Scheme 4).

**Scheme 4:**
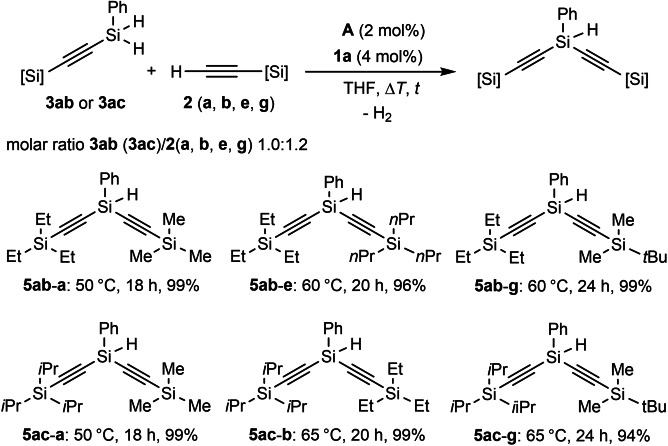
Scope for cobalt‐catalyzed sp C−H silylation of silylacetylenes with dihydro‐bis(silyl)acetylenes.

Finally, we have also demonstrated the utility of obtained silylacetylenes with SiH functionalities in the subsequent hydrosilylation process. A classical Karstedt‐catalyzed Si−H addition to unsaturated systems constitutes a very elegant synthetic pathway in preparation of functionalized organosilicons,^[18]^ and in our case, it enabled the synthesis of novel multifunctional silylacetylenes (Scheme 5).

**Scheme 5:**
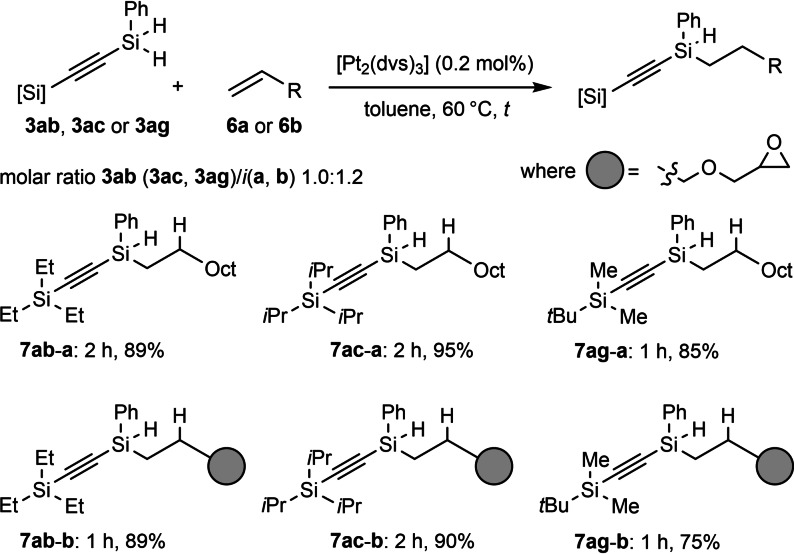
Pt‐catalyzed hydrosilylation of olefins with [Si−H]‐containing bis‐silylacetylenes.

To gain mechanistic insights into this Co‐catalyzed reaction, ^[19]^ we conducted preliminary experiments. Firstly, 2.2 equiv. of **1 a** were added to 1 equiv. of precatalyst **B** in [D_8_]THF and stirred at 50 °C for 1 h. The reaction was tracked on ^1^H NMR, initially giving the mixture of [Co−H] species, as well as PhSiH_2_Cl and dihydrogen (but without any phenylsilane residues; Supplement 2 in the Supporting Information). Next, we increased the amount of **1 a** to 10 equiv., which exclusively resulted in the formation of one [Co−H] entity (Supplement 3 in the Supporting Information), indicating the generation of (PN5P)Co^III^H_2_(SiH_2_Ph) (Scheme 6).^[19c]^ Notably, such Co^I^/Co^III^ mechanism was also suggested in other TM‐catalyzed C−H activation processes.[^18b^, ^18d^] Furthermore, a dehydrogenative coupling between **1 a** and **2 a** was performed in the presence of TEMPO (1 equiv.), and led to the expected product (90 %), thereby implying that radical pathway is likely, not operative.

**Scheme 6:**
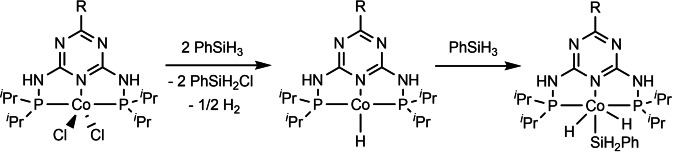
Proposed activation of the precatalyst.

A plausible catalytic cycle based on previous literature and our experimental results is presented in Scheme 7.

**Scheme 7:**
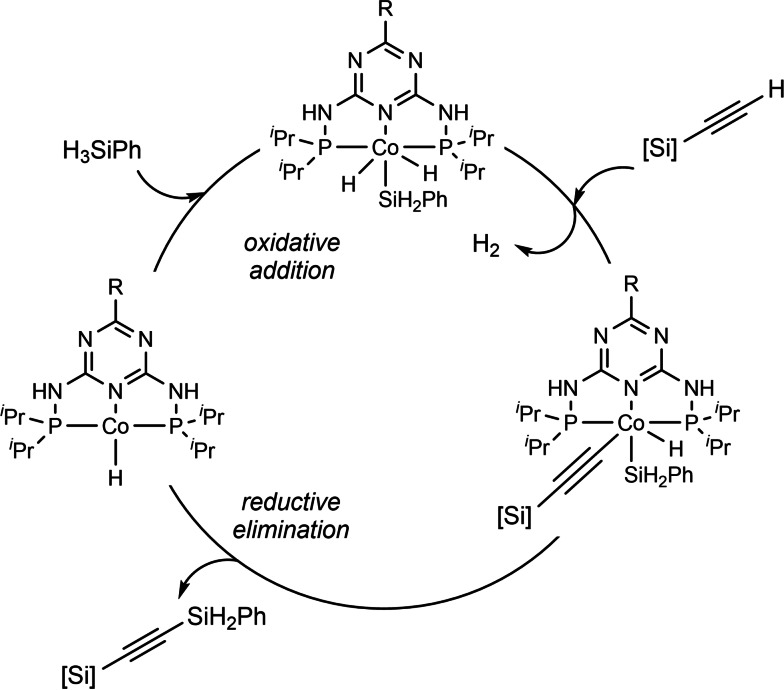
Proposed catalytic cycle.

The cobalt complex (PN5P)Co^III^H_2_(SiH_2_Ph) undergoes ligand replacement with the silylacetylene molecule, with simultaneous liberation of a dihydrogen molecule. Consequently, the alkynyl cobalt complex is generated, in the form of two possible isomers (Supplement 4 in the Supporting Information).^[20]^ Finally, the reaction proceeds by reductive elimination to afford the final product and regenerates the active cobalt(I) catalyst.

In conclusion, we have reported the selective sp C−H silylation of silylacetylenes with primary and secondary hydrosilanes by using cobalt catalysis. Under environmentally benign reaction conditions, a series of symmetrical and unsymmetrical silylacetylenes (44 compounds, including products of hydrosilylation) were synthesized in good to excellent yields (up to 99 %). Considering the combination of desirable features, such as high chemoselectivity, high atom economy, benign reaction conditions, and the use of a 3d‐metal catalyst, this reaction system is expected to provide a promising alternative to existing methodologies and an attractive approach for the synthesis of complex organosilicon compounds. The use of primary silanes as substrates and precatalyst activators is also beneficial as additional activators can be avoided. Mechanistic studies provided strong support for the involvement of Co^I^/Co^III^ pathway.

## Conflict of interest

The authors declare no conflict of interest.

## Supporting information

As a service to our authors and readers, this journal provides supporting information supplied by the authors. Such materials are peer reviewed and may be re‐organized for online delivery, but are not copy‐edited or typeset. Technical support issues arising from supporting information (other than missing files) should be addressed to the authors.

Supporting Information

## Data Availability

The data that support the findings of this study are available in the supplementary material of this article.
