# Supplementary material for: Cobalt‐Catalyzed Dehydrogenative C−H Silylation of Alkynylsilanes
Source: Chemistry. 2021 Nov 8;28(1):e202103629. doi: 10.1002/chem.202103629 (PMC9299208; doi:10.1002/chem.202103629)
Supplement: Supplementary file 1 — Supporting Information [file CHEM-28-0-s001.pdf]

Chemistry–A European Journal

Supporting Information

Cobalt-Catalyzed Dehydrogenative C–H Silylation of Alkynylsilanes

Hanna Stachowiak, Krzysztof Kuciński, Fabian Kallmeier, Rhett Kempe,* and Grzegorz Hreczycho*

CONTENT

GENERAL INFORMATION 6
OPTIMIZATION OF REACTION CONDITIONS 7
Table S1. Optimization for dehydrogenative coupling with primary silanes 7
Table S2. Optimization of Co-pre-catalyst loading and molar ratio for *double*-dehydrogenative coupling 8
Table S3. Optimization of temperature for *double*-dehydrogenative coupling 8
Table S4. Optimization for dehydrogenative coupling with secondary silanes 9
Table S5. Optimization for dehydrogenative coupling with silylacetylenes. 9
GENERAL SYNTHETIC PROCEDURES 10
Cobalt-catalyzed dehydrogenative coupling of silylacetylenes with primary silanes 10
The synthesis of (bis)silylacetylenes 10
The synthesis of tri-silyl-bis(acetylenes) 10
Cobalt-catalyzed dehydrogenative coupling of silylacetylenes with secondary silanes 10
Cobalt-catalyzed dehydrogenative coupling of bis(silyl)acetylenes with silylacetylenes 11
Platinum-catalyzed hydrosilylation of olefins with bis(silyl)acetylenes 11
CHARACTERISATION DATA FOR ALL PRODUCTS 12
Trimethyl((phenylsilyl)ethynyl)silane (3aa) 12
Triethyl((phenylsilyl)ethynyl)silane (3ab) 12
Triisopropyl((phenylsilyl)ethynyl)silane (3ac) 12
Diisopropyl((phenylsilyl)ethynyl)silane (3ad) 13
((Phenylsilyl)ethynyl)tripropylsilane (3ae) 13
Triisobutyl((phenylsilyl)ethynyl)silane (3af) 14
Tert-butyldimethyl((phenylsilyl)ethynyl)silane (3ag) 14
Dimethyl(octyl)((phenylsilyl)ethynyl)silane (3ah) 15
Dimethyl(phenyl)((phenylsilyl)ethynyl)silane (3ai) 15
Methyl(phenyl)((phenylsilyl)ethynyl)(vinyl)silane (3aj) 16
1,2-Bis(((phenylsilyl)ethynyl)dimethylsilyl)ethane (3ak) 16
((Hexylsilyl)ethynyl)trimethylsilane (3ba) 17
Triethyl((hexylsilyl)ethynyl)silane (3bb) 17
((Hexylsilyl)ethynyl)triisopropylsilane (3bc) 17
((Hexylsilyl)ethynyl)diisopropylsilane (3bd) 18
((Hexylsilyl)ethynyl)tripropylsilane (3be) 18
((Hexylsilyl)ethynyl)triisobutylsilane (3bf) 19

Tert-butyl((hexylsilyl)ethynyl)dimethylsilane (3bg) 19
((Hexylsilyl)ethynyl)dimethyl(octyl)silane (3bh)........ 20
((Hexylsilyl)ethynyl)dimethyl(phenyl)silane (3bi)........ 20
((Hexylsilyl)ethynyl)(methyl)(phenyl)(vinyl)silane (3bj) 21
1,2-Bis(((hexylsilyl)ethynyl)dimethylsilyl)ethane (3bk)........ 21
Triisopropyl((p-tolylsilyl)ethynyl)silane (3cc) 22
[Bis[2-(trimethylsilyl)ethynyl]silyl]benzene (4aa)........ 22
[Bis[2-(triethylsilyl)ethynyl]silyl]benzene (4ab)........ 23
[Bis[2-(tripropylsilyl)ethynyl]silyl]benzene (4ae) 23
[Bis[2-(tert-butyl(dimethyl)silyl)ethynyl]silyl]benzene (4ag) 23
[Bis[2-(dimethyl(phenyl)silyl)ethynyl]silyl]benzene (4ai)........ 24
[(Diphenylsilyl)ethynyl]triisopropylsilane (3dc) 24
[(Methyl(phenyl)silyl)ethynyl]triisopropylsilane (3ec)........ 25
[(Methyl(p-tolyl)silyl)ethynyl]triisopropylsilane (3fc) 25
[(Methyl(octyl)silyl)ethynyl]triisopropylsilane (3gc) 26
[[2-(Triethylsilyl)ethynyl][2-(trimethylsilyl)ethynyl]silyl]benzene (5ab-a) 26
[[2-(Triethylsilyl)ethynyl][2-(tri-n-propylsilyl)ethynyl]silyl]benzene (5ab-e)........ 27
[[2-(Tert-butyl(dimethyl)silyl)ethynyl][2-(triethylsilyl)ethynyl]silyl]benzene (5ab-g) 27
[[2-(Triisopropylsilyl)ethynyl][2-(trimethylsilyl)ethynyl]silyl]benzene (5ac-a)........ 28
[[2-(Triethylsilyl)ethynyl][2-(triisopropylsilyl)ethynyl]silyl]benzene (5ac-b)........ 28
[[2-(Tert-butyl(dimethyl)silyl)ethynyl][2-(triisopropylsilyl)ethynyl]silyl]benzene (5ac-g)........ 29
((Decyl(phenyl)silyl)ethynyl)triethylsilane (7ab-a) 29
((Decyl(phenyl)silyl)ethynyl)triisopropylsilane (7ac-a)........ 30
Tert-butyl((decyl(phenyl)silyl)ethynyl)dimethylsilane (7ag-a) 30
Triethyl(((3-(glycidoxy)propyl)(phenyl)silyl)ethynyl)silane (7ab-b)........ 31
Triisopropyl(((3-(glycidoxy)propyl)(phenyl)silyl)ethynyl)silane (7ac-b) 31
Tert-butyl(dimethyl)(((3-(glycidoxy)propyl)(phenyl)silyl)ethynyl)silane (7ag-b) 32
SPECTRA FOR ALL PRODUCTS........ 33
Trimethyl((phenylsilyl)ethynyl)silane (3aa)........ 33
Triethyl((phenylsilyl)ethynyl)silane (3ab)........ 35
Triisopropyl((phenylsilyl)ethynyl)silane (3ac) 37
Diisopropyl((phenylsilyl)ethynyl)silane (3ad)........ 39
((Phenylsilyl)ethynyl)tripropylsilane (3ae) 41
Triisobutyl((phenylsilyl)ethynyl)silane (3af)........ 43

Tert-butyldimethyl((phenylsilyl)ethynyl)silane (3ag) 45
Dimethyl(octyl)((phenylsilyl)ethynyl)silane (3ah) 47
Dimethyl(phenyl)((phenylsilyl)ethynyl)silane (3ai) 49
Methyl(phenyl)((phenylsilyl)ethynyl)(vinyl)silane (3aj) 51
1,2-Bis(((phenylsilyl)ethynyl)dimethylsilyl)ethane (3ak) 53
((Hexylsilyl)ethynyl)trimethylsilane (3ba) 55
Triethyl((hexylsilyl)ethynyl)silane (3bb) 57
((Hexylsilyl)ethynyl)triisopropylsilane (3bc) 59
((Hexylsilyl)ethynyl)diisopropylsilane (3bd) 61
((Hexylsilyl)ethynyl)tripropylsilane (3be) 63
((Hexylsilyl)ethynyl)triisobutylsilane (3bf) 65
Tert-butyl((hexylsilyl)ethynyl)dimethylsilane (3bg) 67
((Hexylsilyl)ethynyl)dimethyl(octyl)silane (3bh) 69
((Hexylsilyl)ethynyl)dimethyl(phenyl)silane (3bi) 71
((Hexylsilyl)ethynyl)(methyl)(phenyl)(vinyl)silane (3bj) 73
1,2-Bis(((hexylsilyl)ethynyl)dimethylsilyl)ethane (3bk) 75
Triisopropyl((p-tolylsilyl)ethynyl)silane (3cc) 77
[Bis[2-(trimethylsilyl)ethynyl]silyl]benzene (4aa) 79
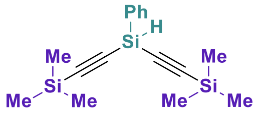

.... 79
[Bis[2-(triethylsilyl)ethynyl]silyl]benzene (4ab) 81
[Bis[2-(tripropylsilyl)ethynyl]silyl]benzene (4ae) 83
[Bis[2-(tert-butyl(dimethyl)silyl)ethynyl]silyl]benzene (4ag) 85
[Bis[2-(dimethyl(phenyl)silyl)ethynyl]silyl]benzene (4ai) 87
[(Diphenylsilyl)ethynyl]triisopropylsilane (3dc) 89
[(Methyl(phenyl)silyl)ethynyl]triisopropylsilane (3ec) 91
[(Methyl(p-tolyl)silyl)ethynyl]triisopropylsilane (3fc) 93
[(Methyl(octyl)silyl)ethynyl]triisopropylsilane (3gc) 95
[[2-(Triethylsilyl)ethynyl][2-(trimethylsilyl)ethynyl]silyl]benzene (5ab-a) 97
[[2-(Triethylsilyl)ethynyl][2-(tri-n-propylsilyl)ethynyl]silyl]benzene (5ab-e) 99
[[2-(Tert-butyl(dimethyl)silyl)ethynyl][2-(triethylsilyl)ethynyl]silyl]benzene (5ab-g) 101
[[2-(Triisopropylsilyl)ethynyl][2-(trimethylsilyl)ethynyl]silyl]benzene (5ac-a) 103
[[2-(Triethylsilyl)ethynyl][2-(triisopropylsilyl)ethynyl]silyl]benzene (5ac-b) 105
[[2-(Tert-butyl(dimethyl)silyl)ethynyl][2-(triisopropylsilyl)ethynyl]silyl]benzene (5ac-g) 107

((Decyl(phenyl)silyl)ethynyl)triethylsilane (7ab-a) 109
((Decyl(phenyl)silyl)ethynyl)triisopropylsilane (7ac-a) 111
Tert-butyl((decyl(phenyl)silyl)ethynyl)dimethylsilane (7ag-a) 113
Triethyl(((3-(glycidoxy)propyl)(phenyl)silyl)ethynyl)silane (7ab-b) 115
Triisopropyl(((3-(glycidoxy)propyl)(phenyl)silyl)ethynyl)silane (7ac-b) 117
Tert-butyl(dimethyl)(((3-(glycidoxy)propyl)(phenyl)silyl)ethynyl)silane (7ag-b) 119
MECHANISTIC STUDIES 121
Supplement 1 121
Supplement 2 122
Supplement 3 123
Supplement 4 124
Supplement 5 124
REFERENCES 124

GENERAL INFORMATION

Air- and moisture sensitive reactions were carried out under argon atmosphere using standard Schlenk techniques or a glove box. Solvents used for all experiments were purchased from Honeyweel or Sigma Aldrich (Merck), dried over calcium hydride (CaH_2) and purified by distillation. Toluene was additionally dried over sodium, and THF over sodium with benzophenone system. Ligands and Co-complexes were prepared in accordance with previously reported methods,[1] using reagents purchased from Sigma Aldrich (Merck) or ABCR GmBH. Commercially available hydrosilanes (*e.g.*, phenylsilane, n-hexylsilane, methylphenylsilane, *ect.*) as well as alkynylsilanes (*e.g.*, trimethylsilylacetylene, triethylsilylacetylene, triisopropylsilylacetylene, *ect.*) were purchased from Sigma Aldrich (Merck), ABCR GmBH, Ambeed, Apollo Scientific or Acros Organics, dried over calcium hydride and purified by distillation. Other hydrosilanes (*e.g.*, methyl-*p*-tolylsilane, methyloctylsilane, *ect.*) and alkynylsilanes (*e.g.*, tri-*n*-propylsilylacetylene, methylphenylvinylsilylacetylene, 1,2-bis(ethynyldimethylsilyl)ethane, *ect.*) were synthesized from corresponding chlorosilanes by well-known procedures using respectively $LiAlH_4$ (reduction process) and ethynylmagnesium bromide solution in THF (Grignard reagent). Potassium tert-butoxide was dried under high vacuum at 70°C and stored in a glove box. Triethylborohydride solutions in THF ($LiHBEt_3$, $NaHBEt_3$ and $KHBEt_3$) were purchased from Sigma Aldrich (Merck) and used as received. The progress of reactions (conversion of alkynylsilane, (bis)silylacetylene or hydrosilane) was monitored by GC chromatography using Bruker Scion 460-GC and Agilent 5977B GC/MSD with Agilent 8860 GC System. The structures of products were determined by NMR spectroscopy and MS spectrometry. The ^{1}H NMR (400 or 600 MHz), ^{13}C NMR (101 or 151 MHz) and ^{29}Si NMR (79 or 119 MHz) spectra were recorded on Bruker Avance III HD NanoBay spectrometer, using chloroform-d_1 ($CDCl_3$), benzene-d_6 (C_6D_6) or tetrahydrofuran-d_8 (THF-d_8) as the solvents. Deuterated solvents were purchased from respectively Deutero GmbH ($CDCl_3$ 99.6 atom% D) and Sigma Aldrich (Merck) (C_6D_6 99.8 atom% D, THF-d_8 99.5 atom% D) and used as received.

OPTIMIZATION OF REACTION CONDITIONS

Table S1. Optimization for dehydrogenative coupling with primary silanes.[a]

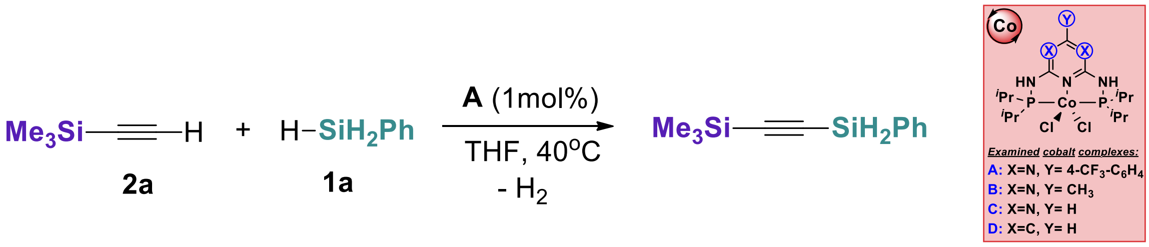

Entry	Variation from standard conditions	Conversion of **2a** [%][b]	Yield of **3aa** [%][c]
1	no change	99	94
2	no catalyst	0	-
3	$CoCl_2$ instead of **A**	0	-
4	0.5 mol% of **A**	94	90
5	**B** instead of **A**	55	-
6	**C** instead of **A**	0	-
7	**D** instead of **A**	62	-
8	with 1.0 eq. of **1a**	99	70[d]
9	with 2 mol% of $LiHBEt_3$	83	-
10	with 2 mol% of $NaHBEt_3$	78	-
11	with 2 mol% of $KHBEt_3$	71	-
12	with 2 mol% of KOtBu	88	-
13	at room temperature	97	91
14	in toluene	0	-
15	in 1,4-dioxane	21	-
16	in diglyme	44	-
17	with 1.3 eq. of **1b** instead of **1a**	93	-
18	with 1.5 eq. of **1b** instead of **1a**	98	90

[a] General reaction conditions: **1a** (1.3 eq.), **2a** (1 eq.), **A** (1.0 mol%), under argon atmosphere, 40°C, 22 h. [b] Conversion of **2a** determined by GC with n-dodecane as the internal standard. [c] Isolated yield. [d] 23% of tri-silyl-bis(acetylene).

Table S2. Optimization of Co-pre-catalyst loading and molar ratio for *double*-dehydrogenative coupling.[a]

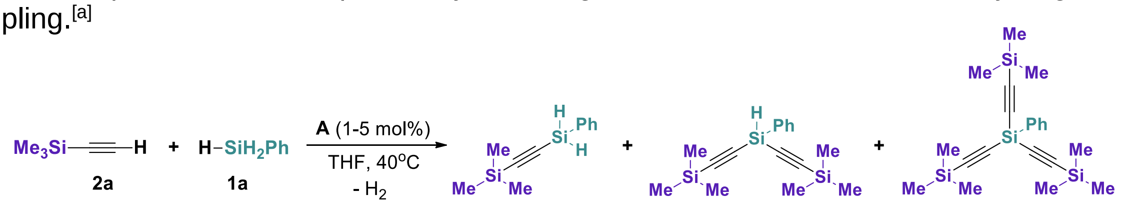

Entry	**A** [mol%]	**2a** eq.	Conversion of **1a** [%][b]	Selectivity [%][c] [mono] : [double] : [triple]
1	1.0	2.2	99	26 : 74 : 0
2	3.0	2.4	99	0 : 98 : 2
3	4.0	3.5	99	0 : 94 : 6
4	5.0	4.0	99	0 : 87 : 13

[a] General reaction conditions: **1a** (1 eq.), **2a** (2.2–4.0 eq.), **A** (1.0–5.0 mol%), under argon atmosphere, 40°C, 20 h. [b] Conversion of **1a** determined by GC with n-dodecane as the internal standard. [c] Selectivity of [mono]:[double]:[triple] dehydrogenative coupling products determined by GC.

Table S3. Optimization of temperature for *double*-dehydrogenative coupling.[a]

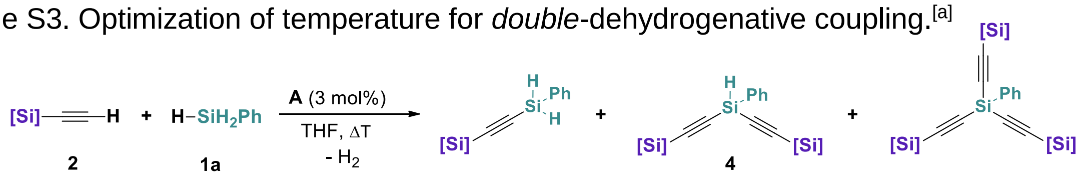

Entry	Silylacetylene	Temp. [°C]	Conversion of **1a** [%][b]	Selectivity [%][c] [mono] : [double] : [triple]	Yield of **4** [%][d]
1	2a	40	99	0 : 98 : 2	99 (**4aa**)
2		40	99	4 : 94 : 0	-
3	2b	50	99	1 : 99 : 0	-
4		60	99	0 : 100 : 0	99 (**4ab**)
5		40	99	17 : 83 : 0	-
6	2e	50	99	3 : 97 : 0	-
7		60	99	1 : 99 : 0	97 (**4ae**)
8		40	83	2 : 98 : 0	-
9	2g	50	91	1 : 99 : 0	-
10		60	96	1 : 99 : 0	92 (**4ag**)
11		40	99	27 : 73 : 0	-
12	2i	50	99	13 : 87 : 0	-
13		60	99	2 : 98 : 0	99 (**4ai**)

[a] General reaction conditions: **1a** (1 eq.), **2a** (2.4 eq.), **A** (3 mol%), under argon atmosphere, temperature (40–60°C), 20 h. [b] Conversion of **1a** determined by GC with n-dodecane as the internal standard. [c] Selectivity of [mono]:[double]:[triple] dehydrogenative coupling products determined by GC. [d] Isolated yield.

Table S4. Optimization for dehydrogenative coupling with secondary silanes.[a]

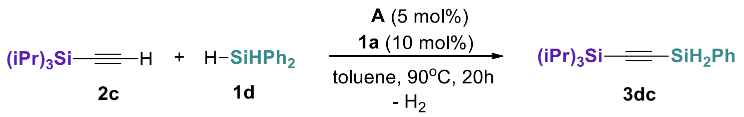

Entry	Variation from standard conditions	Conversion of **2c** [%][b]	Yield of **3dc** [%][c]
1	no change	89	71
2	with 1.0 eq. of **1d**	66	-
3	with 1.2 eq. of **1d**	81	-
4	in THF at 40°C	28	-
5	in THF at 60°C	33	-
6	at 80°C	79	-
7	at 100°C	97[d]	-
8	no **1a**, with 10 mol% of **A**	0	-
9	no **1a**, with 10 mol% of **A**, at 100°C	67[d]	-

[a] General reaction conditions: **1d** (1.5 eq.), **2c** (1 eq.), **A** (5 mol%), **1a** (10 mol%), toluene, under argon atmosphere, 90°C, 20 h. [b] Conversion of 2c determined by GC with n-dodecane as the internal standard. [c] Isolated yield. [d] deterioration of chemoselectivity.

Table S5. Optimization for dehydrogenative coupling with silylacetylenes.[a]

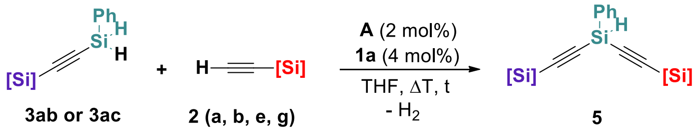

Entry	(Bis)silylacetylene	Silylacetylene	Temp. [°C]	Time [h]	Conversion of **2** [%][b]	Yield of **5** [%][c]
1		**2a**	50	18	97	99 (**5ab-a**)
2		**2e**	50	20	95	-
3	**3ab**		60	20	99	96 (**5ab-e**)
4		**2g**	50	24	94	-
5			60	24	99	99 (**5ab-g**)
6		**2a**	50	18	99	99 (**5ac-a**)
7			50	20	85	-
8		**2b**	60	20	92	-
9	**3ac**		65	20	97	99 (**5ac-b**)
10			50	24	71	-
11		**2g**	60	24	91	-
12			65	24	99	94 (**5ac-g**)

[a] General reaction conditions: **3ab** or **3ac** (1 eq.), **2 (a, b, e, g)** (1.2 eq.), **A** (2 mol%), **1a** (4 mol%), under argon atmosphere, temperature (50–65°C), definite time. [b] Conversion of **2 (a, b, e, g)** determined by GC with n-dodecane as the internal standard. [c] Isolated yield.

GENERAL SYNTHETIC PROCEDURES

Cobalt-catalyzed dehydrogenative coupling of silylacetylenes with primary silanes

The synthesis of (bis)silylacetylenes

Compounds **3aa**–**3aj** and **3ba**–**3bj and 3cc**

To a 12mL vial equipped with a magnetic stirring bar, 0.014M solution of pre-catalyst **A** (10^{-2} mmol, 0.01 eq.) in THF, primary silane (1.3 mmol, 1.3 eq. of phenylsilane or 1.5 mmol, 1.5 eq. of hexylsilane or 1.5 mmol, 1.5 eq. of p-tolylsilane), silylacetylene (1.0 mmol, 1.0 eq.) and 1 mL of THF were added under inert gas atmosphere (glove box). Subsequently, reaction mixture was stirred at 40°C for a specified time. After this time, solvent and volatile residues were evaporated under a high vacuum. In the next step, the catalyst was precipitated by the addition of pentane or hexane (1mL) and filtered from the resulting mixture. Lastly, the solvent was separated under reduced pressure giving desired product. The pure products were identified by ^{1}H, ^{13}C, and ^{29}Si NMR spectroscopies and MS spectrometry.

Compounds **3ak** and **3bk**

To a 12mL vial equipped with a magnetic stirring bar, 0.014M solution of pre-catalyst **A** ($2\cdot10^{-2}$ mmol, 0.02 eq.) in THF, primary silane (2.4 mmol, 2.4 eq. of phenylsilane or hexylsilane), silylacetylene (1.0 mmol, 1.0 eq.) and 1 mL of THF were added under inert gas atmosphere (glove box). Subsequently, reaction mixture was stirred at 40°C for a specified time. After this time, solvent and volatile residues were evaporated under a high vacuum. In the next step, the catalyst was precipitated by the addition of pentane or hexane (1mL) and filtered from the resulting mixture. Lastly, the solvent was separated under reduced pressure giving desired product. The pure products were identified by ^{1}H, ^{13}C, and ^{29}Si NMR spectroscopies and MS spectrometry.

The synthesis of tri-silyl-bis(acetylenes)

Compounds **4aa**, **4ab**, **4ae**, **4ag** and **4ai**

To a 12mL vial equipped with a magnetic stirring bar, 0.014M solution of pre-catalyst **A** ($3\cdot10^{-2}$ mmol, 0.03 eq.) in THF, phenylsilane (1.0 mmol, 1.0 eq.) and silylacetylene (2.4 mmol, 2.4 eq.) were added under inert gas atmosphere (glove box). Subsequently, reaction mixture was stirred at 60°C for specified time. After this time, solvent and volatile residues were evaporated under a high vacuum. In the next step, the catalyst was precipitated by the addition of pentane or hexane (1mL) and filtered from the resulting mixture. Lastly, the solvent was separated under reduced pressure giving desired product. The pure products were identified by ^{1}H, ^{13}C, and ^{29}Si NMR spectroscopies and MS spectrometry.

Cobalt-catalyzed dehydrogenative coupling of silylacetylenes with secondary silanes

Compounds **3dc**–**3gc**

To a 12mL vial equipped with a magnetic stirring bar, 0.03M solution of pre-catalyst **A** ($5\cdot10^{-2}$ mmol, 0.05 eq.) in toluene, phenylsilane (10^{-1} mmol, 0.1 eq.), secondary silane

(1.5 mmol, 1.5 eq.) and silylacetylene (1.0 mmol, 1.0 eq.) were added under inert gas atmosphere (glove box). Subsequently, reaction mixture was stirred at 90°C for a specified time. After this time, solvent and volatile residues were evaporated under a high vacuum. In the next step, the catalyst was precipitated by the addition of pentane or hexane (1mL) and filtered from the resulting mixture. Lastly, the solvent was separated under reduced pressure giving desired product. The pure products were identified by ^{1}H, ^{13}C, and ^{29}Si NMR spectroscopies and MS spectrometry.

Cobalt-catalyzed dehydrogenative coupling of bis(silyl)acetylenes with silylacetylenes

Compounds **5ab–a**, **5ab–e**, **5ab–g**, **5ac–a, 5ac–b** and **5ac–g**

To a 12mL vial equipped with a magnetic stirring bar, 0.014M solution of pre-catalyst **A** ($2 \cdot 10^{-2}$ mmol, 0.02 eq.) in THF, phenylsilane ($4 \cdot 10^{-2}$ mmol, 0.04 eq.), bis(silyl)acetylene (1.0 mmol, 1.0 eq.) and silylacetylene (1.2 mmol, 1.2 eq.) were added under inert gas atmosphere (glove box). Subsequently, reaction mixture was stirred at 50–65°C for a specified time. After this time, solvent and volatile residues were evaporated under a high vacuum. In the next step, the catalyst was precipitated by the addition of pentane or hexane (1mL) and filtered from the resulting mixture. Lastly, the solvent was separated under reduced pressure giving desired product. The pure products were identified by ^{1}H, ^{13}C, and ^{29}Si NMR spectroscopies and MS spectrometry.

Platinum-catalyzed hydrosilylation of olefins with bis(silyl)acetylenes

Compounds **7ab-a**, **7ab-b**, **7ac-a**, **7ac-b**, **7ag-a** and **7ag-b**

To a 12mL vial equipped with a magnetic stirring bar, bis(silyl)acetylene (0.03g, 1.0 eq.), toluene (100μL), olefin (1.2 eq.) and Karstedt catalyst ($2 \cdot 10^{-3}$ mol%) were added under argon atmosphere. Subsequently, reaction mixture was stirred at 60°C for a specified time. After this time, solvent and volatile residues were evaporated under a high vacuum. In the next step, the catalyst was precipitated by the addition of pentane or hexane (1mL) and filtered from the resulting mixture. Lastly, the solvent was separated under reduced pressure giving desired product. The pure products were identified by ^{1}H, ^{13}C, and ^{29}Si NMR spectroscopies and MS spectrometry.

CHARACTERISATION DATA FOR ALL PRODUCTS

Trimethyl((phenylsilyl)ethynyl)silane (3aa)

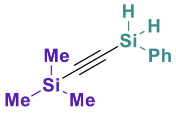

Trimethyl((phenylsilyl)ethynyl)silane was obtained as oil in 94% yield. The title compound was known in the literature,[2-5] and all spectroscopic data are in agreement.[5]

^{1}H NMR (600 MHz, $CDCl_3$) δ (ppm) = 0.28 (s, 9H), 4.64 (s, 2H), 7.42–7.51 (m, 3H), 7.69–7.74 (m, 2H).

^{13}C NMR (151 MHz, $CDCl_3$) δ (ppm) = –0.3, 103.6, 120.0, 128.2, 129.1, 130.2, 135.2.

^{29}Si NMR (79 MHz, $CDCl_3$) δ (ppm) = –61.7, –17.7.

EI-MS m/z (rel. int.): 205 (7.5%, $[M+H]^+$), 189 (100), 163 (15), 145 (25), 135 (53), 105 (18), 73 (30).

EA: $C_{11}H_{16}Si_2$ (204.079): calcd. C 64.63, H 7.89; found C 64.54, H 8.01.

Triethyl((phenylsilyl)ethynyl)silane (3ab)

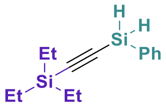

Triethyl((phenylsilyl)ethynyl)silane was obtained as oil in 99% yield. The title compound was known in the literature,[2] but it was isolated and characterized for the first time.

^{1}H NMR (600 MHz, $CDCl_3$) δ (ppm) = 0.71 (q, J = 7.9 Hz, 6H), 1.08 (t, J = 8.0 Hz, 9H), 4.65 (s, 2H), 7.41–7.51 (m, 3H), 7.70–7.75 (m, 2H).

^{13}C NMR (151 MHz, $CDCl_3$) δ (ppm) = 4.2, 7.4, 104.8, 117.7, 128.2, 129.3, 130.1, 135.2.

^{29}Si NMR (79 MHz, $CDCl_3$) δ (ppm) = –62.0, –7.0.

EI-MS m/z (rel. int.): 245 (3%, $[M–H]^+$), 233 (5), 217 (79), 203 (5), 189 (100), 161 (67), 129 (11), 105 (29), 81 (23), 53 (20).

EA: $C_{14}H_{22}Si_2$ (246.126): calcd. C 68.22, H 9.00; found C 68.25, H 8.98.

Triisopropyl((phenylsilyl)ethynyl)silane (3ac)

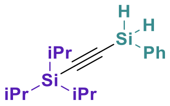

Triisopropyl((phenylsilyl)ethynyl)silane was obtained as oil in 99% yield. The title compound was known in the literature,[2] but it was isolated and characterized for the first time.

^{1}H NMR (600 MHz, $CDCl_3$) δ (ppm) = 1.17 (s, 21H), 4.66 (s, 2H), 7.41–7.50 (m, 3H), 7.71–7.76 (m, 2H).

^{13}C NMR (151 MHz, $CDCl_3$) δ (ppm) = 11.1, 18.6, 105.3, 116.8, 128.1, 129.4, 130.1, 135.1.

^{29}Si NMR (79 MHz, $CDCl_3$) δ (ppm) = –62.3, –1.7.

EI-MS m/z (rel. int.): 288 (3%, $[M–H]^+$), 245 (74), 217 (84), 203 (100), 189 (80), 175 (62), 157 (12), 135 (16), 121 (20), 105 (33), 78 (16), 53 (10).

EA: $C_{17}H_{28}Si_2$ (288.173): calcd. C 70.76, H 9.78; found C 70.75, H 9.74.

Diisopropyl((phenylsilyl)ethynyl)silane (3ad)

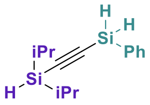

Diisopropyl((phenylsilyl)ethynyl)silane was obtained as oil in 89% yield. The title compound was previously unknown.

^{1}H NMR (600 MHz, $CDCl_3$) δ (ppm) = 1.12–1.21 (m, 14H), 3.82 (s, 1H), 4.67 (s, 2H), 7.42–7.55 (m, 3H), 7.70–7.83 (m, 2H).

^{13}C NMR (151 MHz, $CDCl_3$) δ (ppm) = 10.7, 18.3, 18.5, 106.9, 113.7, 128.2, 128.9, 130.2, 135.2.

^{29}Si NMR (79 MHz, $CDCl_3$) δ (ppm) = –61.8, –15.9.

EI-MS m/z (rel. int.): 247 (3%, $[M+H]^+$), 203 (73), 191 (4), 175 (100), 161 (24), 145 (13), 131 (9), 121 (29), 105 (66), 97 (6), 81 (11), 69 (10), 53 (8).

EA: $C_{14}H_{22}Si_2$ (246.126): calcd. C 68.22, H 9.00; found C 68.31, H 9.07.

((Phenylsilyl)ethynyl)tripropylsilane (3ae)

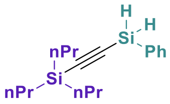

((Phenylsilyl)ethynyl)tripropylsilane was obtained as oil in 90% yield. The title compound was previously unknown.

^{1}H NMR (600 MHz, $CDCl_3$) δ (ppm) = 0.66–0.74 (m, 6H), 1.00–1.08 (m, 9H), 1.44–1.55 (m, 6H), 4.62–4.66 (m, 2H), 7.41–7.51 (m, 3H), 7.69–7.74 (m, 2H).

^{13}C NMR (151 MHz, $CDCl_3$) δ (ppm) = 15.8, 17.5, 18.2, 104.7, 118.6, 128.1, 129.3, 130.1, 135.2.

^{29}Si NMR (79 MHz, $CDCl_3$) δ (ppm) = –62.1, –12.9.

EI-MS m/z (rel. int.): 288 (2%, $[M–H]^+$), 245 (9), 219 (9), 203 (100), 175 (13), 161 (7), 105 (12), 78 (4), 53 (4).

EA: $C_{17}H_{28}Si_2$ (288.173): calcd. C 70.76, H 9.78; found C 70.64, H 9.72.

Triisobutyl((phenylsilyl)ethynyl)silane (3af)

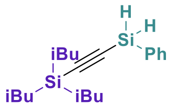

Triisobutyl((phenylsilyl)ethynyl)silane was obtained as oil in 99% yield. The title compound was previously unknown.

^{1}H NMR (600 MHz, $CDCl_3$) δ (ppm) = 0.69–0.76 (m, 6H), 1.01–1.08 (m, 18H), 1.89–1.99 (m, 3H), 4.61–4.65 (m, 2H), 7.40–7.51 (m, 3H), 7.68–7.74 (m, 2H).

^{13}C NMR (151 MHz, $CDCl_3$) δ (ppm) = 24.7, 25.1, 26.3, 105.2, 119.9, 128.1, 129.3, 130.1, 135.2.

^{29}Si NMR (79 MHz, $CDCl_3$) δ (ppm) = –62.1, –16.3.

EI-MS m/z (rel. int.): 273 (27%, $[M–iBu]^+$), 233 (8), 217 (100), 189 (10), 175 (9), 161 (6), 121 (5), 105 (11).

EA: $C_{20}H_{34}Si_2$ (330.220): calcd. C 72.65, H 10.36 found C 72.55, H 10.35.

Tert-butyldimethyl((phenylsilyl)ethynyl)silane (3ag)

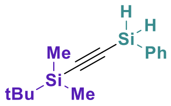

Tert-butyldimethyl((phenylsilyl)ethynyl)silane was obtained as oil in 96% yield. The title compound was previously unknown.

^{1}H NMR (400 MHz, $CDCl_3$) δ (ppm) = 0.20 (s, 6H), 1.02 (s, 9H), 4.63 (s, 2H), 7.39–7.52 (m, 3H), 7.67-7.75 (m, 2H).

^{13}C NMR (101 MHz, $CDCl_3$) δ (ppm) = –4.8, 16.5, 26.0, 104.2, 118.5, 128.2, 129.2, 130.2, 135.2.

^{29}Si NMR (79 MHz, $CDCl_3$) δ (ppm) = –61.9, –7.9.

EI-MS m/z (rel. int.): 247 (3%, $[M+H]^+$), 231 (1), 203 (2), 189 (100), 173 (3), 163 (4), 145 (11), 135 (31), 129 (3), 121 (6), 111 (2), 105 (27), 95 (2), 83 (6), 73 (4), 69 (3), 57 (4).

EA: $C_{14}H_{22}Si_2$ (246.126): calcd. C 68.22, H 9.00; found C 68.27, H 9.01.

Dimethyl(octyl)((phenylsilyl)ethynyl)silane (3ah)

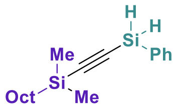

Dimethyl(octyl)((phenylsilyl)ethynyl)silane was obtained as oil in 99% yield. The title compound was previously unknown.

^{1}H NMR (600 MHz, $CDCl_3$) δ (ppm) = 0.20–0.27 (m, 6H), 0.65–0.75 (m, 2H), 0.89–0.98 (m, 3H), 1.29–1.47 (m, 12H), 4.61–4.66 (m, 2H), 7.39–7.51 (m, 3H), 7.67–7.74 (m, 2H).

^{13}C NMR (151 MHz, $CDCl_3$) δ (ppm) = –1.9, 14.2, 15.8, 22.7, 23.7, 29.3, 29.3, 32.0, 33.3, 103.9, 119.6, 128.2, 129.1, 130.2, 135.2.

^{29}Si NMR (79 MHz, $CDCl_3$) δ (ppm) = –61.8, –15.8.

EI-MS m/z (rel. int.): 302 (5%, $[M–H]^+$), 287 (8), 259 (6), 233 (15), 217 (14), 189 (100), 175 (23), 135 (59), 105 (36), 73 (12) 59 (15), 53 (10).

EA: $C_{18}H_{28}Si_2$ (316.168): calcd. C 68.29, H 8.91; found C 68.33, H 8.99.

Dimethyl(phenyl)((phenylsilyl)ethynyl)silane (3ai)

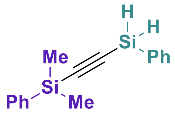

Dimethyl(phenyl)((phenylsilyl)ethynyl)silane was obtained as oil in 92% yield. The title compound was known in the literature,[2] but it was isolated and characterized for the first time.

^{1}H NMR (600 MHz, $CDCl_3$) δ (ppm) = 0.57 (s, 6H), 4.73 (s, 2H), 7.44–7.56 (m, 6H), 7.73–7.79 (m, 4H).

^{13}C NMR (151 MHz, $CDCl_3$) δ (ppm) = –1.0, 105.8, 117.7, 128.0, 128.1, 128.3, 128.9, 129.7, 129.7, 130.3, 133.8, 133.8, 134.9, 135.3, 136.1.

^{29}Si NMR (79 MHz, $CDCl_3$) δ (ppm) = –61.4, –21.9.

EI-MS m/z (rel. int.): 264 (28%, $[M–2H]^+$), 251 (100), 238 (4), 221 (7), 197 (15), 135 (22), 105 (21), 78 (7), 53 (9).

EA: $C_{16}H_{18}Si_2$ (266.195): calcd. C 72.11, H 6.81; found C 72.17, H 6.79.

Methyl(phenyl)((phenylsilyl)ethynyl)(vinyl)silane (3aj)

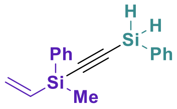

Methyl(phenyl)((phenylsilyl)ethynyl)(vinyl)silane was obtained as oil in 98% yield. The title compound was previously unknown.

^{1}H NMR (600 MHz, $CDCl_3$) δ (ppm) = 0.59–0.66 (m, 3H), 4.68–4.75 (m, 2H), 6.00–6.11 (m, 1H), 6.19–6.28 (m, 1H), 6.29–6.40 (m, 1H), 7.42–7.55 (m, 6H), 7.68–7.80 (m, 4H).

^{13}C NMR (151 MHz, $CDCl_3$) δ (ppm) = –2.8, 107.1, 115.5, 128.1, 128.3, 128.8, 129.9, 130.3, 134.0, 134.3, 134.9, 135.3, 135.5.

^{29}Si NMR (79 MHz, $CDCl_3$) δ (ppm) = –61.2, –28.8.

EI-MS m/z (rel. int.): 279 (14%, $[M+H]^+$), 263 (100), 251 (32), 233 (14), 200 (22), 185 (10), 171 (11), 159 (8), 146 (21), 131 (12), 121 (16), 105 (53), 78 (16), 53 (24).

EA: $C_{17}H_{18}Si_2$ (278.168): calcd. C 73.32, H 6.51; found C 73.17, H 6.48.

1,2-Bis(((phenylsilyl)ethynyl)dimethylsilyl)ethane (3ak)

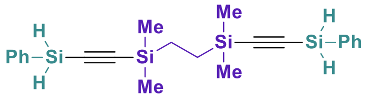

1,2-Bis(((phenylsilyl)ethynyl)dimethylsilyl)ethane was obtained as oil in 98% yield. The title compound was previously unknown.

^{1}H NMR (600 MHz, $CDCl_3$) δ (ppm) = 0.23–0.28 (m, 12H), 0.70–0.74 (m, 4H), 4.63–4.66 (m, 4H), 7.41–7.50 (m, 6H), 7.69–7.74 (m, 4H).

^{13}C NMR (151 MHz, $CDCl_3$) δ (ppm) = –2.5, 8.2, 104.3, 119.1, 128.2, 129.1, 130.2, 135.2.

^{29}Si NMR (119 MHz, $CDCl_3$) δ (ppm) = –61.7, –13.6.

EI-MS m/z (rel. int.): 406 (28%, $[M–H]^+$), 391 (29), 375 (13), 363 (87), 347 (17), 327 (19), 313 (73), 299 (23), 285 (36), 275 (100), 259 (13), 189 (15), 145 (12), 135 (47), 121 (10), 105 (63), 53 (12).

EA: $C_{22}H_{30}Si_4$ (406.142): calcd. C 64.95, H 7.43; found C 65.02, H 7.49.

((Hexylsilyl)ethynyl)trimethylsilane (3ba)

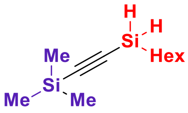

((Hexylsilyl)ethynyl)trimethylsilane was obtained as oil in 90% yield. The title compound was previously unknown.

^{1}H NMR (600 MHz, $CDCl_3$) δ (ppm) = 0.21 (s, 9H), 0.79–0.86 (m, 2H), 0.91 (t, *J* = 6.9 Hz, 3H), 1.27–1.34 (m, 4H), 1.35–1.42 (m, 2H), 1.45–1.52 (m, 2H), 3.94–3.97 (m, 2H).

^{13}C NMR (151 MHz, $CDCl_3$) δ (ppm) = –0.3, 9.7, 14.1, 22.5, 24.7, 31.5, 32.1, 105.3, 118.3.

^{29}Si NMR (119 MHz, $CDCl_3$) δ (ppm) = –58.4, –18.3.

EI-MS m/z (rel. int.): 197 (10%, $[M–Me]^+$), 169 (6), 155 (10), 141 (23), 138 (30), 127 (45), 113 (36), 99 (25), 83 (34), 73 (100), 59 (25).

EA: $C_{11}H_{24}Si_2$ (212.142): calcd. C 62.18, H 11.39; found C 62.22, H 11.42.

Triethyl((hexylsilyl)ethynyl)silane (3bb)

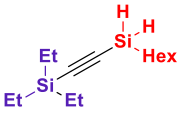

Triethyl((hexylsilyl)ethynyl)silane was obtained as oil in 98% yield. The title compound was previously unknown.

^{1}H NMR (600 MHz, $CDCl_3$) δ (ppm) = 0.64 (q, *J* = 7.9 Hz, 6H), 0.79–0.86 (m, 2H), 0.91 (t, *J* = 6.9 Hz, 3H), 1.03 (t, *J* = 7.9 Hz, 9H), 1.27–1.35 (m, 4H), 1.36–1.43 (m, 2H), 1.47–1.54 (m, 2H), 3.95–3.99 (m, 2H).

^{13}C NMR (151 MHz, $CDCl_3$) δ (ppm) = 4.2, 7.4, 9.8, 14.1, 22.5, 24.8, 31.5, 32.1, 106.5, 115.9.

^{29}Si NMR (119 MHz, $CDCl_3$) δ (ppm) = –58.6, –7.6.

EI-MS m/z (rel. int.): 225 (100%, $[M–Et]^+$), 197 (19), 169 (15), 155 (11), 141 (41), 137 (29), 127 (23), 113 (65), 99 (16), 83 (67), 69 (13), 59 (28).

EA: $C_{14}H_{30}Si_2$ (254.189): calcd. C 66.06, H 11.88; found C 66.08, H 11.97.

((Hexylsilyl)ethynyl)triisopropylsilane (3bc)

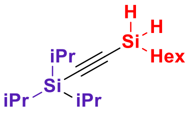

((Hexylsilyl)ethynyl)triisopropylsilane was obtained as oil in 99% yield. The title compound was previously unknown.

^{1}H NMR (600 MHz, $CDCl_3$) δ (ppm) = 0.80–0.86 (m, 2H), 0.91 (t, J = 6.9 Hz, 3H), 1.08–1.14 (m, 21H), 1.28–1.35 (m, 4H), 1.36–1.43 (m, 2H), 1.48–1.55 (m, 2H), 3.97–3.99 (m, 2H).

^{13}C NMR (151 MHz, $CDCl_3$) δ (ppm) = 9.9, 11.1, 14.1, 18.5, 22.5, 24.8, 31.5, 32.1, 107.1, 114.8.

^{29}Si NMR (119 MHz, $CDCl_3$) δ (ppm) = –58.8, –2.2.

EI-MS m/z (rel. int.): 296 (4%, $[M–H]^+$), 253 (63), 211 (10), 183 (10), 169 (34), 157 (100), 141 (41), 127 (36), 113 (37), 99 (11), 73 (7), 59 (8).

EA: $C_{17}H_{36}Si_2$ (296.236): calcd. C 68.83, H 12.23; found C 68.88, H 12.22.

((Hexylsilyl)ethynyl)diisopropylsilane (3bd)

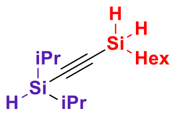

((Hexylsilyl)ethynyl)diisopropylsilane was obtained as oil in 69% yield. The title compound was previously unknown.

^{1}H NMR (600 MHz, $CDCl_3$) δ (ppm) = 0.80–0.87 (m, 2H), 0.91 (t, J = 6.8 Hz, 3H), 1.01–1.14 (m, 14H), 1.26–1.36 (m, 4H), 1.37–1.44 (m, 2H), 1.48–1.56 (m, 2H), 3.72 (s, 1H), 3.95–4.01 (m, 2H).

^{13}C NMR (151 MHz, $CDCl_3$) δ (ppm) = 9.7, 10.6, 14.1, 18.2, 18.4, 22.5, 24.8, 31.5, 32.1, 108.7, 111.8.

^{29}Si NMR (119 MHz, $CDCl_3$) δ (ppm) = –58.4, –16.3.

EI-MS m/z (rel. int.): 255 (2%, $[M+H]^+$), 211 (34), 183 (10), 169 (14), 155 (16), 141 (35), 127 (100), 113 (41), 99 (54), 85 (29), 69 (20), 59 (15).

EA: $C_{14}H_{30}Si_2$ (254.189): calcd. C 66.06, H 11.88; found C 66.11, H 11.92.

((Hexylsilyl)ethynyl)tripropylsilane (3be)

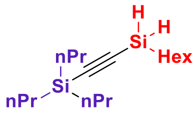

((Hexylsilyl)ethynyl)tripropylsilane was obtained as oil in 99% yield. The title compound was previously unknown.

^{1}H NMR (600 MHz, $CDCl_3$) δ (ppm) = 0.61–0.67 (m, 6H), 0.79–0.85 (m, 2H), 0.92 (t, J = 6.9 Hz, 3H), 1.01 (t, J = 7.3 Hz, 9H), 1.27–1.35 (m, 4H), 1.36–1.41 (m, 2H), 1.42–1.47 (m, 6H), 1.48–1.54 (m, 2H), 3.94–3.98 9m, 2H).

^{13}C NMR (151 MHz, $CDCl_3$) δ (ppm) = 9.8, 14.1, 15.8, 17.4, 18.2, 22.5, 24.8, 31.5, 32.1, 106.5, 116.7.

^{29}Si NMR (119 MHz, $CDCl_3$) δ (ppm) = –58.7, –13.4.

EI-MS m/z (rel. int.): 297 (14%, $[M+H]^+$), 269 (13), 253 (64), 227 (11), 211 (39), 183 (12), 169 (48), 157 (29), 141 (72), 127 (100), 113 (43), 99 (29), 83 (11).

EA: $C_{17}H_{36}Si_2$ (296.236): calcd. C 68.83, H 12.23; found C 68.77, H 12.20.

((Hexylsilyl)ethynyl)triisobutylsilane (3bf)

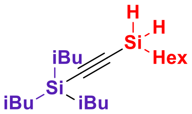

((Hexylsilyl)ethynyl)triisobutylsilane was obtained as oil in 98% yield. The title compound was previously unknown.

^{1}H NMR (600 MHz, $CDCl_3$) δ (ppm) = 0.67 (d, J = 7.0 Hz, 6H), 0.78–0.83 (m, 2H), 0.92 (t, J = 6.9 Hz, 3H), 1.01 (d, J = 6.7 Hz, 18H), 1.27–1.35 (m, 4H), 1.36–1.42 (m, 2H), 1.46–1.53 (m, 2H), 1.85–1.93 (m, 3H), 3.93–3.97 (m, 2H).

^{13}C NMR (151 MHz, $CDCl_3$) δ (ppm) = 9.8, 14.1, 22.5, 24.8, 25.0, 26.2, 31.5, 32.2, 107.1, 118.0.

^{29}Si NMR (119 MHz, $CDCl_3$) δ (ppm) = –58.7, –16.9.

EI-MS m/z (rel. int.): 338 (2%, $[M–H]^+$), 281 (41), 241 (16), 225 (60), 199 (30), 183 (14), 169 (31), 155 (54), 141 (100), 127 (23), 113 (76), 99 (9), 85 (13), 59 (11).

EA: $C_{20}H_{42}Si_2$ (338.283): calcd. C 70.92, H 12.50; found C 70.81, H 12.47.

Tert-butyl((hexylsilyl)ethynyl)dimethylsilane (3bg)

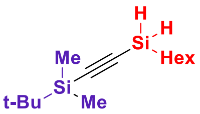

Tert-butyl((hexylsilyl)ethynyl)dimethylsilane was obtained as oil in 97% yield. The title compound was previously unknown.

^{1}H NMR (400 MHz, $CDCl_3$) δ (ppm) = 0.15 (s, 6H), 0.78–0.87 (m, 2H), 0.89–0.94 (m, 3H), 0.97 (s, 9H), 1.26–1.54 (m, 8H), 3.93–3.99 (m, 2H).

^{13}C NMR (101 MHz, $CDCl_3$) δ (ppm) = –4.8, 9.7, 14.1, 16.4, 22.5, 24.8, 26.0, 31.5, 32.1, 106.0, 116.7.

^{29}Si NMR (79 MHz, $CDCl_3$) δ (ppm) = –58.6, –8.4.

EI-MS m/z (rel. int.): 255 (3%, $[M+H]^+$), 197 (100), 169 (6), 155 (10), 151 (11), 141 (27), 137 (17), 127 (25), 113 (23), 97 (13), 85 (15), 73 (15), 69 (4), 59 (16).

EA: $C_{14}H_{30}Si_2$ (254.189): calcd. C 66.06, H 11.88; found C 66.16, H 11.95.

((Hexylsilyl)ethynyl)dimethyl(octyl)silane (3bh)

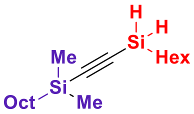

((Hexylsilyl)ethynyl)dimethyl(octyl)silane was obtained as oil in 90% yield. The title compound was previously unknown.

^{1}H NMR (600 MHz, $CDCl_3$) δ (ppm) = 0.18 (s, 6H), 0.62-0.67 (m, 2H), 0.80–0.86 (m, 2H), 0.89–0.94 (m, 6H), 1.26–1.34 (m, 12H), 1.35–1.44 (m, 6H), 1.46–1.54 (m, 2H), 3.94–3.98 (m, 2H).

^{13}C NMR (151 MHz, $CDCl_3$) δ (ppm) = –1.9, 9.7, 14.1, 15.9, 22.5, 22.7, 23.7, 24.8, 29.3, 29.3, 31.5, 32.0, 32.1, 33.2, 105.7, 117.9.

^{29}Si NMR (119 MHz, $CDCl_3$) δ (ppm) = –58.5, –16.3.

EI-MS m/z (rel. int.): 295 (5%, $[M–Me]^+$), 255 (12), 241 (28), 225 (17), 211 (12), 197 (15), 183 (13), 169 (30), 155 (40), 141 (75), 127 (100), 113 (60), 99 (22), 85 (20), 73 (30), 59 (45).

EA: $C_{18}H_{38}Si_2$ (310.251): calcd. C 69.59, H 12.33; found C 69.70, H 12.40.

((Hexylsilyl)ethynyl)dimethyl(phenyl)silane (3bi)

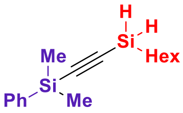

((Hexylsilyl)ethynyl)dimethyl(phenyl)silane was obtained as oil in 88% yield. The title compound was previously unknown.

^{1}H NMR (600 MHz, $CDCl_3$) δ (ppm) = 0.49 (s, 1H), 0.85–0.91 (m, 2H), 0.94 (t, J = 6.8 Hz, 3H), 1.30–1.38 (m, 4H), 1.39–1.46 (m, 2H), 1.50–1.57 (m, 2H), 4.00–4.04 (m, 2H), 7.39–7.47 (m, 3H), 7.66–7.71 (m, 2H).

^{13}C NMR (151 MHz, $CDCl_3$) δ (ppm) = –1.0, 9.7, 14.1, 22.5, 24.8, 31.5, 32.1, 107.5, 116.0, 127.9, 129.6, 133.7, 136.3.

^{29}**Si NMR** (119 MHz, $CDCl_3$) δ (ppm) = –58.0, –22.4.

EI-MS m/z (rel. int.): 274 (6%, $[M–H]^+$), 259 (46), 231 (8), 215 (11), 205 (20), 190 (100), 175 (51), 159 (22), 145 (48), 135 (57), 121 (41), 105 (51), 93 (11), 85 (12), 73 (11), 59 (13), 53 (15).

EA: $C_{16}H_{26}Si_2$ (274.157): calcd. C 70.00, H 9.55; found C 69.91, H 9.46.

((Hexylsilyl)ethynyl)(methyl)(phenyl)(vinyl)silane (3bj)

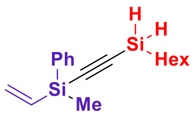

((Hexylsilyl)ethynyl)(methyl)(phenyl)(vinyl)silane was obtained as oil in 89% yield. The title compound was previously unknown.

^{1}**H NMR** (600 MHz, $CDCl_3$) δ (ppm) = 0.57 (s, 3H), 0.86–0.91 (m, 2H), 0.94 (t, *J* = 6.8 Hz, 3H), 1.30–1.39 (m, 4H), 1.40–1.47 (m, 2H), 1.51–1.59 (m, 2H), 4.03–4.05 (m, 2H), 6.00 (dd, *J* = 19.9, 3.8 Hz, 1H), 6.20 (dd, *J* = 14.4, 3.8 Hz, 1H), 6.30 (dd, *J* = 19.9, 14.4 Hz, 1H), 7.39–7.48 (m, 3H), 7.66–7.71 (m, 2H).

^{13}**C NMR** (151 MHz, $CDCl_3$) δ (ppm) = –2.7, 9.6, 14.1, 22.6, 24.8, 31.5, 32.2, 108.9, 113.8, 128.0, 129.8, 134.2, 134.2, 134.5, 135.2.

^{29}**Si NMR** (119 MHz, $CDCl_3$) δ (ppm) = –57.9, –29.2.

EI-MS m/z (rel. int.): 287 (24%, $[M+H]^+$), 271 (14), 243 (7), 229 (9), 215 (17), 202 (100), 187 (56), 174 (48), 157 (29), 146 (66), 131 (24), 121 (45), 105 (51), 85 (15), 53 (22).

EA: $C_{17}H_{26}Si_2$ (286.157): calcd. C 71.25, H 9.15; found C 71.09, H 9.01.

1,2-Bis(((hexylsilyl)ethynyl)dimethylsilyl)ethane (3bk)

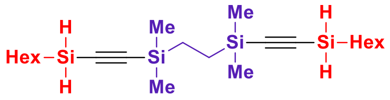

1,2-Bis(((hexylsilyl)ethynyl)dimethylsilyl)ethane was obtained as oil in 95% yield. The title compound was previously unknown.

^{1}**H NMR** (600 MHz, $CDCl_3$) δ (ppm) = 0.18–0.20 (m, 12H), 0.63 (s, 4H), 0.80–0.85 (m, 4H), 0.91 (t, *J* = 6.9 Hz, 6H), 1.29–1.33 (m, 8H), 1.37–1.41 (m, 4H), 1.46–1.52 (m, 4H), 3.93–3.99 (m, 3H).

^{13}**C NMR** (151 MHz, $CDCl_3$) δ (ppm) = –2.5, 8.1, 9.7, 14.1, 22.5, 24.7, 31.5, 32.1, 105.9, 117.4.

^{29}**Si NMR** (119 MHz, $CDCl_3$) δ (ppm) = –58.5, –14.3.

EI-MS m/z (rel. int.): 337 (3%, [M–Hex]$^+$), 323 (4), 307 (6), 295 (4), 281 (6), 267 (7), 254 (100), 239 (80), 227 (30), 209 (28), 197 (25), 182 (14), 169 (12), 154 (10), 141 (29), 127 (30), 112 (46), 96 (28), 85 (31), 73 (26), 69 (8), 59 (43), 55 (10).

EA: $C_{22}H_{46}Si_4$ (422.268): calcd. C 62.48, H 10.96; found C 62.46, H 11.01.

Triisopropyl((p-tolylsilyl)ethynyl)silane (3cc)

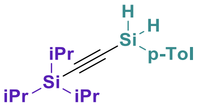

Triisopropyl((p-tolylsilyl)ethynyl)silane was obtained as oil in 99% yield. The title compound was previously unknown.

^{1}H NMR (400 MHz, $CDCl_3$) δ (ppm) = 1.16 (s, 21H), 2.42 (s, 3H), 4.64 (s, 2H), 7.22–7.31 (m, 2H), 7.55–7.67 (m, 2H).

^{13}C NMR (101 MHz, $CDCl_3$) δ (ppm) = 11.1, 18.6, 21.6, 105.7, 116.4, 125.6, 129.0, 135.2, 140.2.

^{29}Si NMR (79 MHz, $CDCl_3$) δ (ppm) = –62.5, –1.80.

EI-MS m/z (rel. int.): 302 (4%, [M–H]$^+$), 259 (100), 231 (32), 217 (24), 203 (32), 189 (35), 173 (6), 159 (10), 149 (10), 135 (14), 119 (27), 101 (6), 59 (3).

EA: $C_{18}H_{30}Si_2$ (302.189): calcd. C 71.44, H 9.99; found C 71.50, H 9.91.

[Bis[2-(trimethylsilyl)ethynyl]silyl]benzene (4aa)

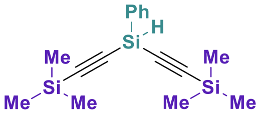

[Bis[2-(trimethylsilyl)ethynyl]silyl]benzene was obtained as oil in 99% yield. The title compound was known in the literature,[2] but it was isolated and characterized for the first time.

^{1}H NMR (600 MHz, $CDCl_3$) δ (ppm) = 0.22–0.30 (m, 18H), 4.86–4.90 (m, 1H), 7.39–7.53 (m, 3H), 7.75–7.80 (m, 2H).

^{13}C NMR (151 MHz, $CDCl_3$) δ (ppm) = –0.3, 104.1, 119.0, 128.2, 130.2, 130.4, 134.8.

^{29}Si NMR (79 MHz, $CDCl_3$) δ (ppm) = –67.4, –17.5.

EI-MS m/z (rel. int.): 300 (9%, [M–H]$^+$), 285 (100), 259 (14), 243 (10), 135 (25), 105 (10), 73 (37), 45 (14).

EA: $C_{16}H_{24}Si_3$ (300.119): calcd. C 63.93, H 8.05; found C 64.04, H 7.99.

[Bis[2-(triethylsilyl)ethynyl]silyl]benzene (4ab)

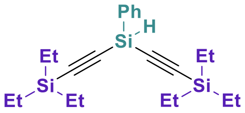

[Bis[2-(triethylsilyl)ethynyl]silyl]benzene was obtained as oil in 99% yield. The title compound was known in the literature,[2] but it was isolated and characterized for the first time.

^{1}H NMR (400 MHz, $CDCl_3$) δ (ppm) = 0.69 (q, *J* = 7.9 Hz, 12H), 1.06 (t, *J* = 7.9 Hz, 18H), 4.88 (s, 1H), 7.40–7.51 (m, 3H), 7.76–7.82 (m, 2H).

^{13}C NMR (101 MHz, $CDCl_3$) δ (ppm) = 4.2, 7.4, 105.8, 116.6, 128.1, 130.3, 130.5, 134.8.

^{29}Si NMR (79 MHz, $CDCl_3$) δ (ppm) = –67.8, –6.8.

EI-MS m/z (rel. int.): 384 (16%, $[M–H]^+$), 355 (100), 327 (31), 299 (22), 271 (17), 243 (18), 215 (16), 187 (9), 163 (16), 149 (13), 135 (16), 121 (14), 105 (20).

EA: $C_{22}H_{36}Si_3$ (384.212): calcd. C 68.67, H 9.43; found C 68.64, H 9.44.

[Bis[2-(tripropylsilyl)ethynyl]silyl]benzene (4ae)

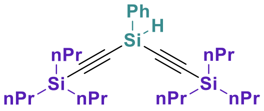

[Bis[2-(tripropylsilyl)ethynyl]silyl]benzene was obtained as oil in 97% yield. The title compound was previously unknown.

^{1}H NMR (400 MHz, $CDCl_3$) δ (ppm) = 0.68 (p, 12H), 1.02 (t, *J* = 7.3 Hz, 18H), 1.41–1.54 (m, 12H), 4.85 (s, 1H), 7.38–7.51 (m, 3H), 7.73–7.80 (m, 2H).

^{13}C NMR (101 MHz, $CDCl_3$) δ (ppm) = 15.7, 17.4, 18.2, 105.7, 117.3, 128.0, 130.2, 130.6, 134.8.

^{29}Si NMR (79 MHz, $CDCl_3$) δ (ppm) = –67.8, –12.8.

EI-MS m/z (rel. int.): 468 (4%, $[M–H]^+$), 425 (28), 383 (81), 341 (89), 299 (100), 257 (93), 241 (5), 229 (23), 215 (72), 201 (11), 191 (15), 185 (21), 173 (17), 159 (25), 149 (22), 142 (32), 135 (53), 131 (17), 121 (74), 114 (14), 105 (38), 97 (10), 81 (9), 73 (9), 69 (9), 59 (5).

EA: $C_{28}H_{48}Si_3$ (468.306): calcd. C 71.72, H 10.32; found C 71.67, H 10.24.

[Bis[2-(tert-butyl(dimethyl)silyl)ethynyl]silyl]benzene (4ag)

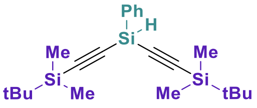

[Bis[2-(tert-butyl(dimethyl)silyl)ethynyl]silyl]benzene was obtained as oil in 92% yield. The title compound was previously unknown.

^{1}H NMR (400 MHz, $CDCl_3$) δ (ppm) = 0.19 (d, *J* = 1.1 Hz, 12H), 1.01 (s, 18H), 4.87 (s, 1H), 7.38–7.53 (m, 3H), 7.73–7.80 (m, 2H).

^{13}C NMR (101 MHz, $CDCl_3$) δ (ppm) = –4.9, 16.6, 26.0, 105.1, 117.4, 128.1, 130.3, 134.7.

^{29}Si NMR (79 MHz, $CDCl_3$) δ (ppm) = –67.6, –7.7.

EI-MS m/z (rel. int.): 384 (8%, $[M–H]^+$), 327 (100), 285 (18), 271 (12), 255 (24), 239 (18), 229 (18), 217 (20), 203 (19), 191 (12), 177 (12), 159 (22), 145 (22), 135 (76), 129 (12), 105 (19), 83 (14), 73 (53), 57 (18).

EA: $C_{22}H_{36}Si_3$ (384.212): calcd. C 68.67, H 9.43; found C 68.77, H 9.44.

[Bis[2-(dimethyl(phenyl)silyl)ethynyl]silyl]benzene (4ai)

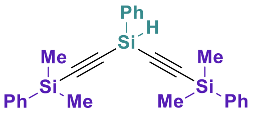

[Bis[2-(dimethyl(phenyl)silyl)ethynyl]silyl]benzene was obtained as oil in 99% yield. The title compound was previously unknown.

^{1}H NMR (400 MHz, $CDCl_3$) δ (ppm) = 0.54 (s, 12H), 4.97 (s, 1H), 7.40–7.53 (m, 10H), 7.70–7.74 (m, 3H), 7.79–7.85 (m, 2H).

^{13}C NMR (101 MHz, $CDCl_3$) δ (ppm) = –1.1, 106.1, 116.9, 128.0, 128.2, 129.7, 130.5, 133.8, 134.9, 136.0.

^{29}Si NMR (79 MHz, $CDCl_3$) δ (ppm) = –66.9, –21.5.

EI-MS m/z (rel. int.): 424 (63%, $[M–H]^+$), 409 (61), 365 (10), 346 (15), 331 (48), 289 (14), 279 (20), 264 (18), 249 (17), 221 (30), 207 (14), 197 (100), 181 (11), 159 (25), 145 (48), 135 (95), 129 (26), 119 (12), 105 (58).

EA: $C_{26}H_{28}Si_3$ (424.150): calcd. C 73.52, H 6.64; found C 73.47, H 6.54.

[(Diphenylsilyl)ethynyl]triisopropylsilane (3dc)

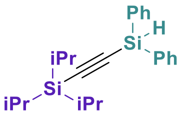

((Diphenylsilyl)ethynyl)triisopropylsilane was obtained as oil in 71% yield. The title compound was known in the literature, [2,6] and all spectroscopic data are in agreement. [6]

^{1}H NMR (400 MHz, $CDCl_3$) δ (ppm) = 1.01–1.21 (m, 21H), 5.23 (s, 1H), 7.38–7.50 (m, 7H), 7.72–7.78 (m, 3H).

^{13}C NMR (101 MHz, $CDCl_3$) δ (ppm) = 11.2, 18.7, 107.9, 116.6, 128.1, 130.0, 132.5, 134.4, 135.1, 135.9.

^{29}Si NMR (79 MHz, $CDCl_3$) δ (ppm) = –42.1, –1.7.

EI-MS m/z (rel. int.): 364 (3%, $[M–H]^+$), 321 (100), 293 (17), 279 (12), 265 (17), 251 (21), 237 (6), 207 (7), 197 (21), 183 (11), 159 (5), 145 (10), 135 (17), 129 (5), 121 (11), 105 (38), 95 (1), 78 (4), 69 (2), 59 (3), 53 (2).

EA: $C_{23}H_{32}Si_2$ (364.204): calcd. C 77.75, H 8.85; found C 77.64, H 8.84.

[(Methyl(phenyl)silyl)ethynyl]triisopropylsilane (3ec)

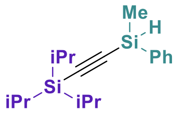

[(Methyl(phenyl)silyl)ethynyl]triisopropylsilane was obtained as oil in 89% yield. The title compound was previously unknown.

^{1}H NMR (400 MHz, $CDCl_3$) δ (ppm) = 0.50–0.59 (m, 3H), 1.08–1.22 (m, 21H), 4.69–4.76 (m, 1H), 7.41–7.47 (m, 3H), 7.68–7.79 (m, 2H).

^{13}C NMR (101 MHz, $CDCl_3$) δ (ppm) = –3.3, 11.2, 18.6, 110.1, 114.6, 128.0, 129.7, 134.4.

^{29}Si NMR (79 MHz, $CDCl_3$) δ (ppm) = –38.7, –2.1.

EI-MS m/z (rel. int.): 302 (3%, $[M–H]^+$), 287 (1), 259 (100), 243 (1), 231 (32), 217 (30), 203 (37), 189 (39), 175 (9), 159 (11), 145 (18), 135 (37), 131 (5), 121 (17), 105 (25), 101 (4), 95 (3), 83 (4), 73 (7), 59 (4), 53 (2).

EA: $C_{18}H_{30}Si_2$ (302.189): calcd. C 71.44, H 9.99; found C 71.53, H 9.94.

[(Methyl(p-tolyl)silyl)ethynyl]triisopropylsilane (3fc)

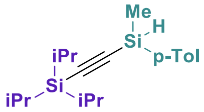

[(Methyl(p-tolyl)silyl)ethynyl]triisopropylsilane was obtained as oil in 73% yield. The title compound was previously unknown.

^{1}H NMR (400 MHz, $CDCl_3$) δ (ppm) = 0.46–0.58 (m, 3H), 1.16 (s, 21H), 2.42 (s, 3H), 4.66–4.73 (m, 1H), 7.17–7.33 (m, 2H), 7.56–7.70 (m, 2H).

^{13}**C NMR** (101 MHz, $CDCl_3$) δ (ppm) = –3.2, 11.1, 18.6, 21.5, 110.4, 113.6, 128.8, 130.2, 134.5, 139.2.

^{29}**Si NMR** (79 MHz, $CDCl_3$) δ (ppm) = –41.0, –2.2.

EI-MS m/z (rel. int.): 316 (4%, $[M–H]^+$), 301 (1), 273 (100), 257 (1), 245 (25), 231 (23), 217 (27), 203 (33), 189 (8), 173 (8), 159 (16), 149 (30), 145 (7), 135 (15), 119 (25), 109 (6), 101 (2), 93 (5), 83 (5), 73 (7), 59 (5), 53 (2).

EA: $C_{19}H_{32}Si_2$ (316.204): calcd. C 72.07, H 10.19; found C 72.13, H 10.28.

[(Methyl(octyl)silyl)ethynyl]triisopropylsilane (3gc)

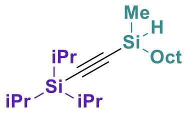

[(Methyl(octyl)silyl)ethynyl]triisopropylsilane was obtained as oil in 71% yield. The title compound was previously unknown.

^{1}**H NMR** (400 MHz, $CDCl_3$) δ (ppm) = –0.07–0.51 (m, 3H), 0.69–1.46 (m, 38H), 4.08 (s, 1H).

^{13}**C NMR** (101 MHz, $CDCl_3$) δ (ppm) = –4.5, 11.1, 13.5, 14.1, 18.6, 22.7, 24.2, 29.2, 29.4, 32.0, 32.9, 112.0, 112.6.

^{29}**Si NMR** (79 MHz, $CDCl_3$) δ (ppm) = –36.0, –2.5.

EI-MS m/z (rel. int.): 338 (1%, $[M–H]^+$), 295 (100), 267 (5), 253 (18), 239 (1), 225 (11), 211 (8), 197 (2), 183 (41), 179 (15), 167 (9), 155 (40), 141 (45), 127 (42), 113 (44), 97 (19), 85 (14), 73 (23), 59 (21), 55 (3).

EA: $C_{20}H_{42}Si_2$ (338.283): calcd. C 70.92, H 12.50; found C 71.01, H 12.52.

[[2-(Triethylsilyl)ethynyl][2-(trimethylsilyl)ethynyl]silyl]benzene (5ab-a)

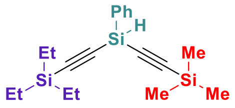

[[2-(Triethylsilyl)ethynyl][2-(trimethylsilyl)ethynyl]silyl]benzene was obtained as oil in 99% yield. The title compound was previously unknown.

^{1}**H NMR** (400 MHz, $CDCl_3$) δ (ppm) = 0.25 (s, 9H), 0.69 (q, J = 7.9 Hz, 6H), 1.06 (t, J = 7.9 Hz, 9H), 4.88 (s, 1H), 7.39–7.53 (m, 3H), 7.74–7.89 (m, 2H).

^{13}**C NMR** (101 MHz, $CDCl_3$) δ (ppm) = –0.4, 4.2, 7.4, 104.4, 105.5, 116.9, 118.8, 128.1, 130.3, 134.8.

^{29}**Si NMR** (79 MHz, $CDCl_3$) δ (ppm) = –67.6, –17.5, –6.8.

EI-MS m/z (rel. int.): 342 (3%, $[M–H]^+$), 327 (5), 313 (100), 299 (3), 285 (46), 257 (52), 225 (3), 207 (3), 135 (6), 111 (4), 105 (8), 73 (4), 59 (6), 53 (6), 45 (7).

EA: $C_{19}H_{30}Si_3$ (342.166): calcd. C 66.59, H 8.82; found C 66.50, H 8.85.

[[2-(Triethylsilyl)ethynyl][2-(tri-n-propylsilyl)ethynyl]silyl]benzene (5ab-e)

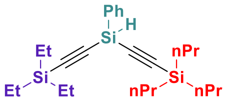

[[2-(Triethylsilyl)ethynyl][2-(tri-n-propylsilyl)ethynyl]silyl]benzene was obtained as oil in 96% yield. The title compound was previously unknown.

^{1}H NMR (400 MHz, $CDCl_3$) δ (ppm) = 0.61–0.74 (m, 12H), 1.04 (dt, *J* = 13.1, 7.6 Hz, 18H), 1.41–1.55 (m, 6H), 4.87 (s, 1H), 7.39–7.51 (m, 3H), 7.74–7.81 (m, 2H).

^{13}C NMR (101 MHz, $CDCl_3$) δ (ppm) = 4.2, 7.4, 15.7, 17.4, 18.2, 105.7, 105.8, 116.5, 117.4, 128.0, 130.3, 130.5, 134.8.

^{29}Si NMR (79 MHz, $CDCl_3$) δ (ppm) = –67.8, –12.7, –6.8.

EI-MS m/z (rel. int.): 426 (10%, $[M–H]^+$), 397 (6), 383 (50), 355 (24), 341 (100), 313 (53), 299 (89), 285 (16), 271 (64), 257 (11), 243 (44), 229 (10), 215 (45), 199 (8), 185 (23), 177 (20), 173 (15), 159 (31), 149 (24), 142 (51), 131 (25), 128 (49), 121 (17), 114 (26), 105 (53), 97 (9), 82 (17), 69 (9), 59 (12).

EA: $C_{19}H_{30}Si_3$ (426.259): calcd. C 70.34, H 9.92; found C 70.30, H 9.87.

[[2-(Tert-butyl(dimethyl)silyl)ethynyl][2-(triethylsilyl)ethynyl]silyl]benzene (5ab-g)

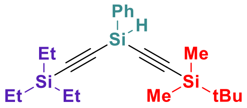

[[2-(Tert-butyl(dimethyl)silyl)ethynyl][2-(triethylsilyl)ethynyl]silyl]benzene was obtained as oil in 99% yield. The title compound was previously unknown.

^{1}H NMR (400 MHz, $CDCl_3$) δ (ppm) = 0.19 (s, 6H), 0.63–0.74 (q, *J* = 7.9 Hz, 6H), 1.01 (s, 9H), 1.02–1.10 (t, *J* = 7.9 Hz, 9H), 4.88 (s, 1H), 7.39–7.51 (m, 3H), 7.74–7.82 (m, 2H).

^{13}C NMR (101 MHz, $CDCl_3$) δ (ppm) = –4.9, 4.2, 7.4, 16.6, 26.0, 105.2, 105.7, 116.6, 117.3, 128.1, 130.3, 134.8.

^{29}Si NMR (79 MHz, $CDCl_3$) δ (ppm) = –67.7, –7.7, –6.8.

EI-MS m/z (rel. int.): 384 (5%, $[M–H]^+$), 355 (2), 327 (100), 299 (17), 271 (18), 257 (3), 243 (16), 225 (4), 213 (4), 199 (4), 189 (5), 173 (7), 159 (7), 145 (11), 135 (19), 121 (15), 111 (6), 105 (15), 96 (3), 83 (7), 73 (7), 59 (6), 57 (9).

EA: $C_{22}H_{36}Si_3$ (384.212): calcd. C 68.67, H 9.43; found C 68.56, H 9.37.

[[2-(Triisopropylsilyl)ethynyl][2-(trimethylsilyl)ethynyl]silyl]benzene (5ac-a)

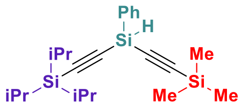

[[2-(Triisopropylsilyl)ethynyl][2-(trimethylsilyl)ethynyl]silyl]benzene was obtained as oil in 99% yield. The title compound was previously unknown.

^{1}H NMR (400 MHz, $CDCl_3$) δ (ppm) = 0.25 (s, 9H), 1.15 (s, 21H), 4.88 (s, 1H), 7.37–7.54 (m, 3H), 7.73–7.84 (m, 2H).

^{13}C NMR (101 MHz, $CDCl_3$) δ (ppm) = –0.4, 11.1, 18.6, 104.6, 106.3, 116.0, 118.6, 128.1, 130.3, 130.5, 134.8.

^{29}Si NMR (79 MHz, $CDCl_3$) δ (ppm) = –67.7, –17.5, –1.5.

EI-MS m/z (rel. int.): 384 (2%, $[M–H]^+$), 369 (1), 341 (100), 313 (8), 299 (23), 285 (6), 271 (20), 257 (23), 241 (5), 225 (7), 217 (7), 203 (6), 187 (6), 175 (7), 159 (10), 145 (16), 135 (79), 128 (12), 121 (9), 111 (7), 105 (16), 97 (5), 83 (10), 73 (20), 69 (4), 59 (6).

EA: $C_{22}H_{36}Si_3$ (384.212): calcd. C 68.67, H 9.43; found C 68.54, H 9.40.

[[2-(Triethylsilyl)ethynyl][2-(triisopropylsilyl)ethynyl]silyl]benzene (5ac-b)

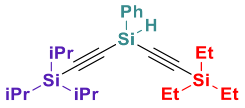

[[2-(Triethylsilyl)ethynyl][2-(triisopropylsilyl)ethynyl]silyl]benzene was obtained as oil in 99% yield. The title compound was previously unknown.

^{1}H NMR (400 MHz, $CDCl_3$) δ (ppm) = 0.64–0.72 (q, J = 7.9 Hz, 6H), 1.02–1.09 (t, J = 7.9 Hz, 9H), 1.14 (s, 21H), 4.88 (s, 1H), 7.39–7.51 (m, 3H), 7.75–7.84 (m, 2H).

^{13}C NMR (101 MHz, $CDCl_3$) δ (ppm) = 4.2, 7.4, 11.1, 18.6, 106.0, 106.5, 115.7, 116.3, 128.1, 130.2, 130.6, 134.7.

^{29}Si NMR (79 MHz, $CDCl_3$) δ (ppm) = –69.7, –6.8, –1.6.

EI-MS m/z (rel. int.): 426 (1%, $[M–H]^+$), 397 (1), 383 (100), 355 (10), 341 (16), 327 (3), 313 (9), 299 (6), 285 (5), 271 (5), 257 (6), 243 (6), 229 (6), 175 (5), 159 (6), 149 (19), 145 (6), 135 (16), 128 (5), 121 (15), 111 (7), 105 (11), 97 (3), 83 (7), 73 (3), 69 (3), 59 (6).

EA: $C_{25}H_{42}Si_3$ (426.259): calcd. C 70.34, H 9.92; found C 70.30, H 9.95.

[[2-(Tert-butyl(dimethyl)silyl)ethynyl][2-(triisopropylsilyl)ethynyl]silyl]benzene (5ac-g)

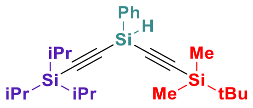

[[2-(Tert-butyl(dimethyl)silyl)ethynyl][2-(triisopropylsilyl)ethynyl]silyl]benzene was obtained as oil in 94% yield. The title compound was previously unknown.

^{1}H NMR (400 MHz, $CDCl_3$) δ (ppm) = 0.15–0.23 (d, *J* = 2.5 Hz, 6H), 1.00 (s, 9H), 1.14 (s, 21H), 4.87 (s, 1H), 7.37–7.53 (m, 3H), 7.73–7.84 (m, 2H).

^{13}C NMR (101 MHz, $CDCl_3$) δ (ppm) = –4.9, 11.1, 16.6, 18.6, 26.0, 105.4, 106.4, 115.7, 117.1, 128.1, 130.3, 130.6, 134.7.

^{29}Si NMR (79 MHz, $CDCl_3$) δ (ppm) = –67.8, –7.7, –1.5.

EI-MS m/z (rel. int.): 426 (2%, $[M–H]^+$), 383 (100), 369 (6), 341 (49), 327 (8), 313 (3), 299 (31), 283 (10), 271 (7), 257 (11), 241 (14), 225 (9), 217 (8), 203 (9), 191 (7), 175 (10), 159 (13), 145 (18), 135 (60), 129 (7), 121 (11), 111 (8), 105 (14), 97 (6), 83 (16), 73 (24), 57 (13).

EA: $C_{25}H_{42}Si_3$ (426.259): calcd. C 70.34, H 9.92; found C 70.27, H 9.91.

((Decyl(phenyl)silyl)ethynyl)triethylsilane (7ab-a)

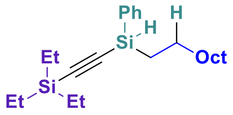

((Decyl(phenyl)silyl)ethynyl)triethylsilane was obtained as oil in 89% yield. The title compound was previously unknown.

^{1}H NMR (400 MHz, C_6D_6) δ (ppm) = 0.46–0.75 (m, 7H), 0.86–0.99 (m, 6H), 1.01–1.16 (m, 9H), 1.18–148 (m, 12H), 1.51–1.75 (m, 2H), 4.87–4.94 (m, 1H), 7.16–7.35 (m, 3H), 7.69–8.08 (m, 2H).

^{13}C NMR (101 MHz, C_6D_6) δ (ppm) = 4.3, 7.4, 13.2, 14.0, 22.8, 24.2, 29.4, 29.4, 29.6, 29.7, 32.0, 32.9, 109.3, 115.9, 128.0, 129.8, 132.8, 134.7.

^{29}Si NMR (79 MHz, C_6D_6) δ (ppm) = –36.8, –7.6.

EI-MS m/z (rel. int.): 386 (2%, $[M–H]^+$), 357 (34), 329 (16), 308 (3), 279 (30), 270 (6), 257 (2), 251 (4), 245 (20), 231 (4), 217 (100), 209 (5), 201 (4), 189 (82), 175 (8), 168 (5), 161 (70), 145 (8), 135 (21), 121 (8), 115 (11), 105 (47), 94 (5), 83 (12), 69 (3), 57 (8), 53 (3).

EA: $C_{20}H_{42}Si_2$ (386.283): calcd. C 74.53, H 10.95; found C 74.63, H 11.02.

((Decyl(phenyl)silyl)ethynyl)triisopropylsilane (7ac-a)

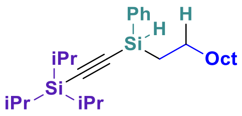

((Decyl(phenyl)silyl)ethynyl)triisopropylsilane was obtained as oil in 95% yield. The title compound was previously unknown.

^{1}H NMR (400 MHz, C_6D_6) δ (ppm) = 0.85–1.00 (m, 5H), 1.04–1.21 (m, 22H), 1.23–1.39 (m, 13H), 1.51–1.70 (m, 2H), 4.86–4.94 (m, 1H), 7.16–7.31 (m, 3H), 7.70–8.04 (m, 2H).

^{13}C NMR (101 MHz, C_6D_6) δ (ppm) = 11.1, 13.3, 14.0, 18.5, 22.8, 24.2, 29.4, 29.4, 29.6, 29.7, 32.0, 32.9, 110.0, 115.0, 128.0, 129.8, 132.9, 134.7.

^{29}Si NMR (79 MHz, C_6D_6) δ (ppm) = –36.9, –2.1.

EI-MS m/z (rel. int.): 428 (1%, $[M–H]^{+}$), 385 (100), 357 (3), 343 (11), 315 (3), 301 (3), 287 (4), 269 (2), 245 (29), 229 (3), 217 (25), 203 (24), 189 (24), 175 (28), 159 (12), 145 (12), 135 (16), 121 (24), 111 (3), 105 (22), 97 (4), 83 (4), 73 (4), 59 (4).

EA: $C_{27}H_{48}Si_2$ (428.329): calcd. C 75.62, H 11.28; found C 75.63, H 11.22.

Tert-butyl((decyl(phenyl)silyl)ethynyl)dimethylsilane (7ag-a)

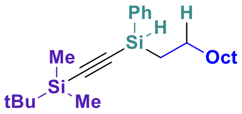

Tert-butyl((decyl(phenyl)silyl)ethynyl)dimethylsilane was obtained as oil in 85% yield. The title compound was previously unknown.

^{1}H NMR (400 MHz, C_6D_6) δ (ppm) = –0.01–0.21 (m, 6H), 0.77–1.12 (m, 16H), 1.16–1.40 (m, 12H), 1.45–1.66 (m, 2H), 4.85–4.94 (m, 1H), 7.16–7.32 (m, 3H), 7.68–8.02 (m, 2H).

^{13}C NMR (101 MHz, C_6D_6) δ (ppm) = –5.0, 13.1, 14.0, 16.3, 22.8, 24.2, 25.9, 29.3, 29.4, 29.6, 29.7, 32.0, 32.9, 108.7, 116.8, 128.0, 129.8, 132.8, 134.7.

^{29}Si NMR (79 MHz, C_6D_6) δ (ppm) = –36.8, –8.4.

EI-MS m/z (rel. int.): 386 (1%, $[M–H]^{+}$), 329 (66), 308 (1), 269 (4), 251 (7), 245 (7), 231 (5), 217 (9), 203 (9), 189 (100), 173 (12), 159 (5), 145 (13), 135 (52), 121 (11), 111 (3), 105 (19), 97 (3), 83 (5), 73 (5), 57 (6).

EA: $C_{24}H_{42}Si_2$ (386.283): calcd. C 74.53, H 10.95; found C 74.67, H 11.05.

Triethyl(((3-(glycidoxy)propyl)(phenyl)silyl)ethynyl)silane (7ab-b)

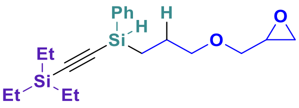

Triethyl(((3-(glycidoxy)propyl)(phenyl)silyl)ethynyl)silane was obtained as oil in 89% yield. The title compound was previously unknown.

^{1}H NMR (400 MHz, C_6D_6) δ (ppm) = 0.56–0.74 (m, 6H), 0.88–1.36 (m, 14H), 1.77–1.96 (m, 1H), 2.14–2.27 (m, 1H), 2.29–2.47 (m, 1H), 2.77–2.96 (m, 1H), 3.08–3.21 (m, 1H), 3.23–3.48 (m, 2H), 4.88–4.99 (m, 1H), 7.24–7.41 (m, 3H), 7.74–8.17 (m, 2H).

^{13}C NMR (101 MHz, C_6D_6) δ (ppm) = 4.3, 7.4, 9.6, 24.5, 43.4, 50.4, 71.5, 73.0, 109.1, 116.2, 128.1, 129.9, 132.6, 134.8.

^{29}Si NMR (79 MHz, C_6D_6) δ (ppm) = –36.5, –7.4.

EI-MS m/z (rel. int.): 360 (2%, $[M–H]^+$), 343 (1), 303 (1), 301 (1), 283 (11), 273 (19), 267 (5), 259 (17), 245 (24), 231 (49), 225 (17), 217 (15), 211 (13), 203 (100), 195 (20), 187 (45), 175 (95), 167 (18), 161 (75), 151 (16), 147 (53), 143 (27), 131 (60), 123 (11), 115 (50), 111 (26), 105 (95), 94 (20), 87 (42), 83 (24), 71 (7), 65 (10), 59 (17), 53 (10).

EA: $C_{20}H_{32}O_2Si_2$ (360.194): calcd. C 66.61, H 8.94; found C 66.50, H 9.00.

Triisopropyl(((3-(glycidoxy)propyl)(phenyl)silyl)ethynyl)silane (7ac-b)

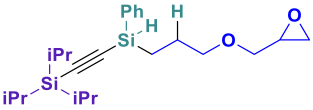

Triisopropyl(((3-(glycidoxy)propyl)(phenyl)silyl)ethynyl)silane was obtained as oil in 90% yield. The title compound was previously unknown.

^{1}H NMR (400 MHz, C_6D_6) δ (ppm) = 0.77–1.40 (m, 25H), 1.71–1.87 (m, 1H), 2.08–2.20 (m, 1H), 2.22–2.32 (m, 1H), 2.76–2.86 (m, 1H), 2.99–3.13 (m, 1H), 3.18–3.39 (m, 2H), 4.82–4.88 (m, 1H), 7.16–7.32 (m, 3H), 7.64–8.01 (m, 2H).

^{13}C NMR (101 MHz, C_6D_6) δ (ppm) = 9.6, 11.1, 18.5, 24.4, 43.3, 50.3, 71.4, 72.9, 109.7, 115.2, 128.0, 129.8, 132.7, 134.7.

^{29}Si NMR (79 MHz, C_6D_6) δ (ppm) = –36.7, –2.0.

EI-MS m/z (rel. int.): 359 (1%, $[M–iPr]^+$), 345 (2), 325 (10), 317 (6), 309 (10), 301 (19), 287 (26), 283 (5), 173 (18), 267 (2), 259 (35), 247 (25), 231 (63), 217 (53), 203 (57), 197 (16), 189 (100), 181 (38), 175 (34), 165 (33), 159 (28), 145 (49), 139 (38), 131 (37), 121 (40), 115 (30), 111 (47), 105 (73), 101 (19), 94 (33), 83 (60), 69 (34), 59 (21), 53 (11).

EA: $C_{23}H_{38}O_2Si_2$ (360.194): calcd. C 68.60, H 9.51; found C 68.41, H 9.50.

Tert-butyl(dimethyl)(((3-(glycidoxy)propyl)(phenyl)silyl)ethynyl)silane (7ag-b)

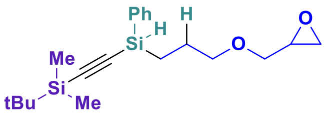

Tert-butyl(dimethyl)(((3-(glycidoxy)propyl)(phenyl)silyl)ethynyl)silane was obtained as oil in 75% yield. The title compound was previously unknown.

^{1}H NMR (400 MHz, C_6D_6) δ (ppm) = –0.05–0.21 (m, 6H), 0.78–1.19 (m, 13H), 1.72–1.83 (m, 1H), 2.10–2.19 (m, 1H), 2.21–2.32 (m, 1H), 2.68–2.91 (m, 1H), 2.95–3.14 (m, 1H), 3.16–3.42 (m, 2H), 4.83–4.88 (m, 1H), 7.17–7.36 (m, 3H), 7.60–8.15 (m, 2H).

^{13}C NMR (101 MHz, C_6D_6) δ (ppm) = –5.0, 9.5, 16.3, 24.4, 25.9, 43.3, 50.3, 71.5, 72.9, 108.5, 116.9, 128.0, 129.8, 132.5, 134.7.

^{29}Si NMR (79 MHz, C_6D_6) δ (ppm) = –36.6, –8.3.

EI-MS m/z (rel. int.): 360 (2%, $[M–H]^{+}$), 343 (0.5), 259 (4), 245 (10), 231 (18), 225 (1), 219 (5), 203 (100), 195 (30), 189 (8), 183 (10), 179 (14), 173 (12), 159 (13), 145 (19), 135 (36), 131 (12), 119 (23), 105 (27), 91 (9), 83 (14), 73 (14), 67 (3), 57 (10).

EA: $C_{20}H_{32}O_2Si_2$ (360.194): calcd. C 66.61, H 8.94; found C 66.70, H 9.06.

SPECTRA FOR ALL PRODUCTS

Trimethyl((phenylsilyl)ethynyl)silane (3aa)

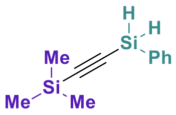

^{1}H NMR

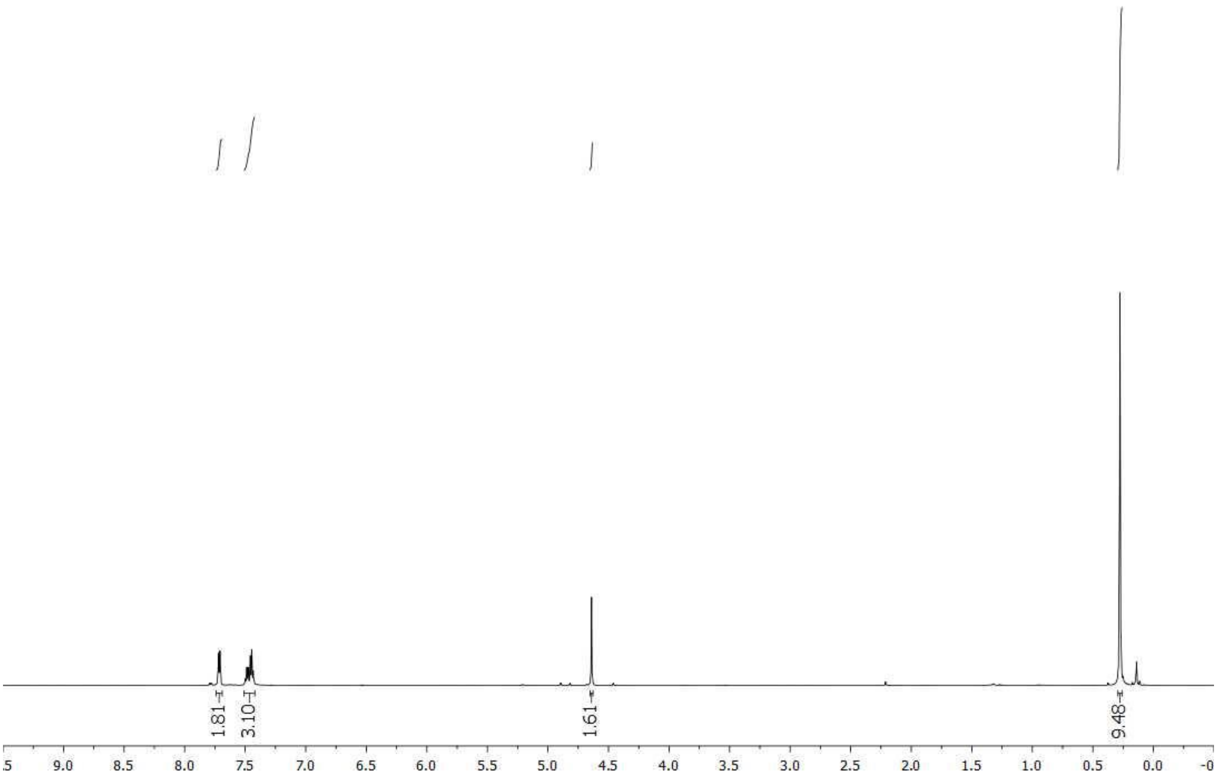

^{13}C NMR

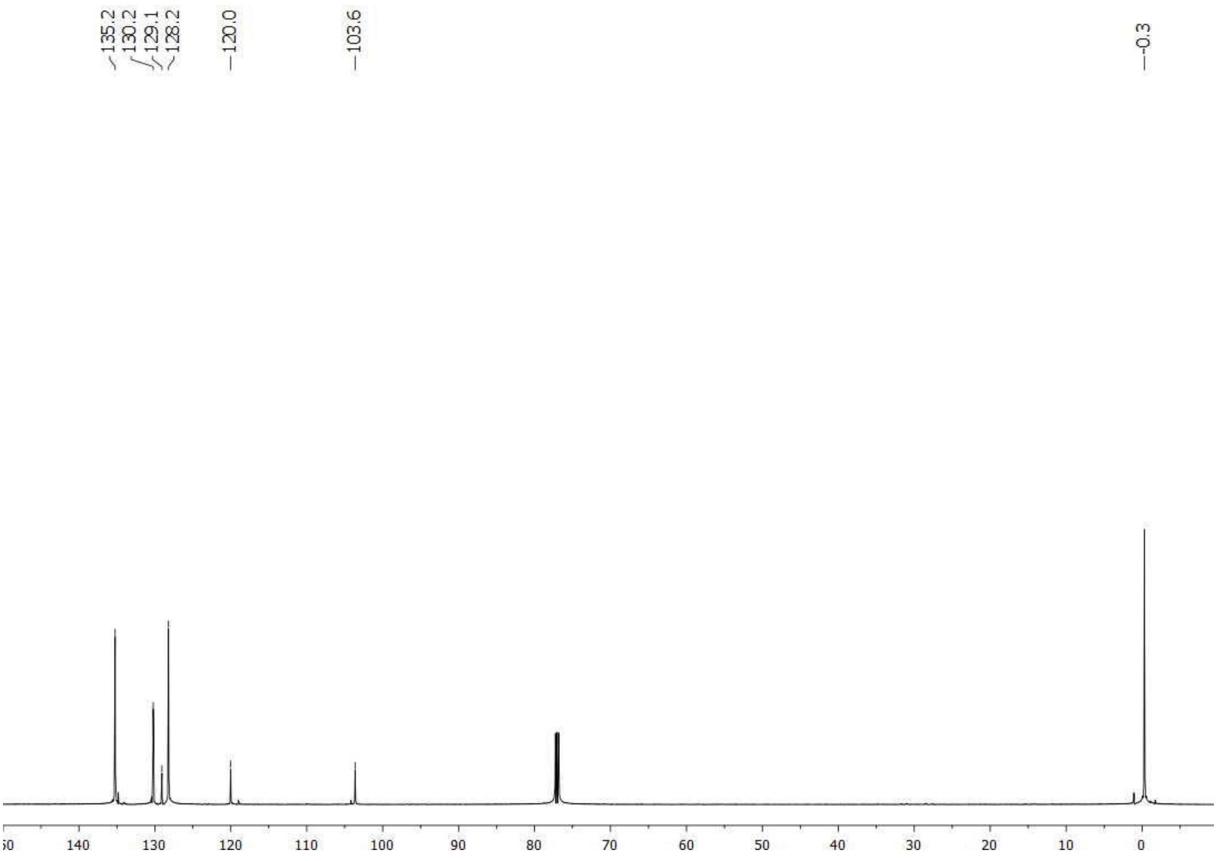

^{29}Si NMR

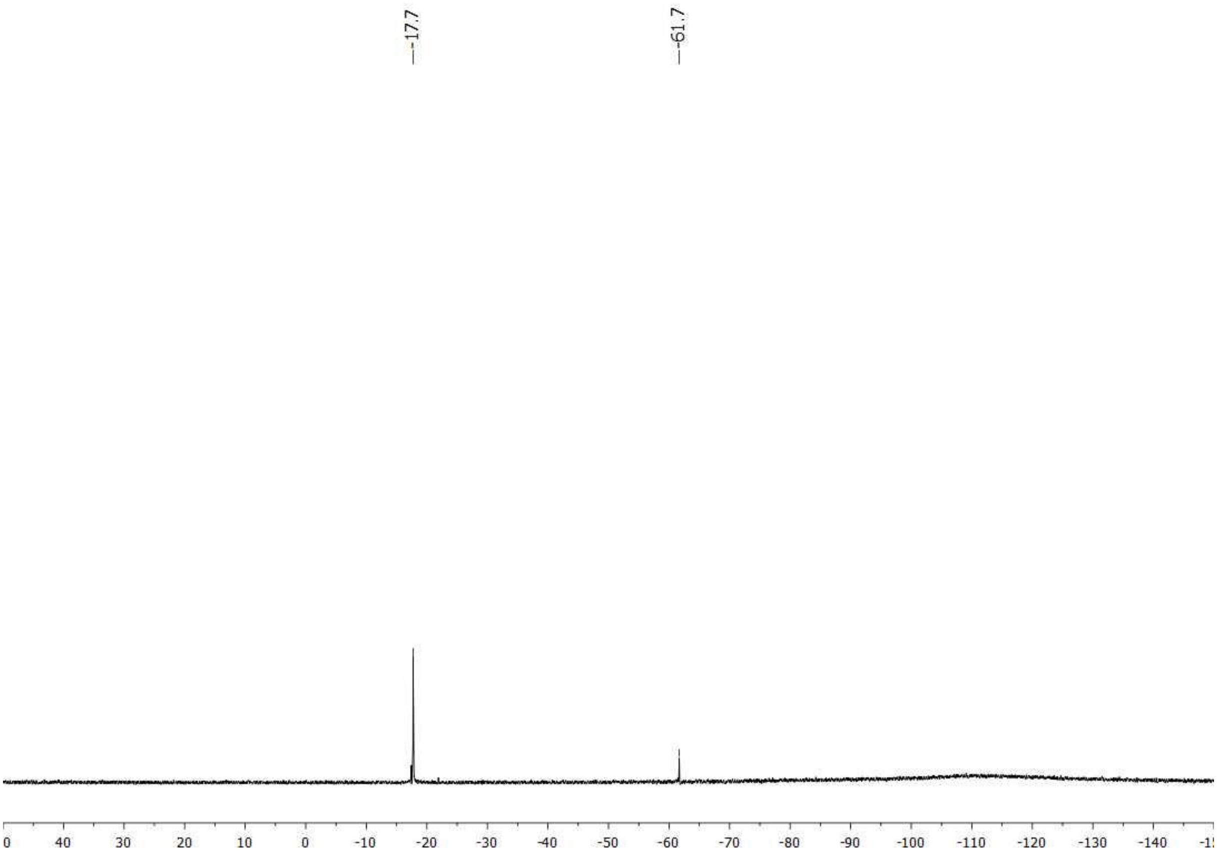

Triethyl((phenylsilyl)ethynyl)silane (3ab)

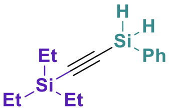

^{1}H NMR

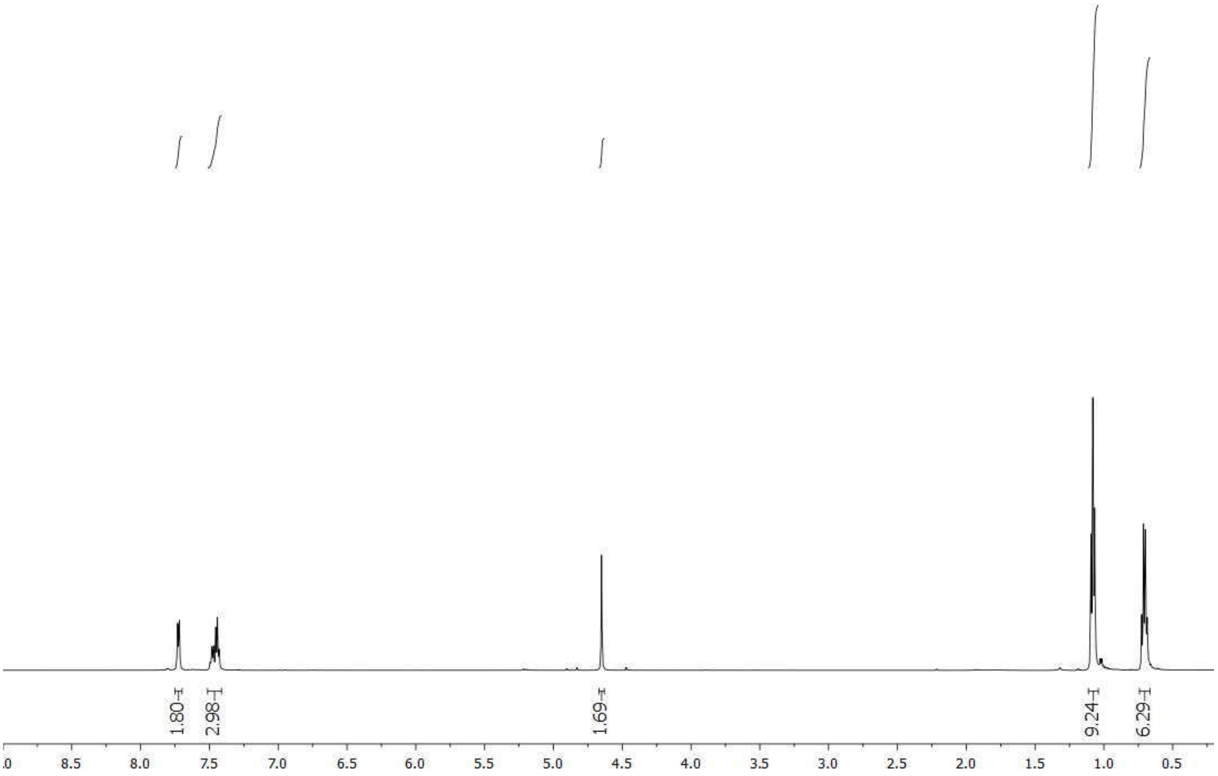

^{13}C NMR

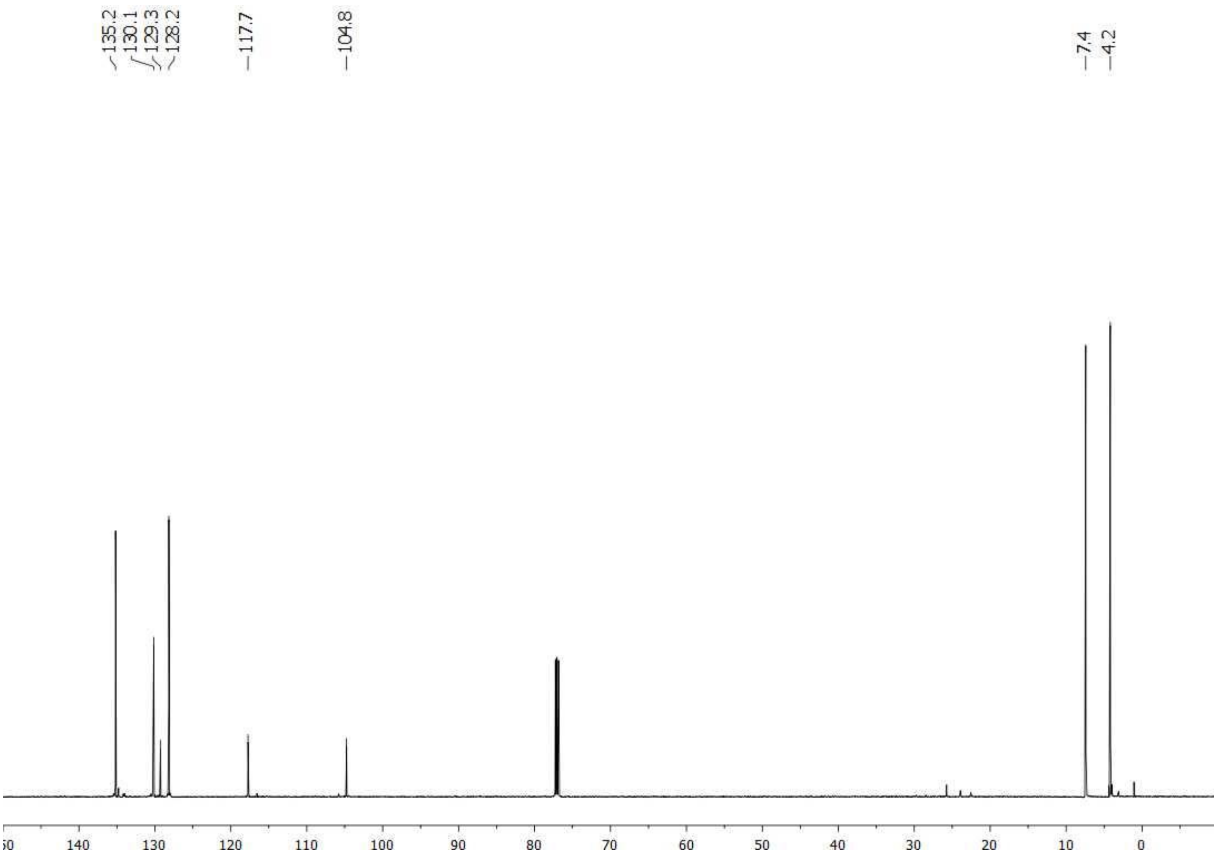

^{29}Si NMR

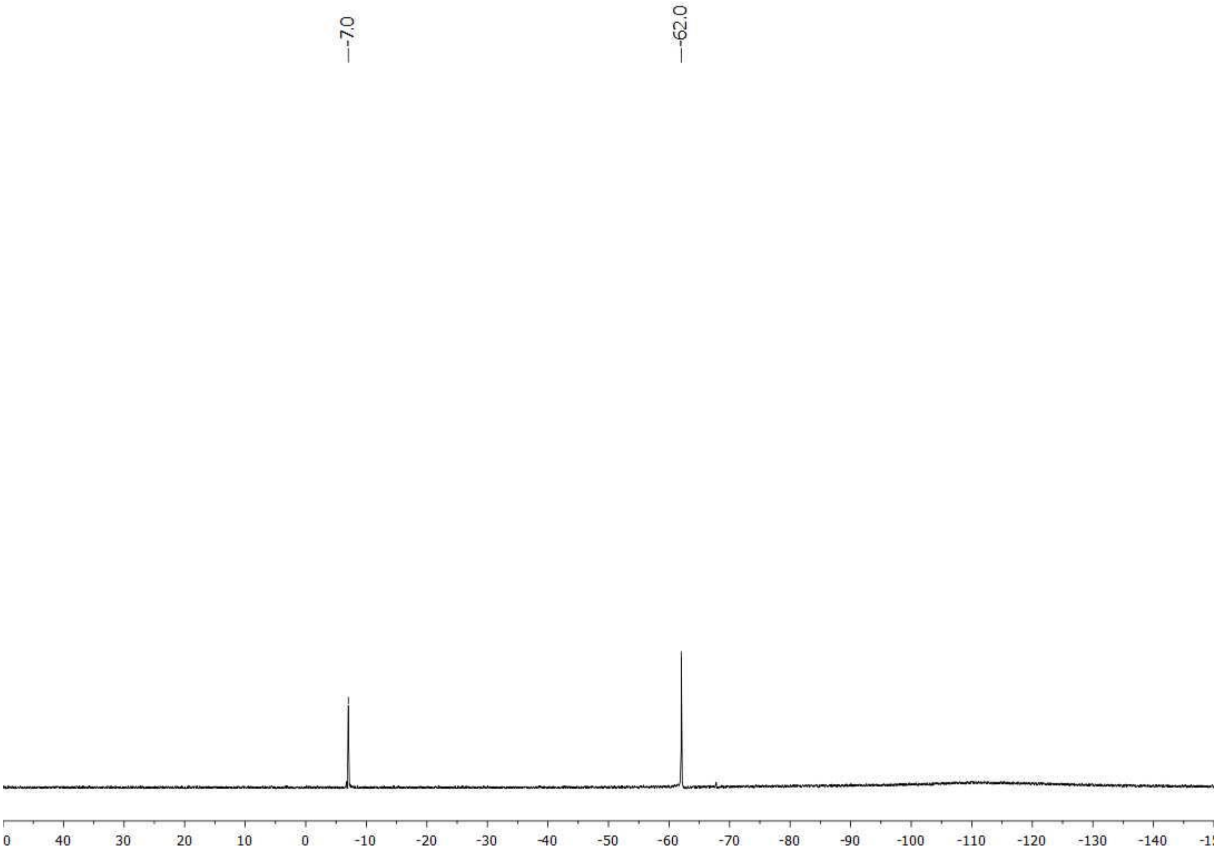

Triisopropyl((phenylsilyl)ethynyl)silane (3ac)

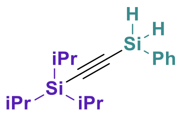

^{1}H NMR

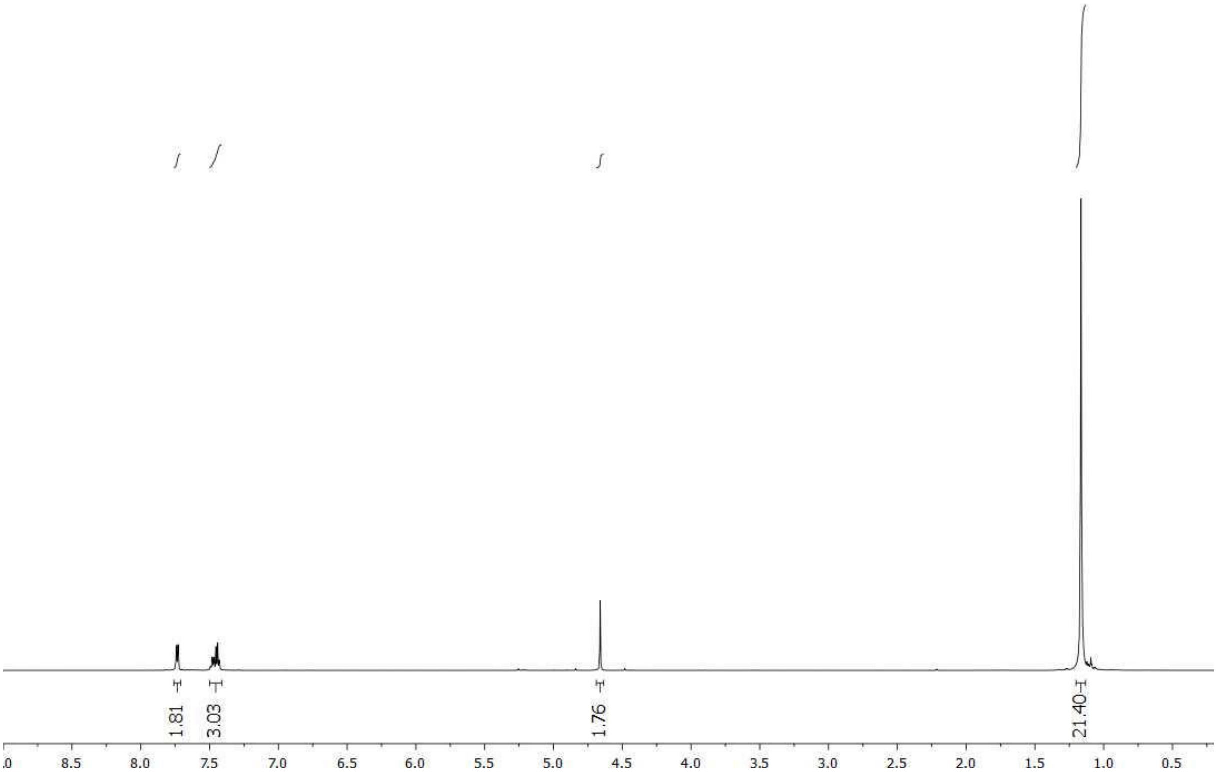

^{13}C NMR

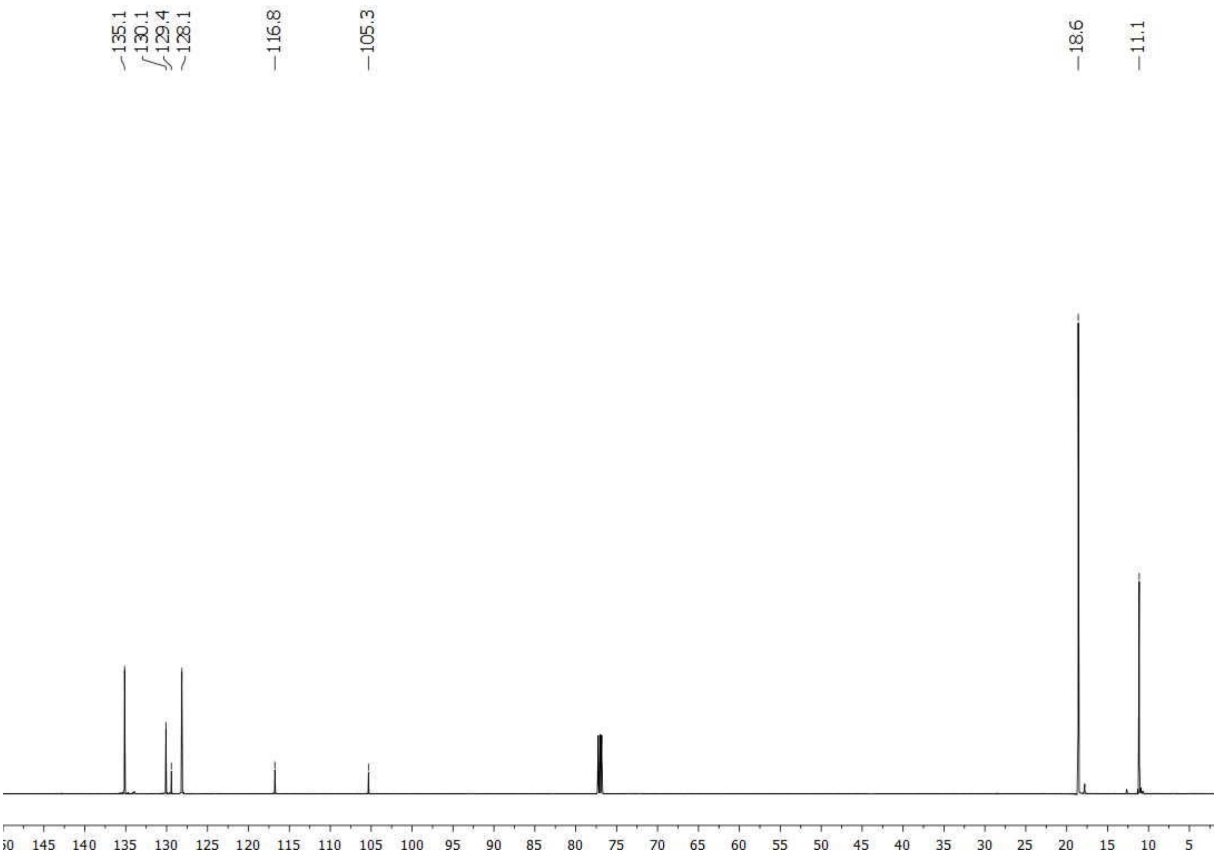

^{29}Si NMR

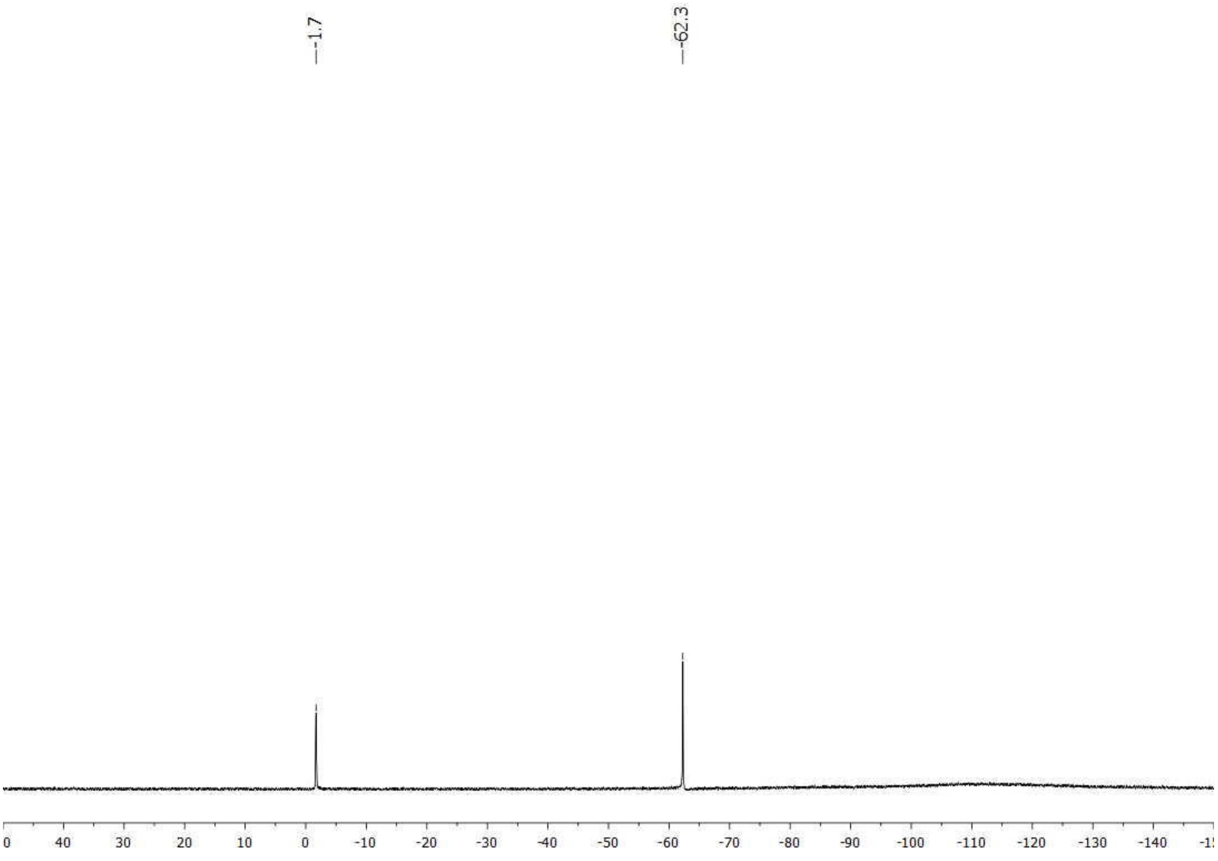

Diisopropyl((phenylsilyl)ethynyl)silane (3ad)

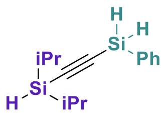

^{1}H NMR

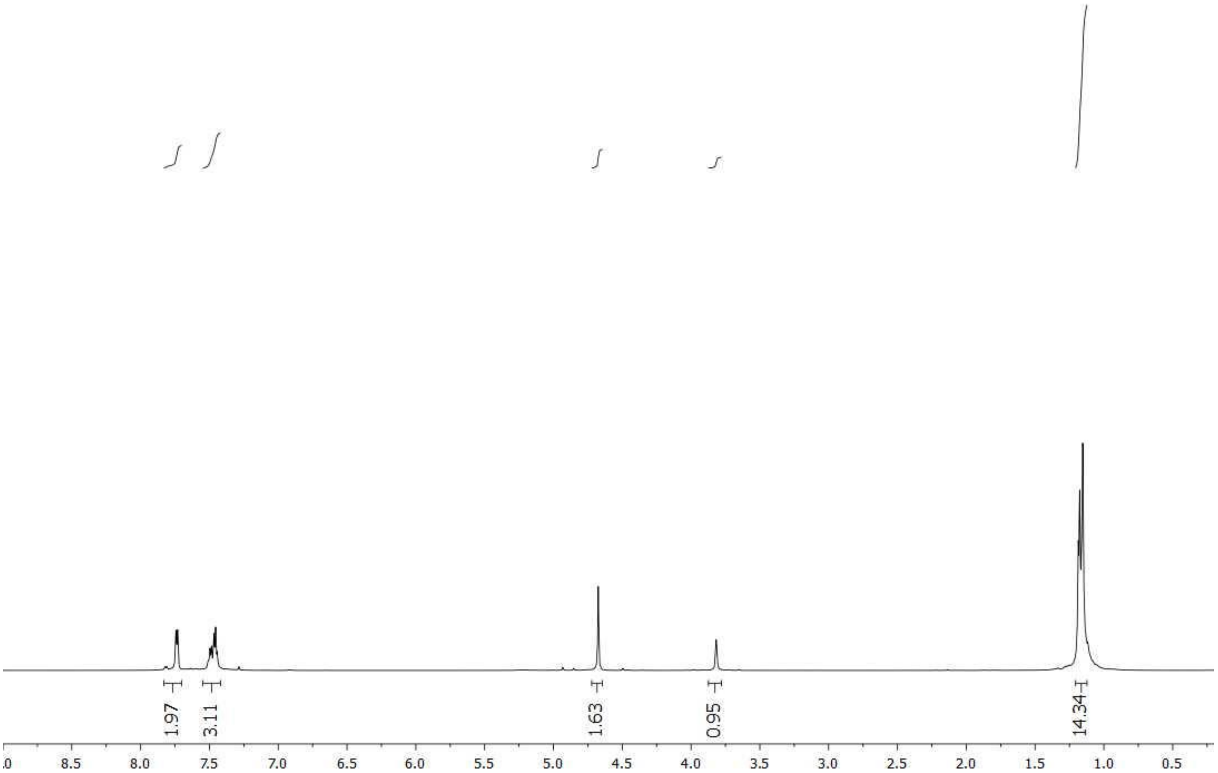

^{13}C NMR

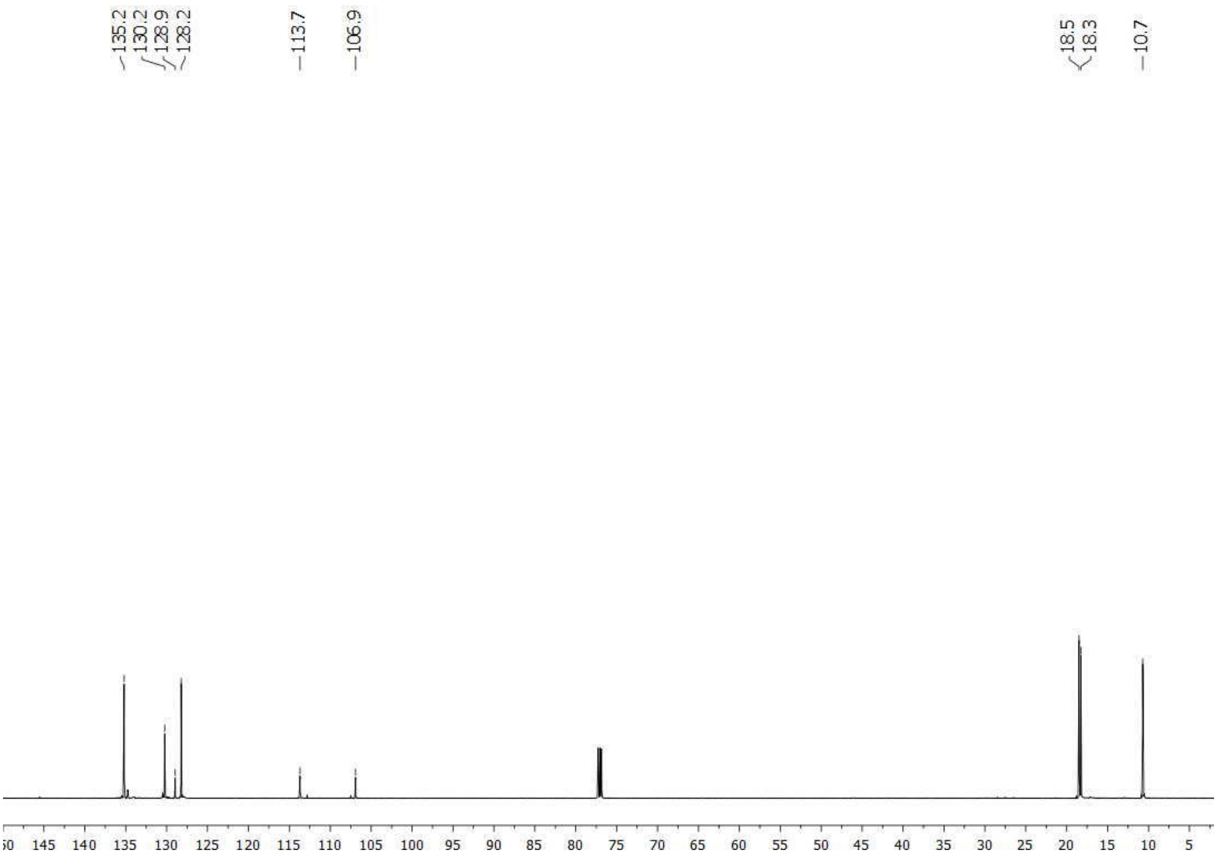

^{29}Si NMR

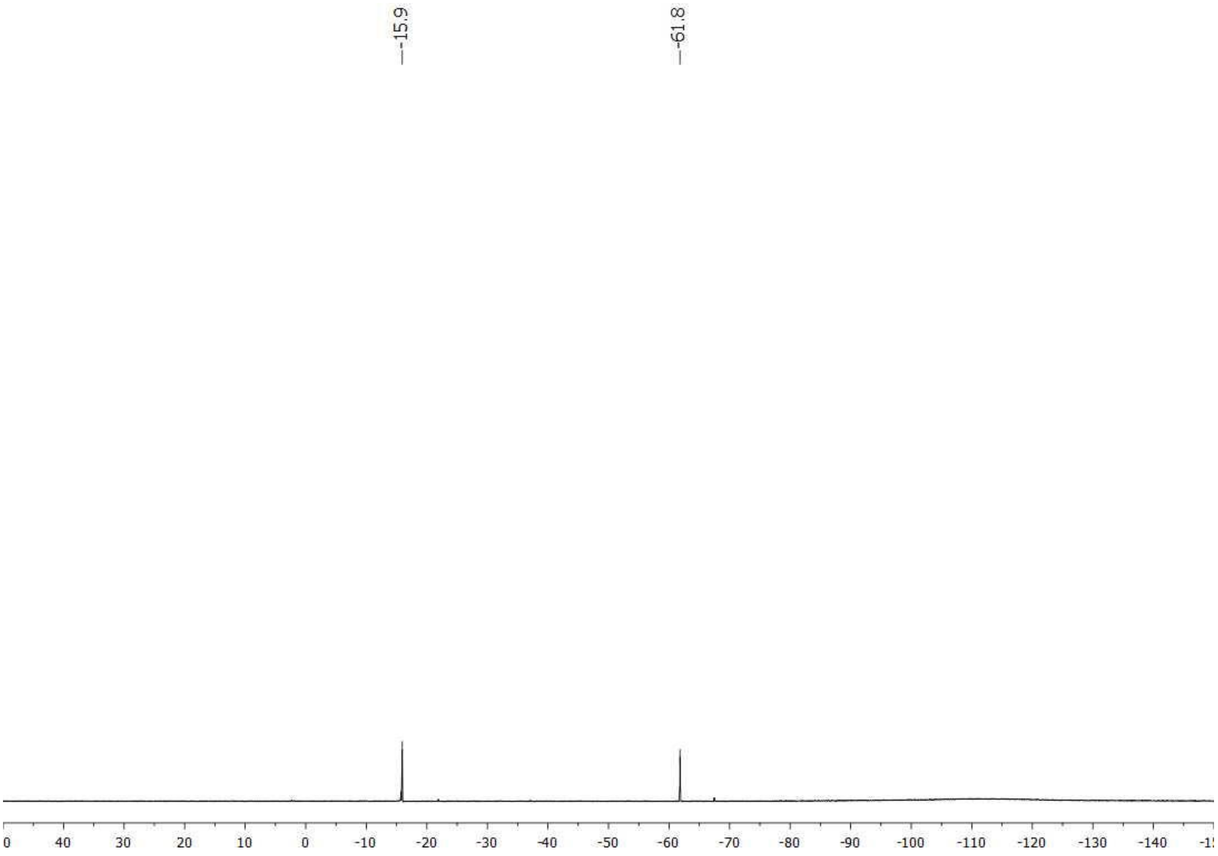

((Phenylsilyl)ethynyl)tripropylsilane (3ae)

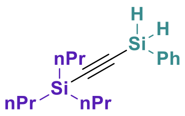

^{1}H NMR

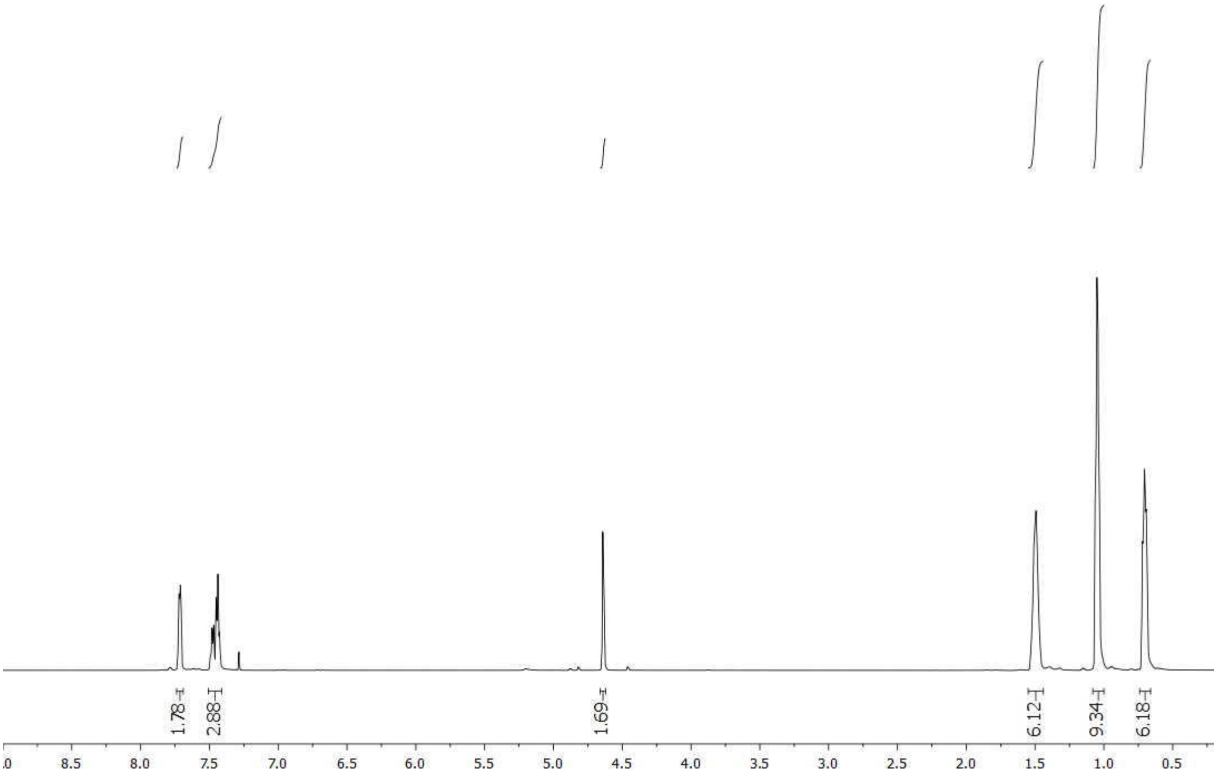

^{13}C NMR

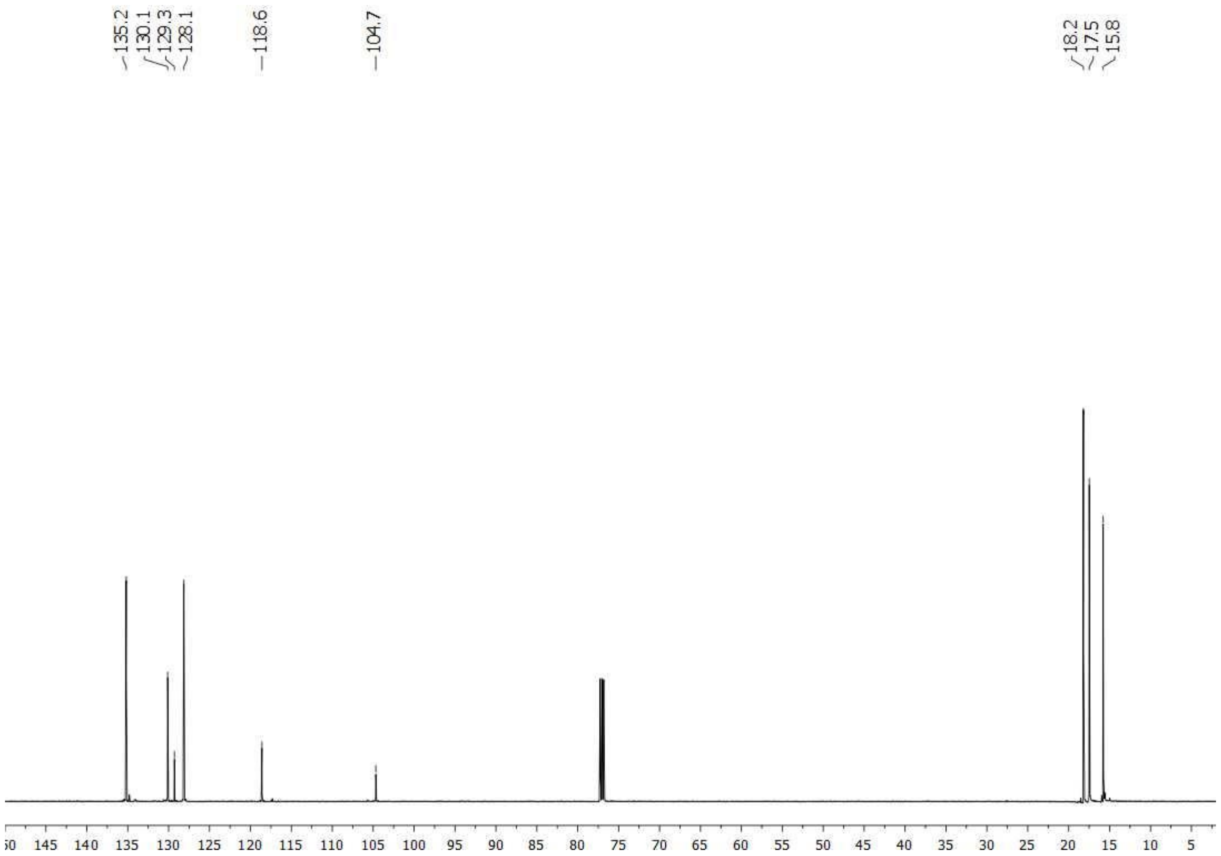

^{29}Si NMR

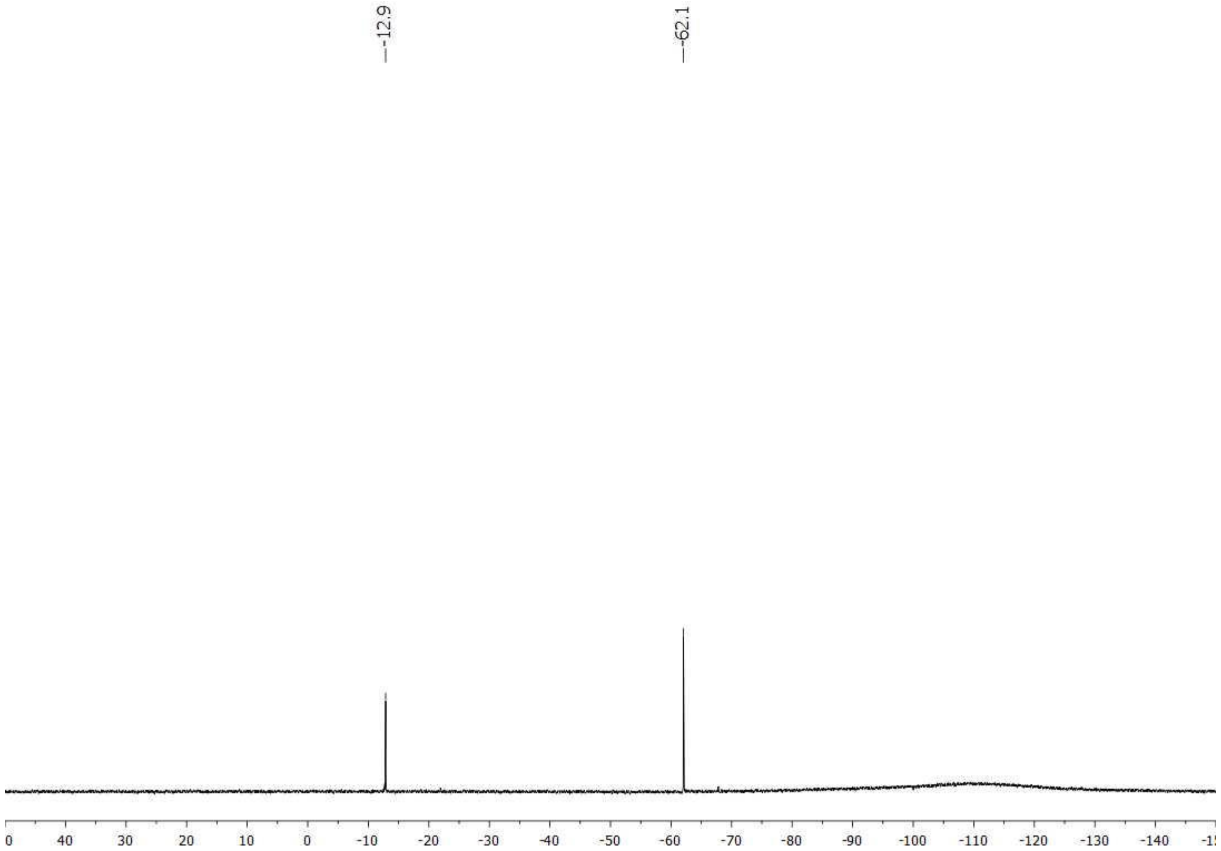

Triisobutyl((phenylsilyl)ethynyl)silane (3af)

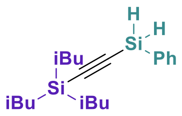

^{1}H NMR

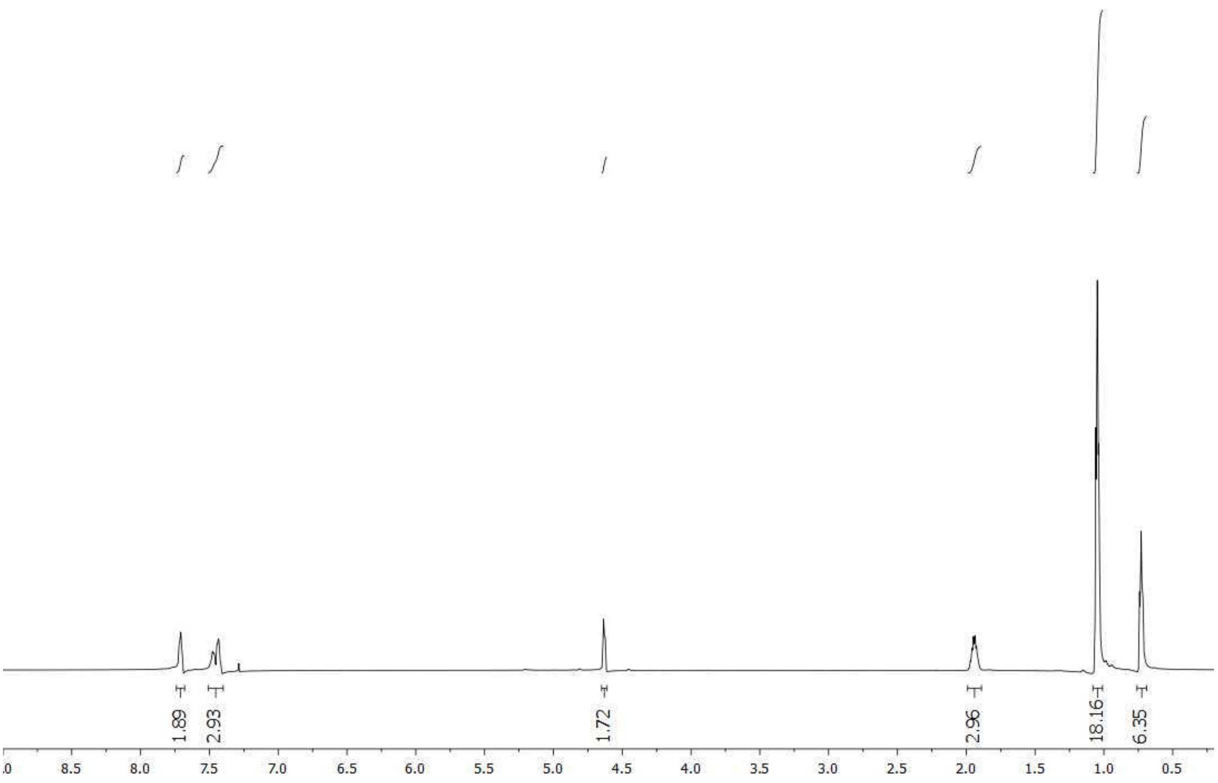

^{13}C NMR

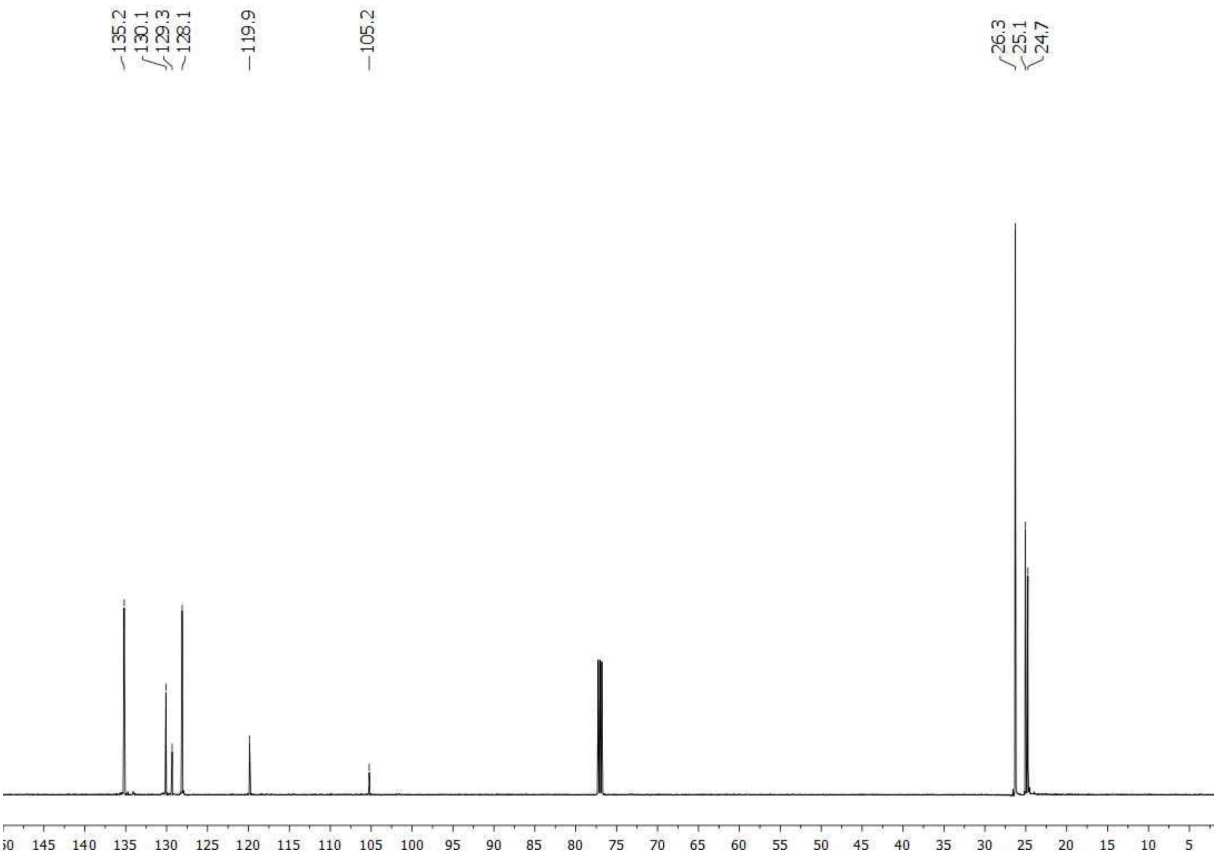

^{29}Si NMR

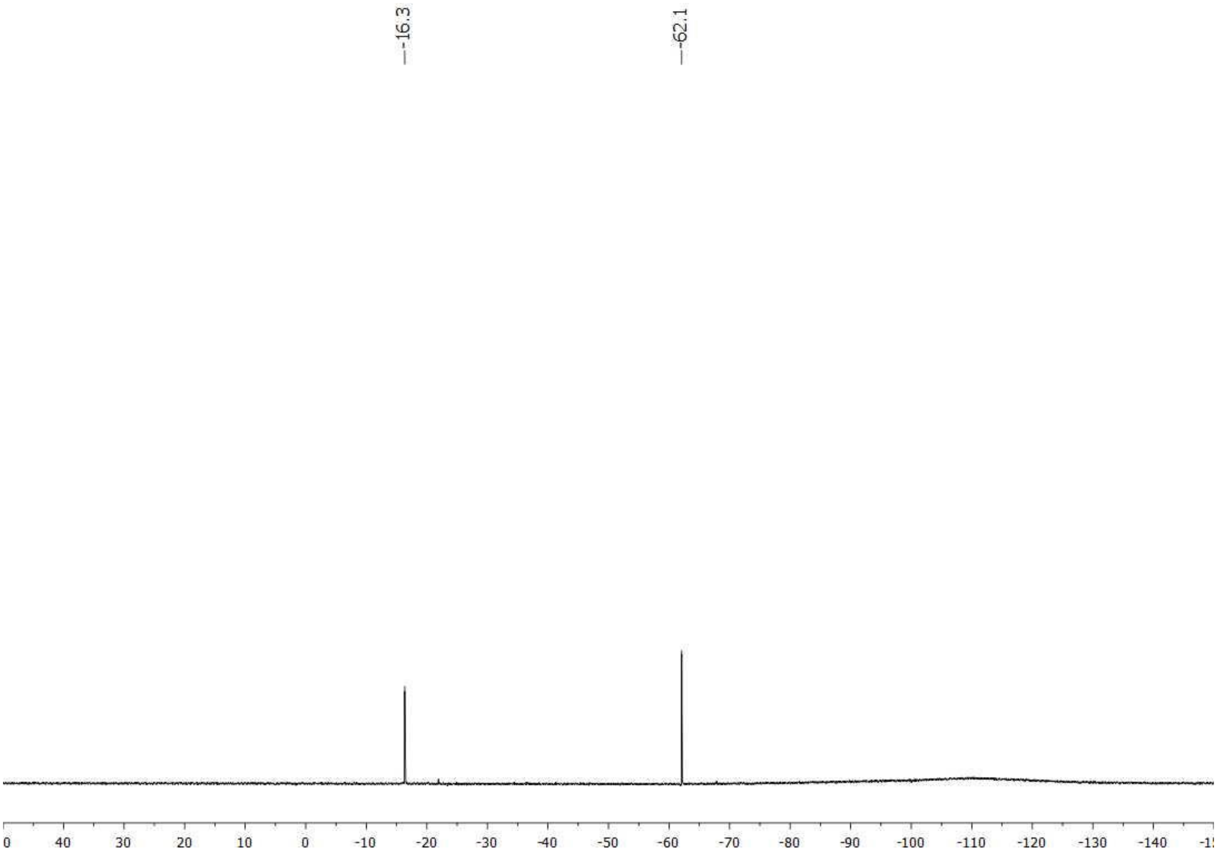

Tert-butyldimethyl((phenylsilyl)ethynyl)silane (3ag)

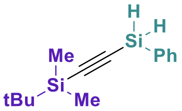

^{1}H NMR

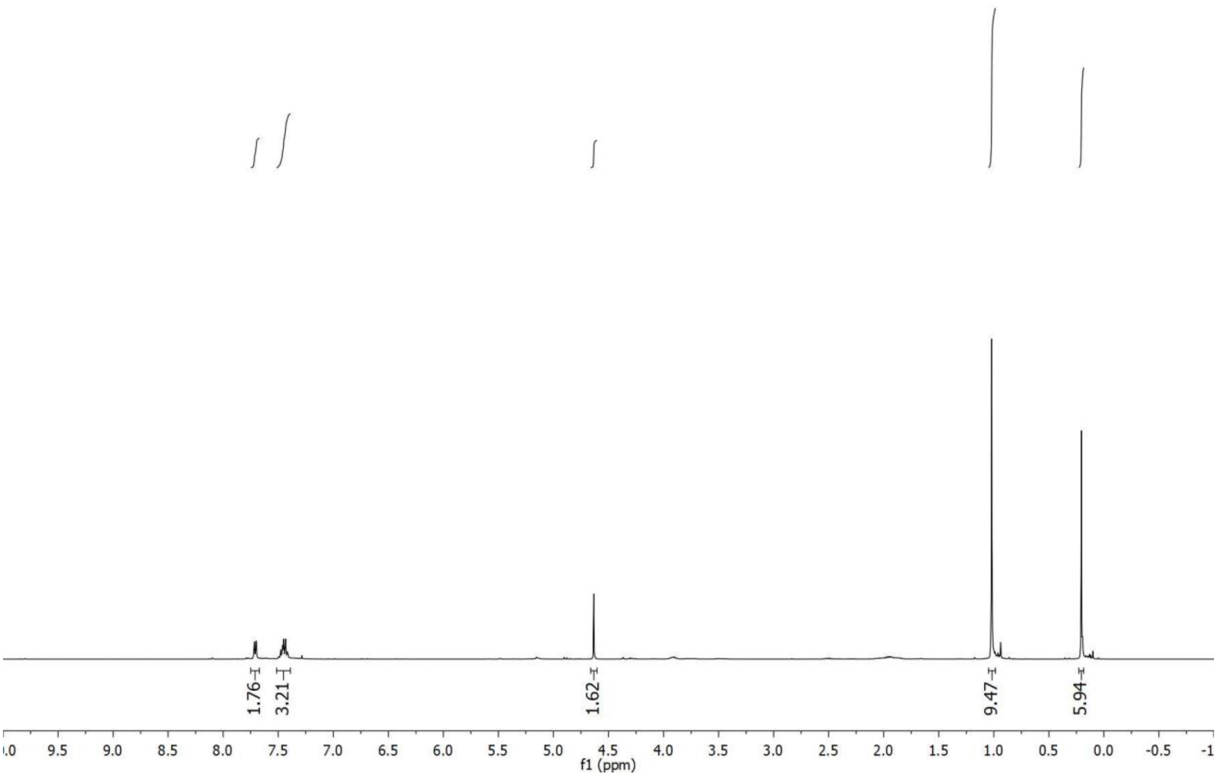

^{13}C NMR

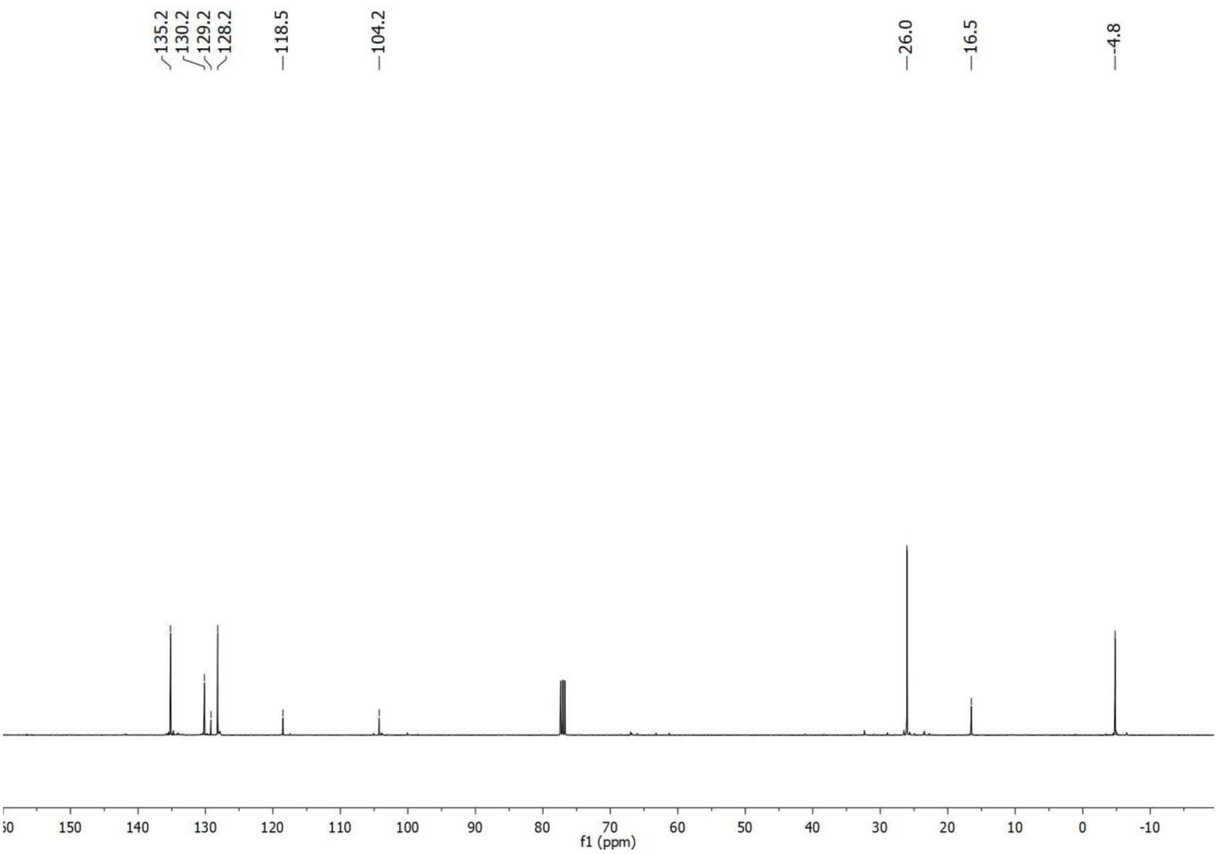

^{29}Si NMR

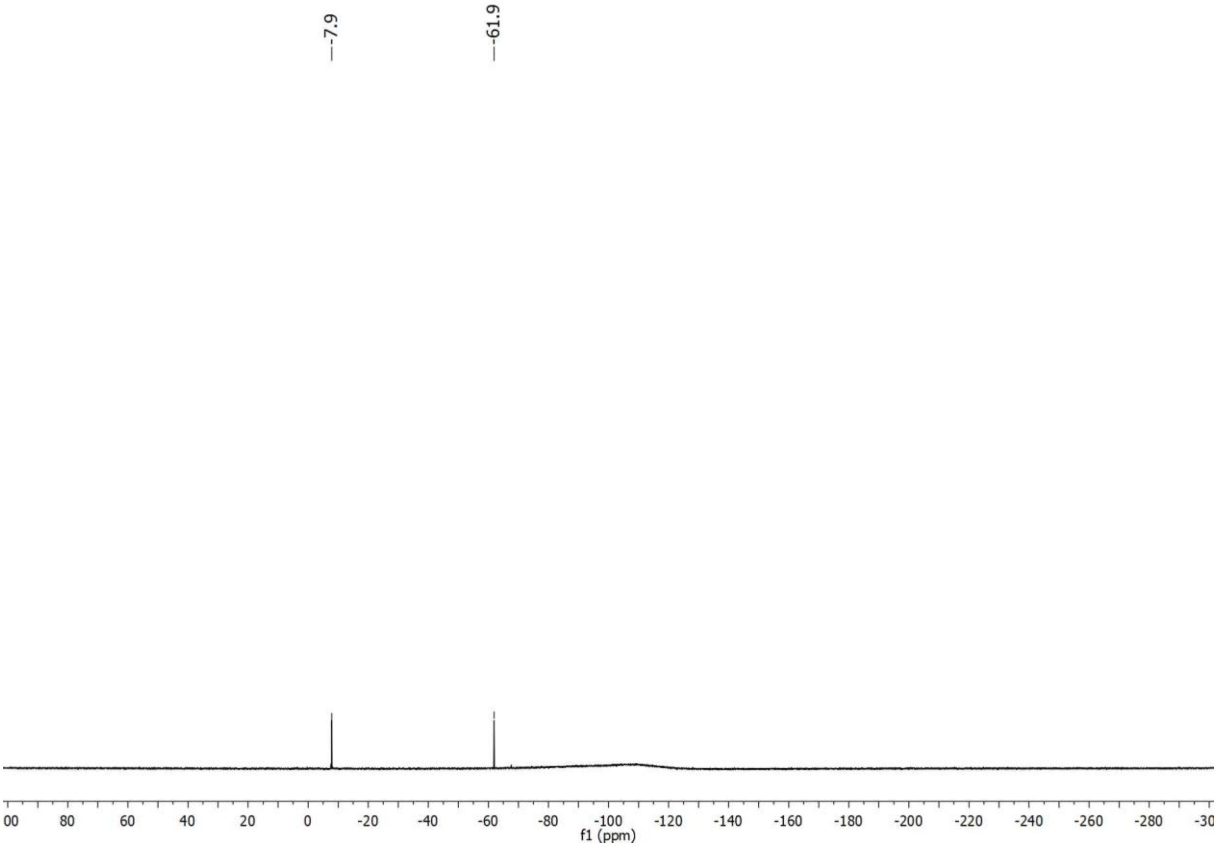

Dimethyl(octyl)((phenylsilyl)ethynyl)silane (3ah)

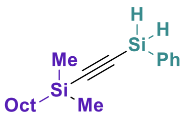

^{1}H NMR

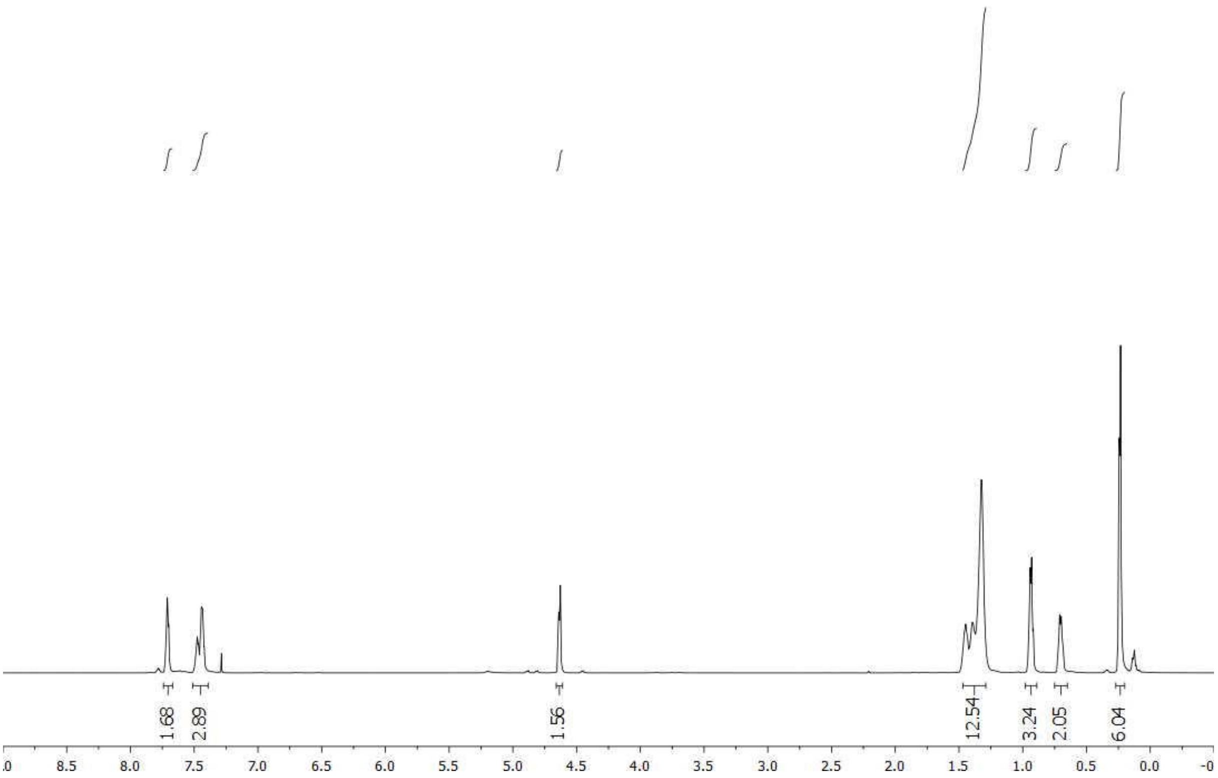

^{13}C NMR

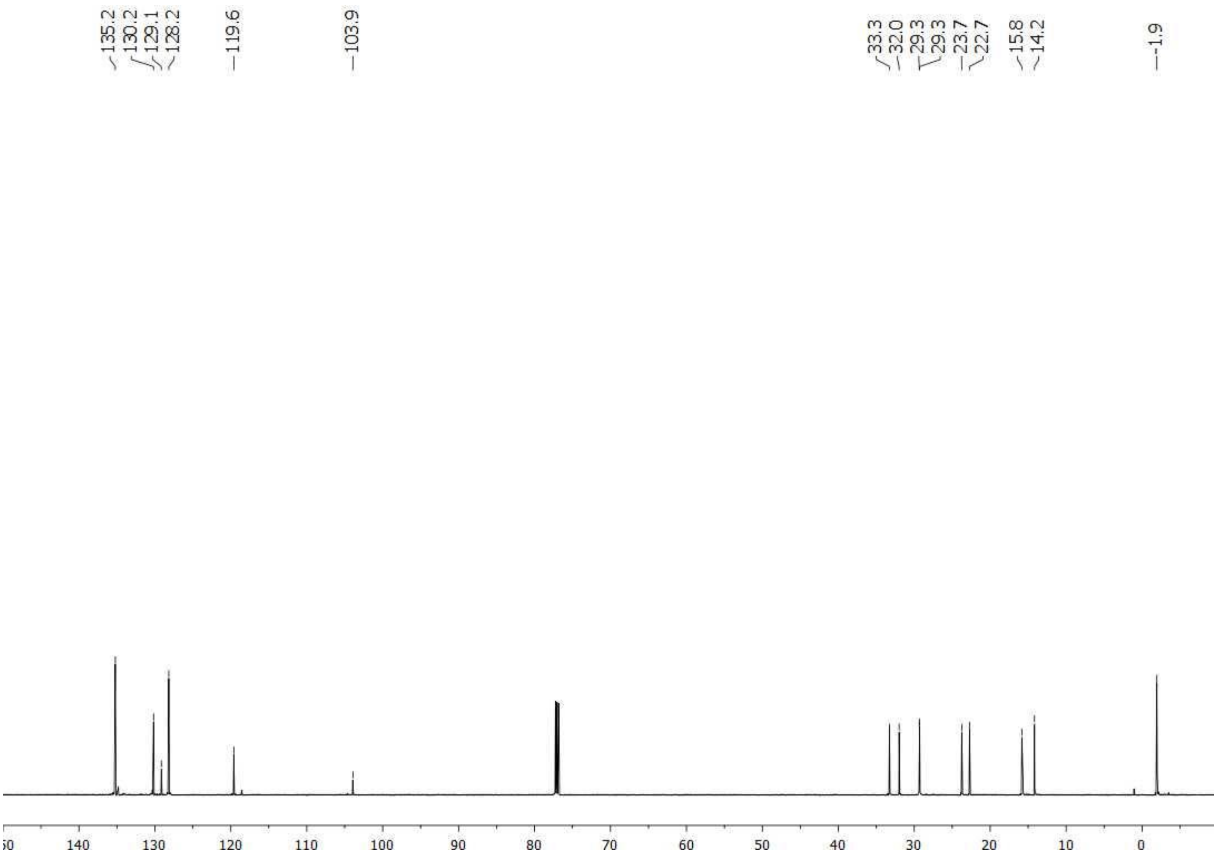

^{29}Si NMR

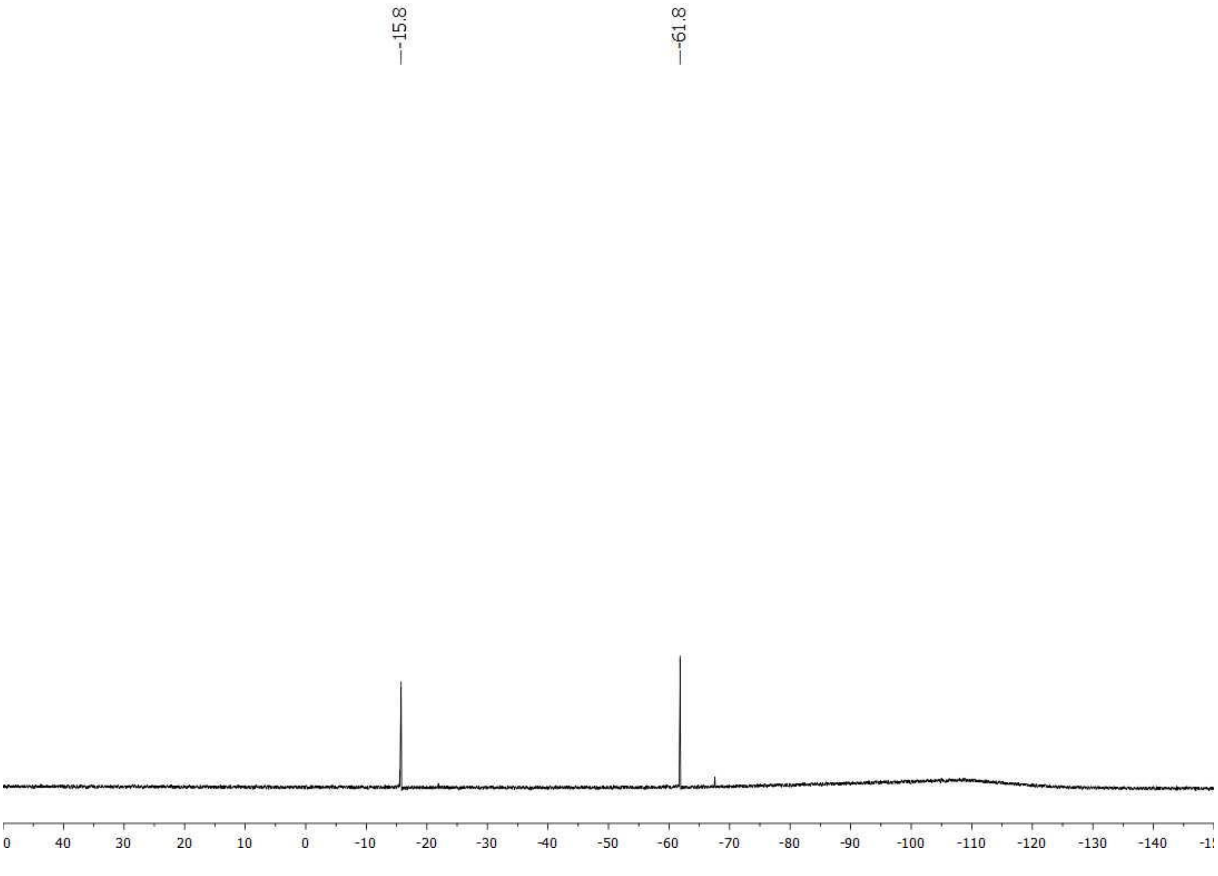

Dimethyl(phenyl)((phenylsilyl)ethynyl)silane (3ai)

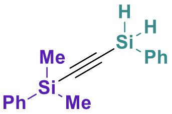

^{1}H NMR

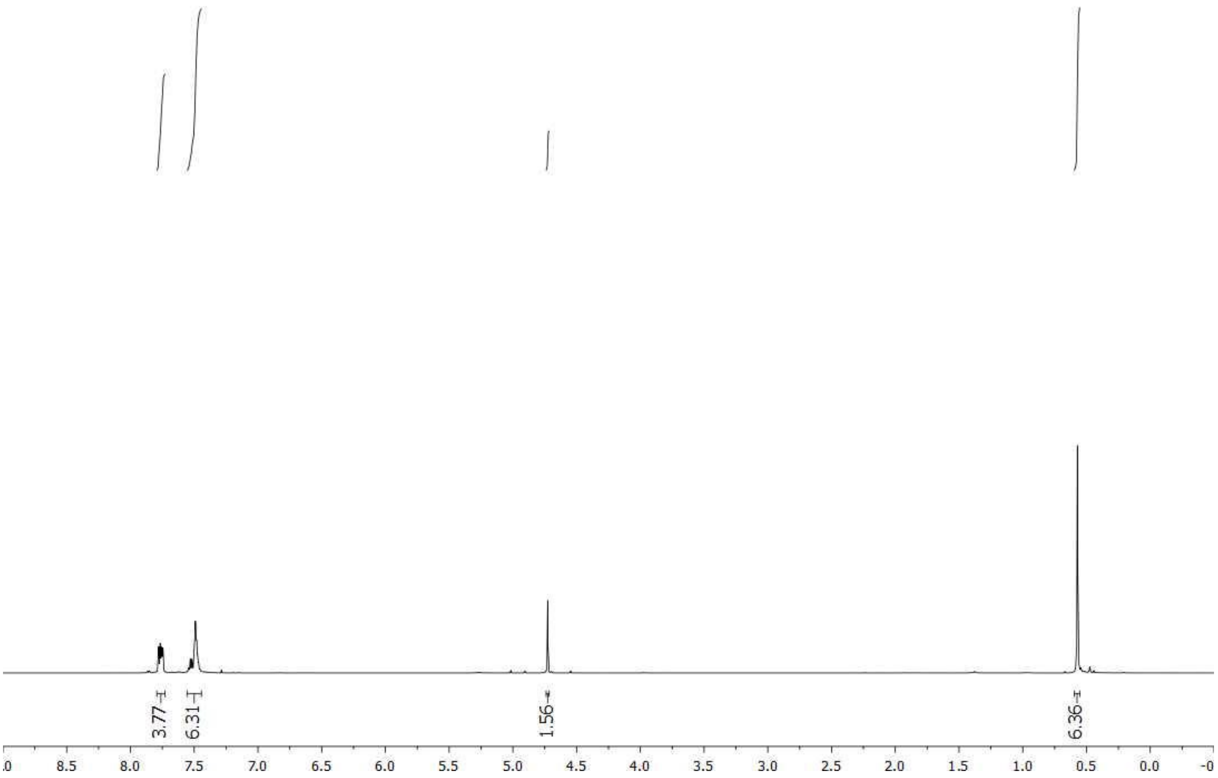

^{13}C NMR

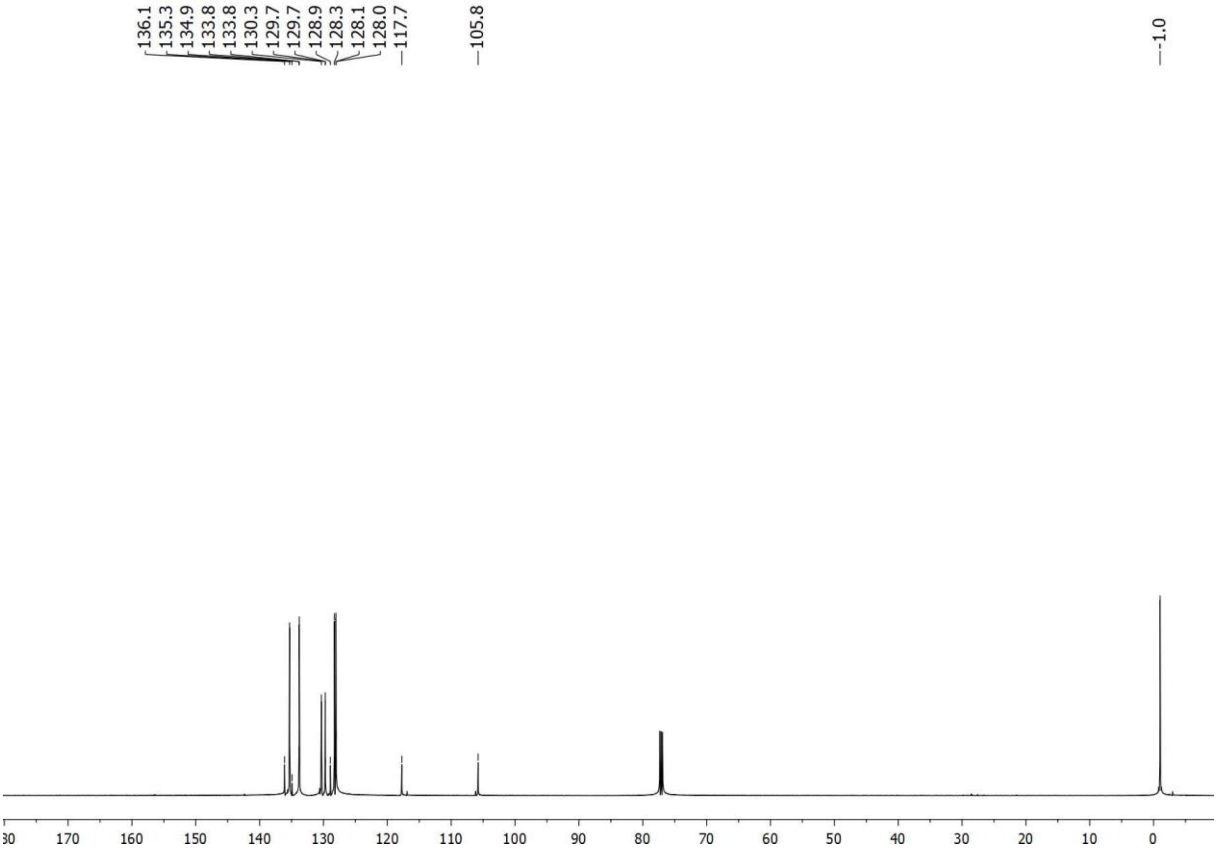

^{29}Si NMR

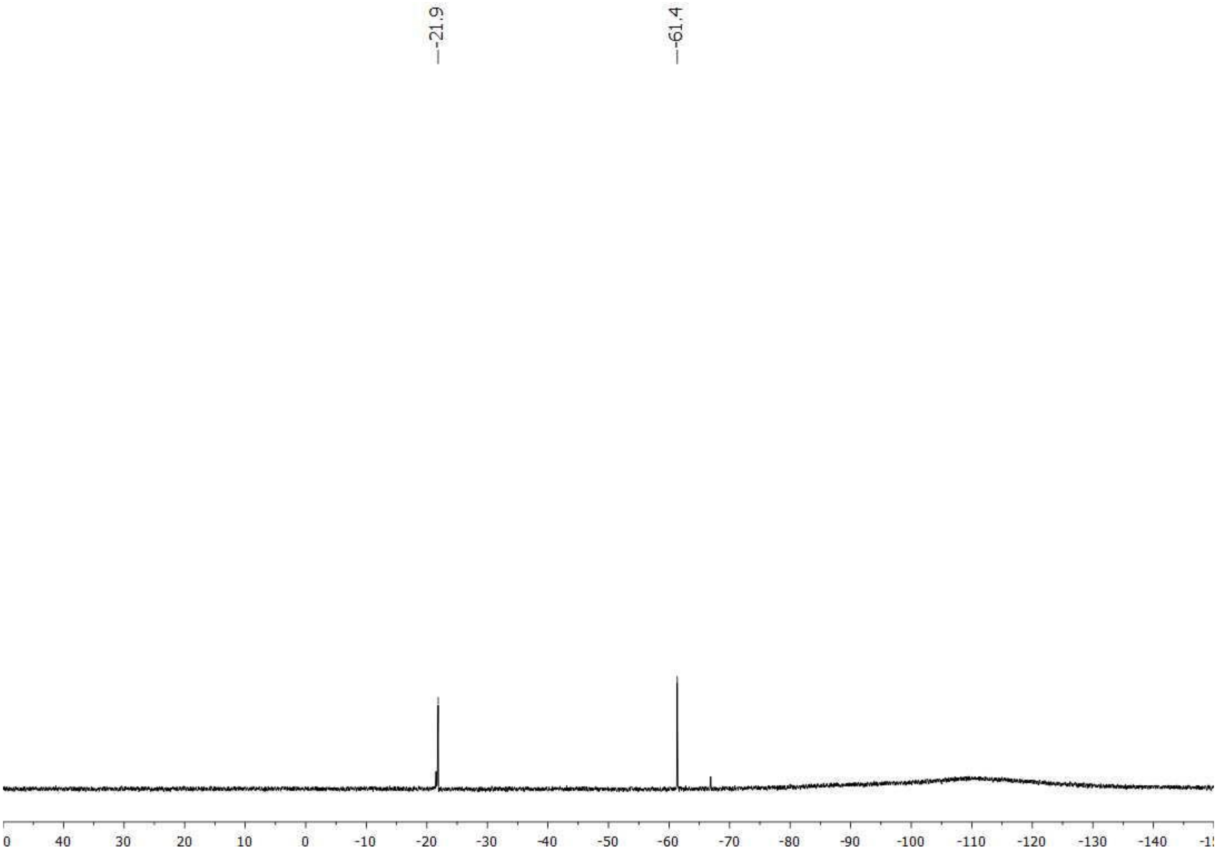

Methyl(phenyl)((phenylsilyl)ethynyl)(vinyl)silane (3aj)

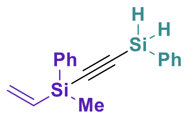

^{1}H NMR

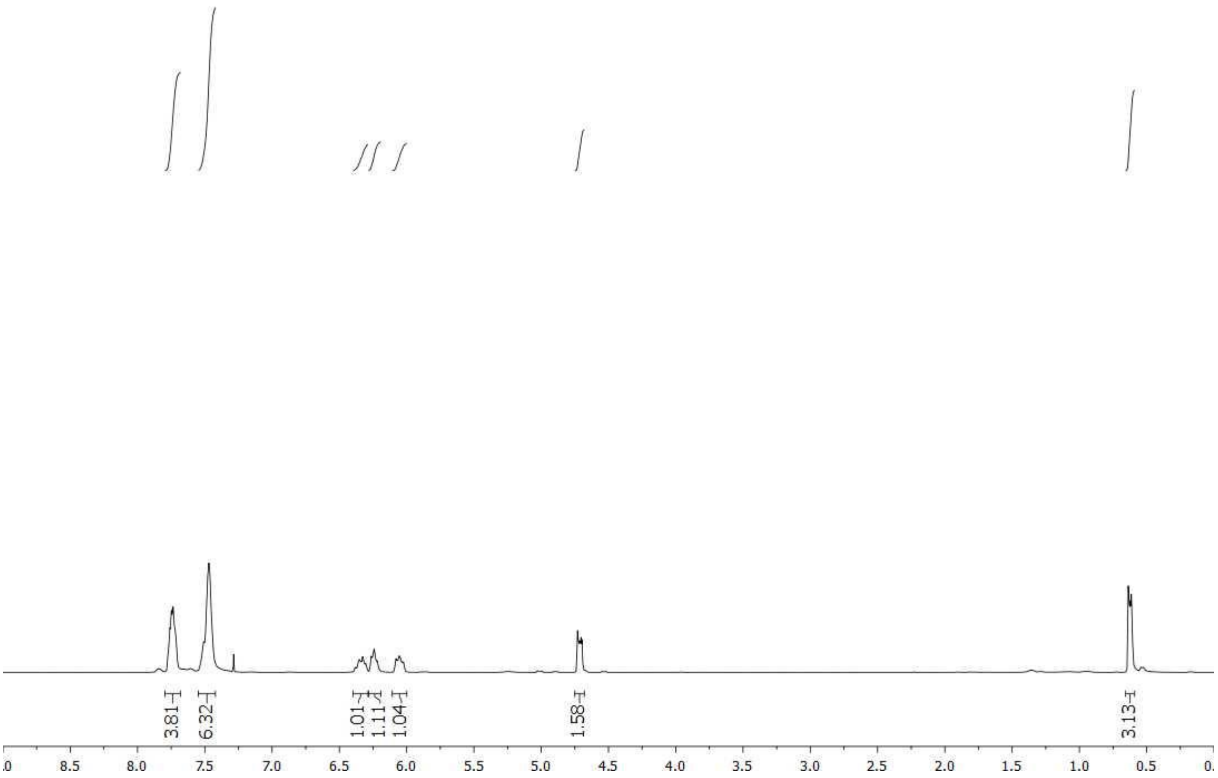

^{13}C NMR

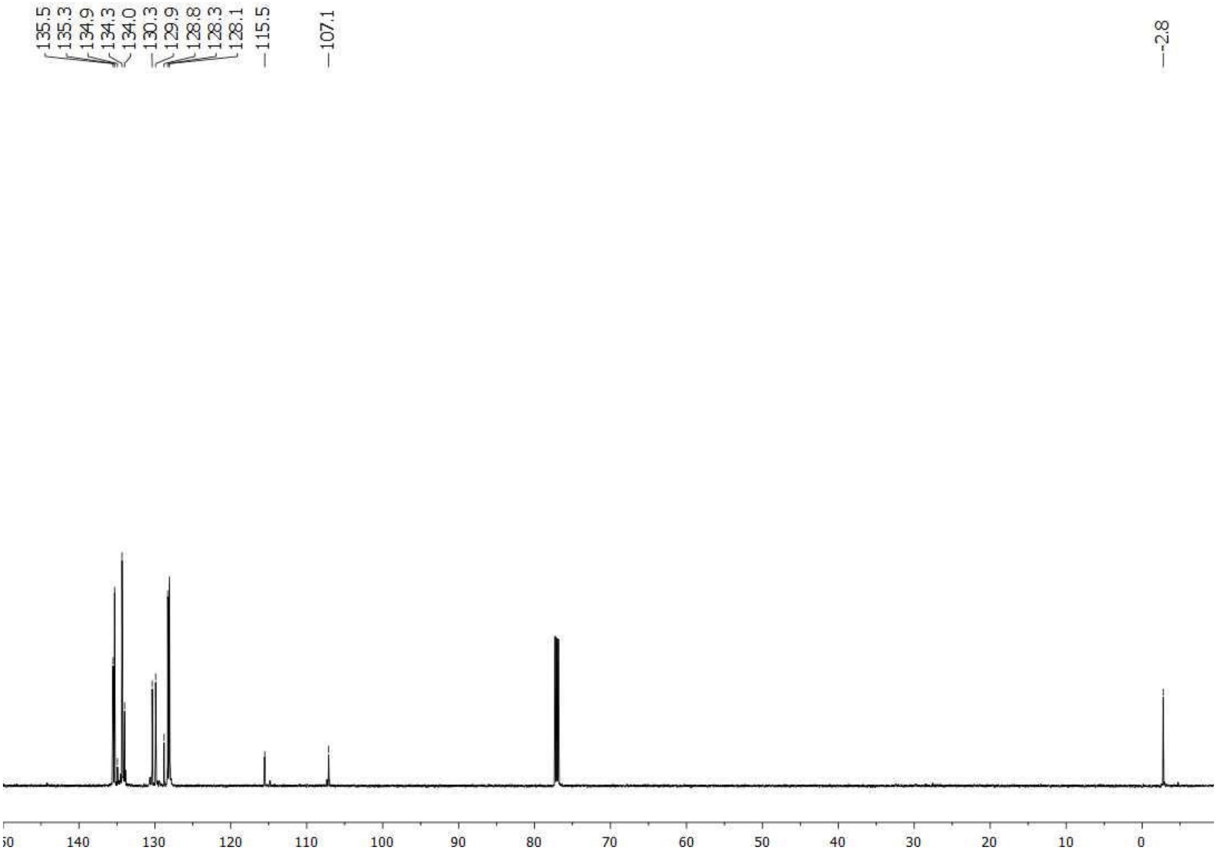

^{29}Si NMR

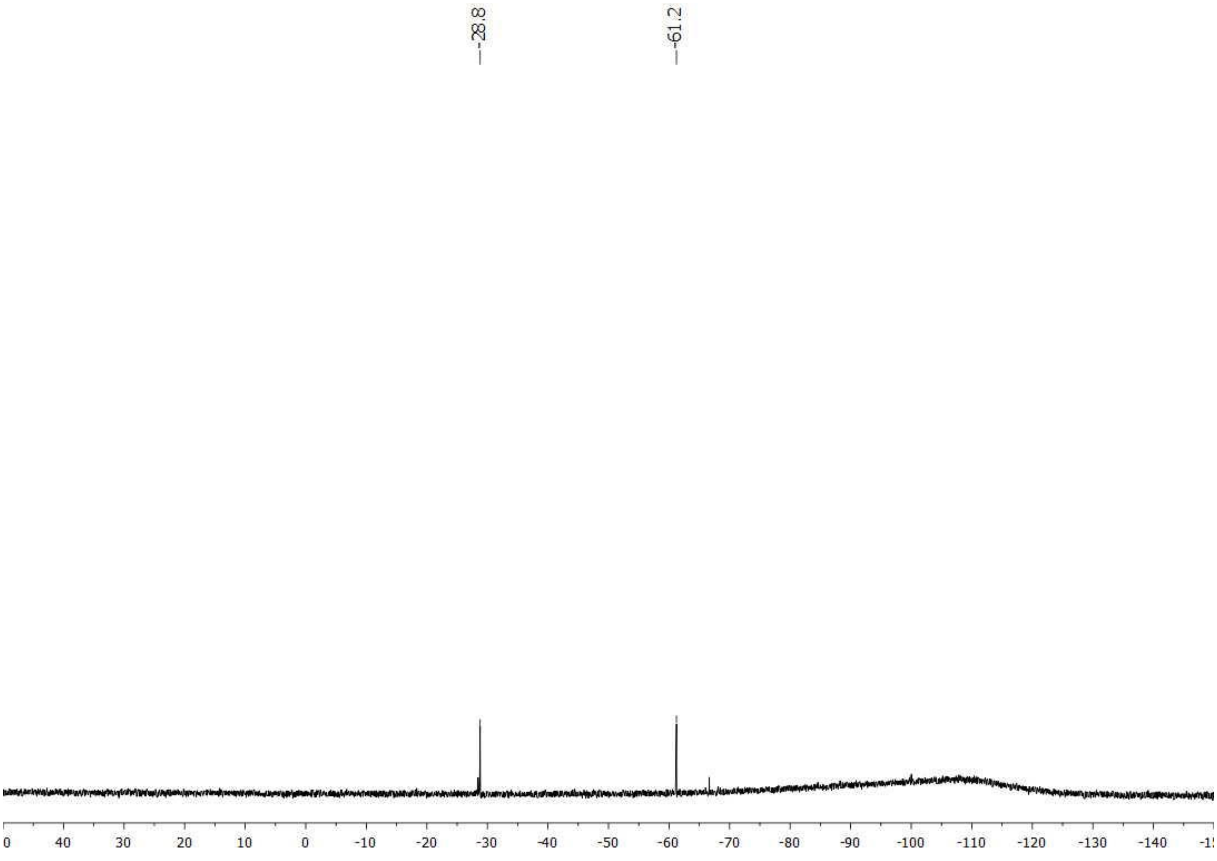

1,2-Bis(((phenylsilyl)ethynyl)dimethylsilyl)ethane (3ak)

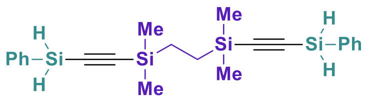

^{1}H NMR

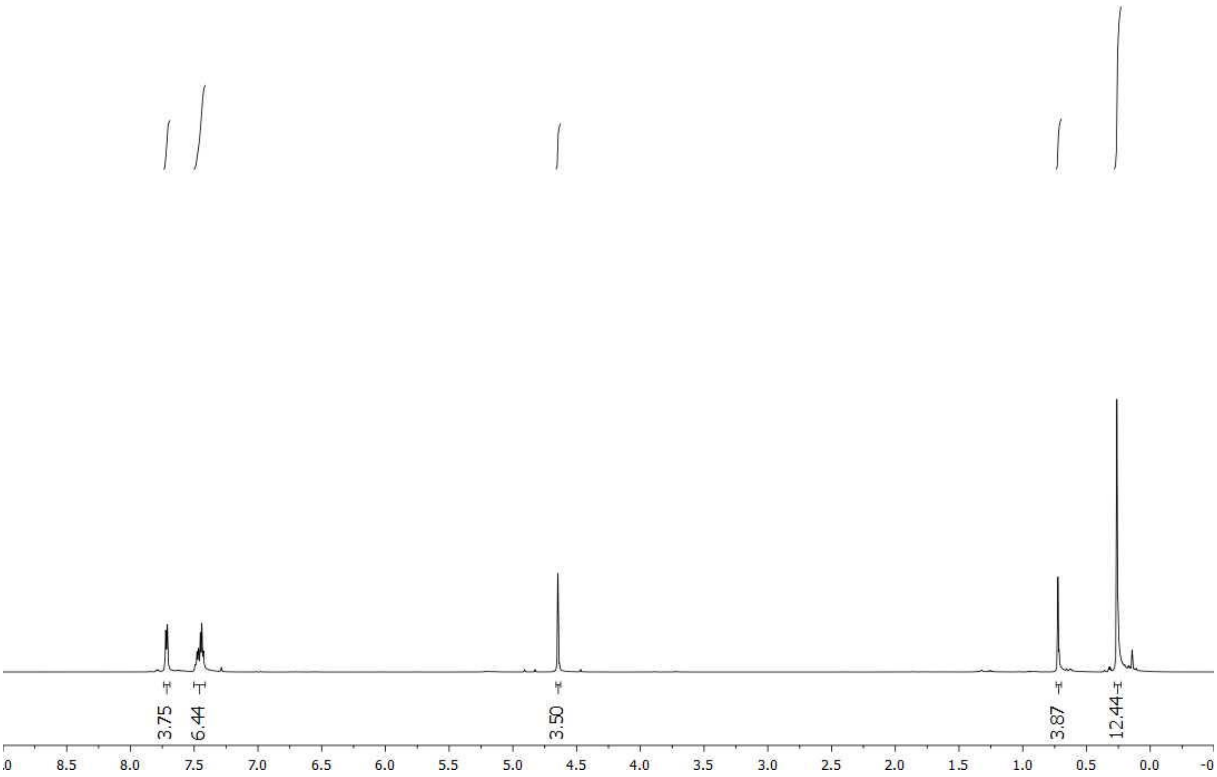

^{13}C NMR

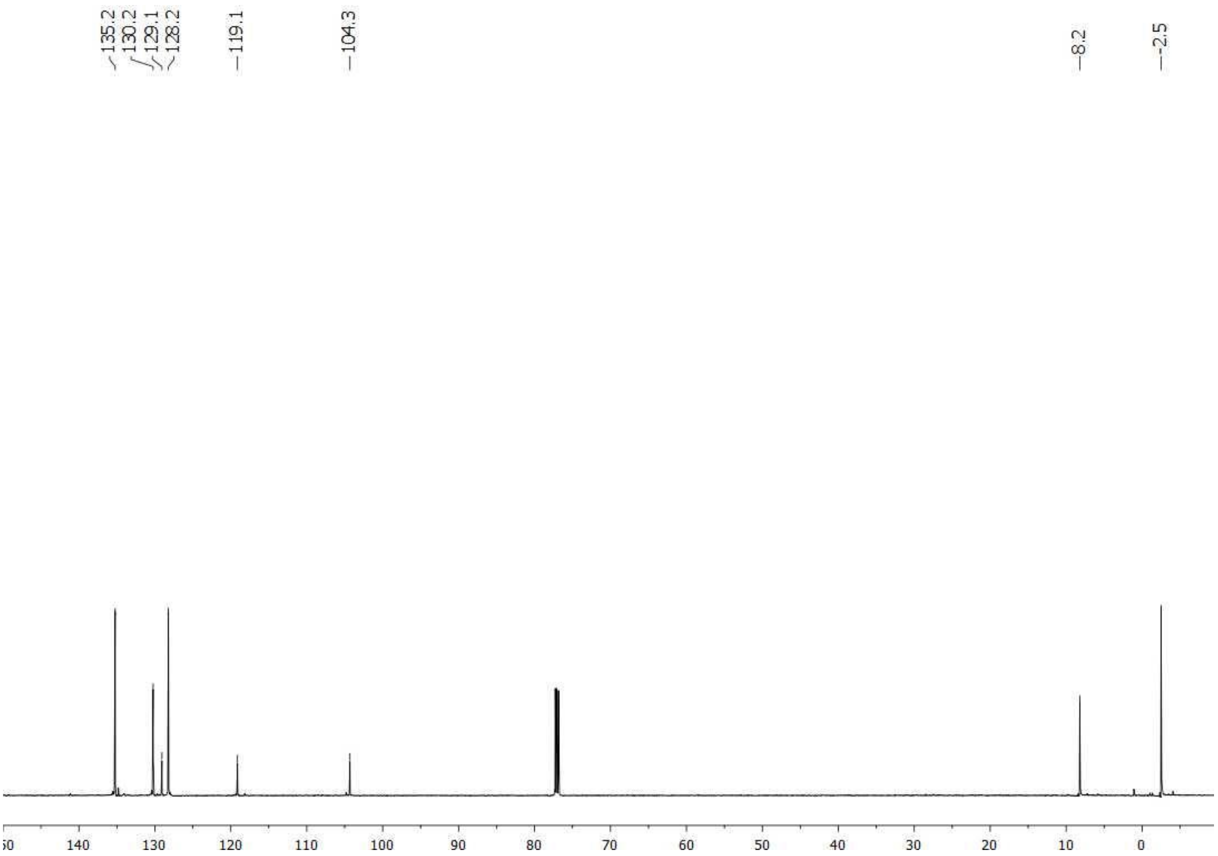

^{29}Si NMR

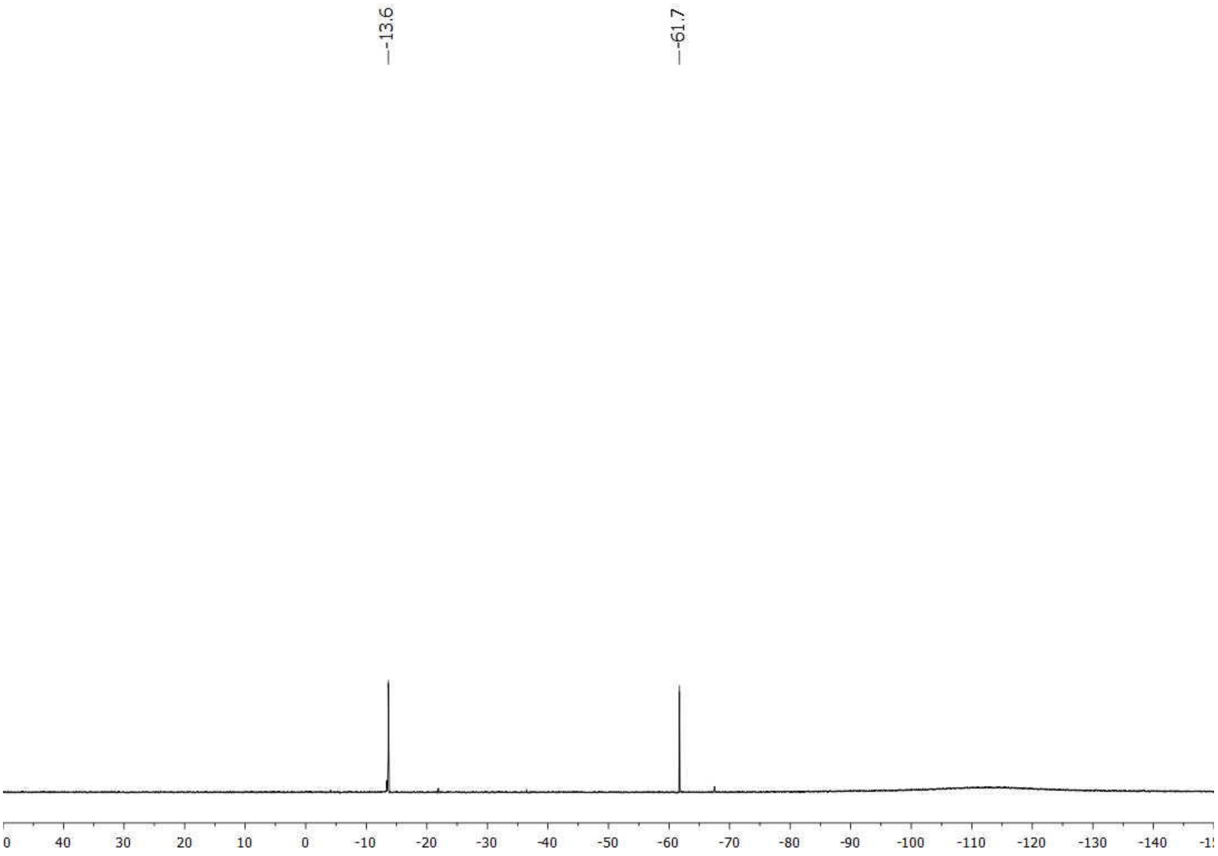

((Hexylsilyl)ethynyl)trimethylsilane (3ba)

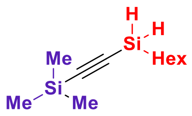

^{1}H NMR

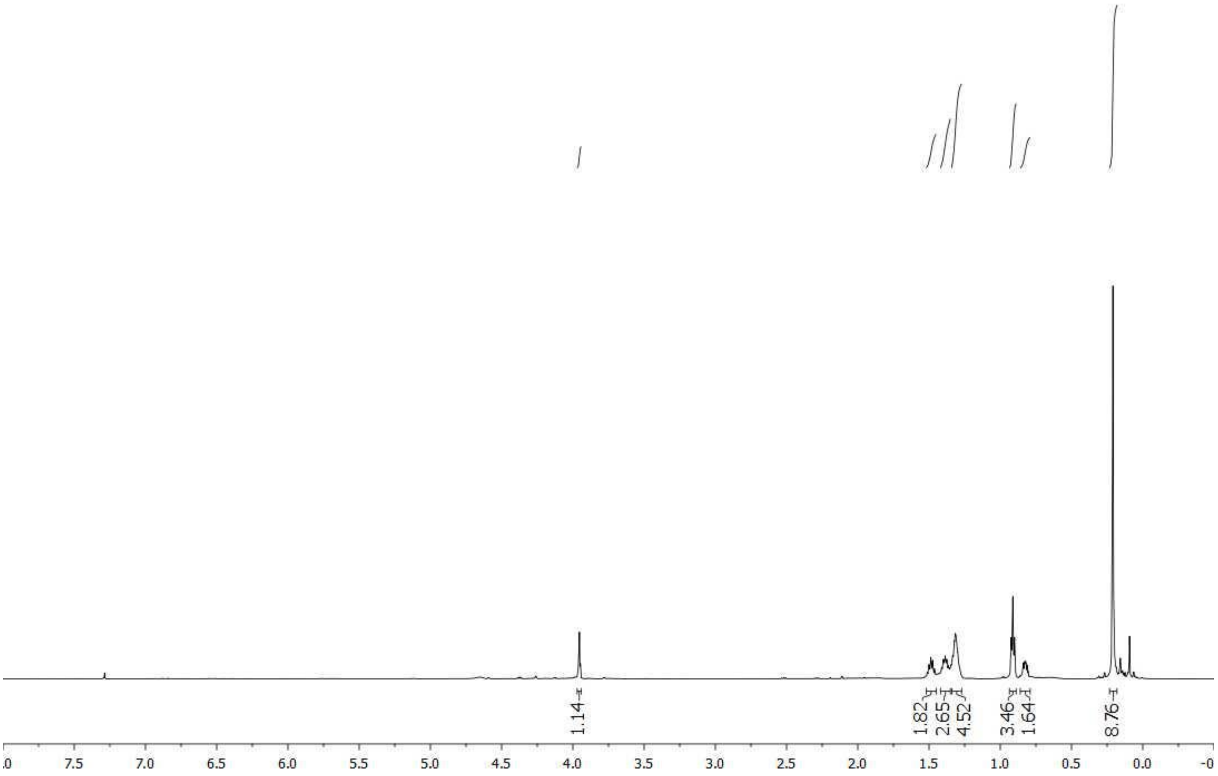

^{13}C NMR

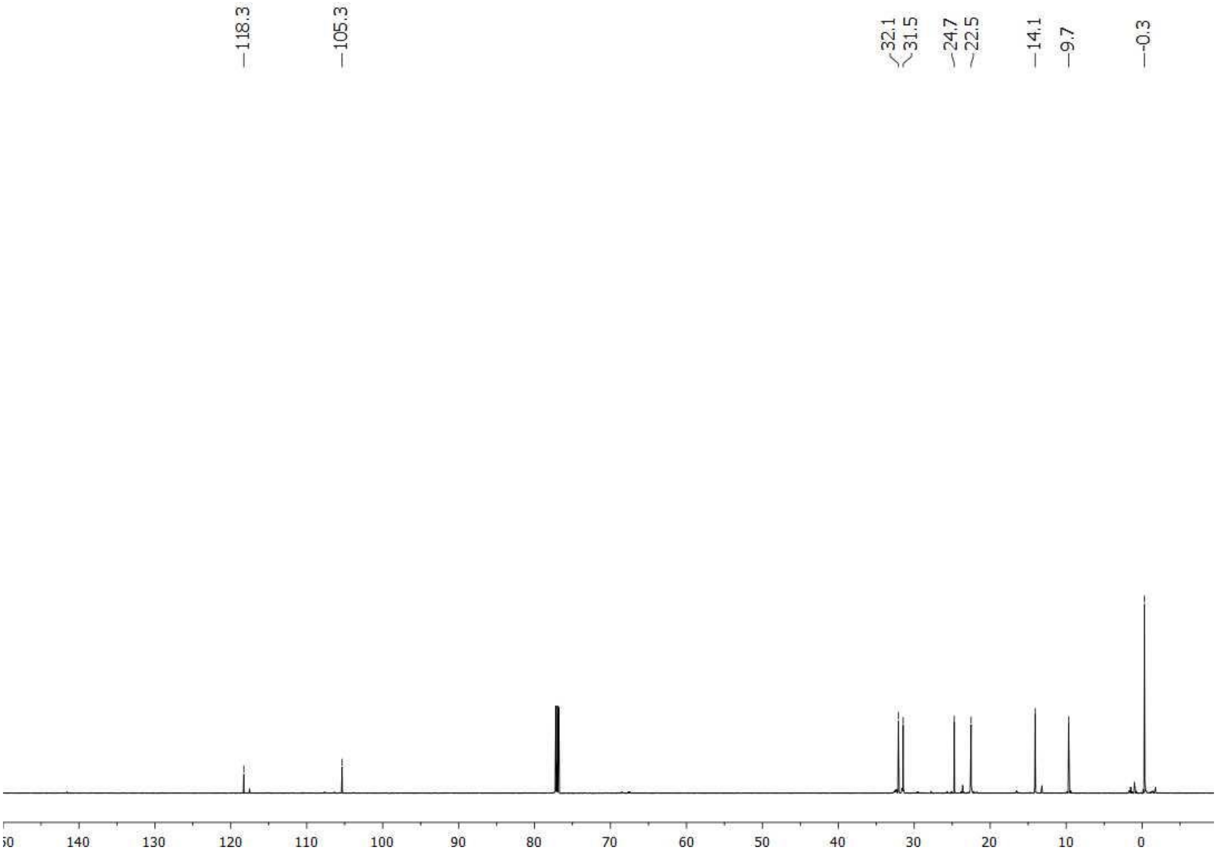

^{29}Si NMR

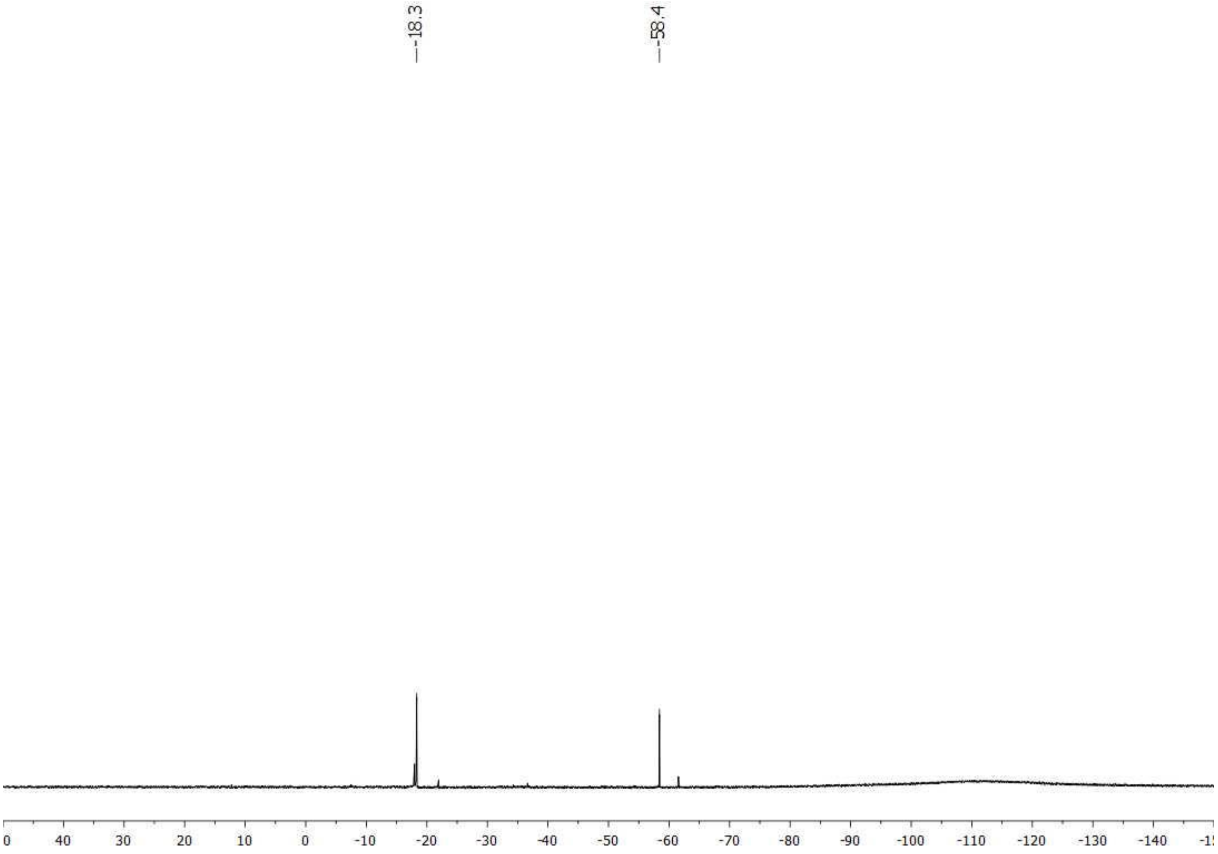

Triethyl((hexylsilyl)ethynyl)silane (3bb)

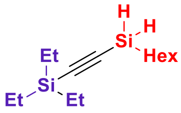

^{1}H NMR

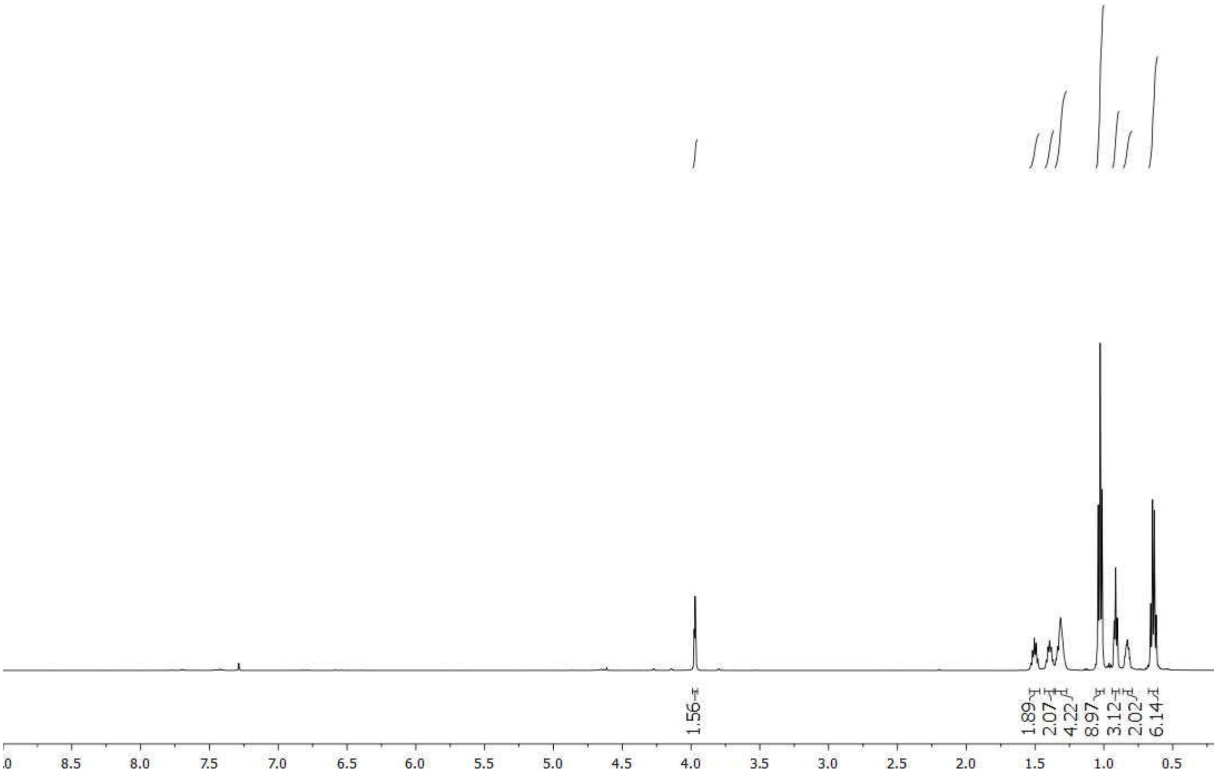

^{13}C NMR

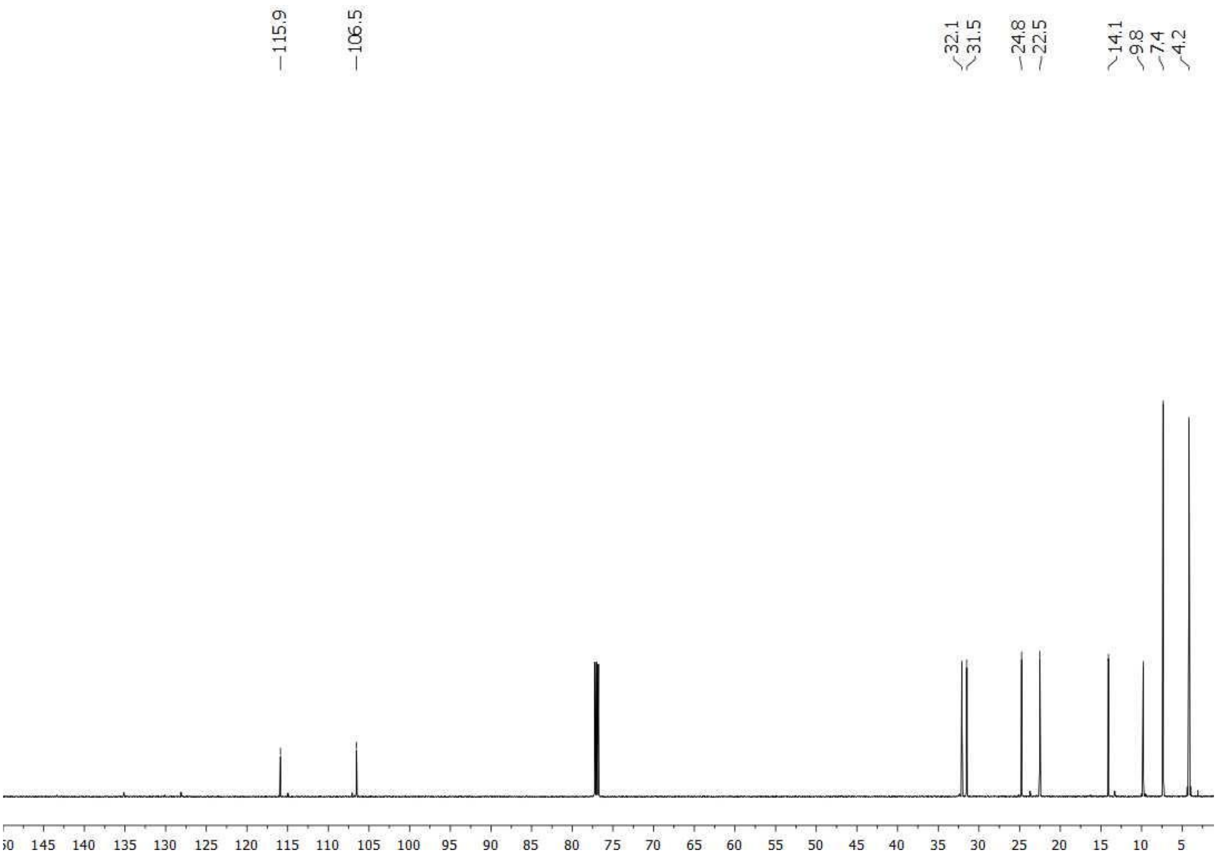

^{29}Si NMR

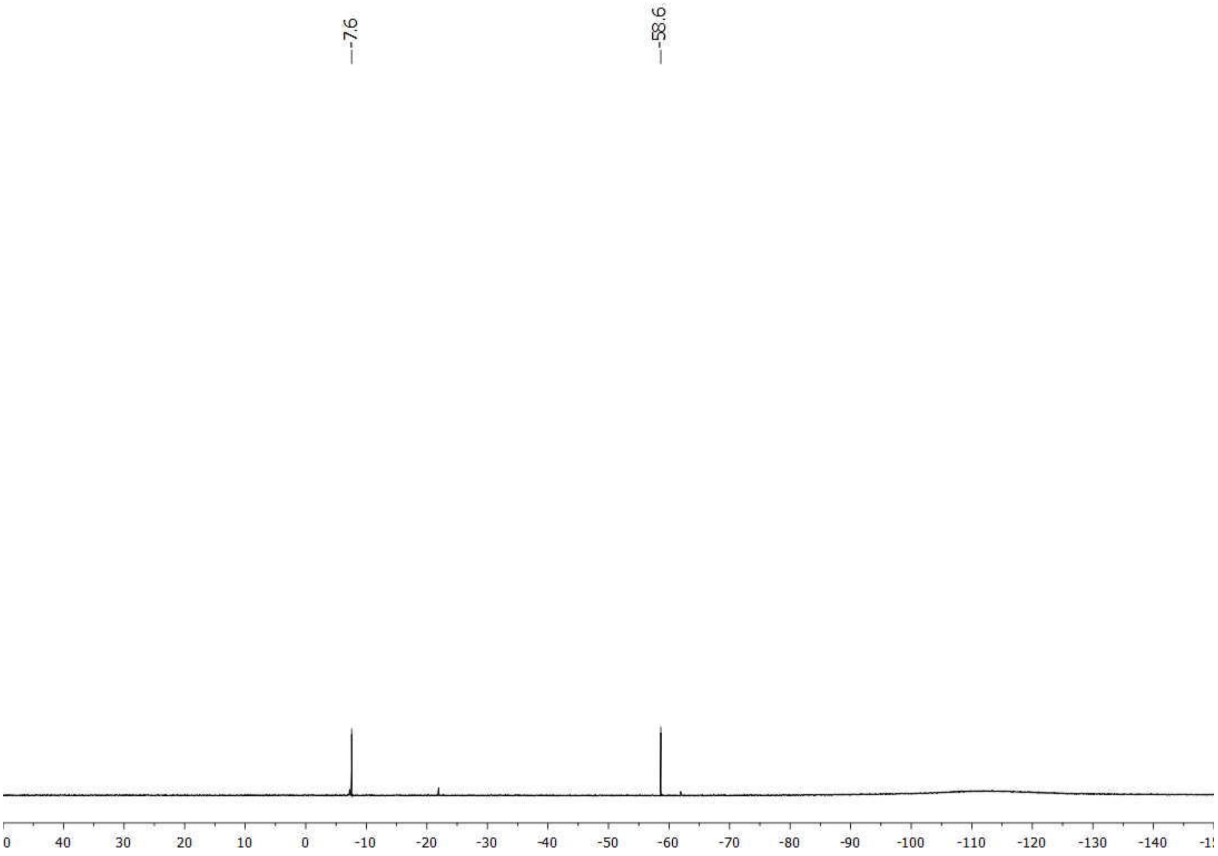

((Hexylsilyl)ethynyl)triisopropylsilane (3bc)

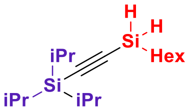

^{1}H NMR

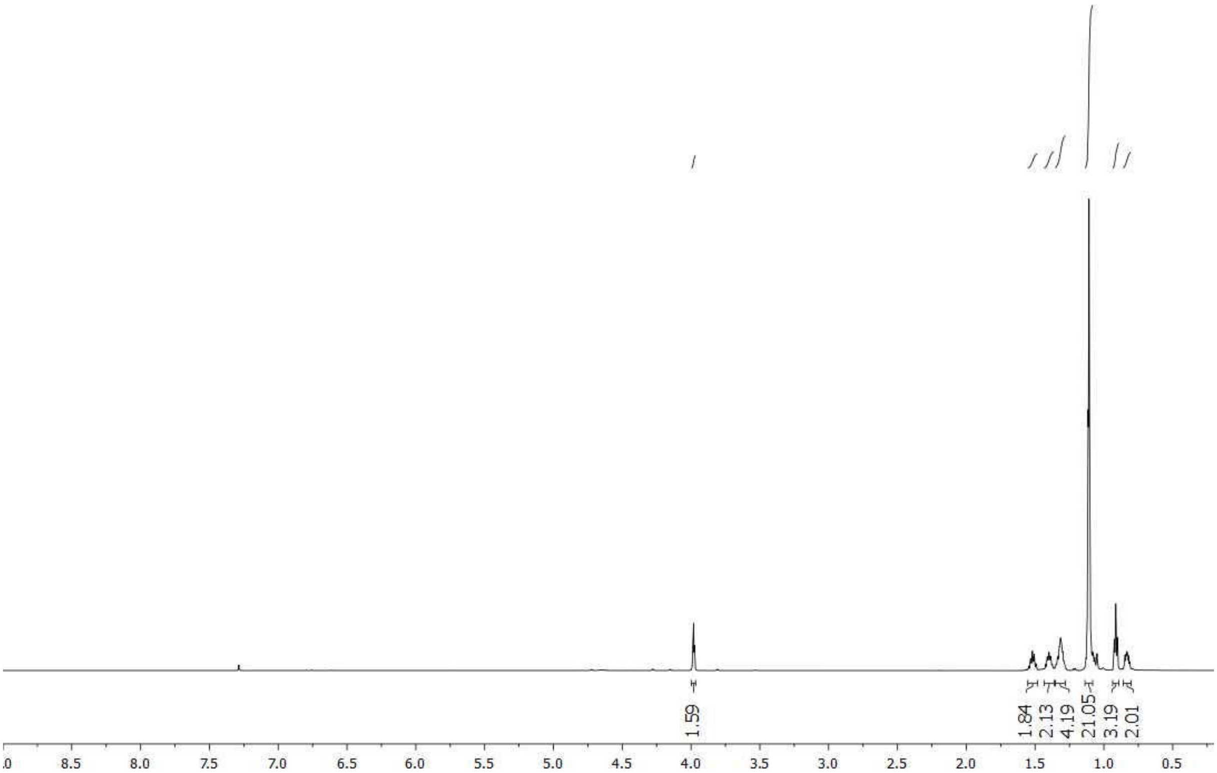

^{13}C NMR

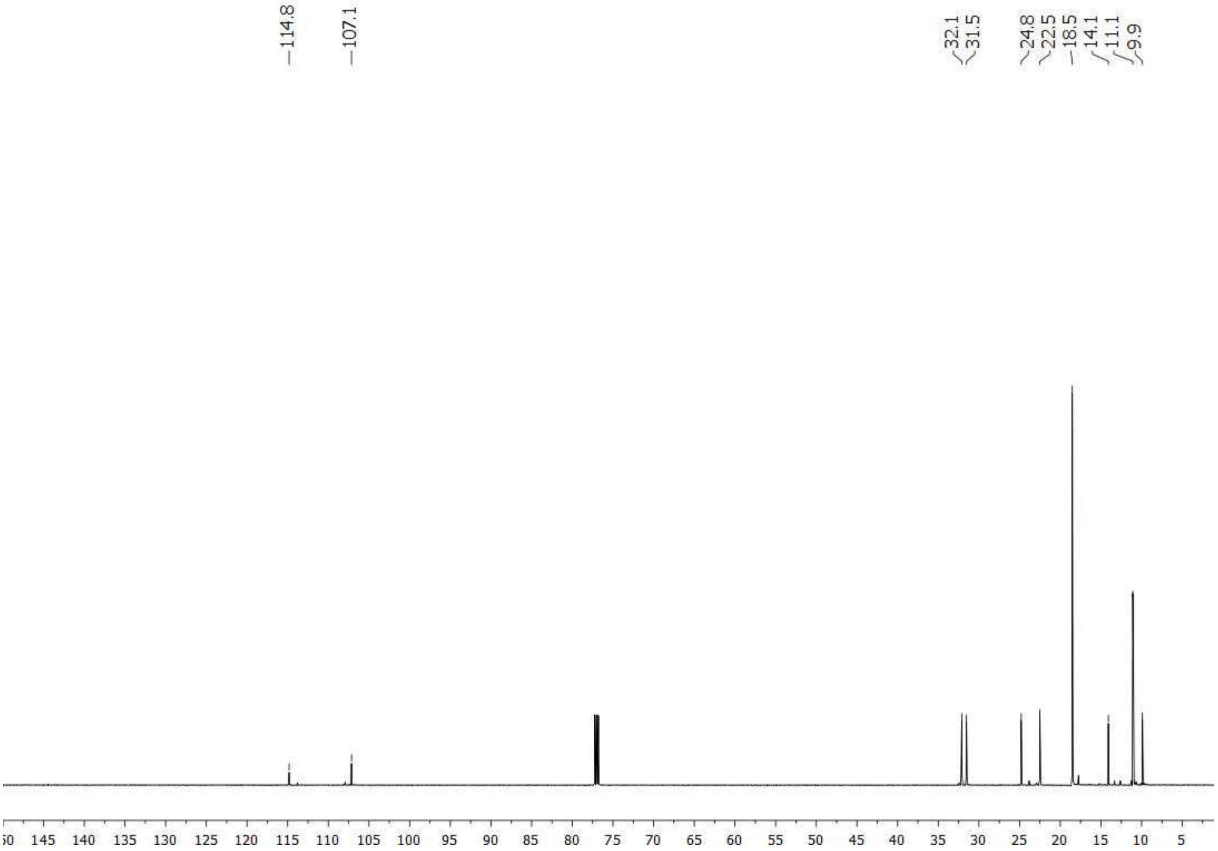

^{29}Si NMR

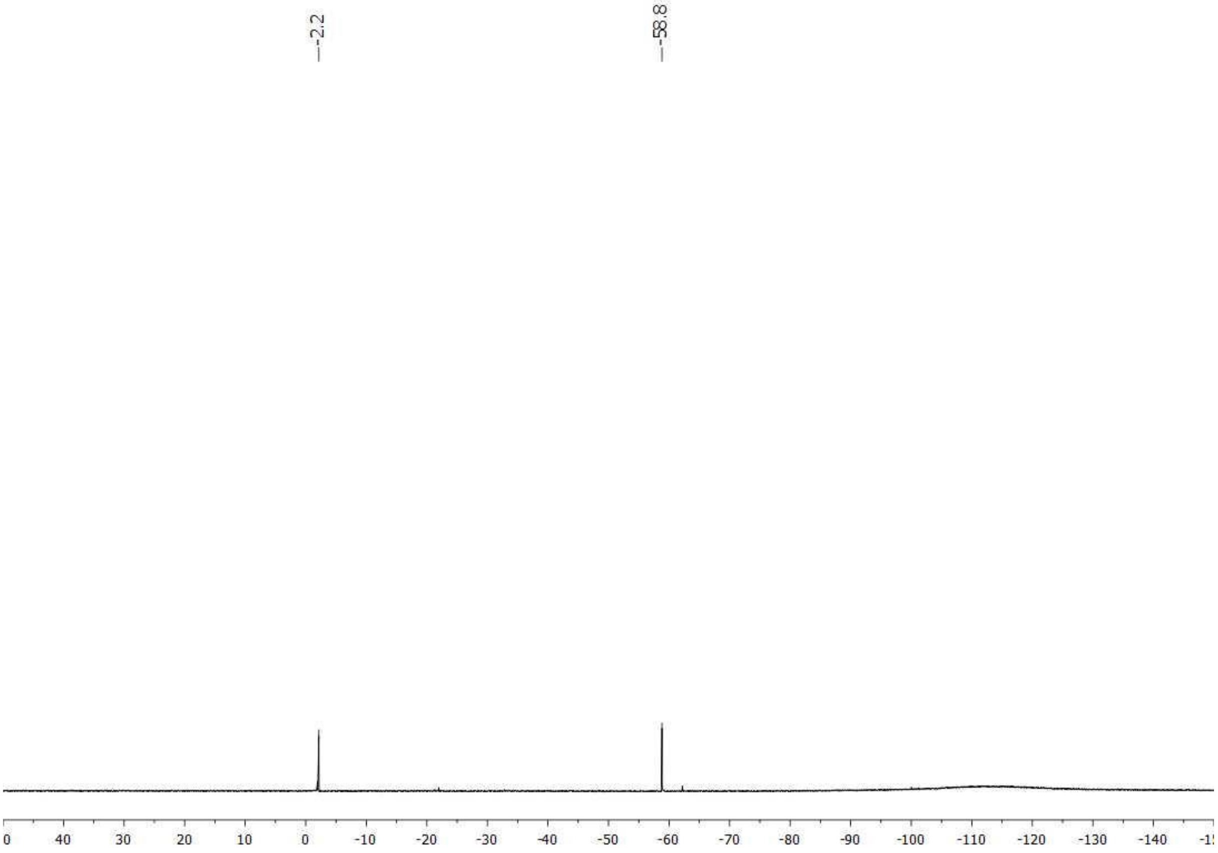

((Hexylsilyl)ethynyl)diisopropylsilane (3bd)

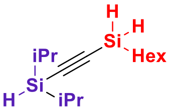

^{1}H NMR

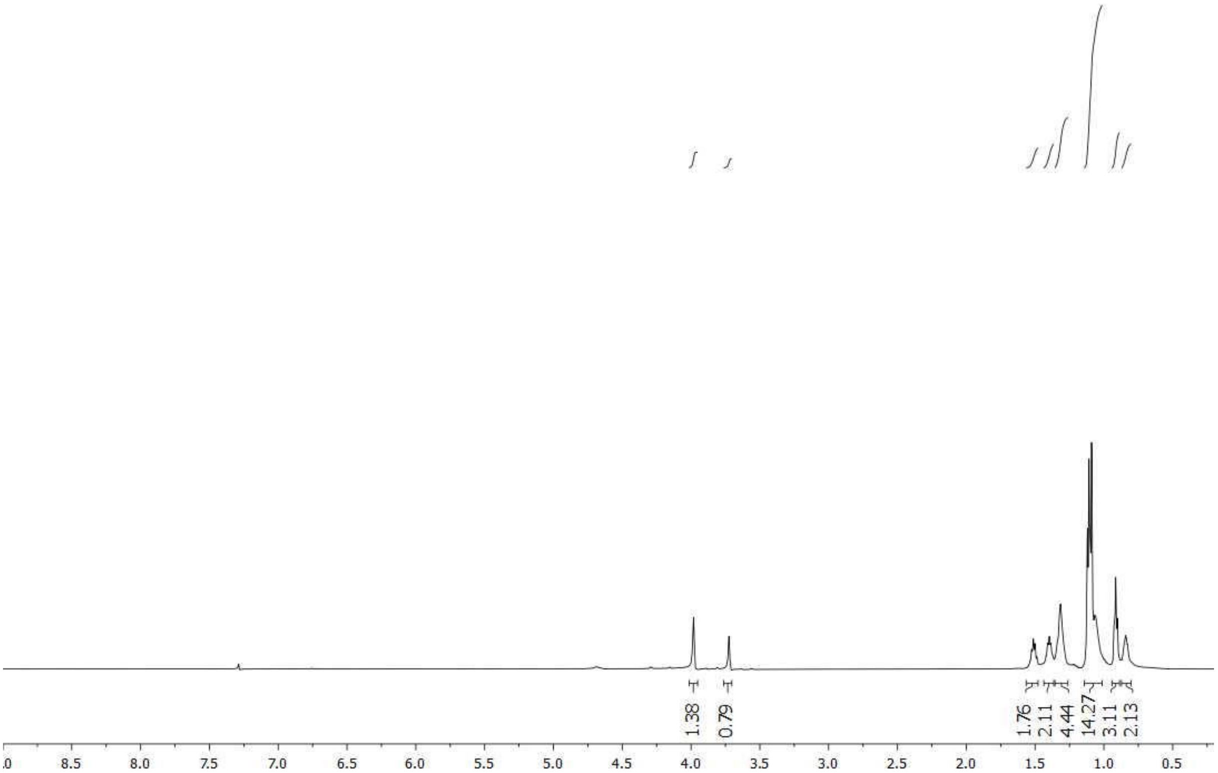

13C NMR

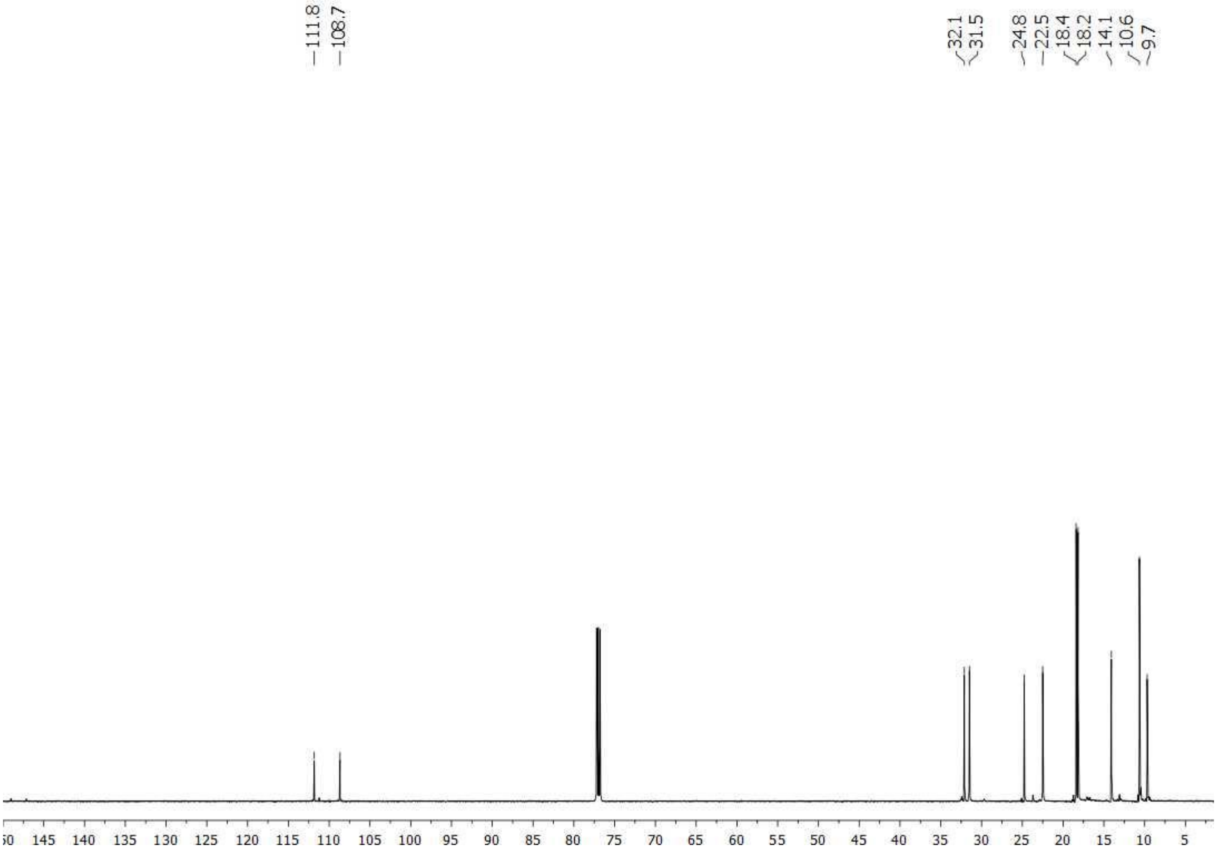

29Si NMR

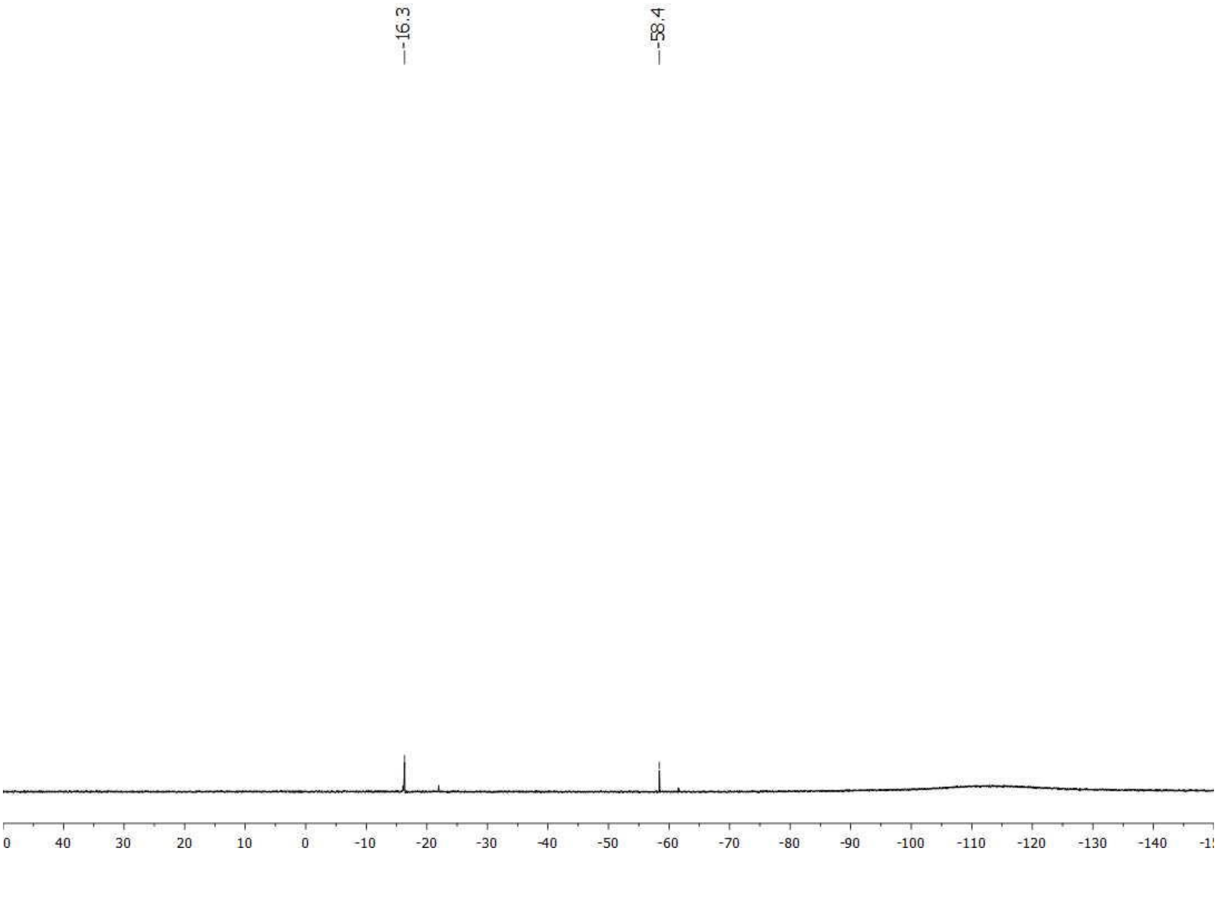

((Hexylsilyl)ethynyl)tripropylsilane (3be)

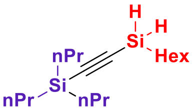

^{1}H NMR

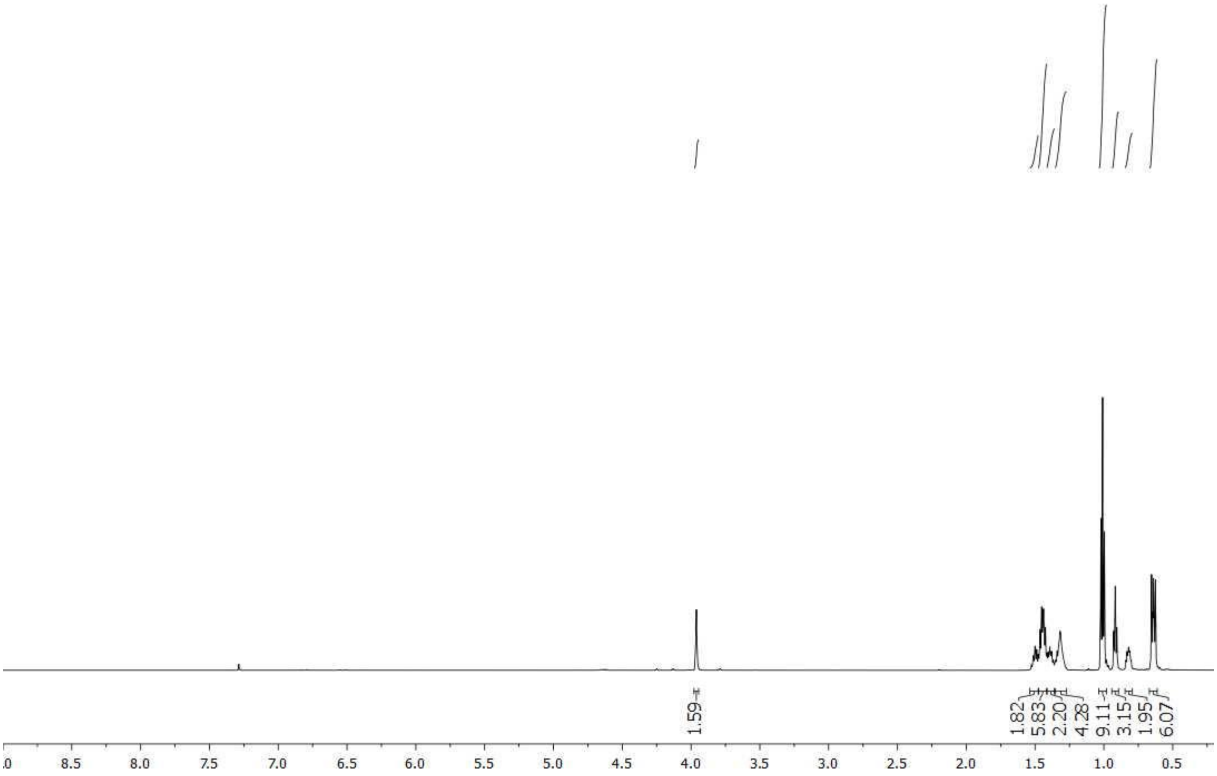

^{13}C NMR

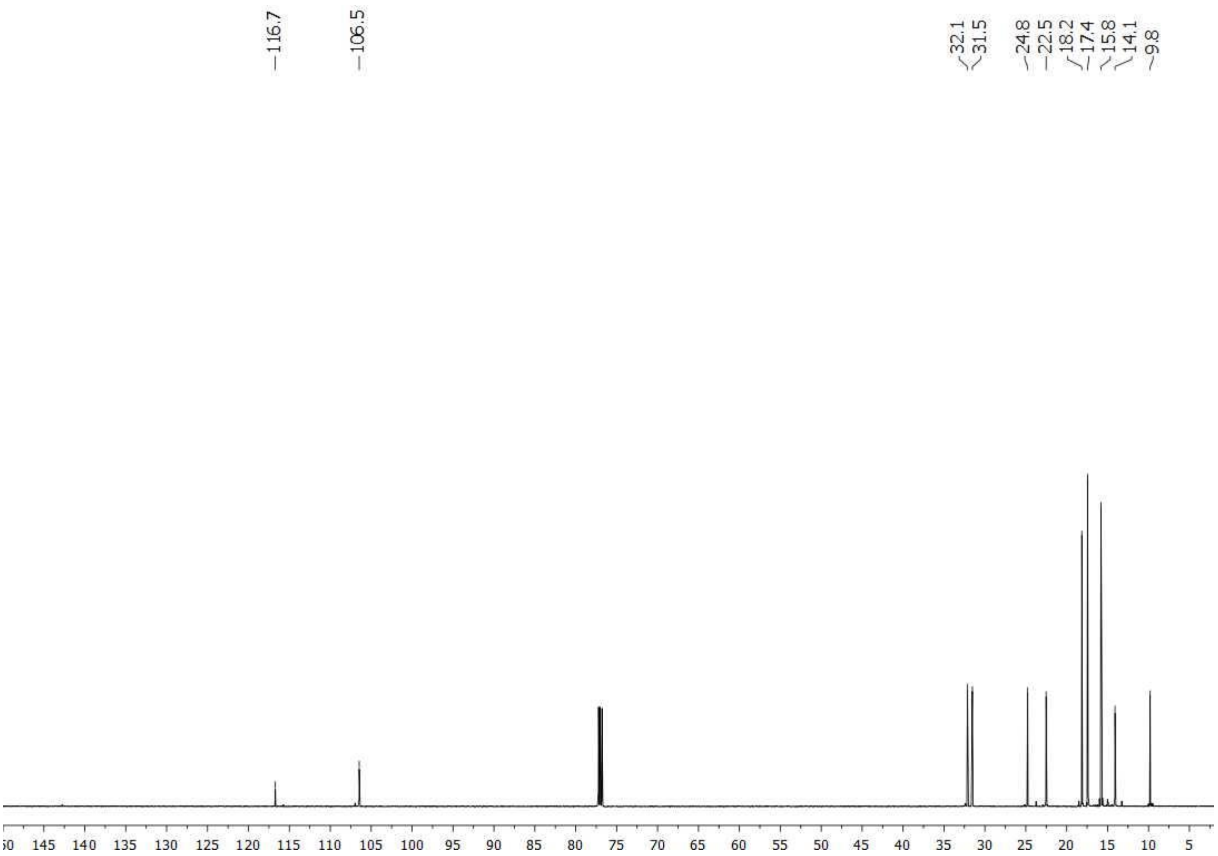

^{29}Si NMR

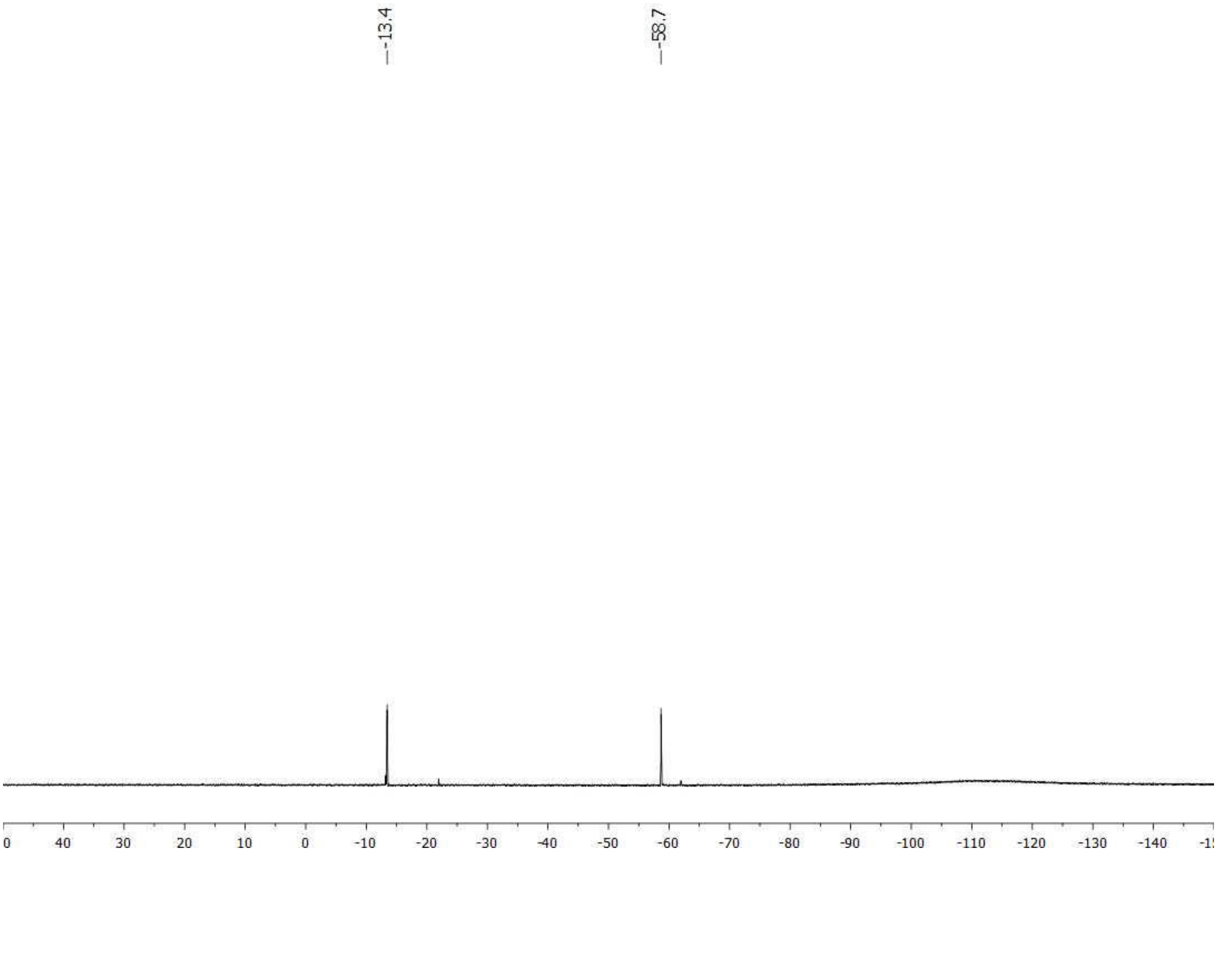

((Hexylsilyl)ethynyl)triisobutylsilane (3bf)

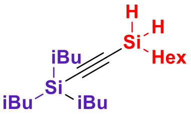

^{1}H NMR

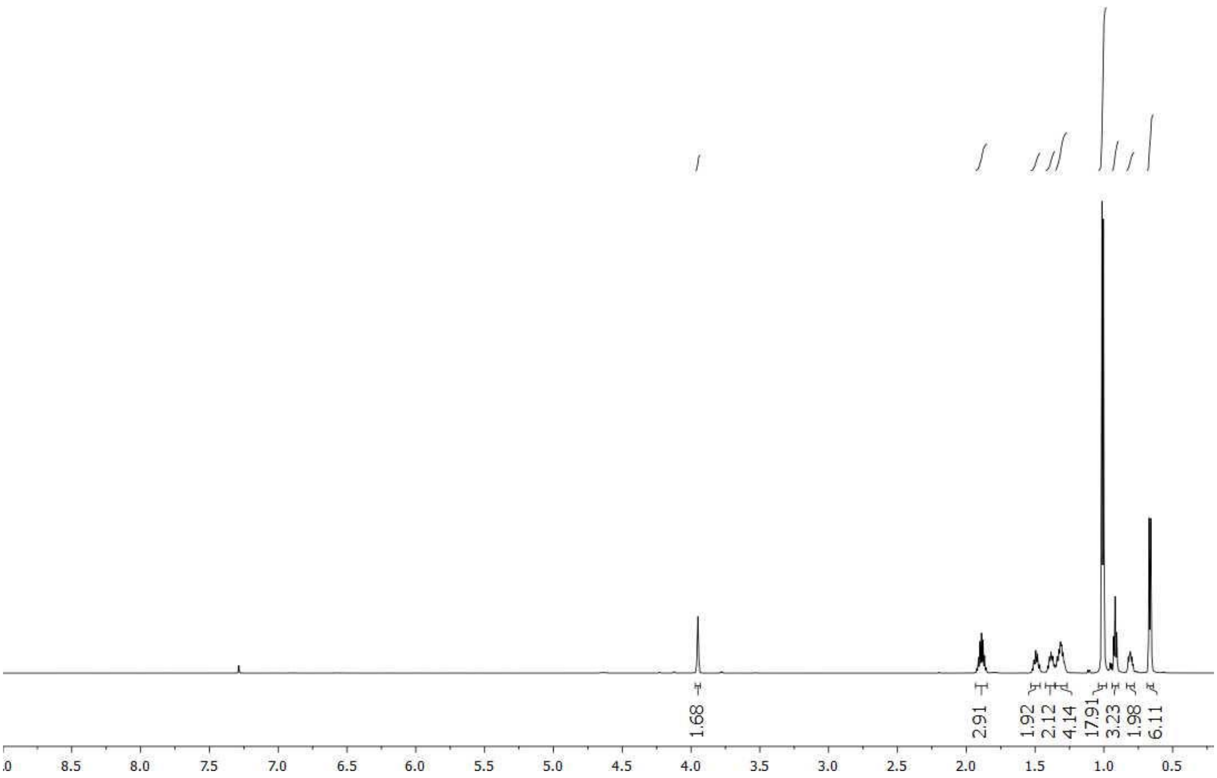

^{13}C NMR

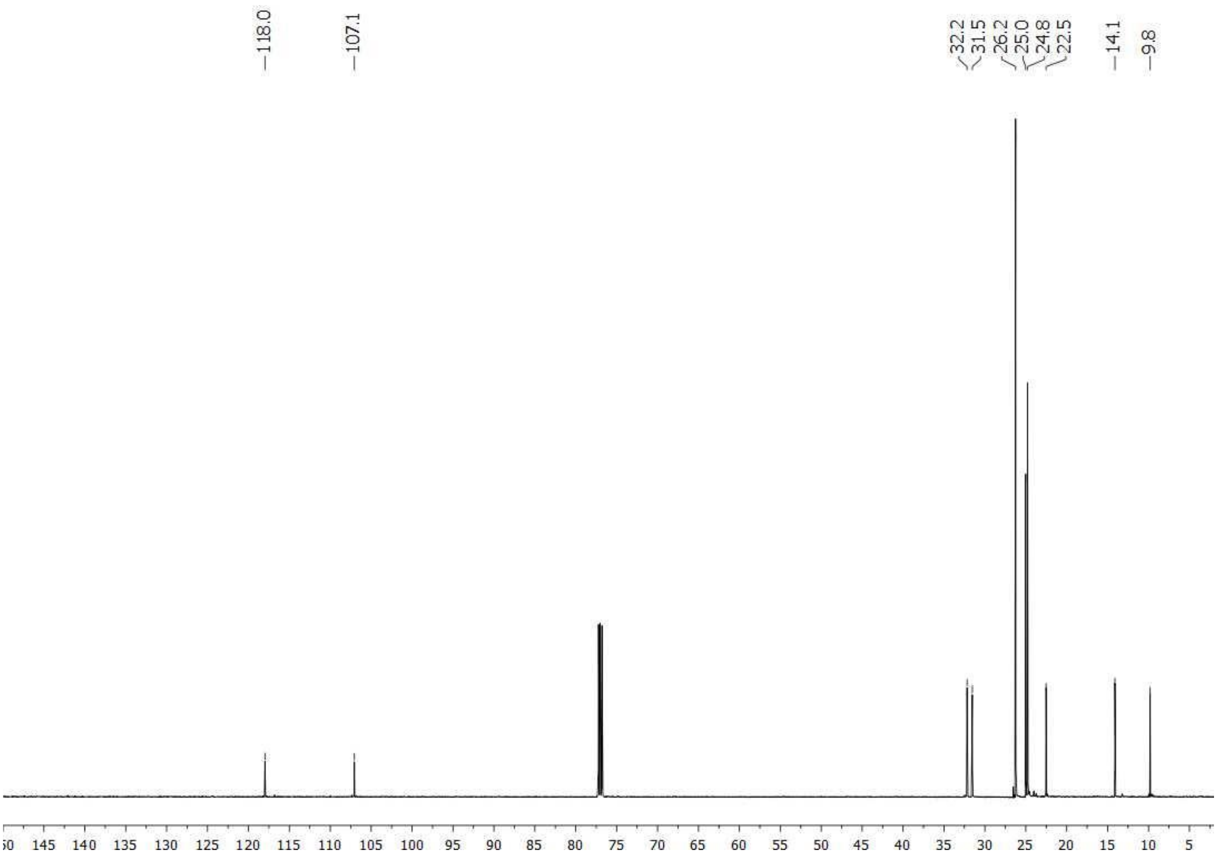

^{29}Si NMR

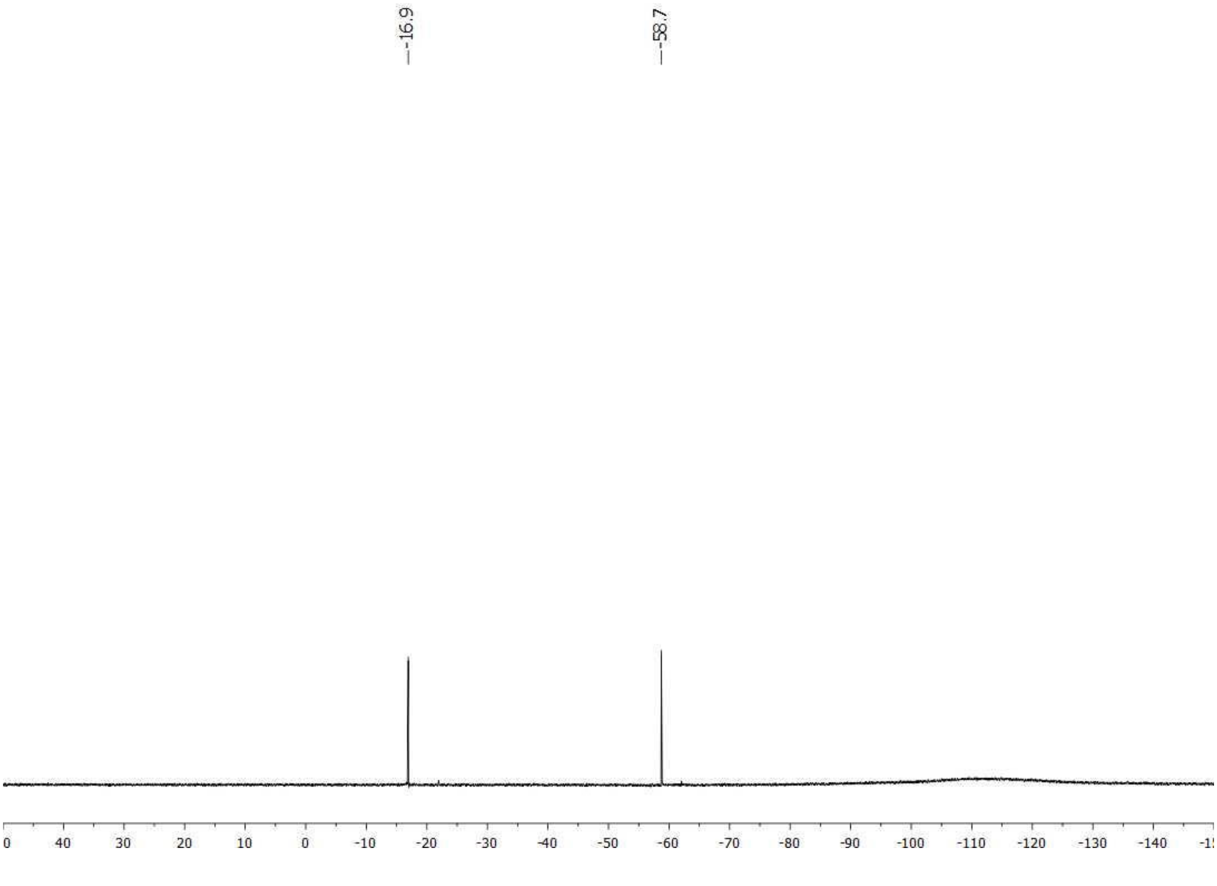

Tert-butyl((hexylsilyl)ethynyl)dimethylsilane (3bg)

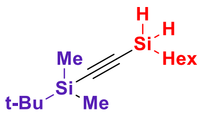

^{1}H NMR

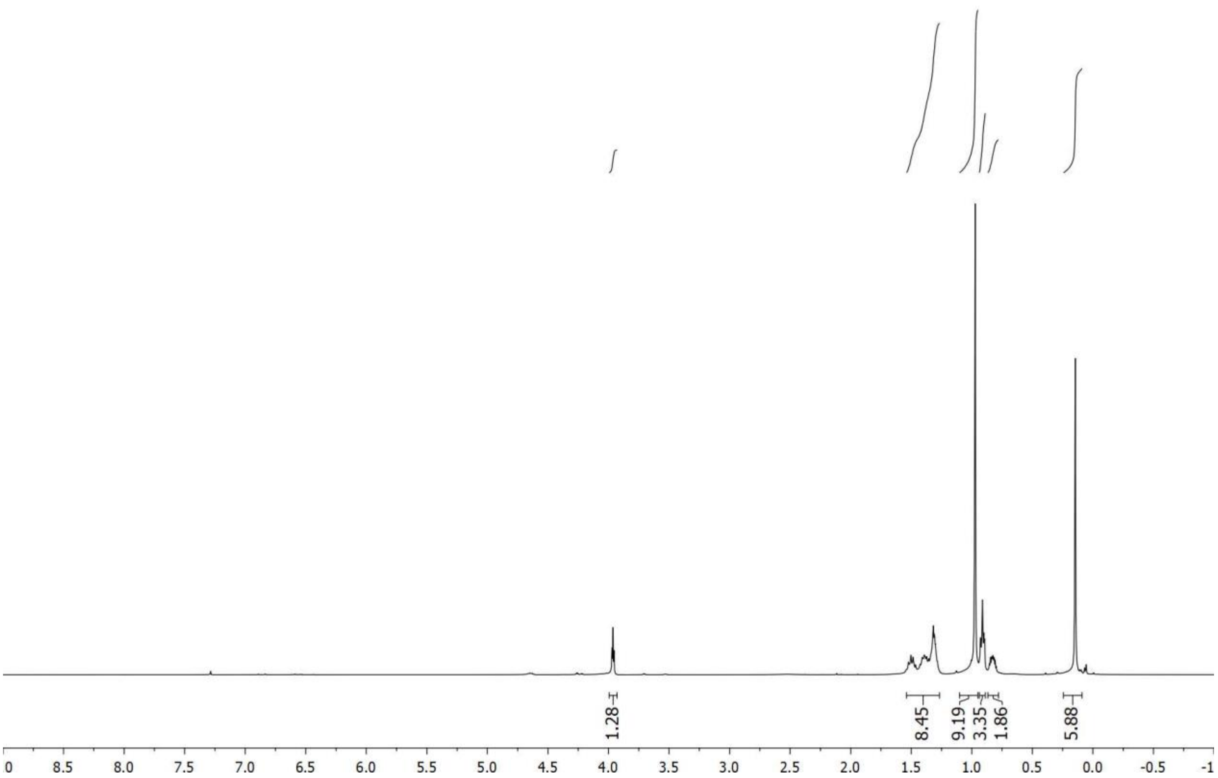

^{13}C NMR

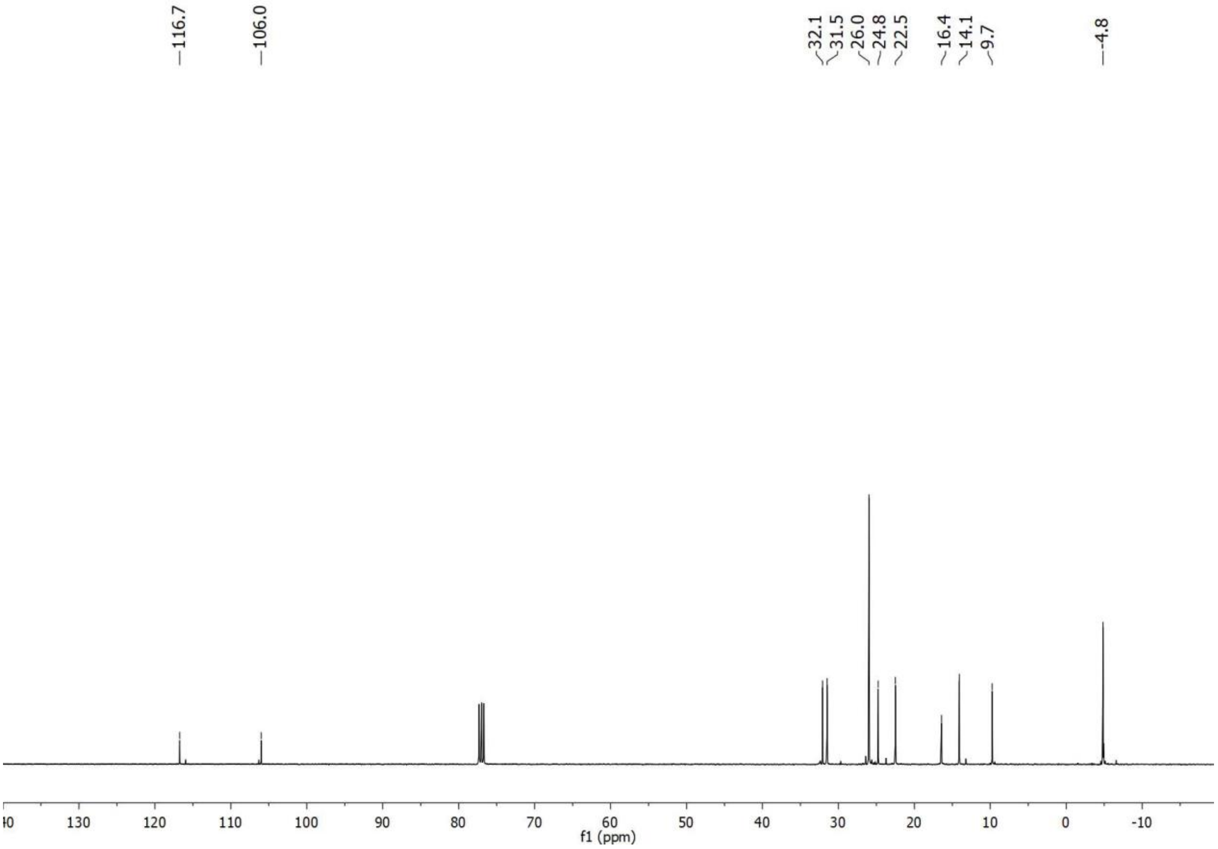

^{29}Si NMR

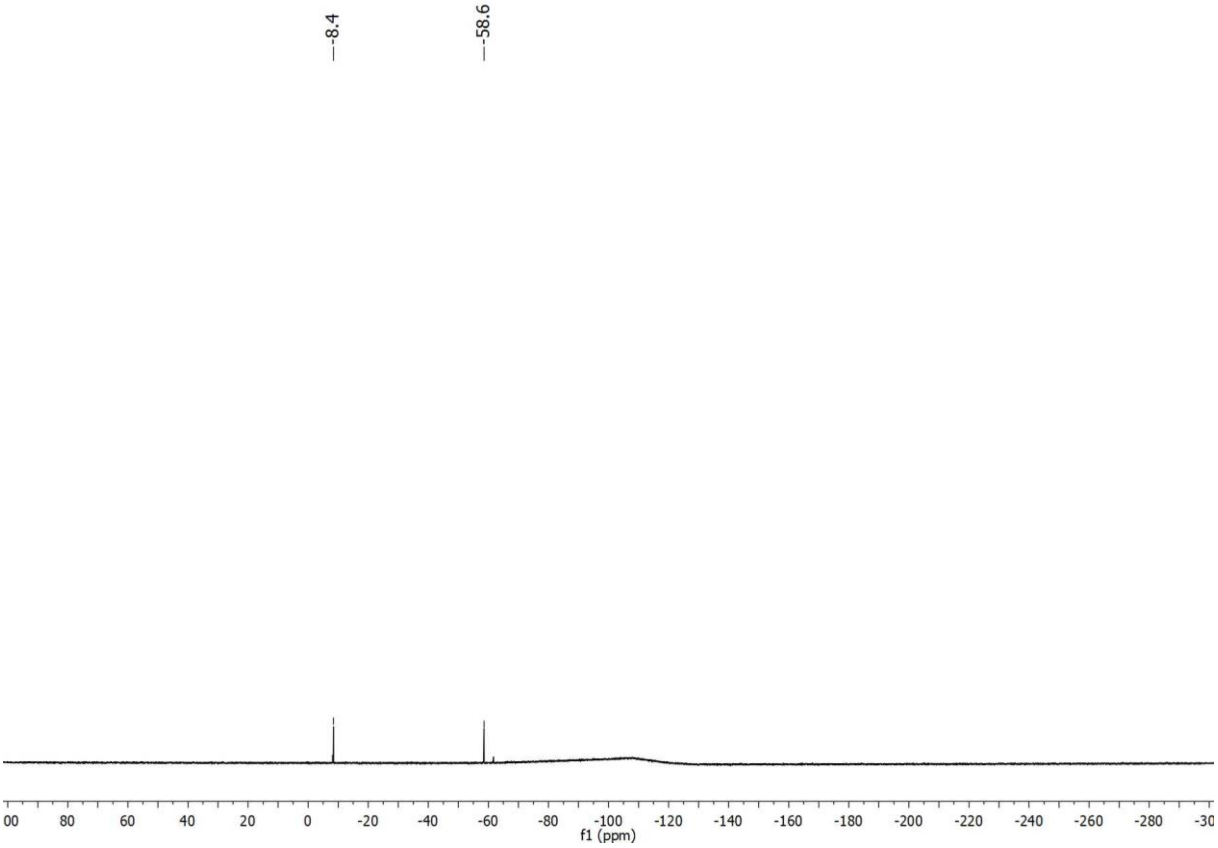

((Hexylsilyl)ethynyl)dimethyl(octyl)silane (3bh)

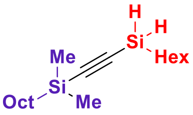

^{1}H NMR

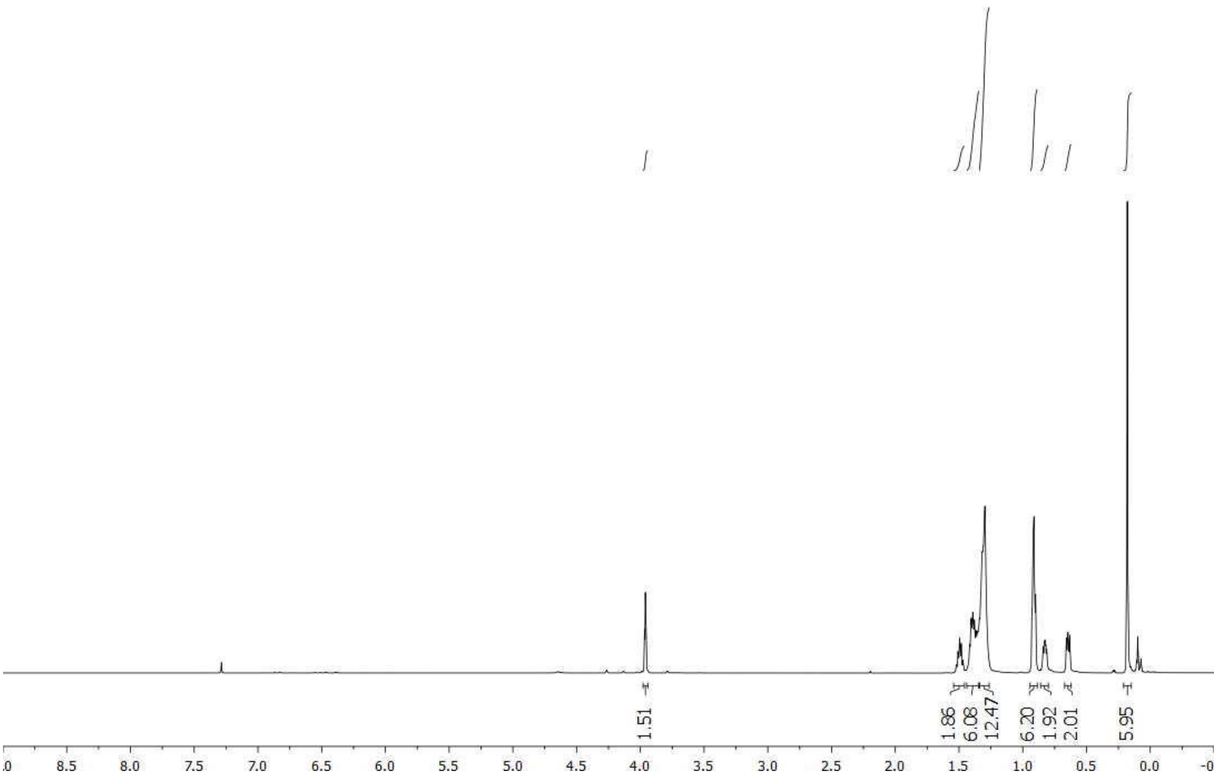

^{13}C NMR

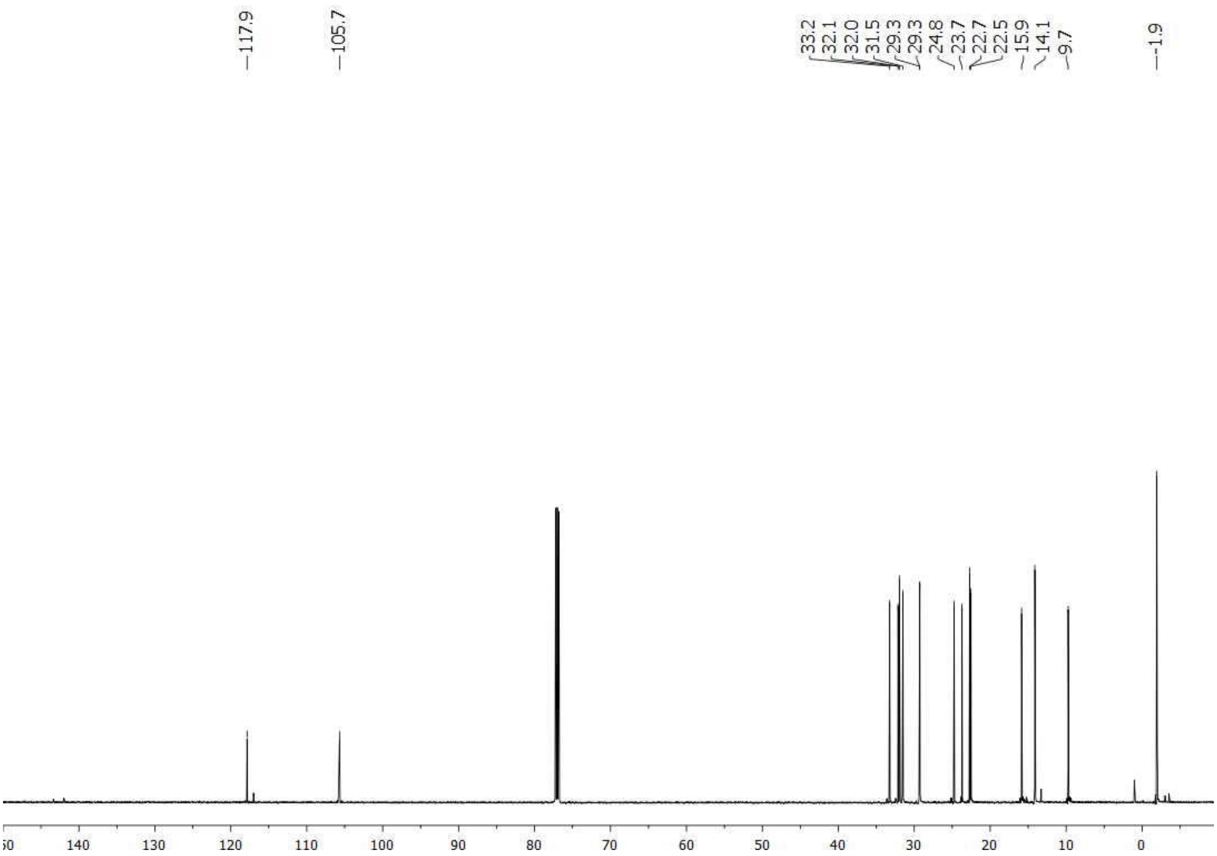

^{29}Si NMR

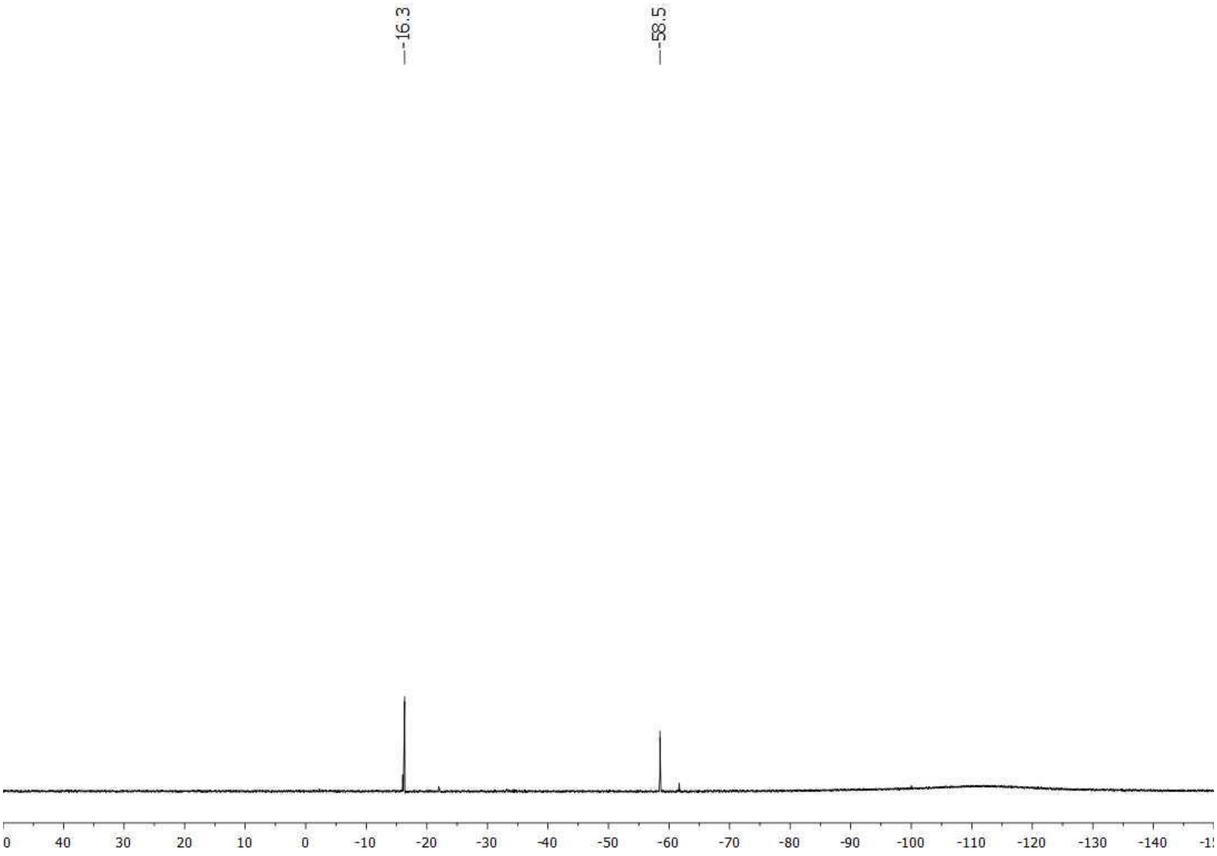

((Hexylsilyl)ethynyl)dimethyl(phenyl)silane (3bi)

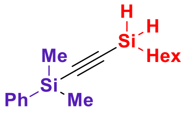

^{1}H NMR

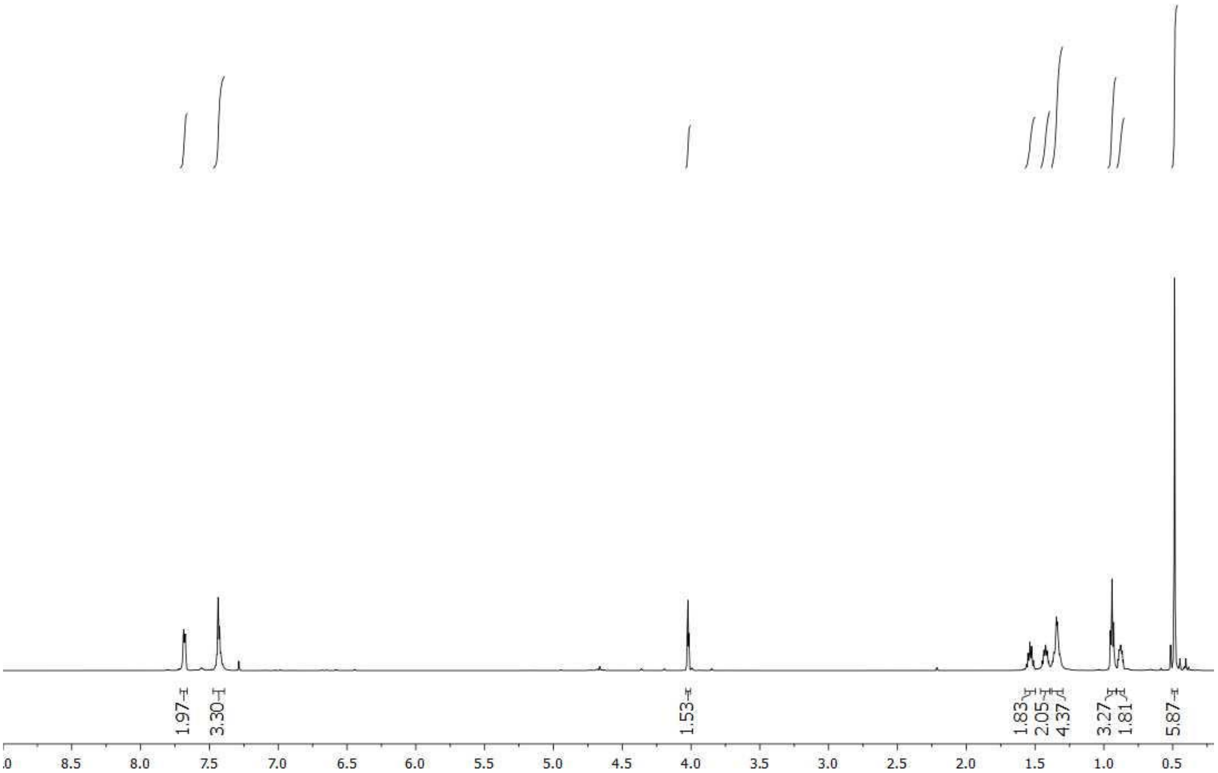

^{13}C NMR

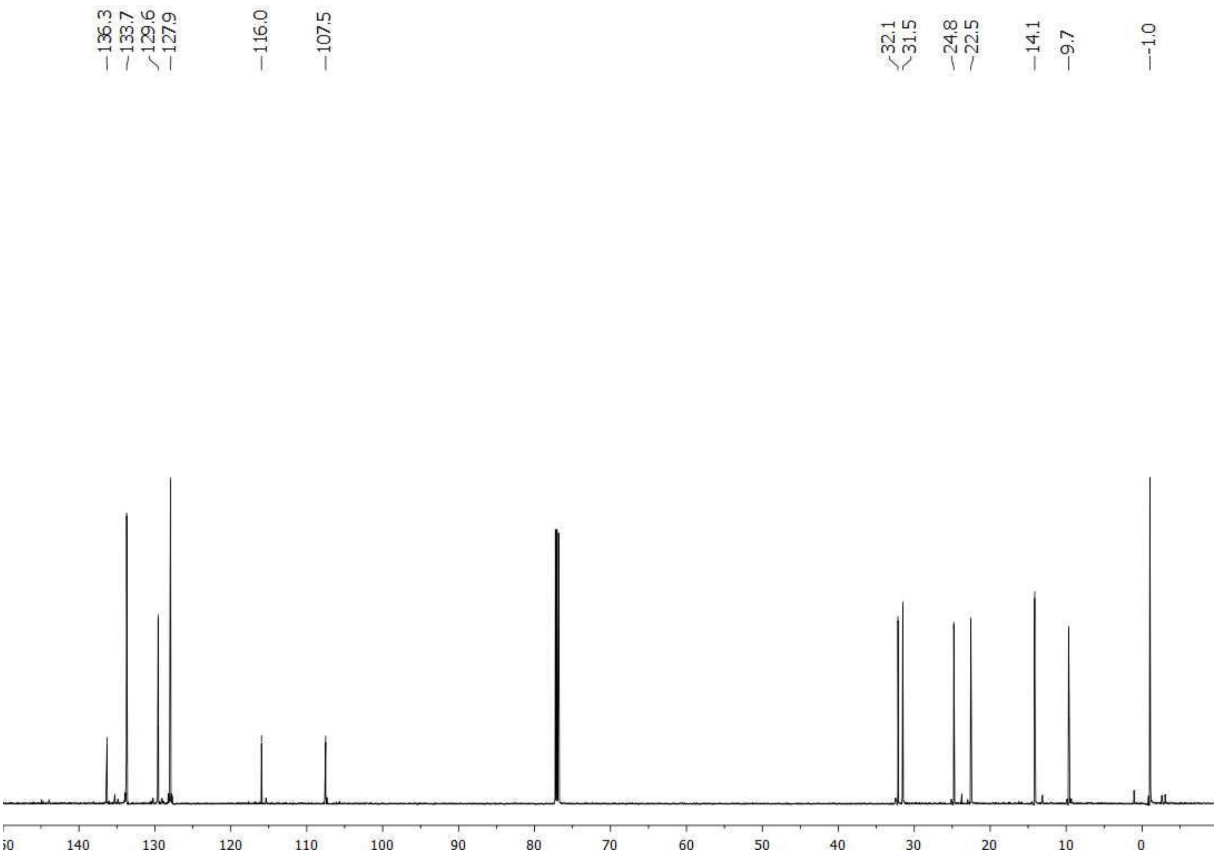

^{29}Si NMR

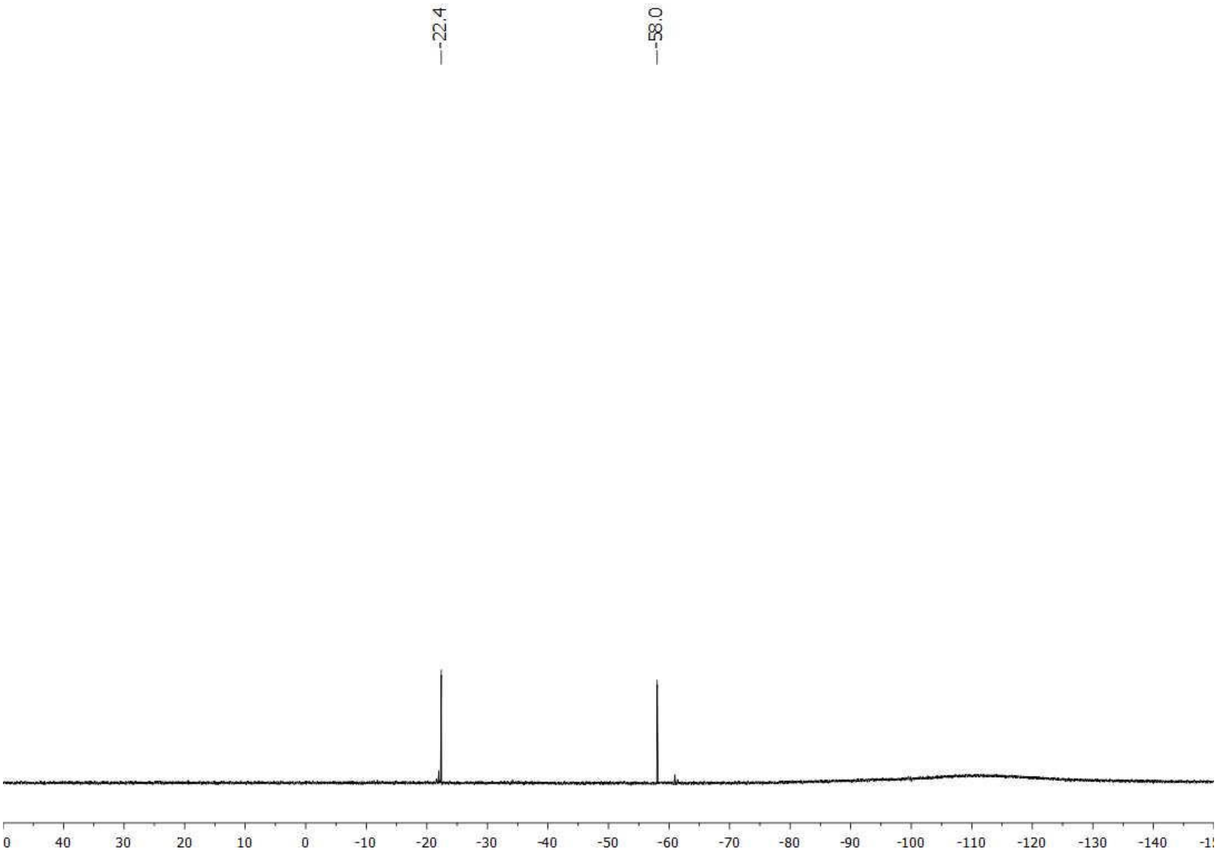

((Hexylsilyl)ethynyl)(methyl)(phenyl)(vinyl)silane (3bj)

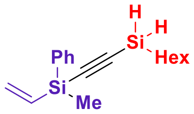

^{1}H NMR

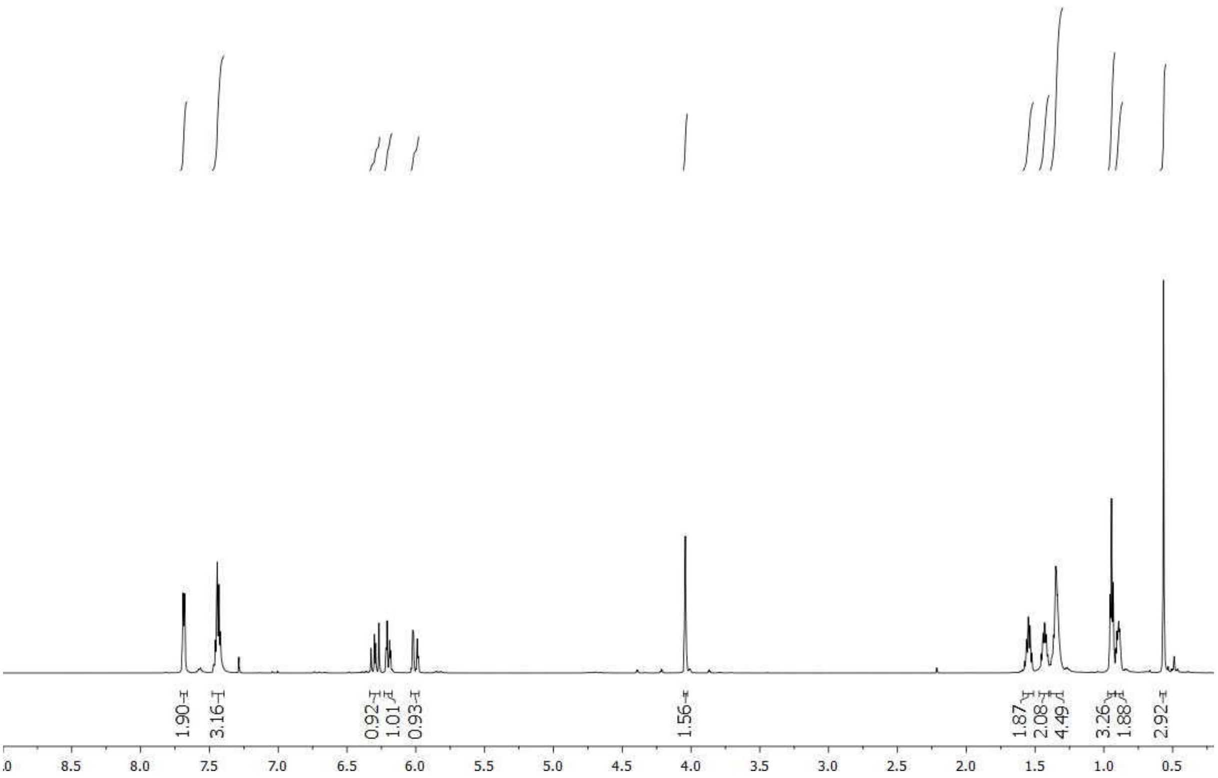

^{13}C NMR

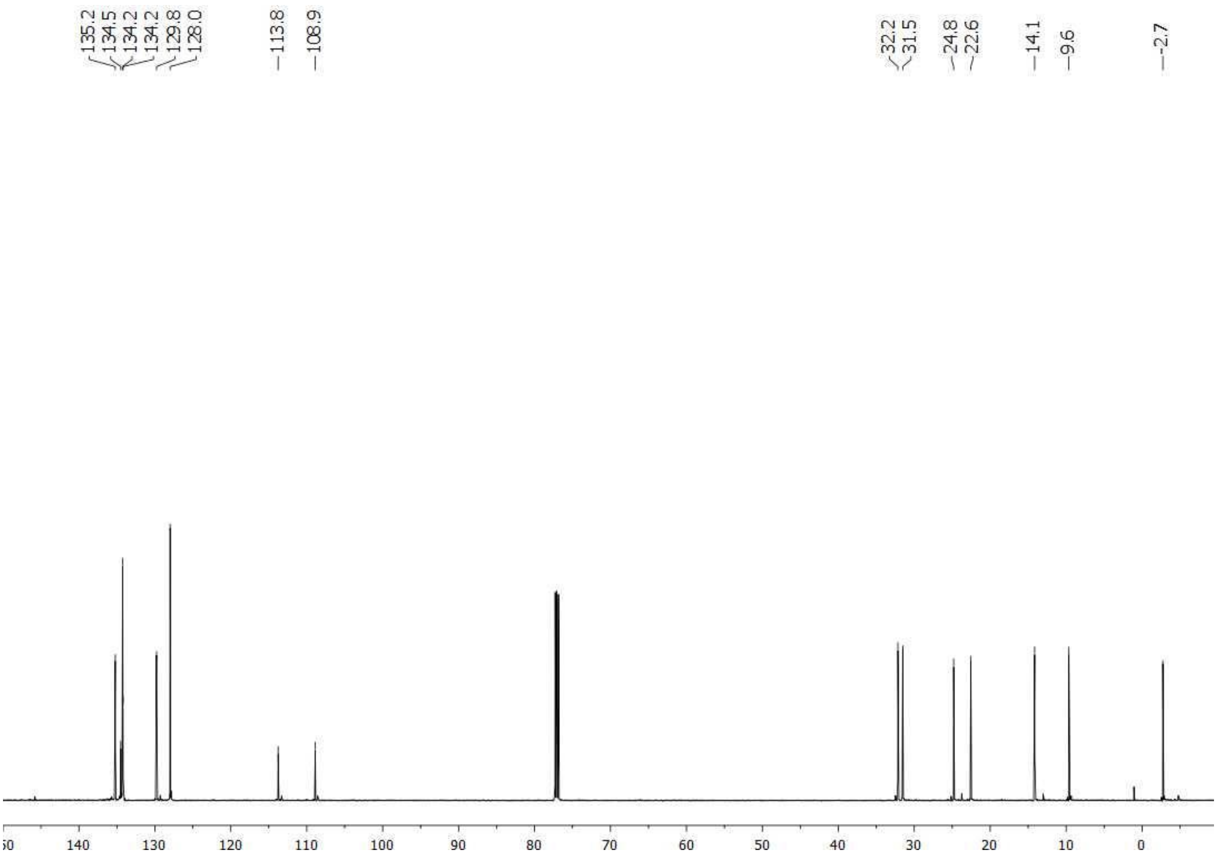

^{29}Si NMR

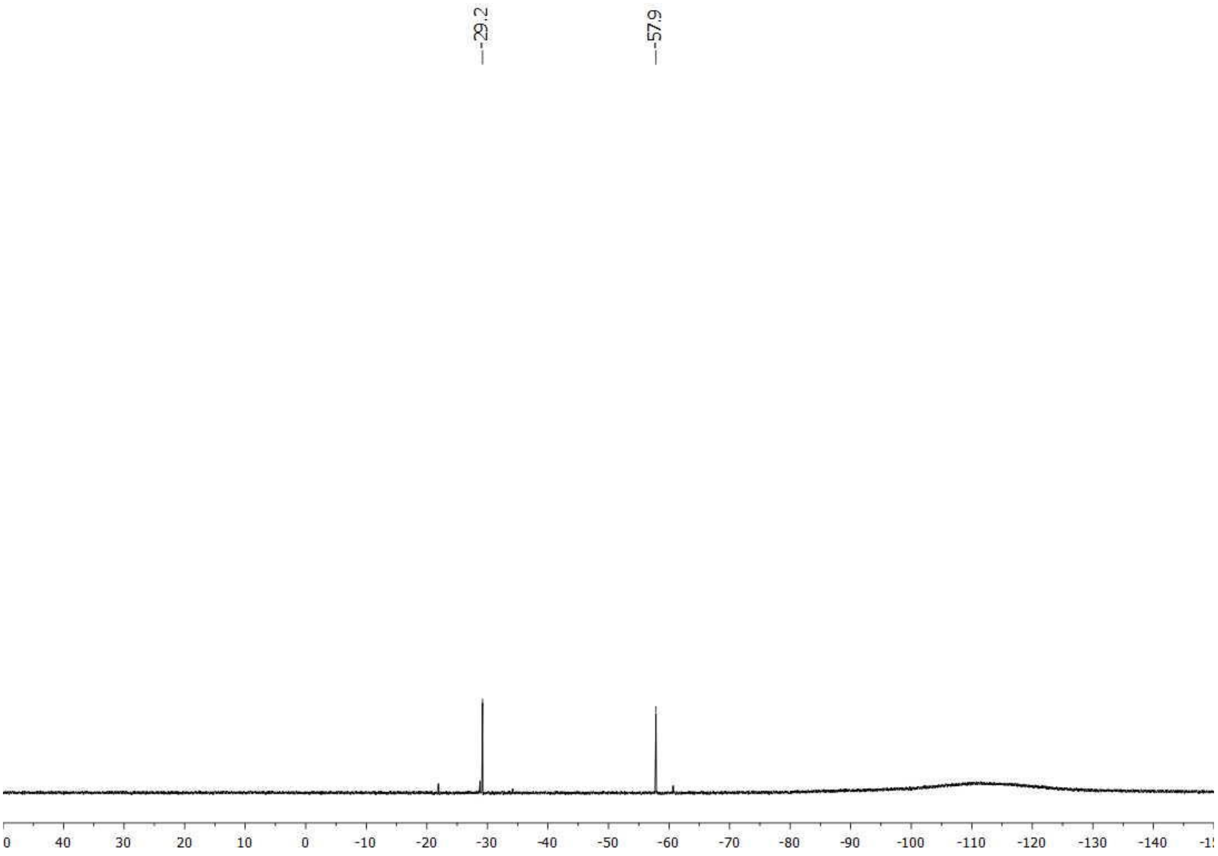

1,2-Bis(((hexylsilyl)ethynyl)dimethylsilyl)ethane (3bk)

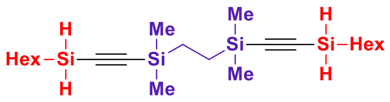

^{1}H NMR

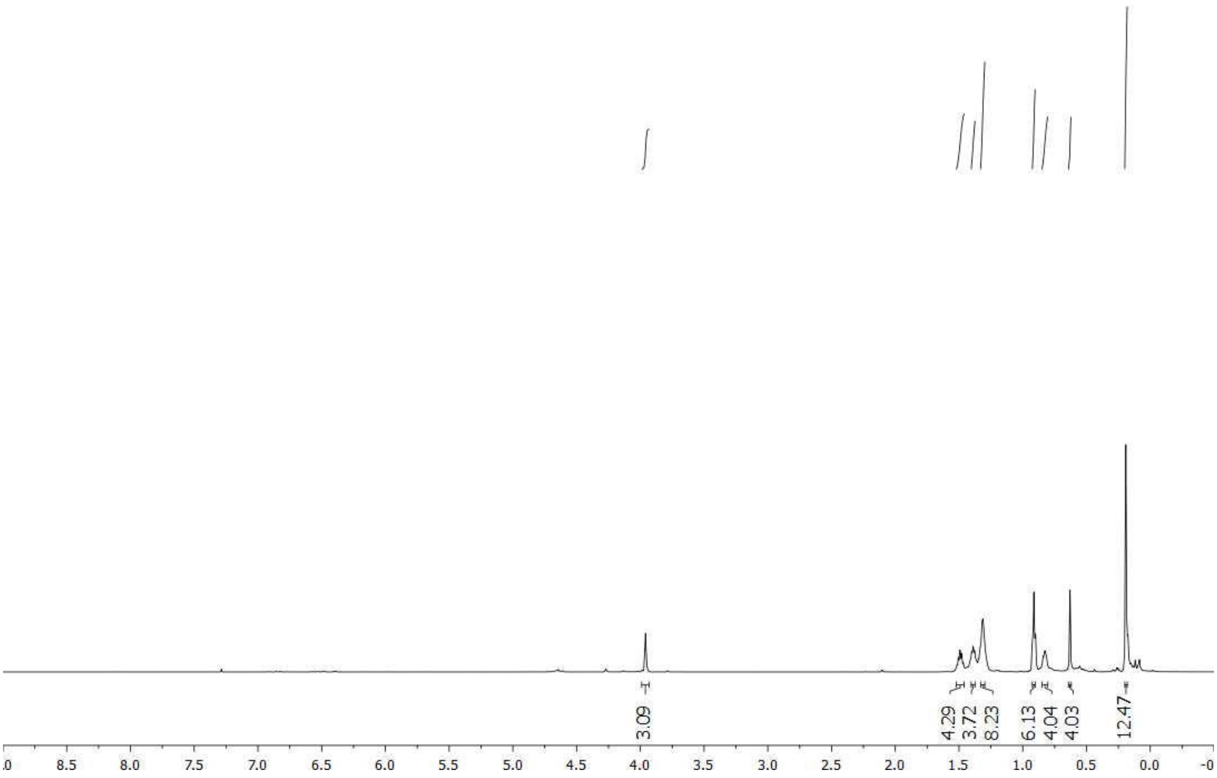

^{13}C NMR

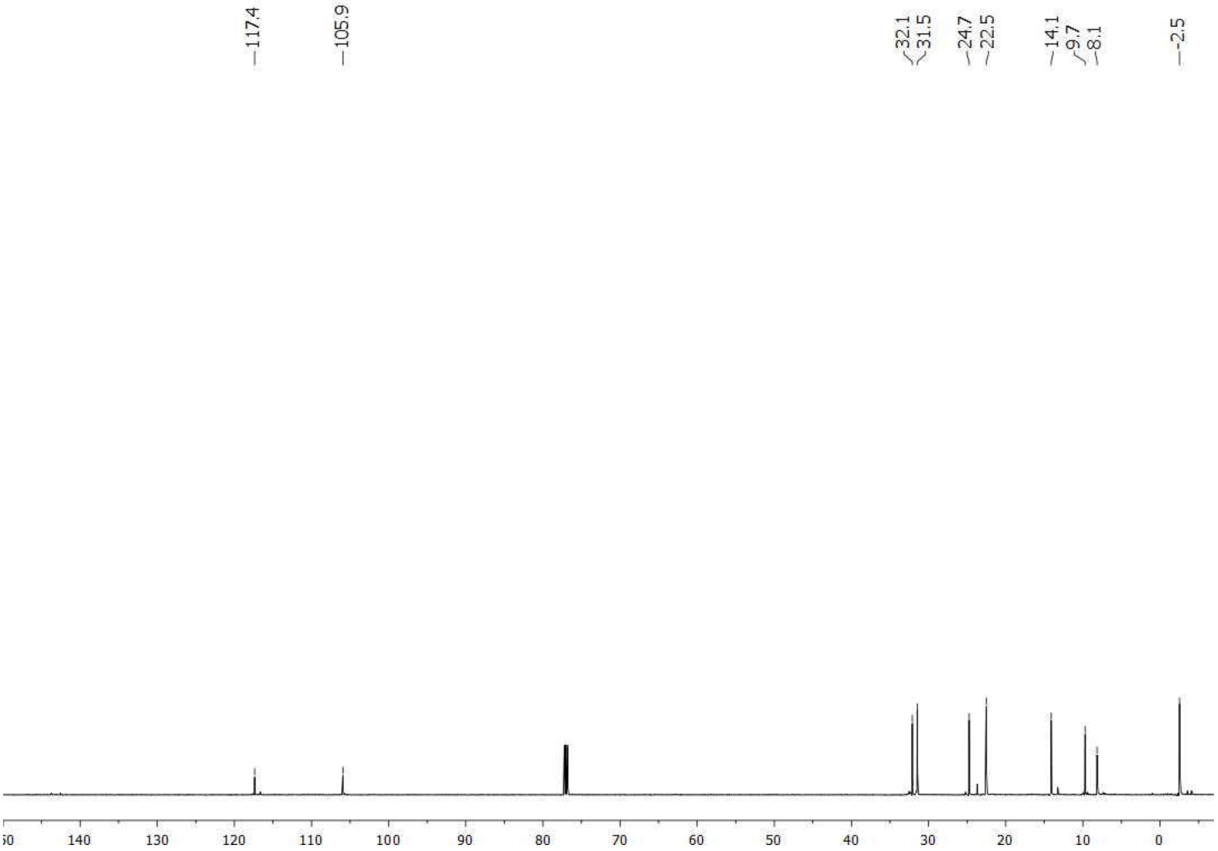

^{29}Si NMR

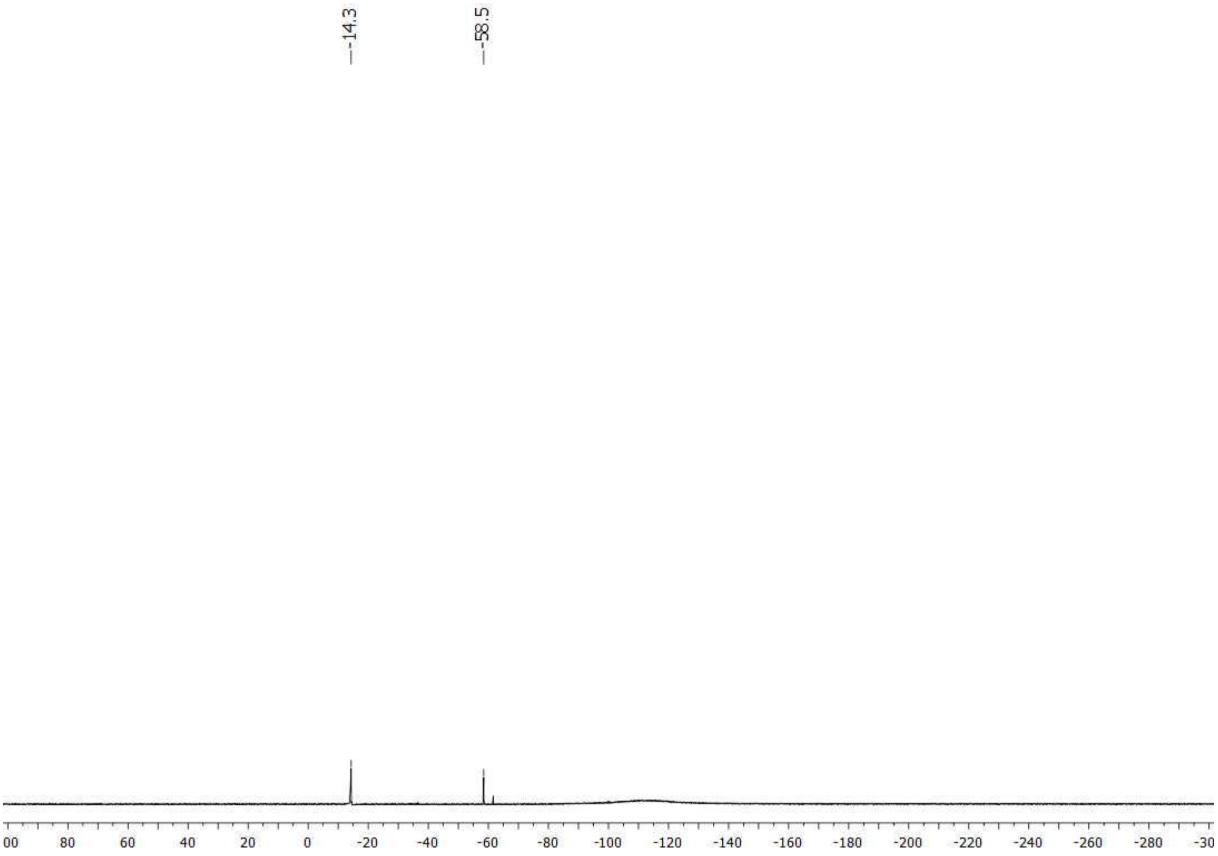

Triisopropyl((p-tolylsilyl)ethynyl)silane (3cc)

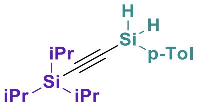

^{1}H NMR

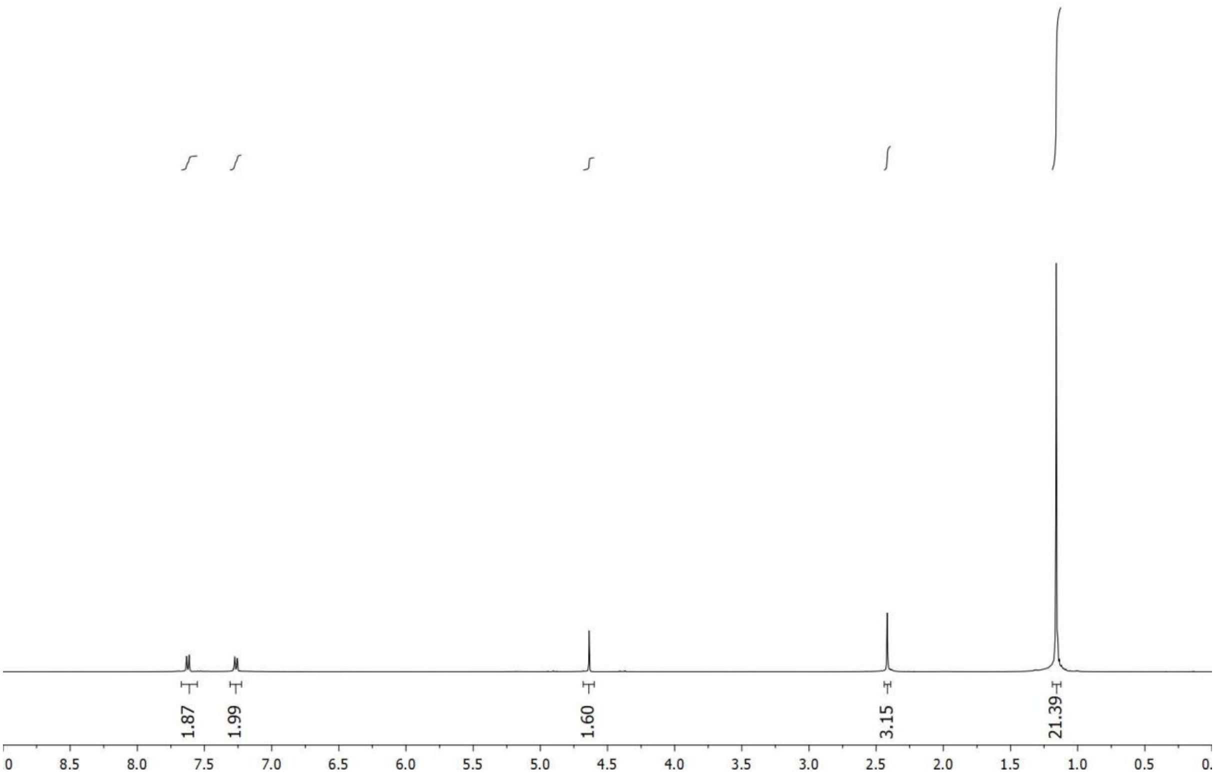

^{13}C NMR

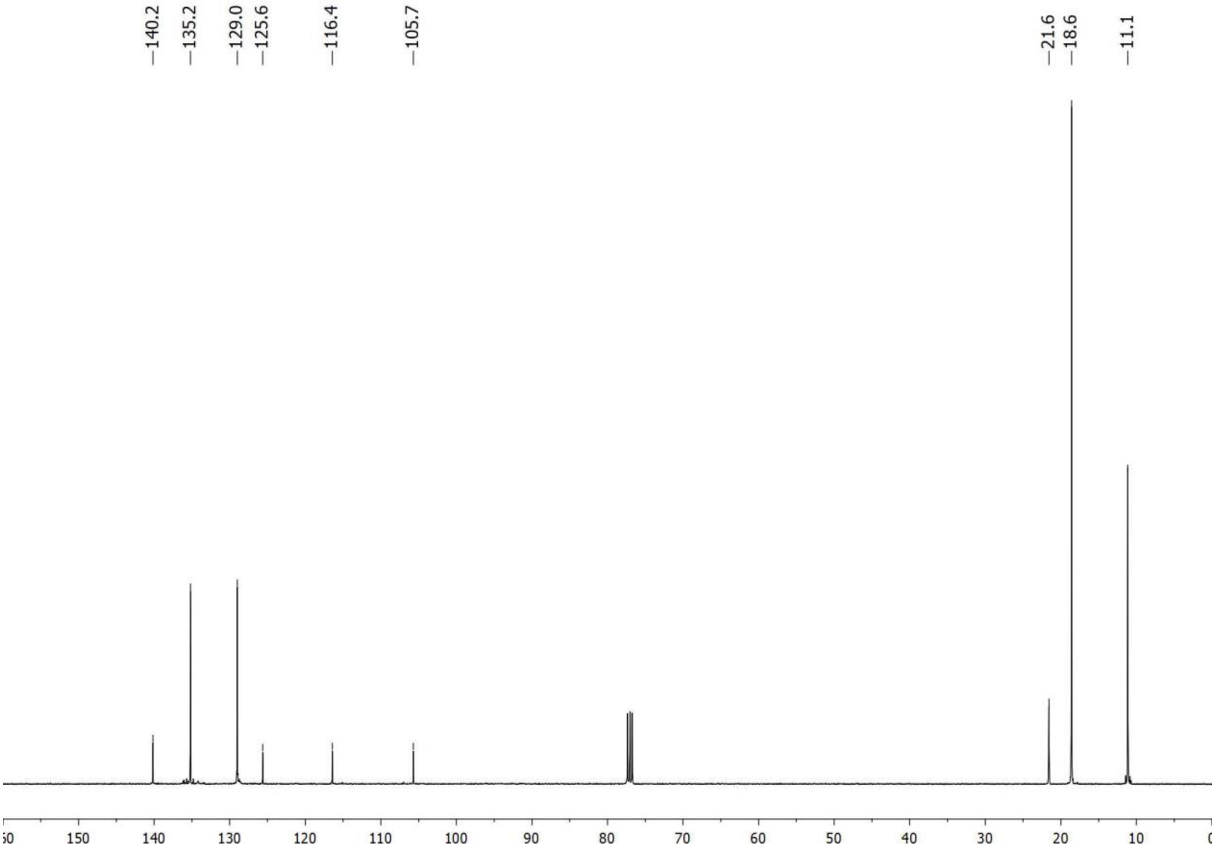

^{29}Si NMR

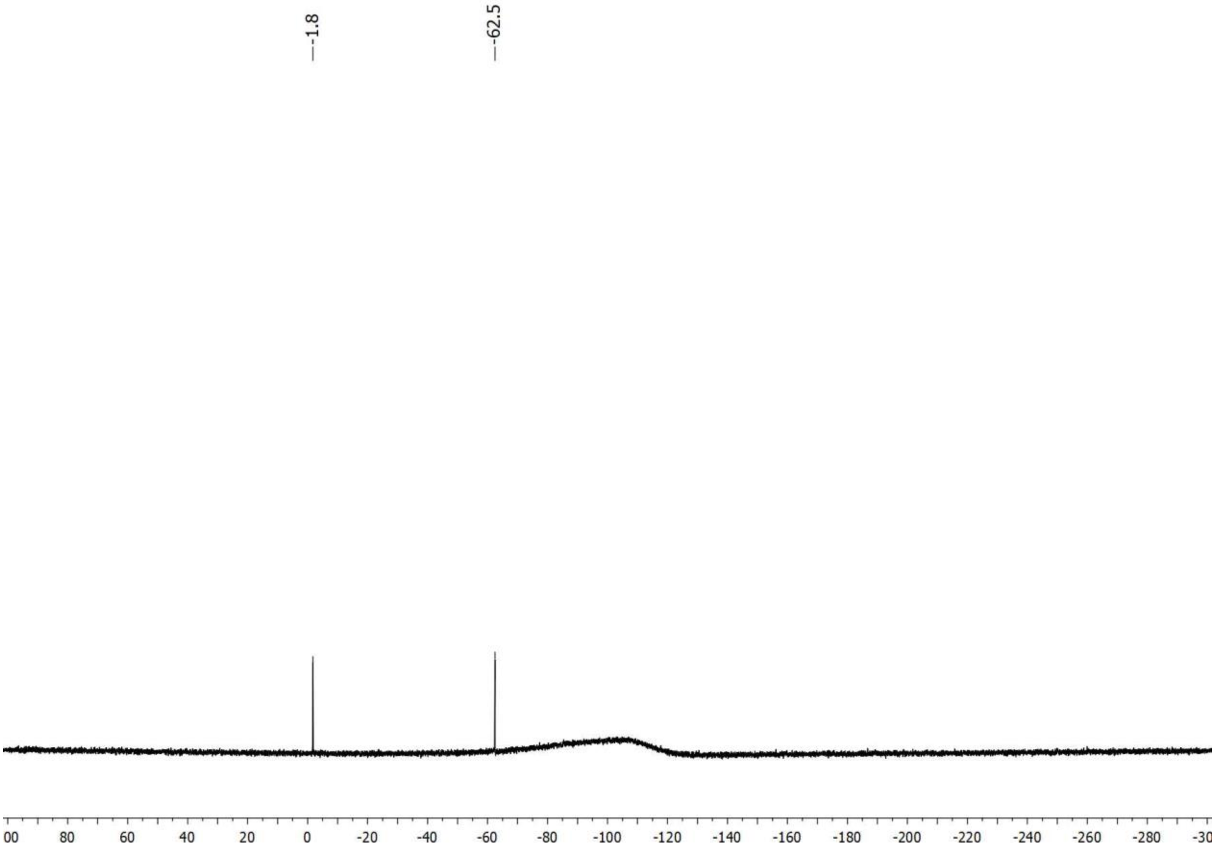

[Bis[2-(trimethylsilyl)ethynyl]silyl]benzene (4aa)

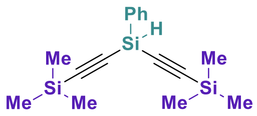

^{1}H NMR

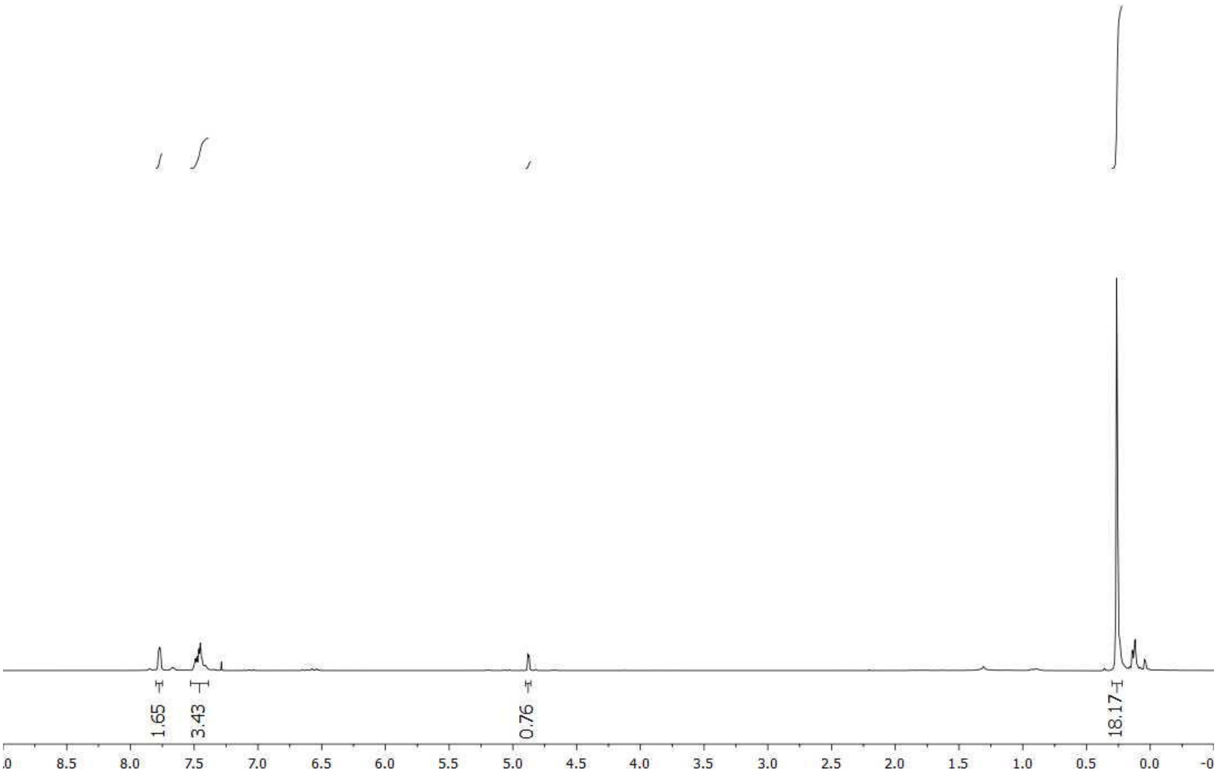

^{13}C NMR

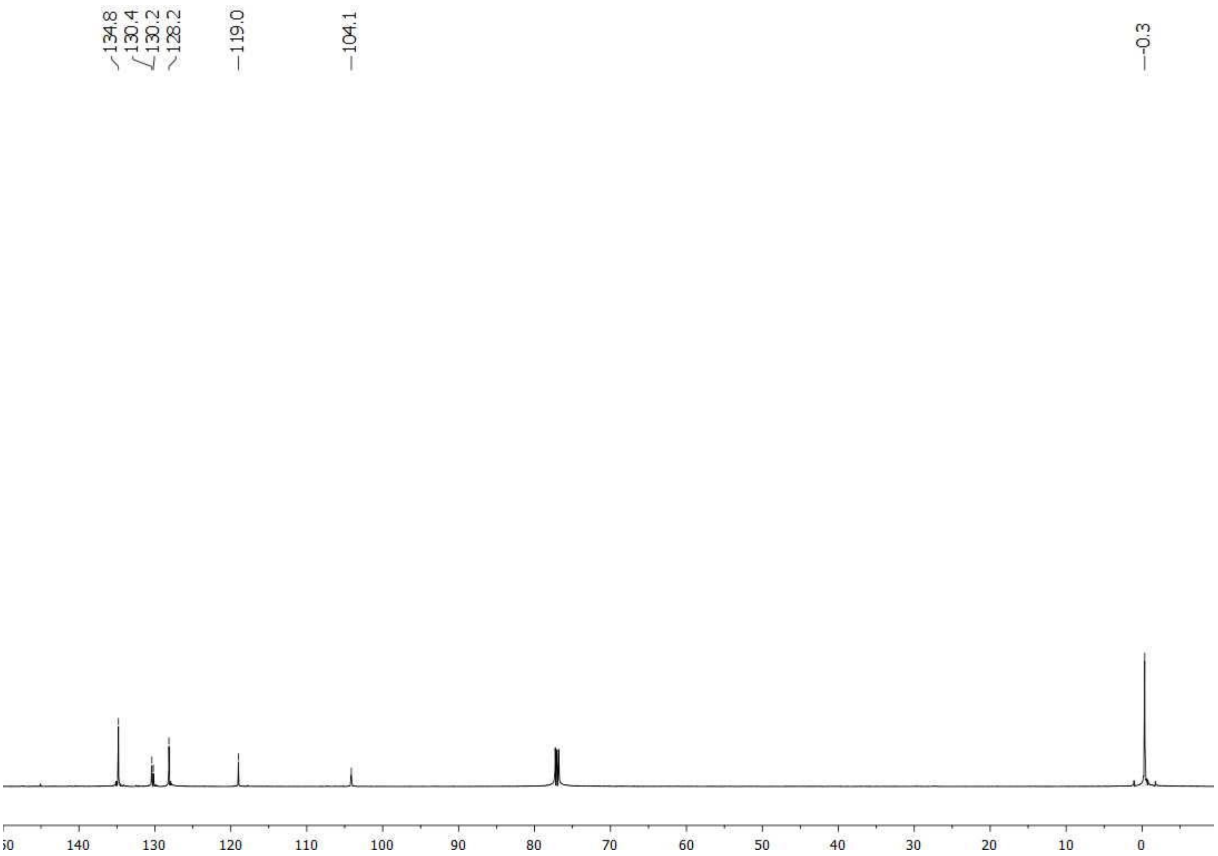

^{29}Si NMR

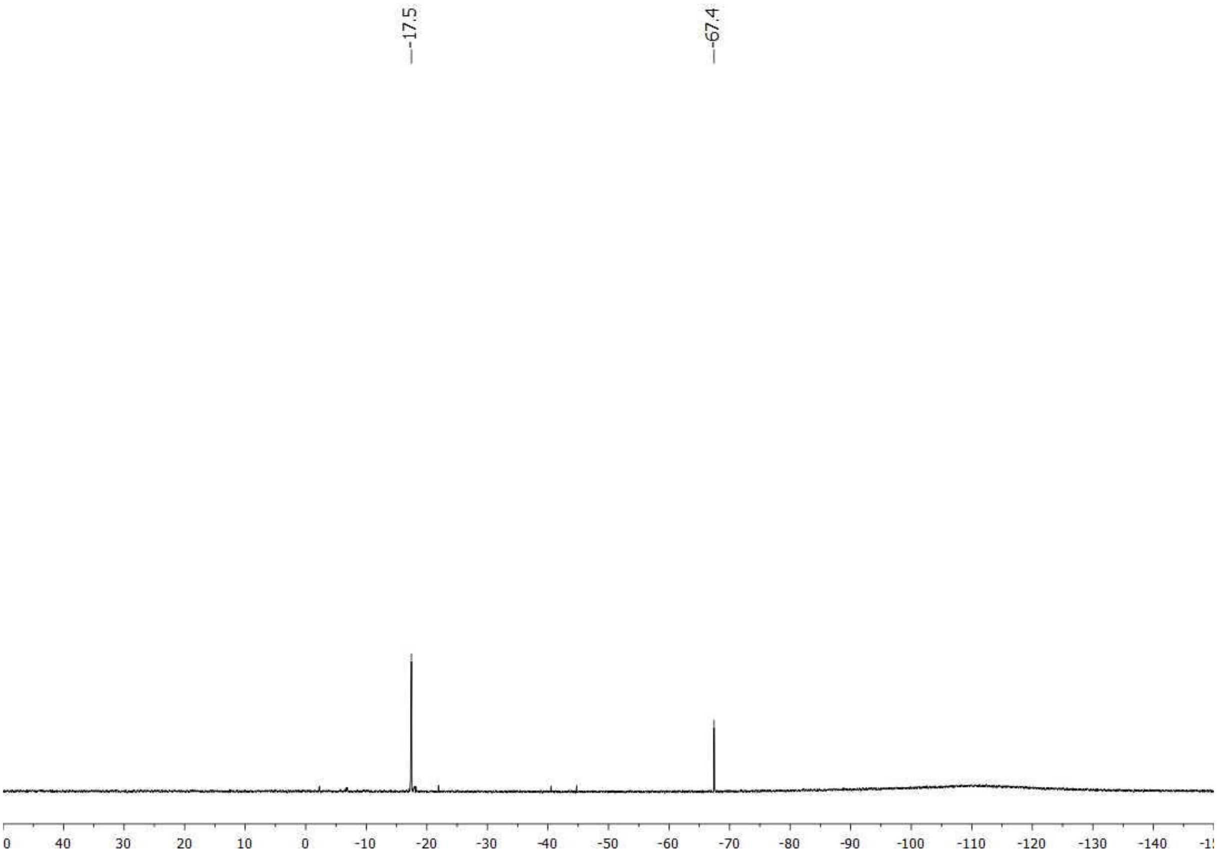

[Bis[2-(triethylsilyl)ethynyl]silyl]benzene (4ab)

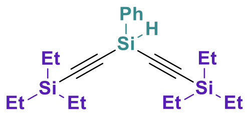

^{1}H NMR

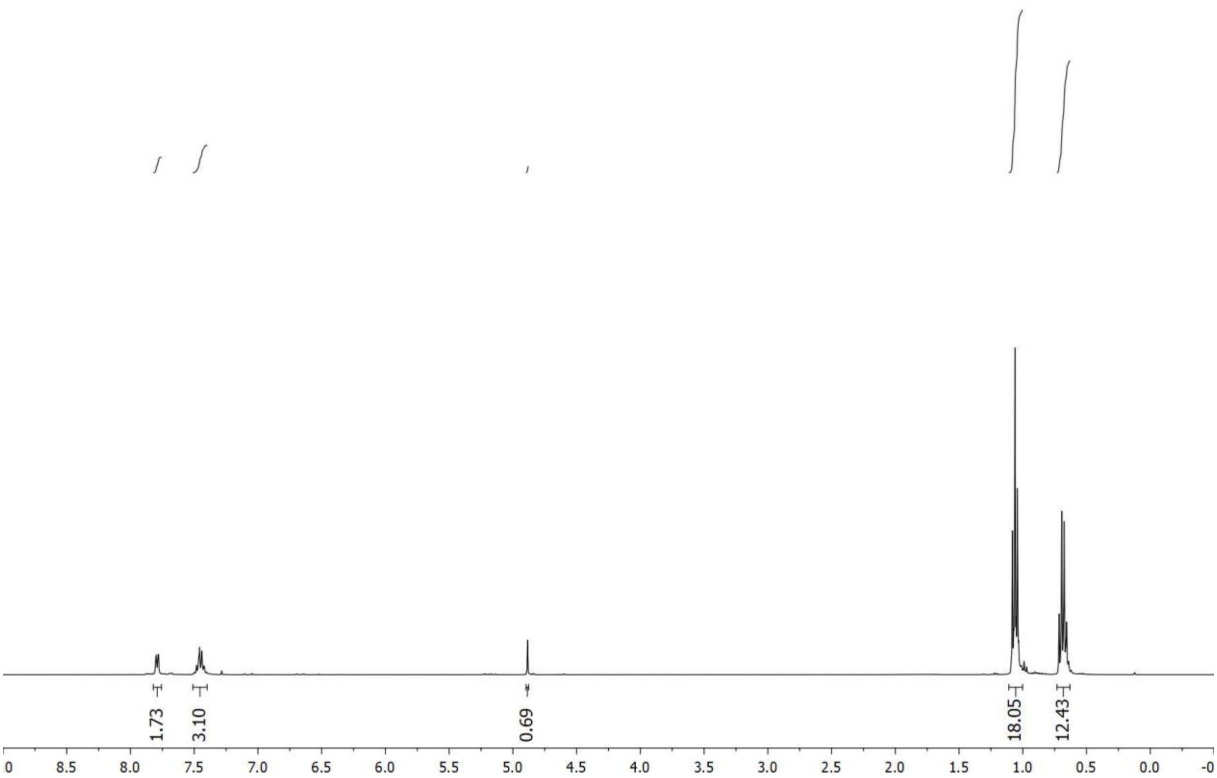

^{13}C NMR

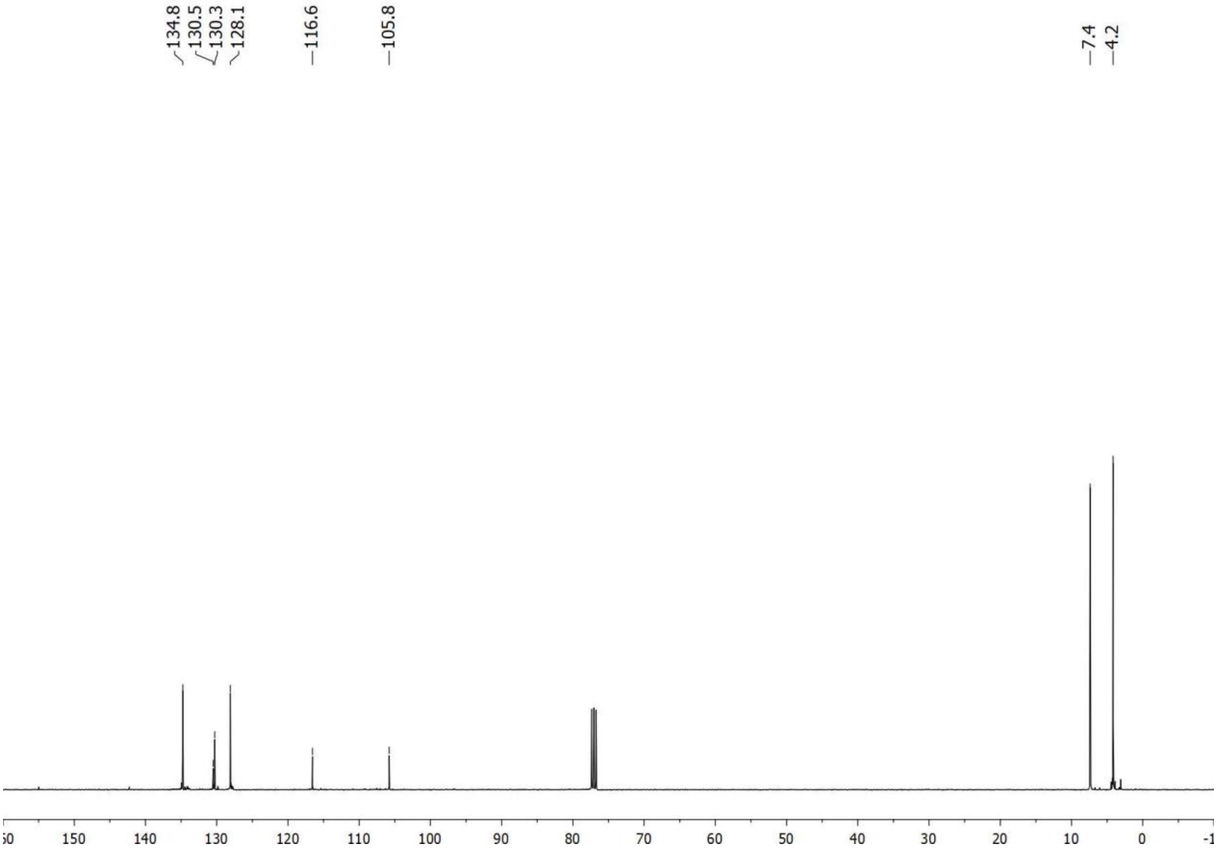

^{29}Si NMR

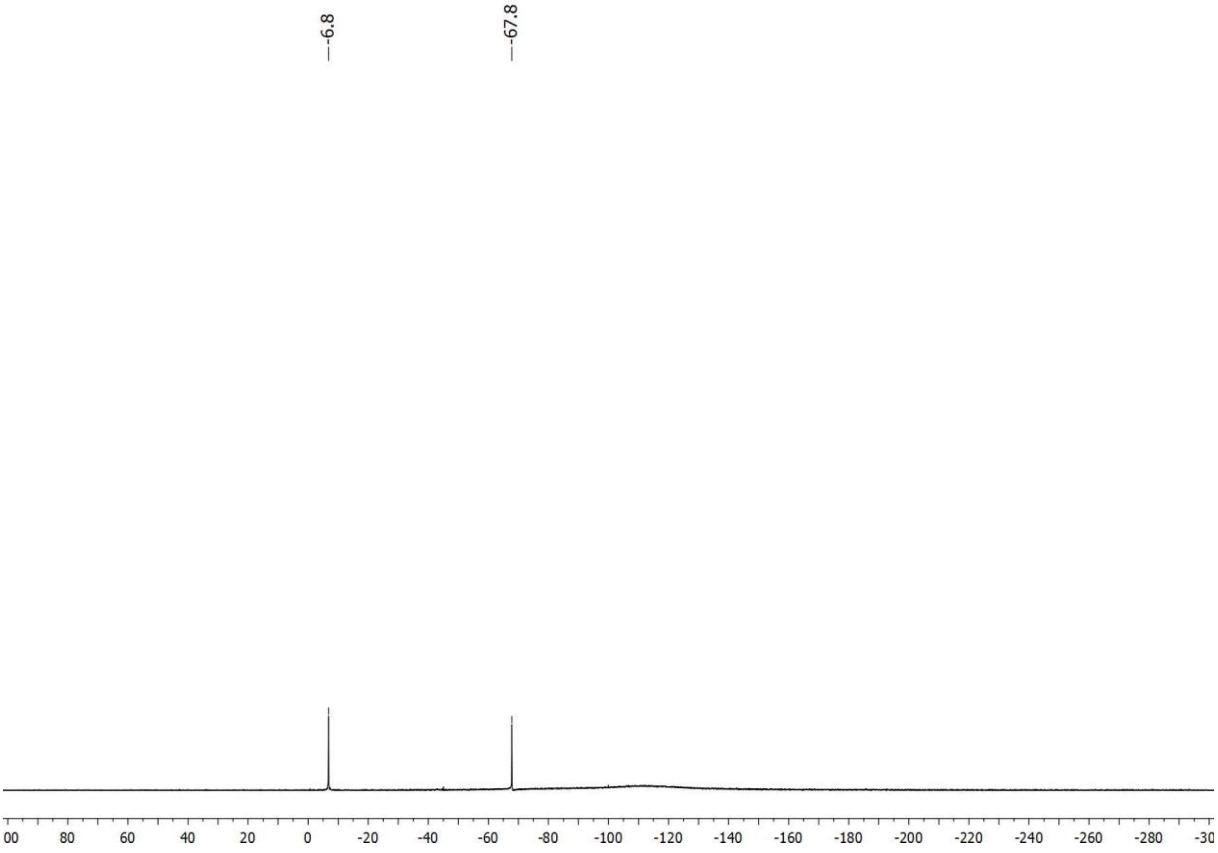

[Bis[2-(tripropylsilyl)ethynyl]silyl]benzene (4ae)

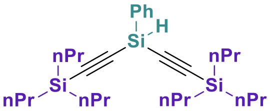

^{1}H NMR

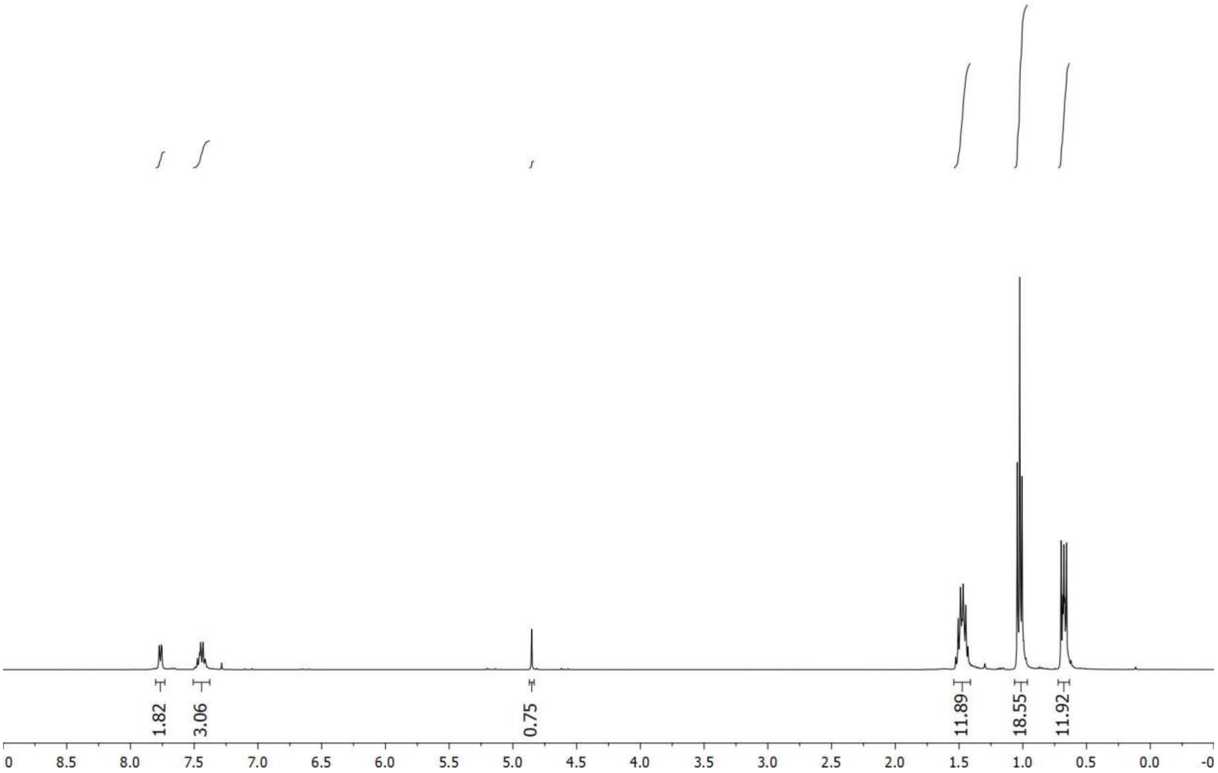

^{13}C NMR

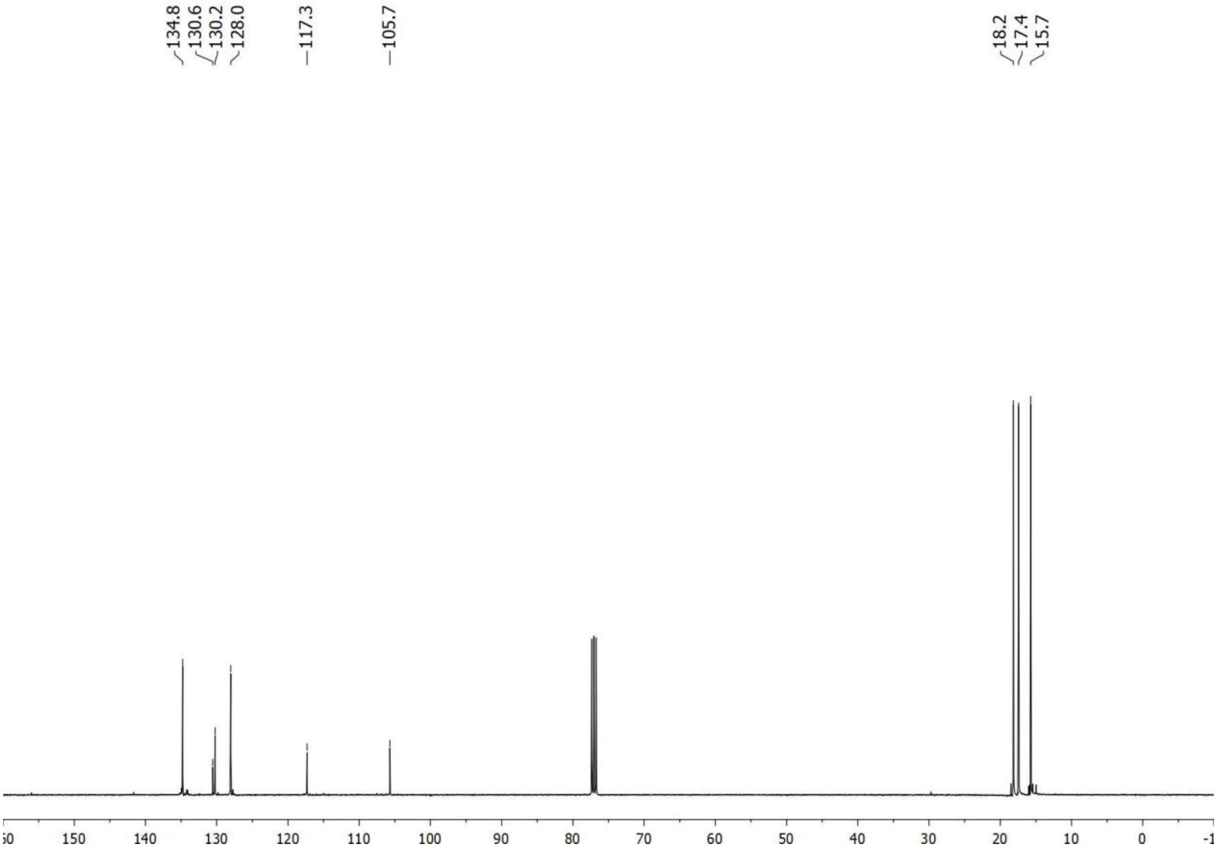

^{29}Si NMR

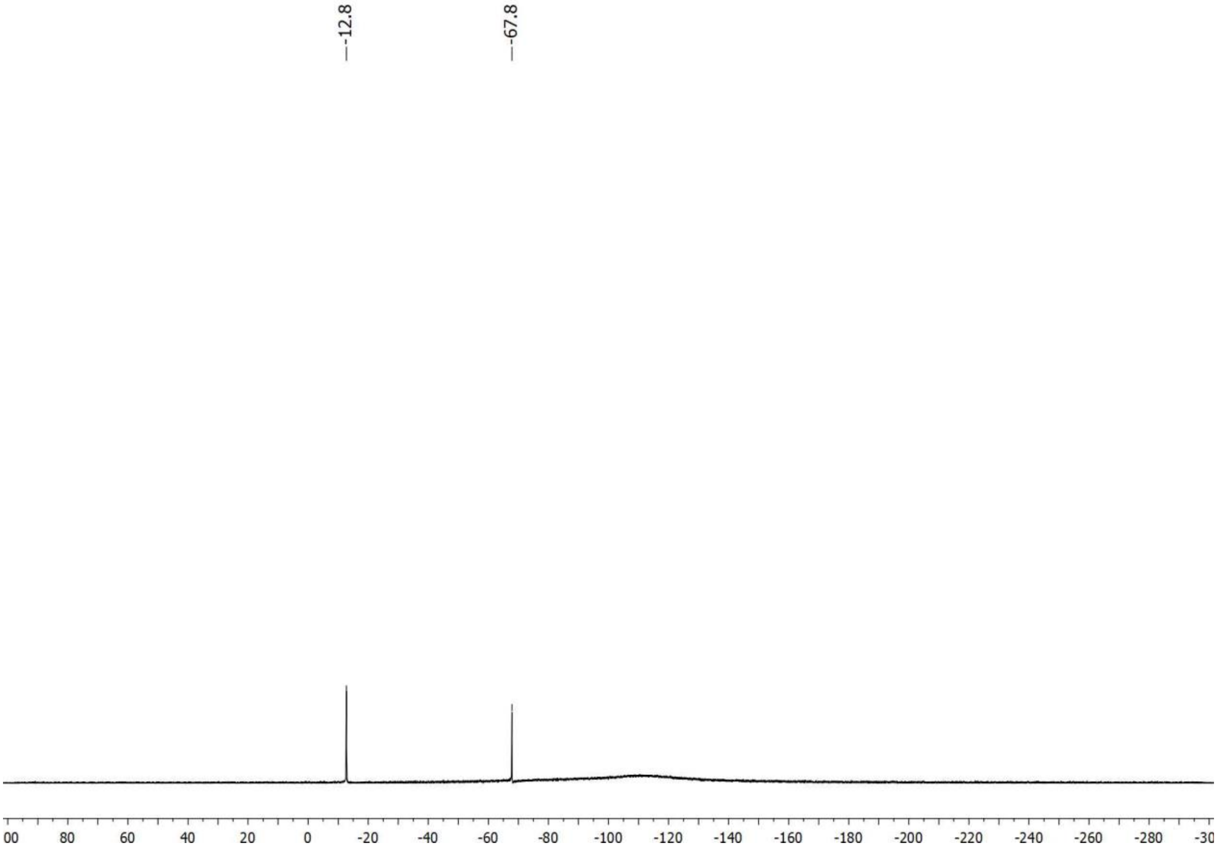

[Bis[2-(tert-butyl(dimethyl)silyl)ethynyl]silyl]benzene (4ag)

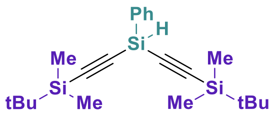

^{1}H NMR

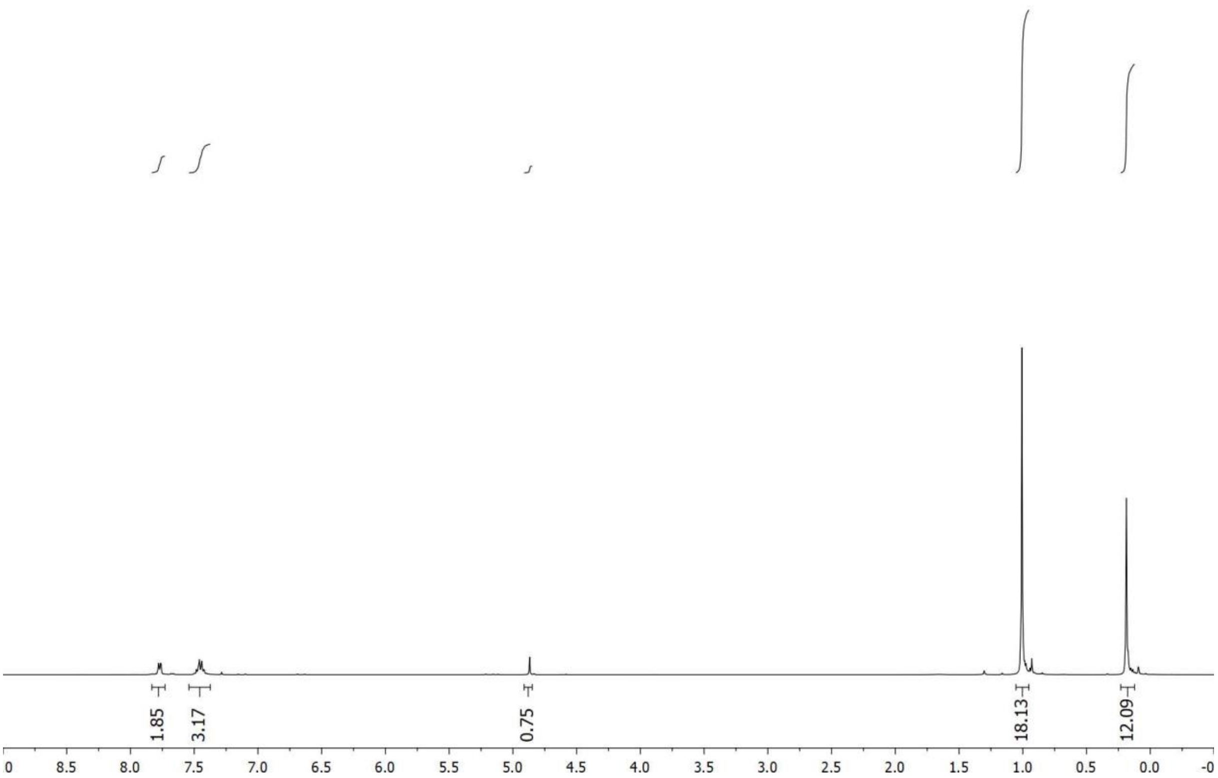

^{13}C NMR

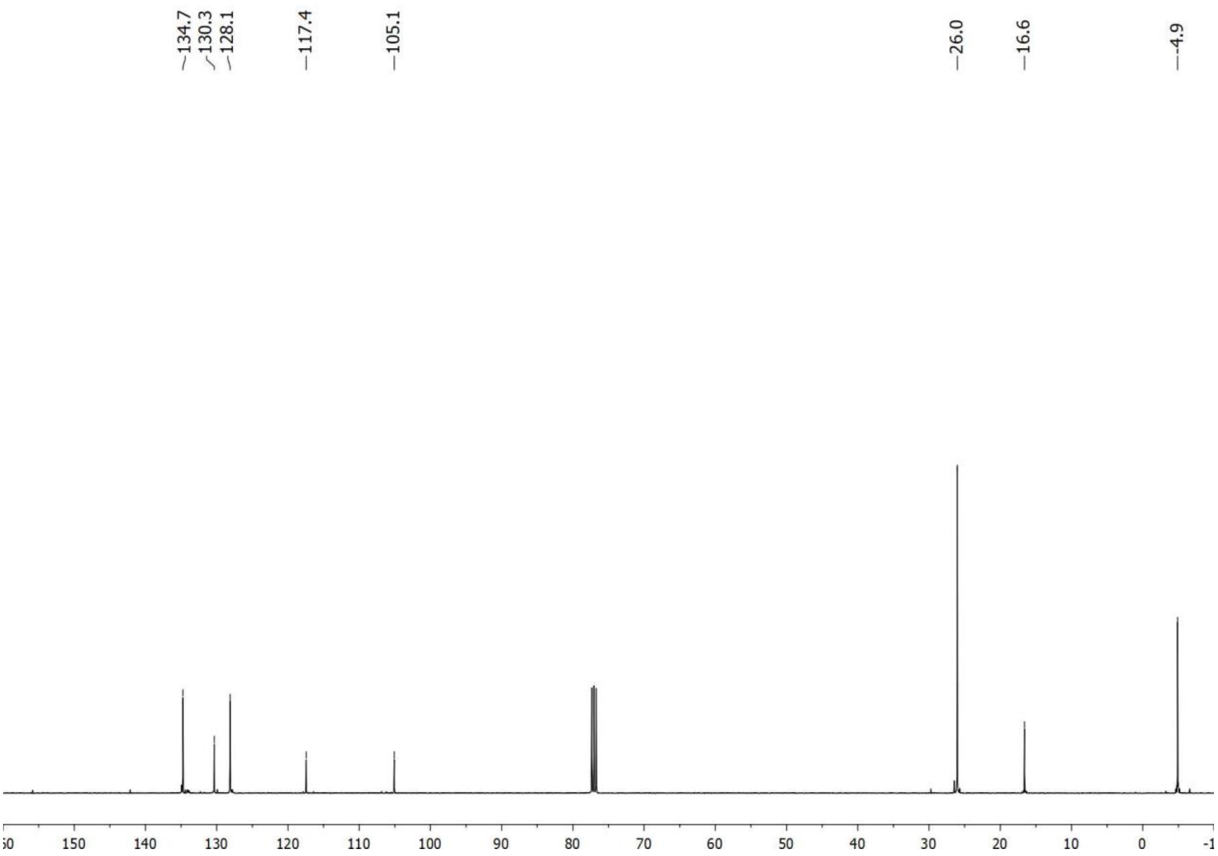

^{29}Si NMR

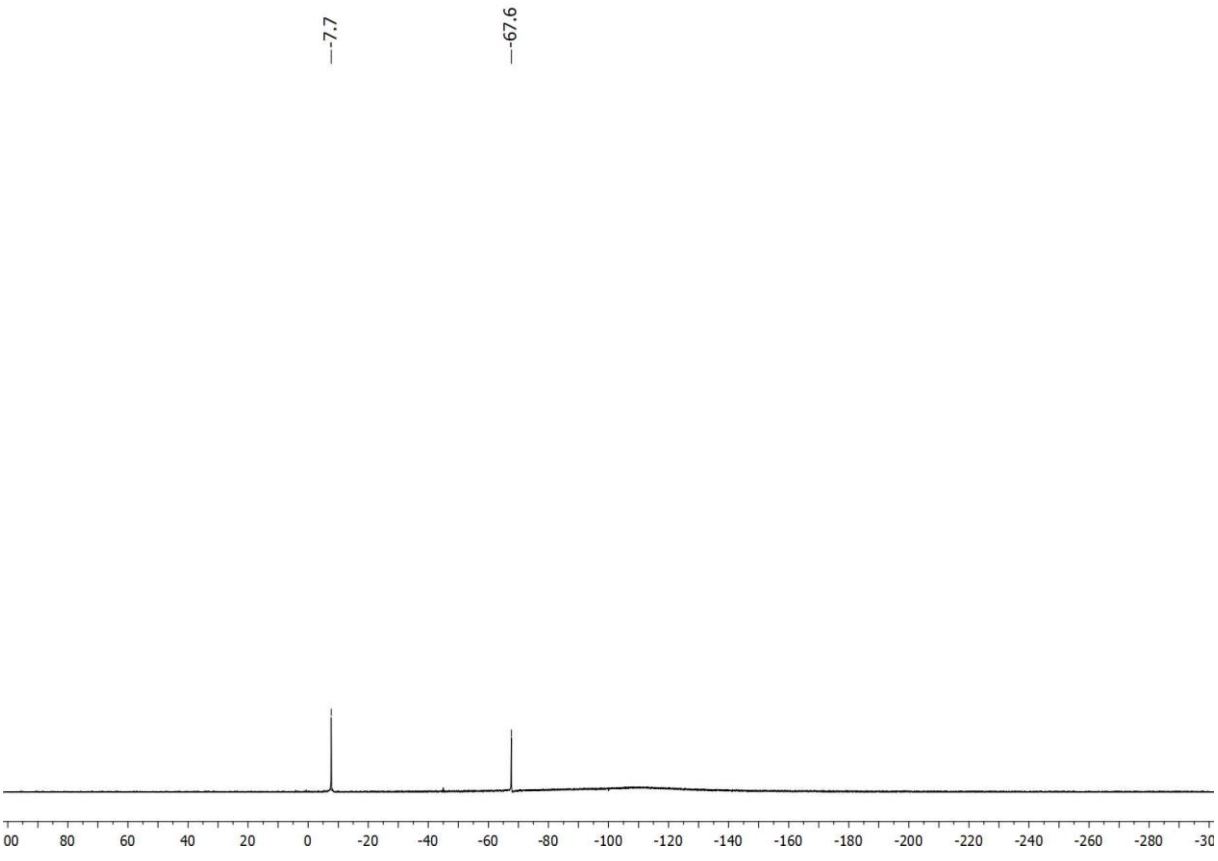

[Bis[2-(dimethyl(phenyl)silyl)ethynyl]silyl]benzene (4ai)

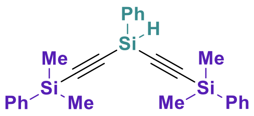

^{1}H NMR

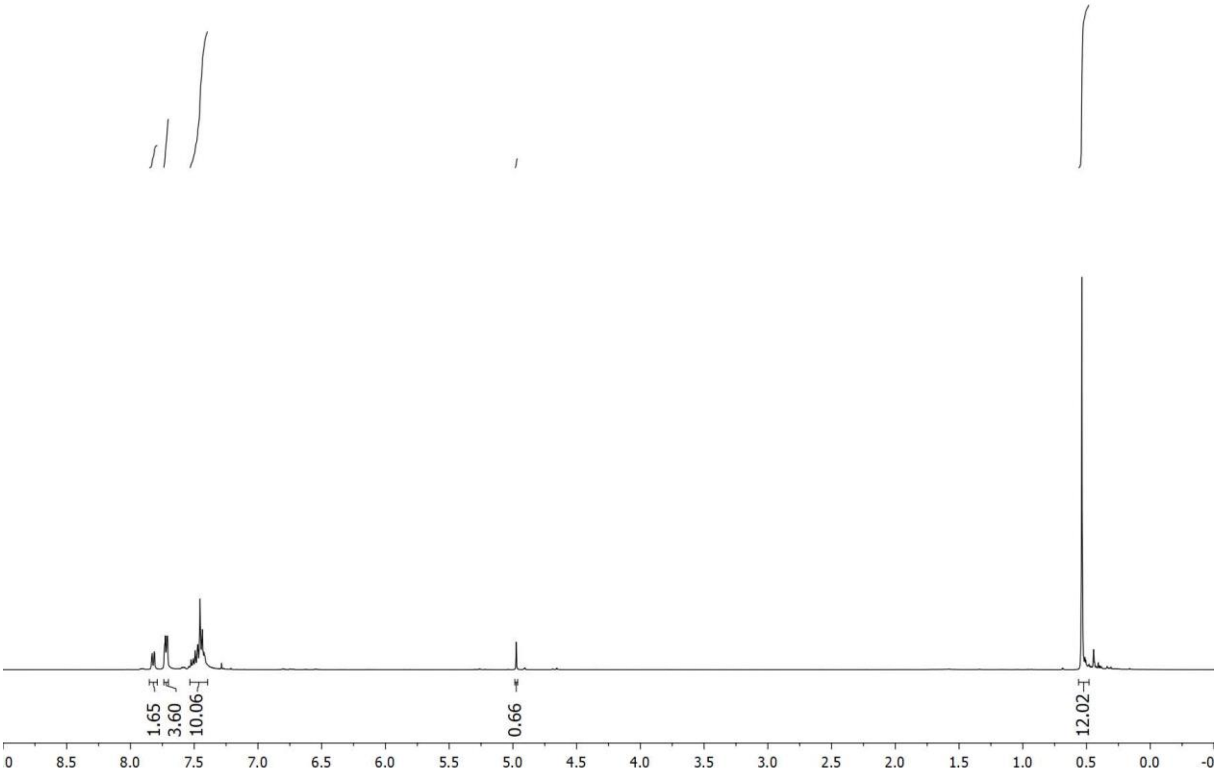

^{13}C NMR

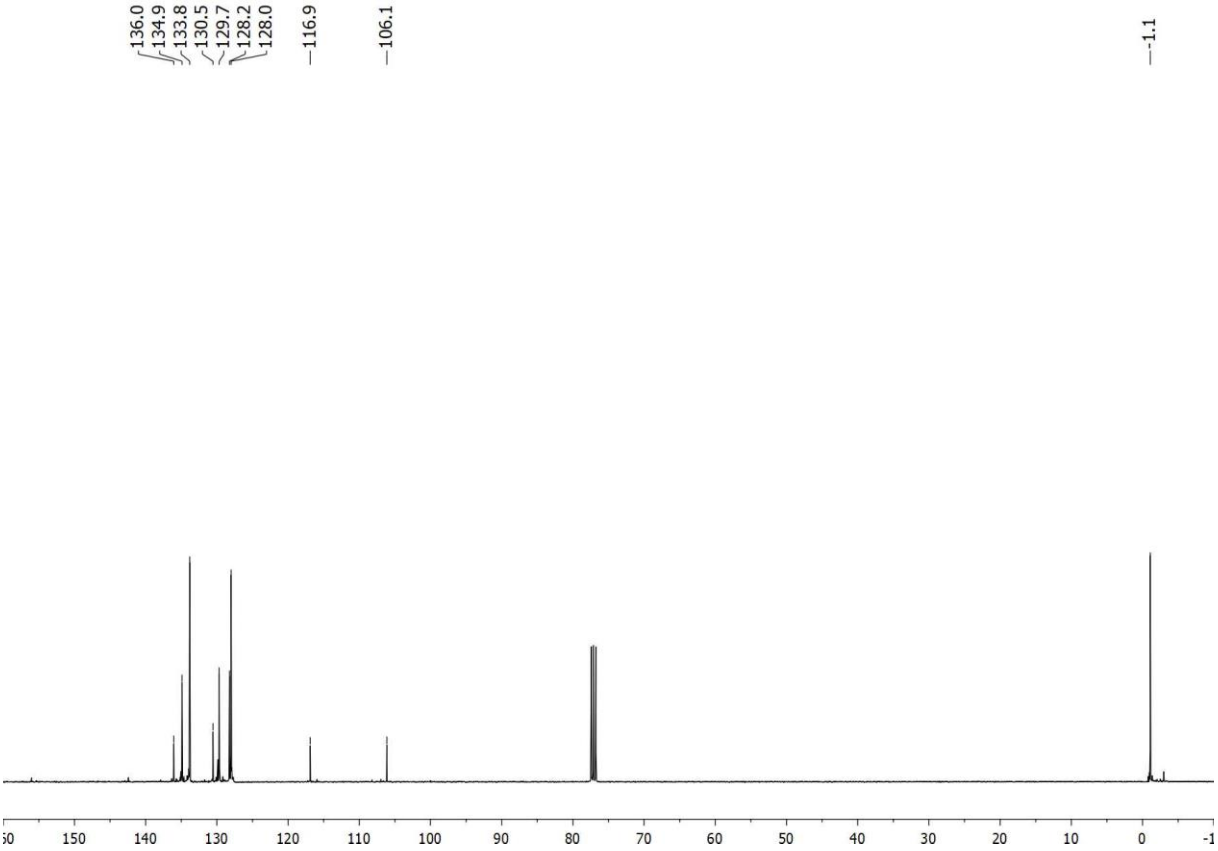

^{29}Si NMR

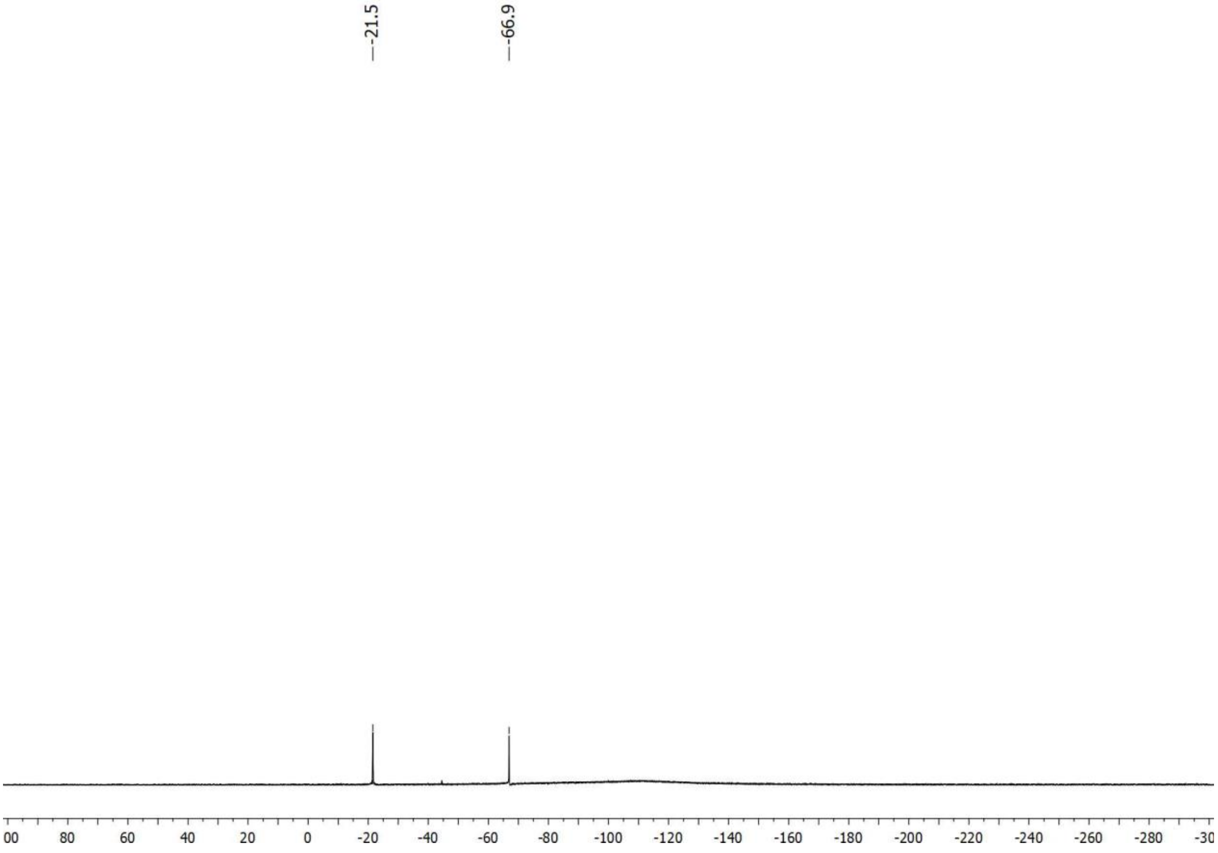

[(Diphenylsilyl)ethynyl]triisopropylsilane (3dc)

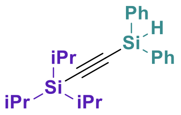

^{1}H NMR

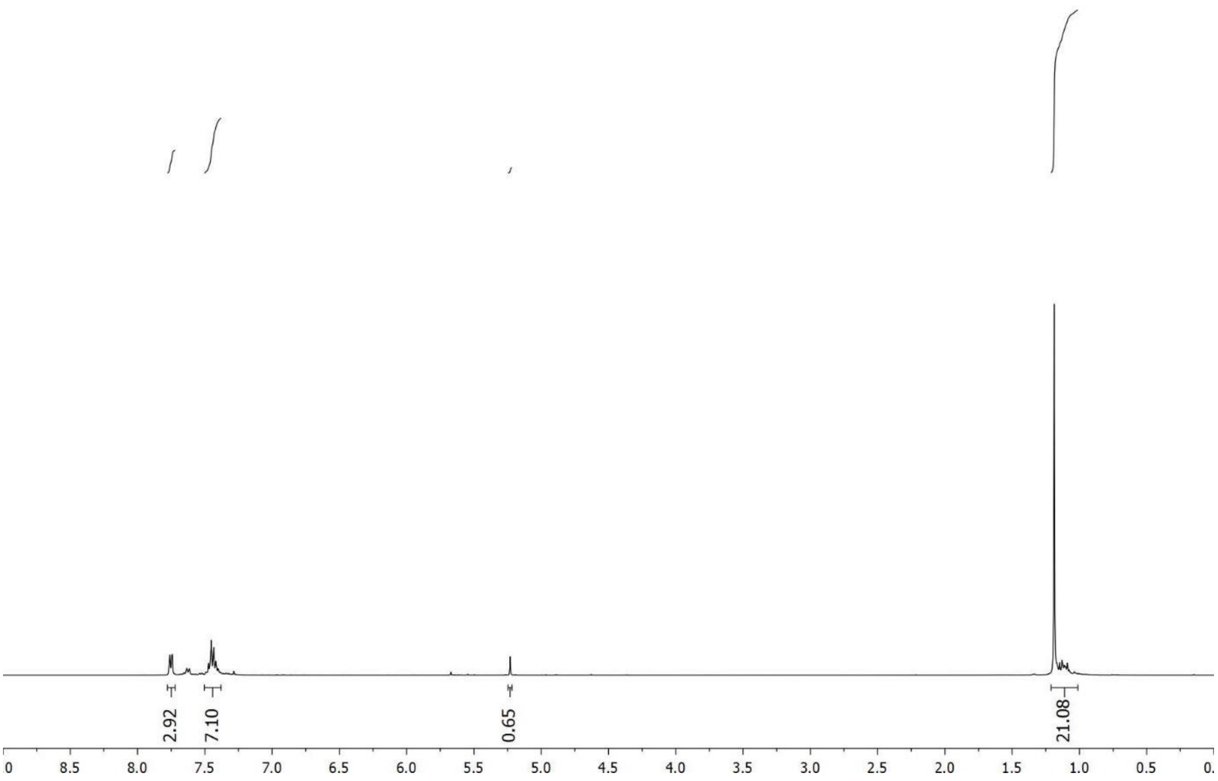

^{13}C NMR

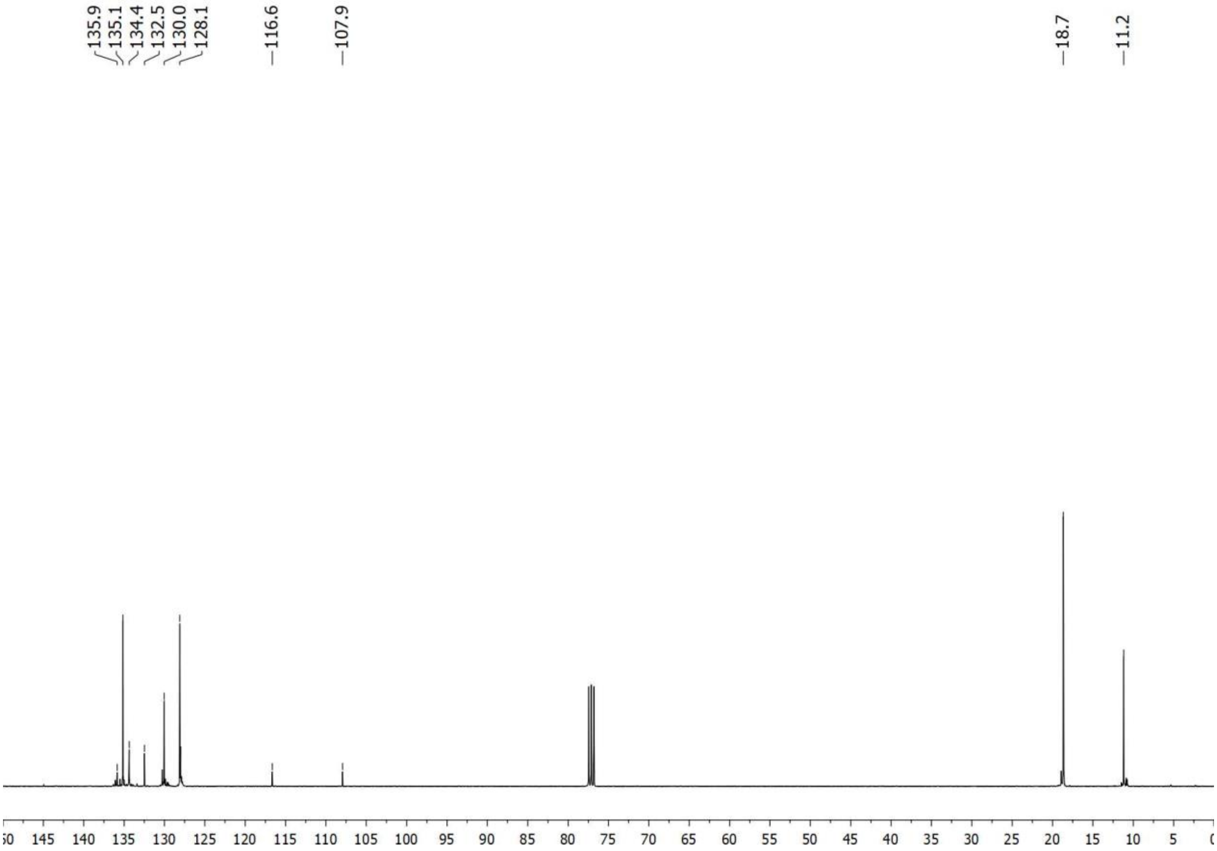

^{29}Si NMR

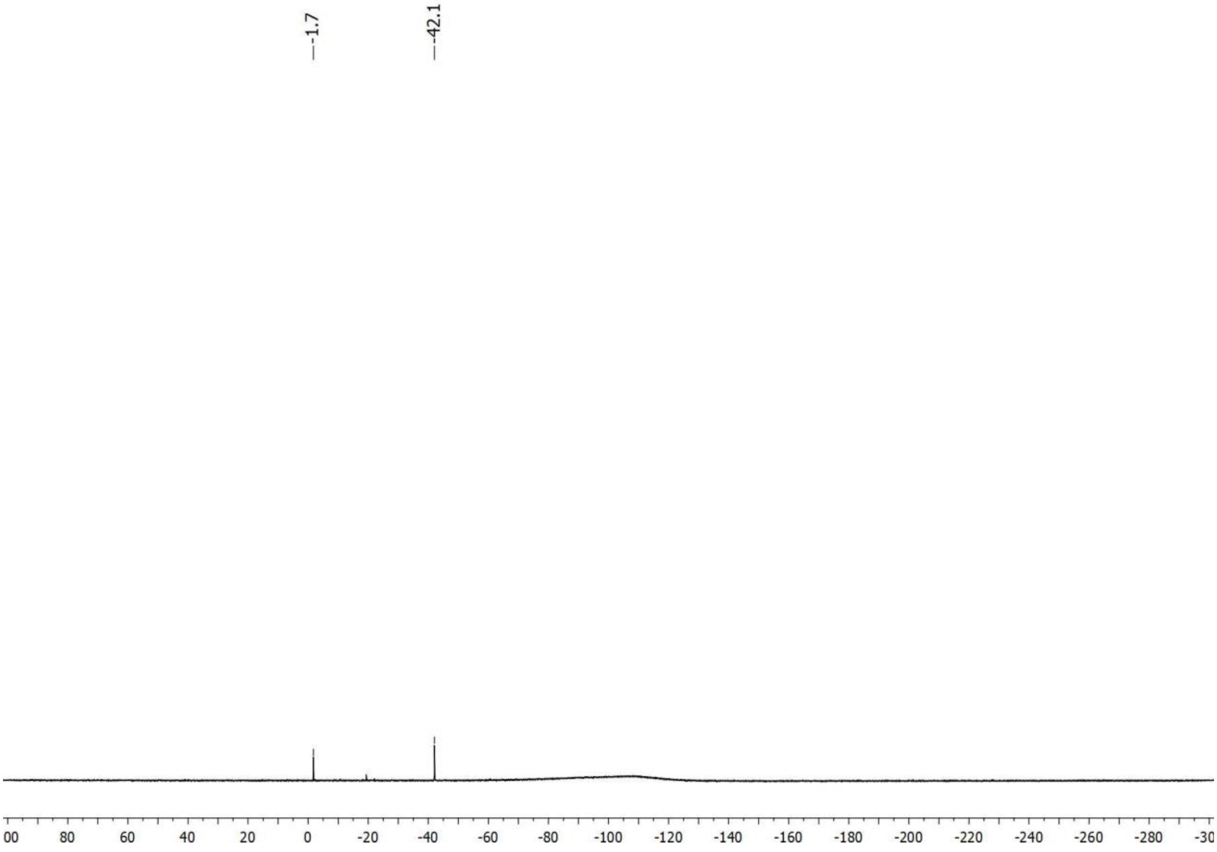

[(Methyl(phenyl)silyl)ethynyl]triisopropylsilane (3ec)

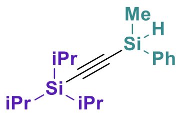

^{1}H NMR

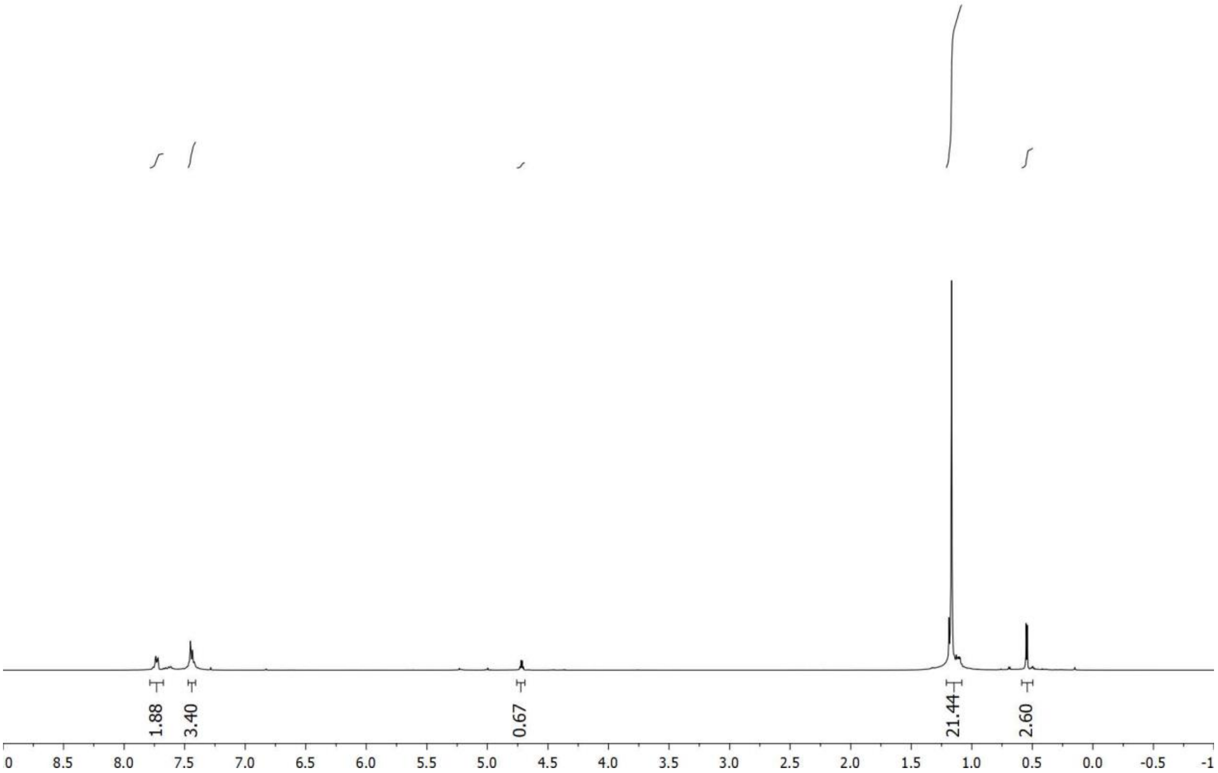

^{13}C NMR

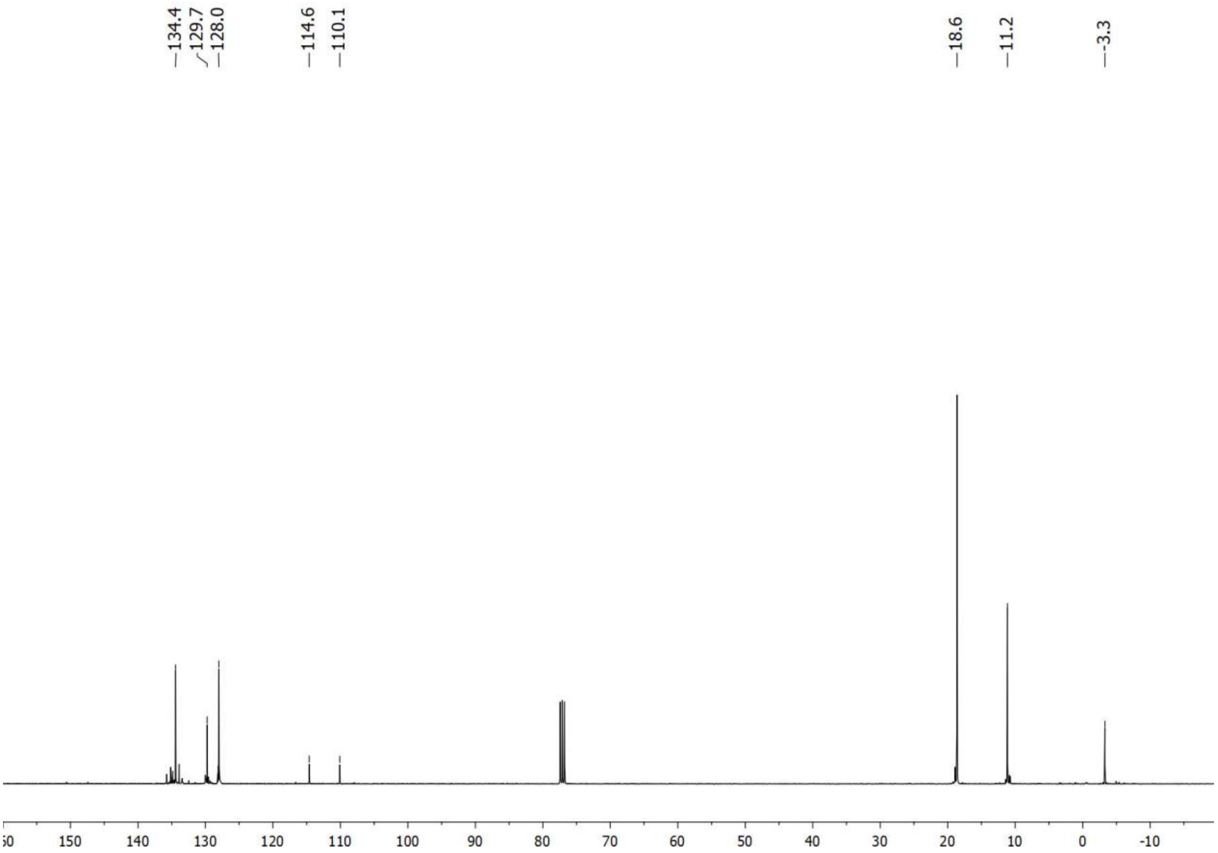

^{29}Si NMR

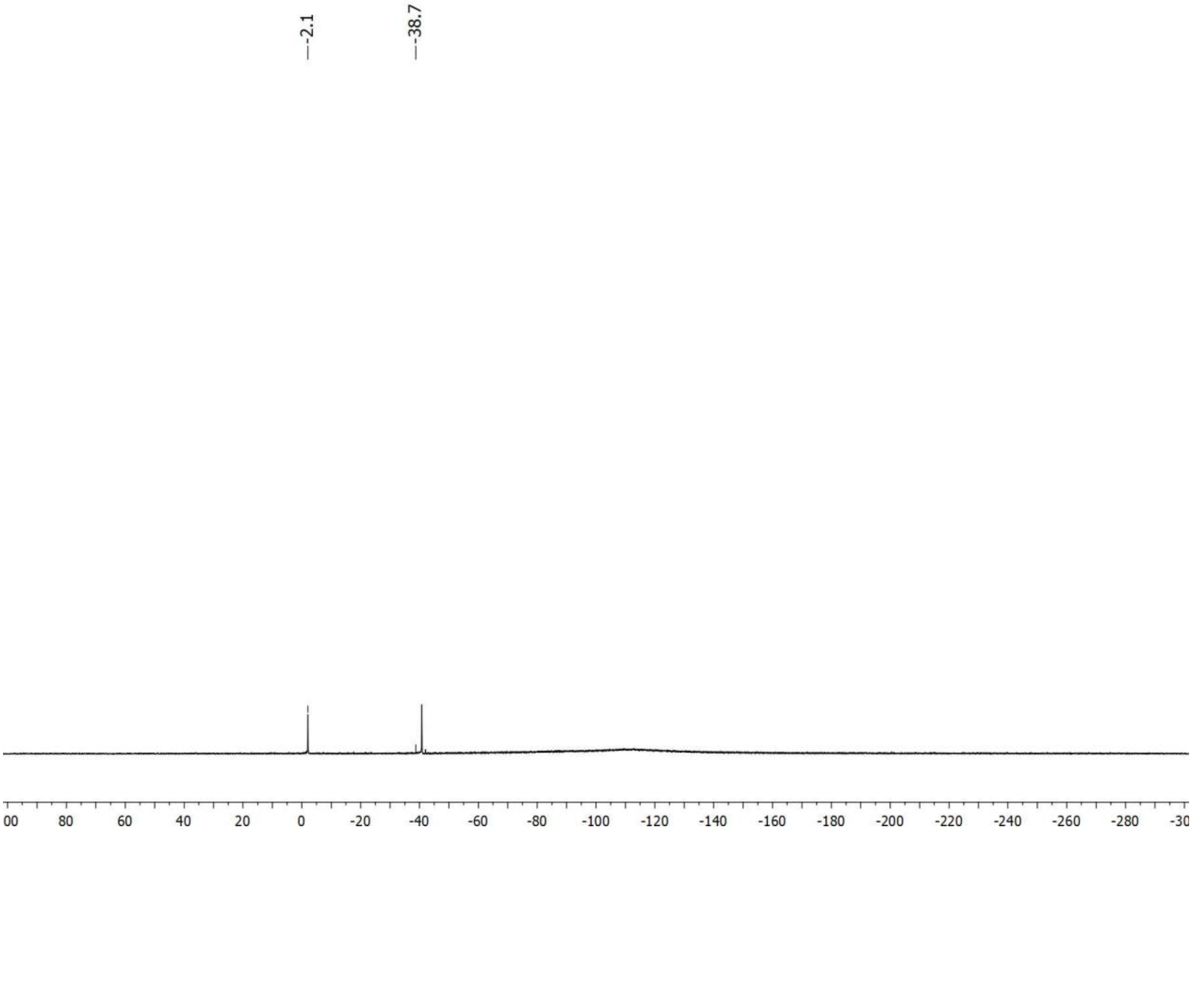

[(Methyl(p-tolyl)silyl)ethynyl]triisopropylsilane (3fc)

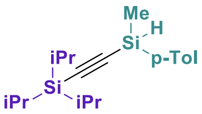

^{1}H NMR

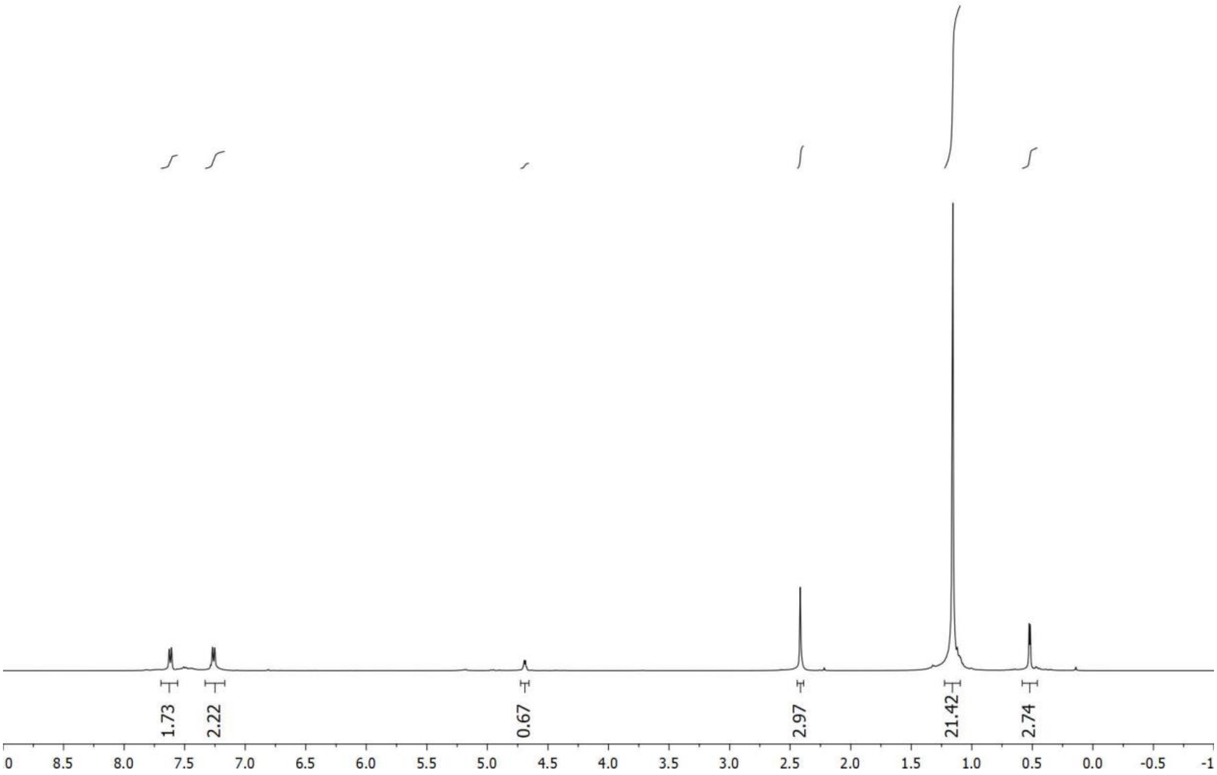

^{13}C NMR

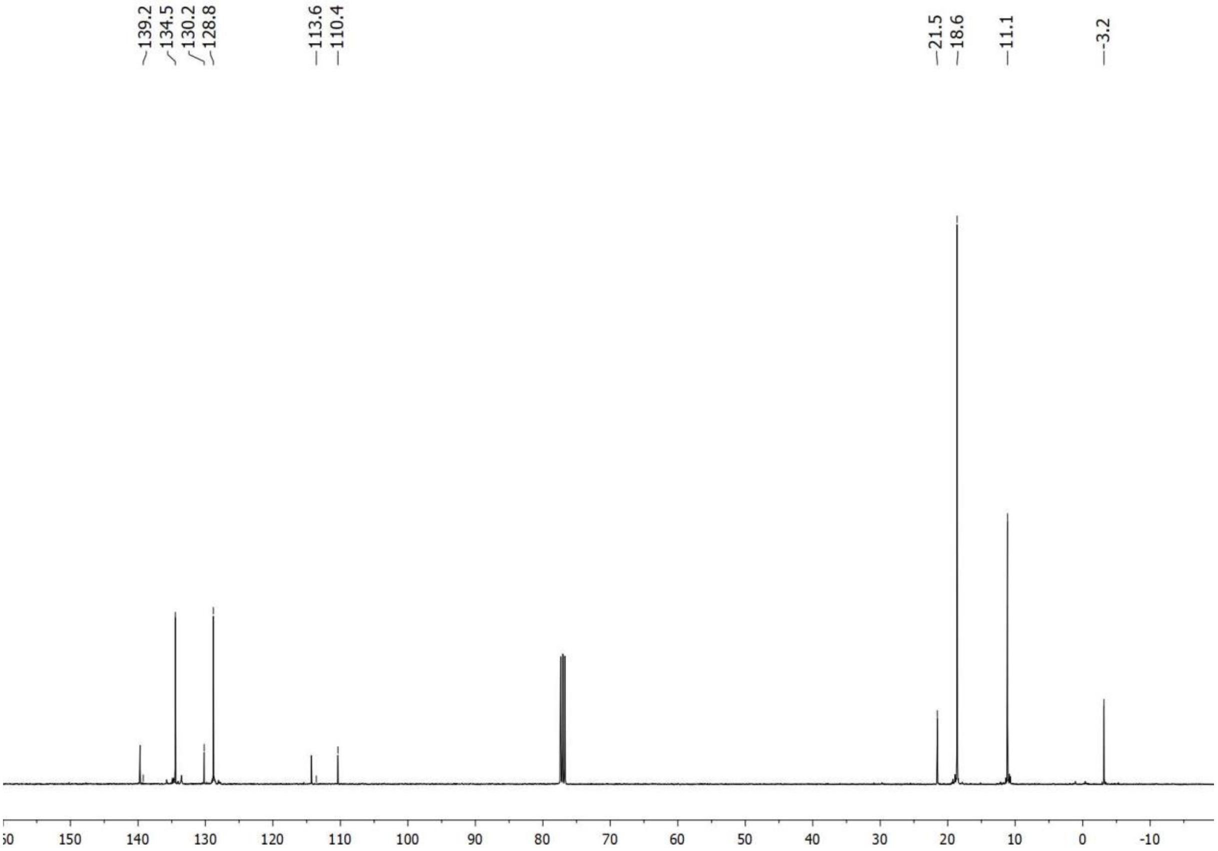

^{29}Si NMR

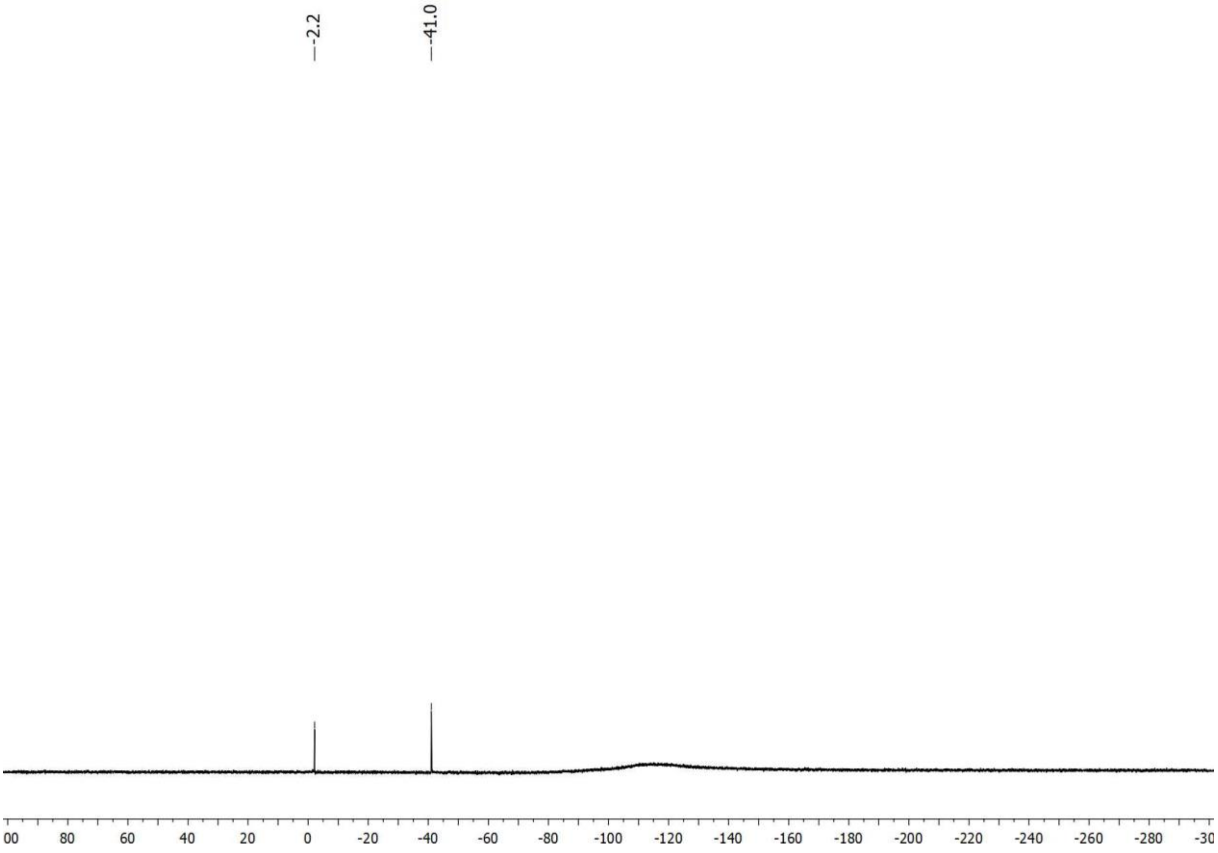

[(Methyl(octyl)silyl)ethynyl]triisopropylsilane (3gc)

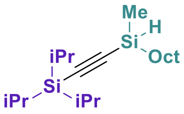

^{1}H NMR

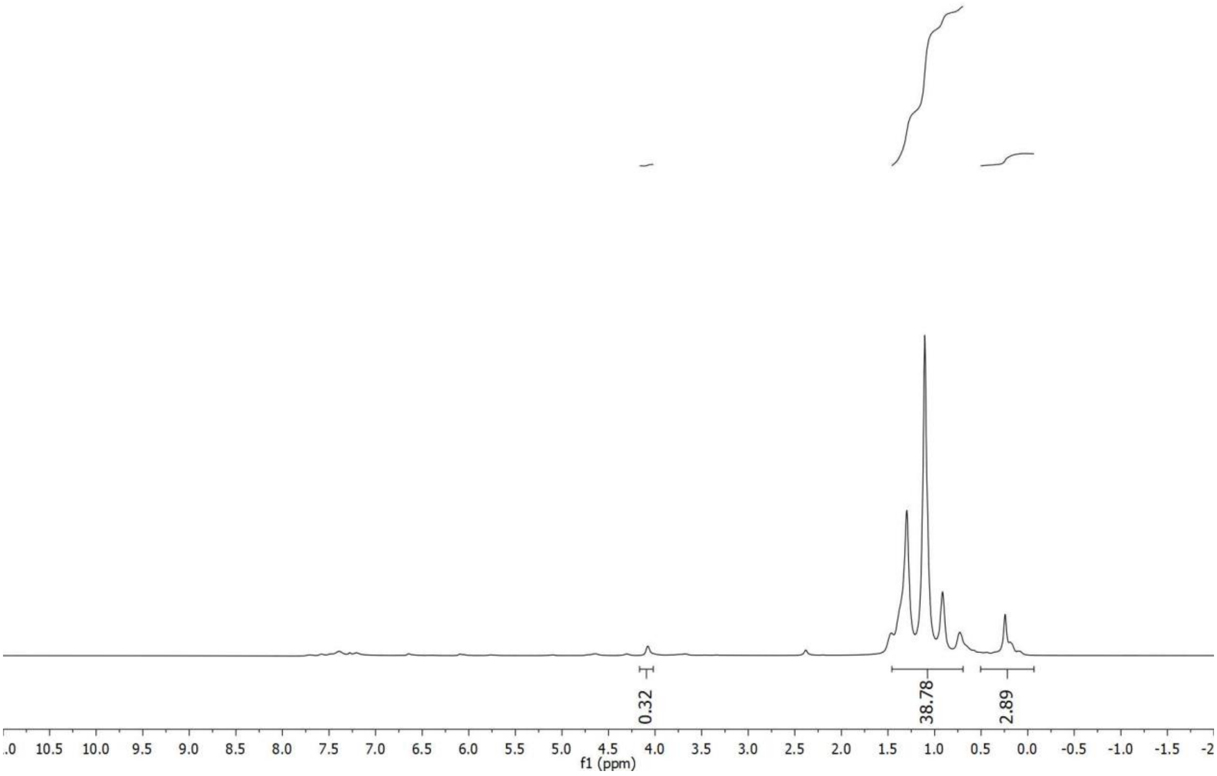

^{13}C NMR

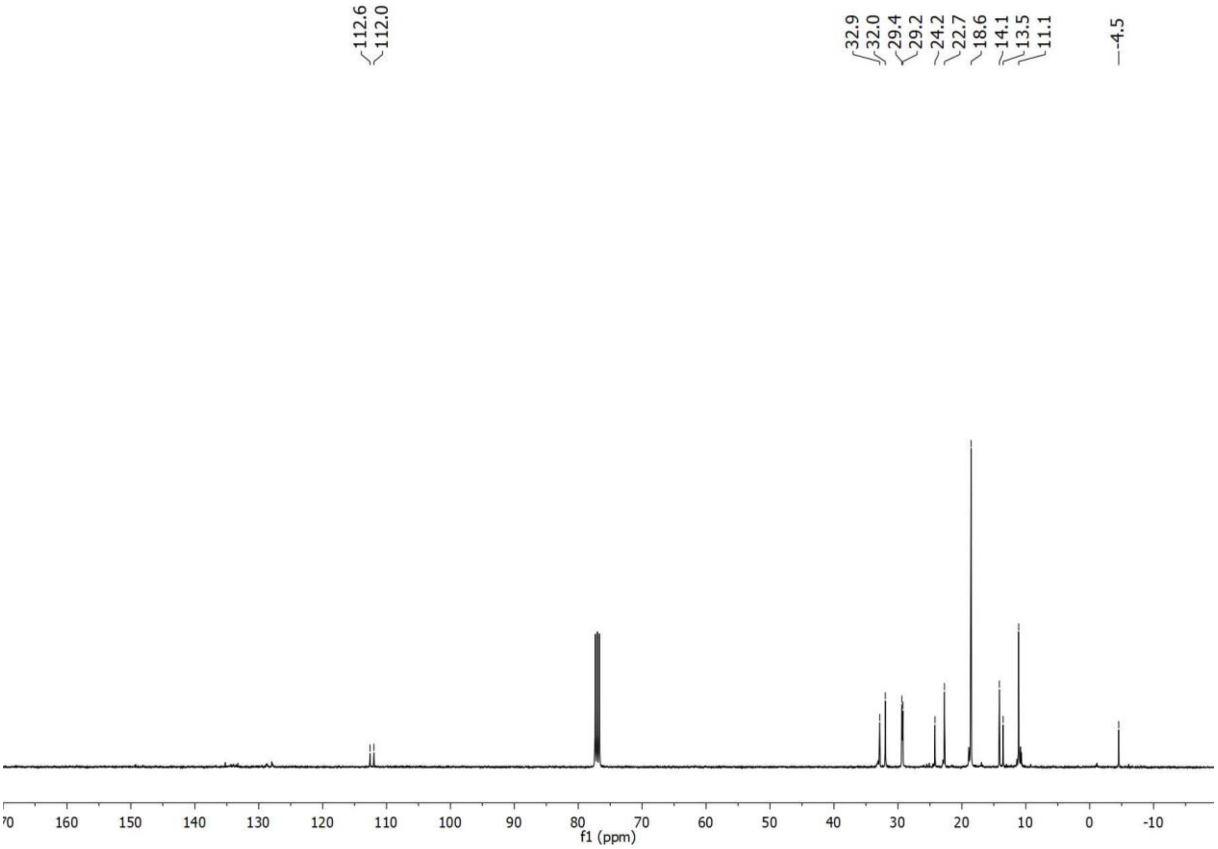

^{29}Si NMR

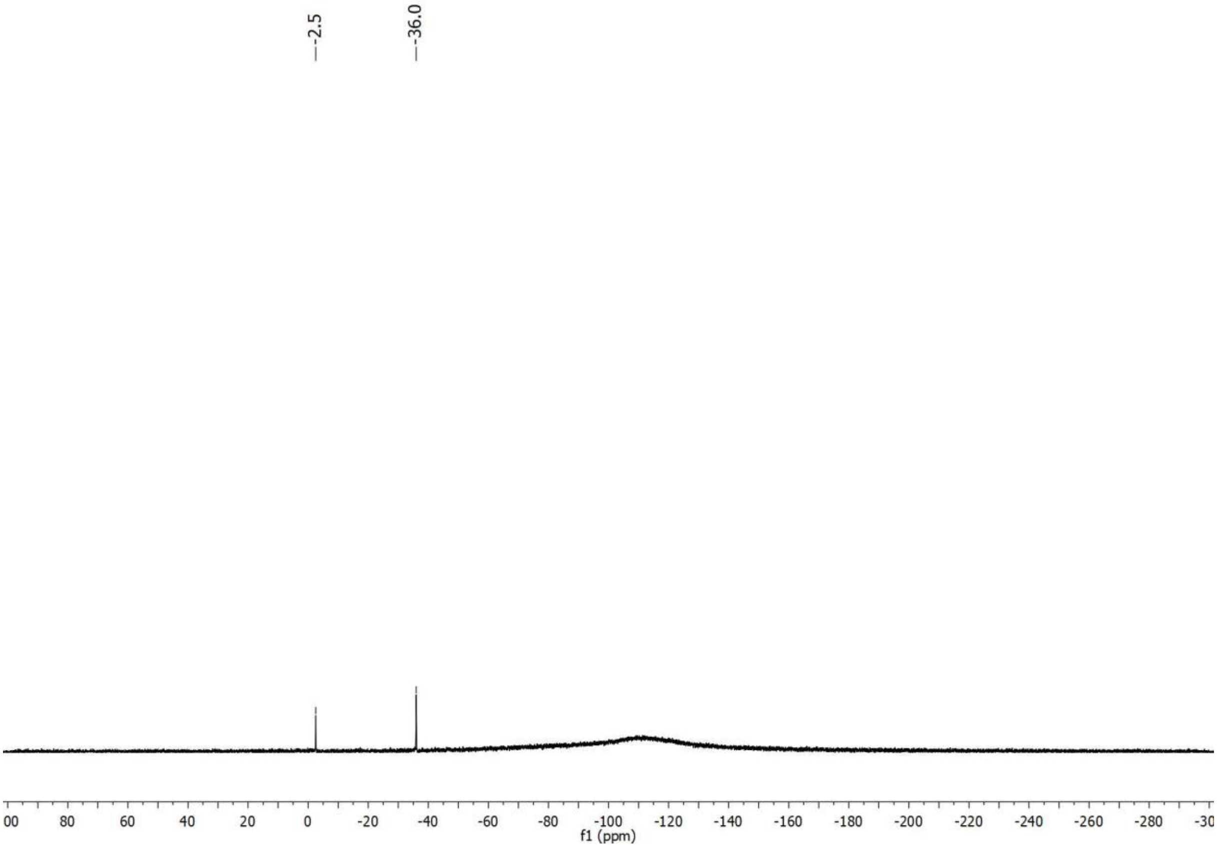

[[2-(Triethylsilyl)ethynyl][2-(trimethylsilyl)ethynyl]silyl]benzene (5ab-a)

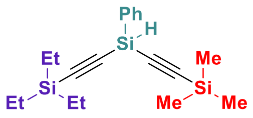

^{1}H NMR

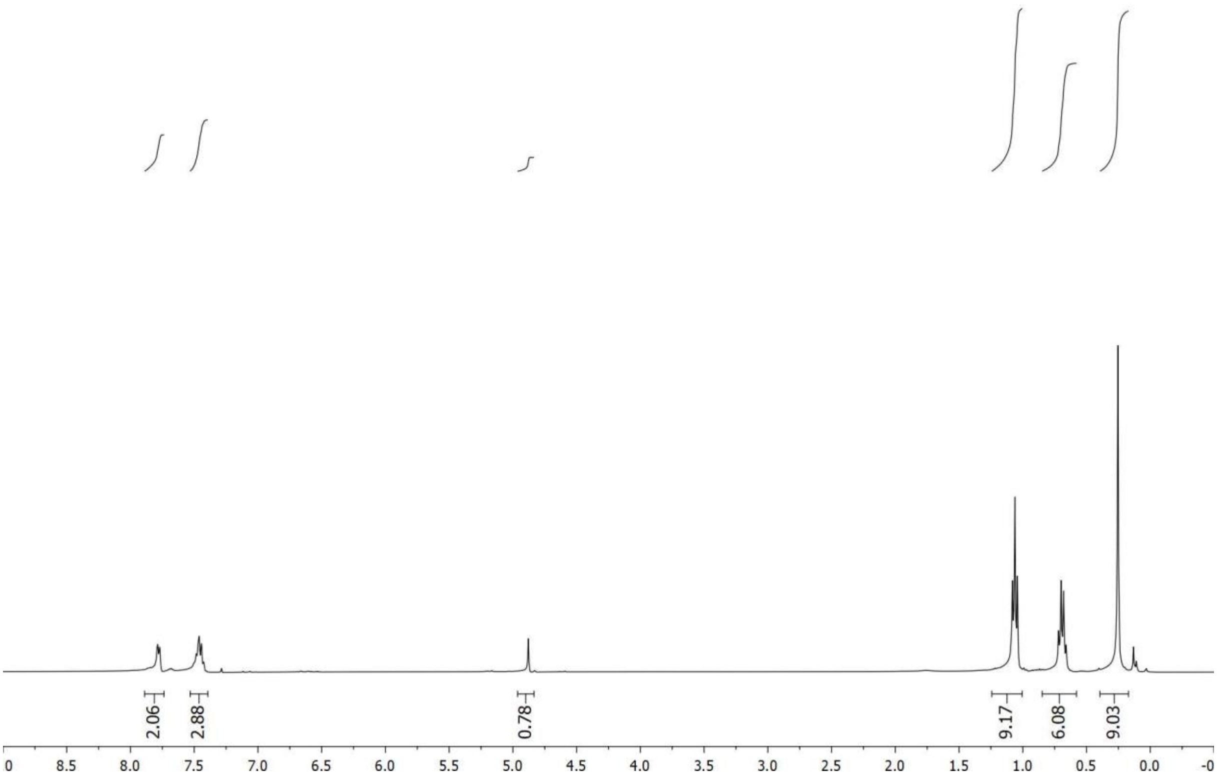

^{13}C NMR

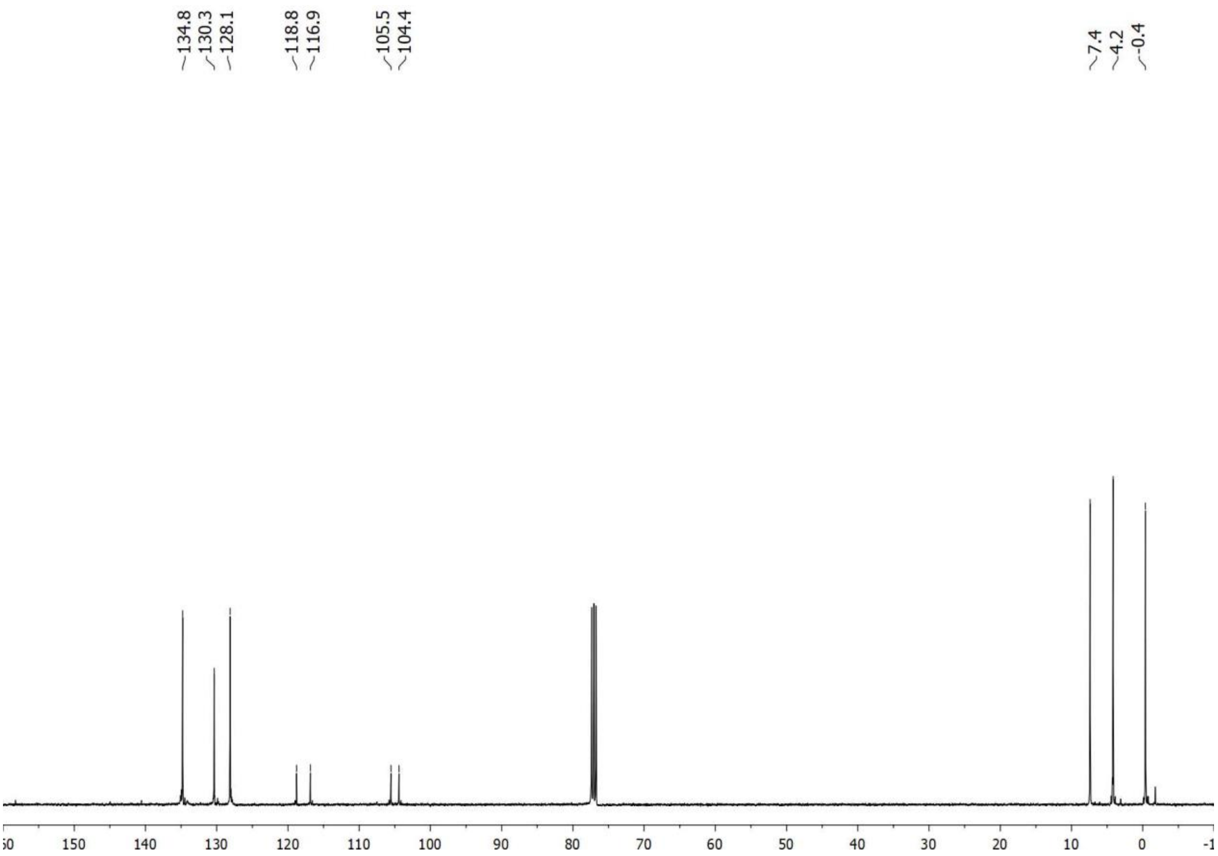

^{29}Si NMR

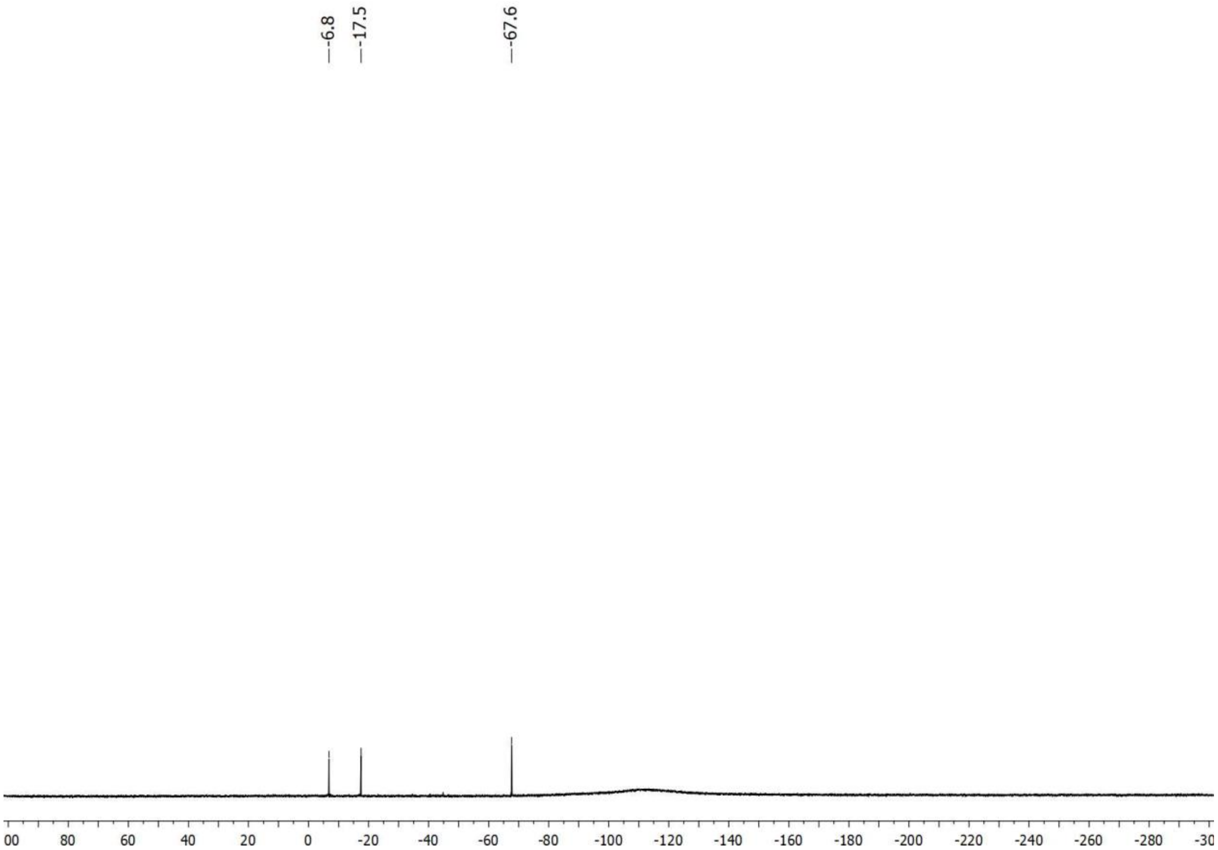

[[2-(Triethylsilyl)ethynyl][2-(tri-n-propylsilyl)ethynyl]silyl]benzene (5ab-e)

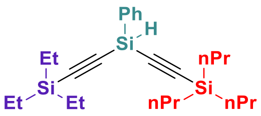

^{1}H NMR

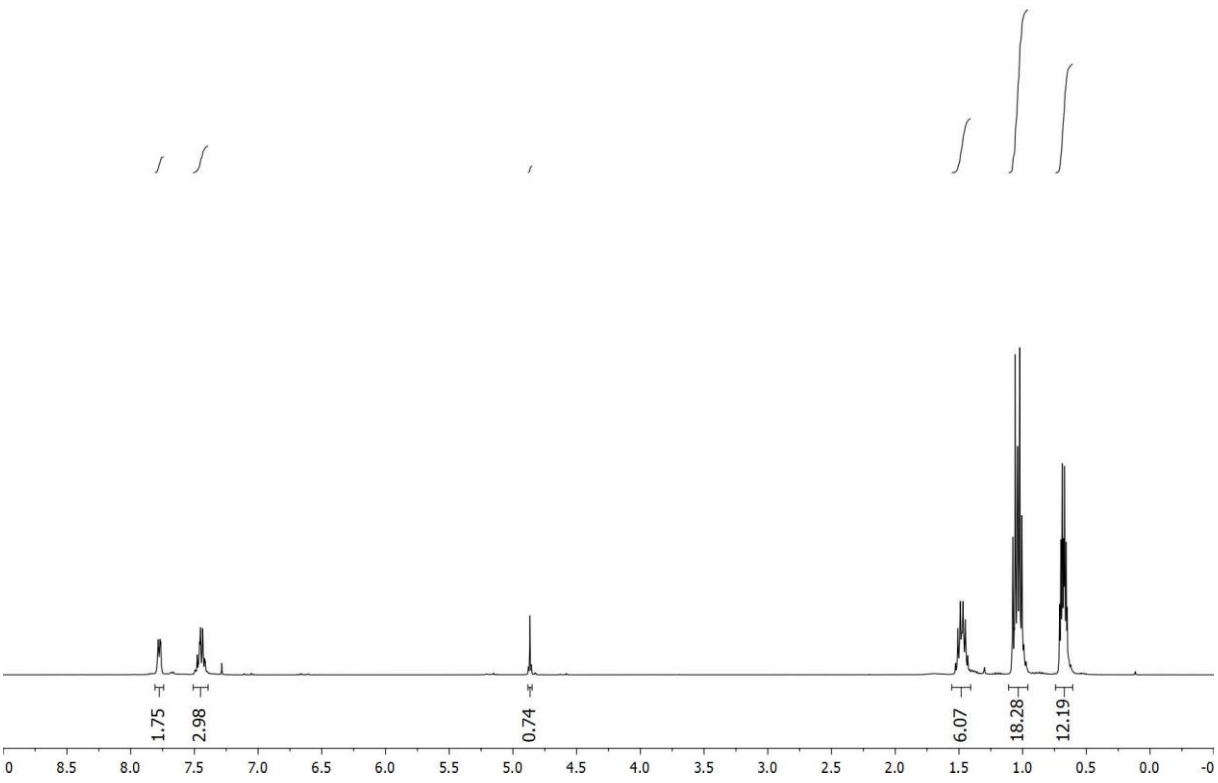

^{13}C NMR

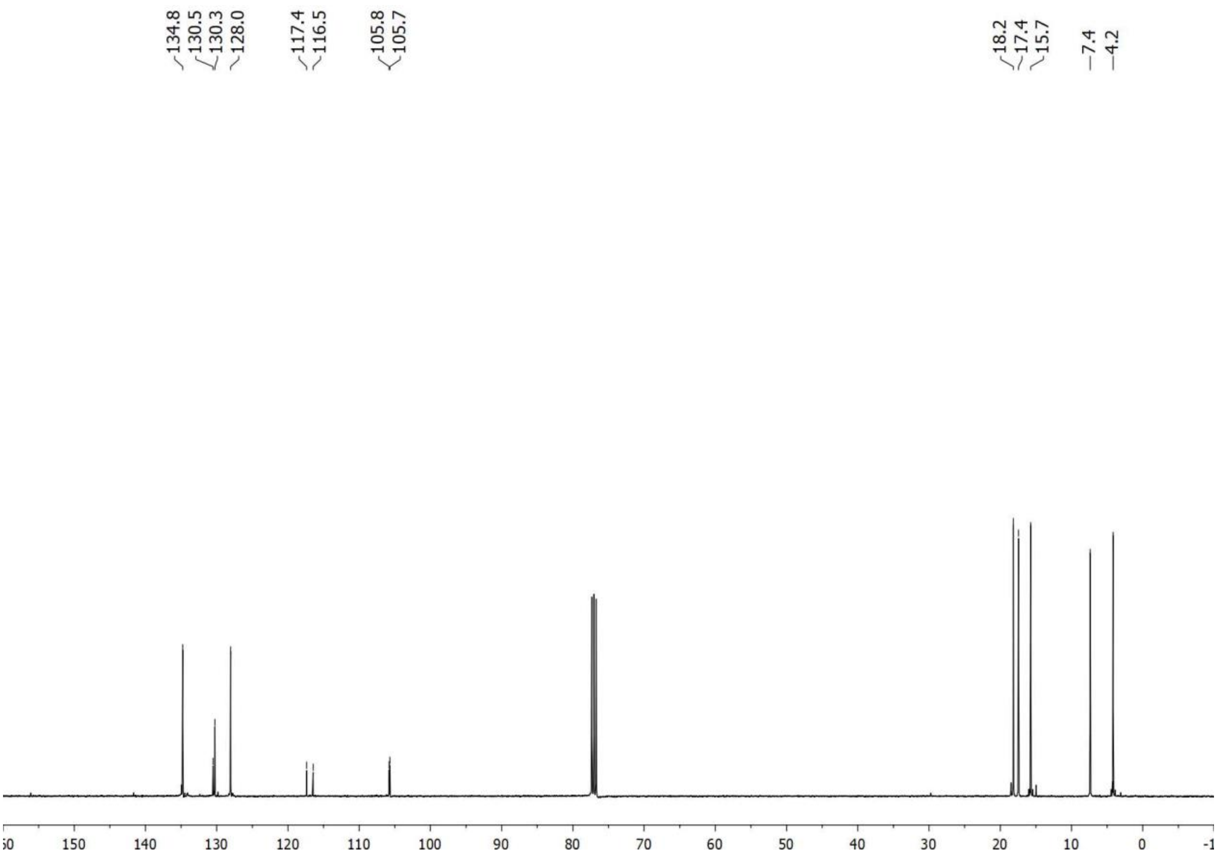

^{29}Si NMR

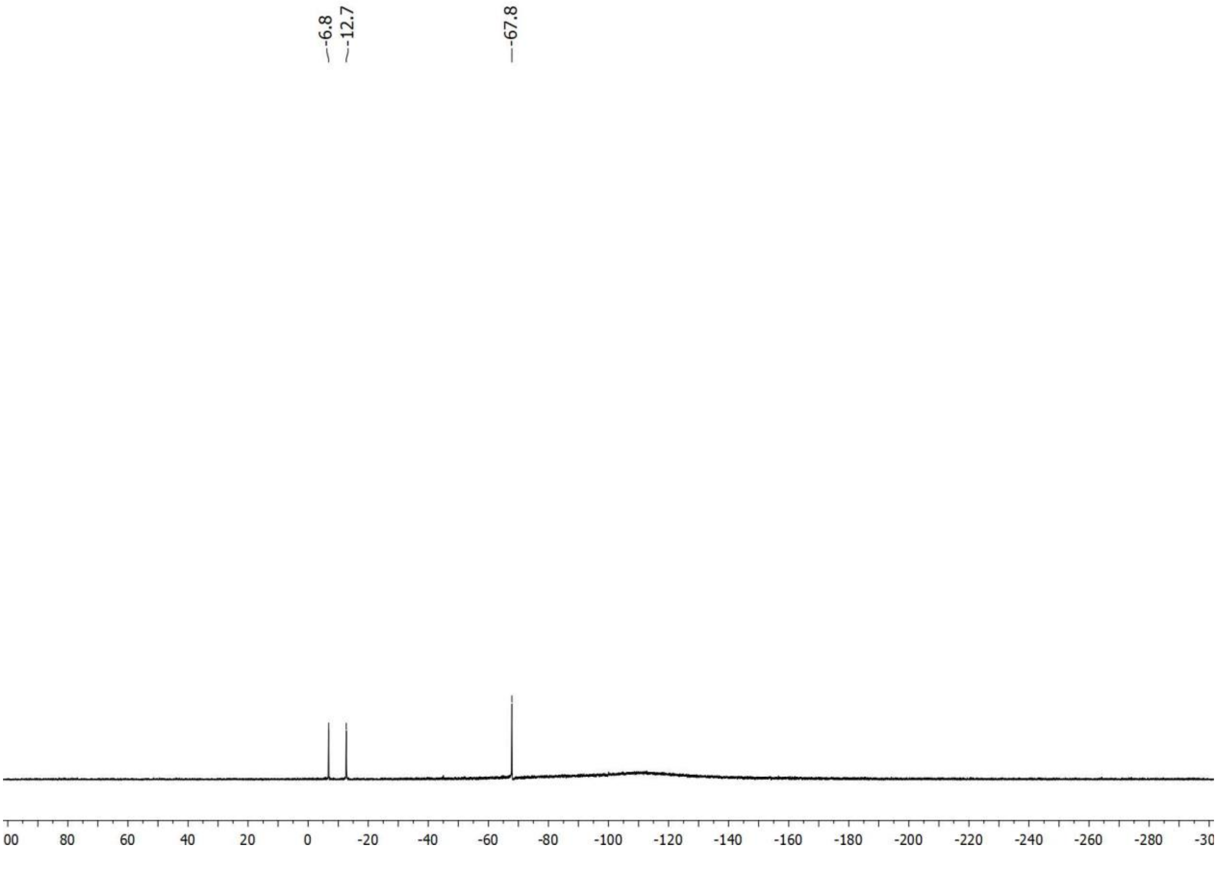

[[2-(Tert-butyl(dimethyl)silyl)ethynyl][2-(triethylsilyl)ethynyl]silyl]benzene (5ab-g)

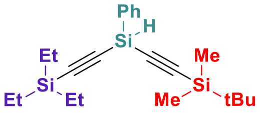

^{1}H NMR

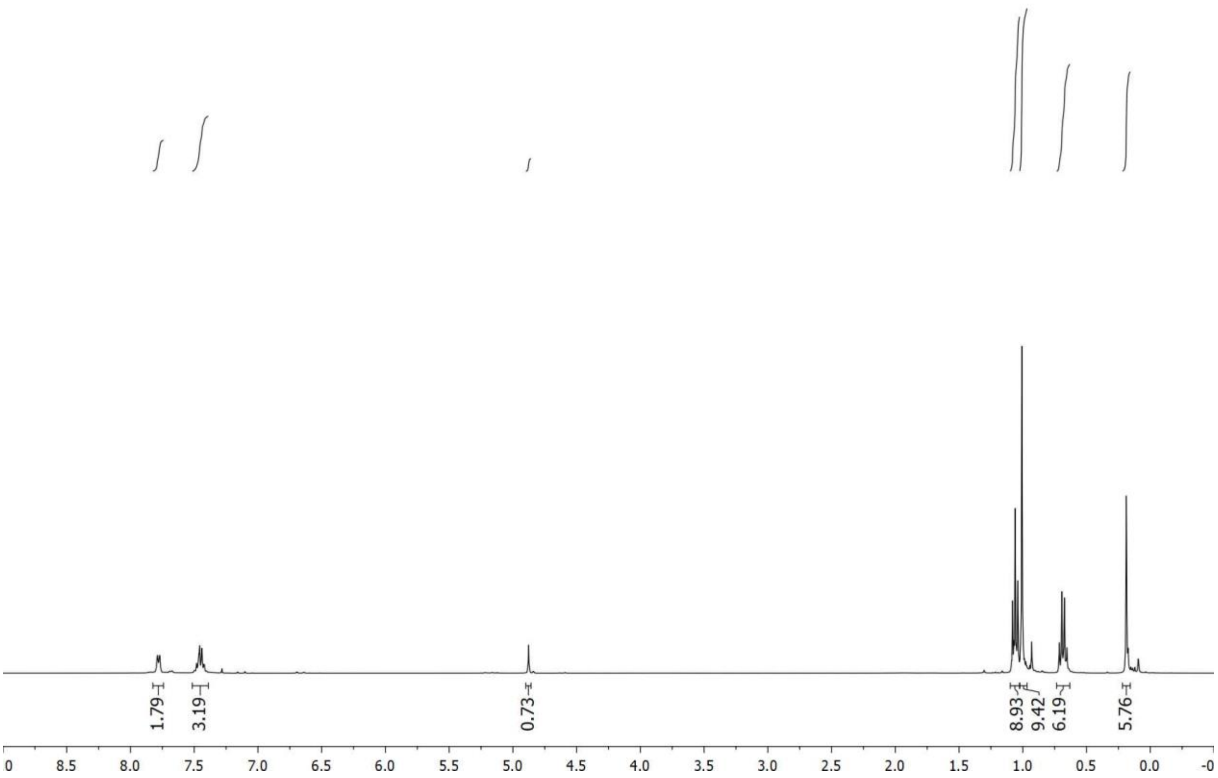

^{13}C NMR

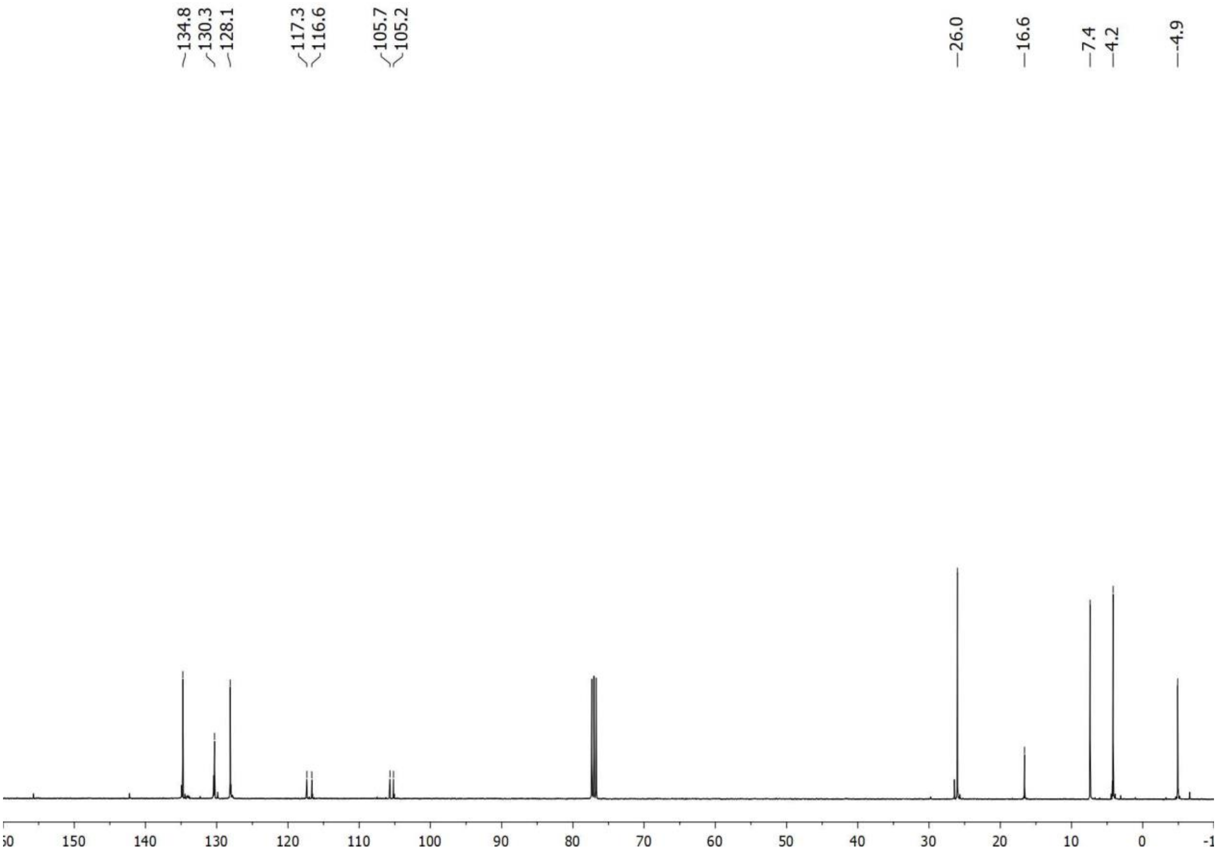

^{29}Si NMR

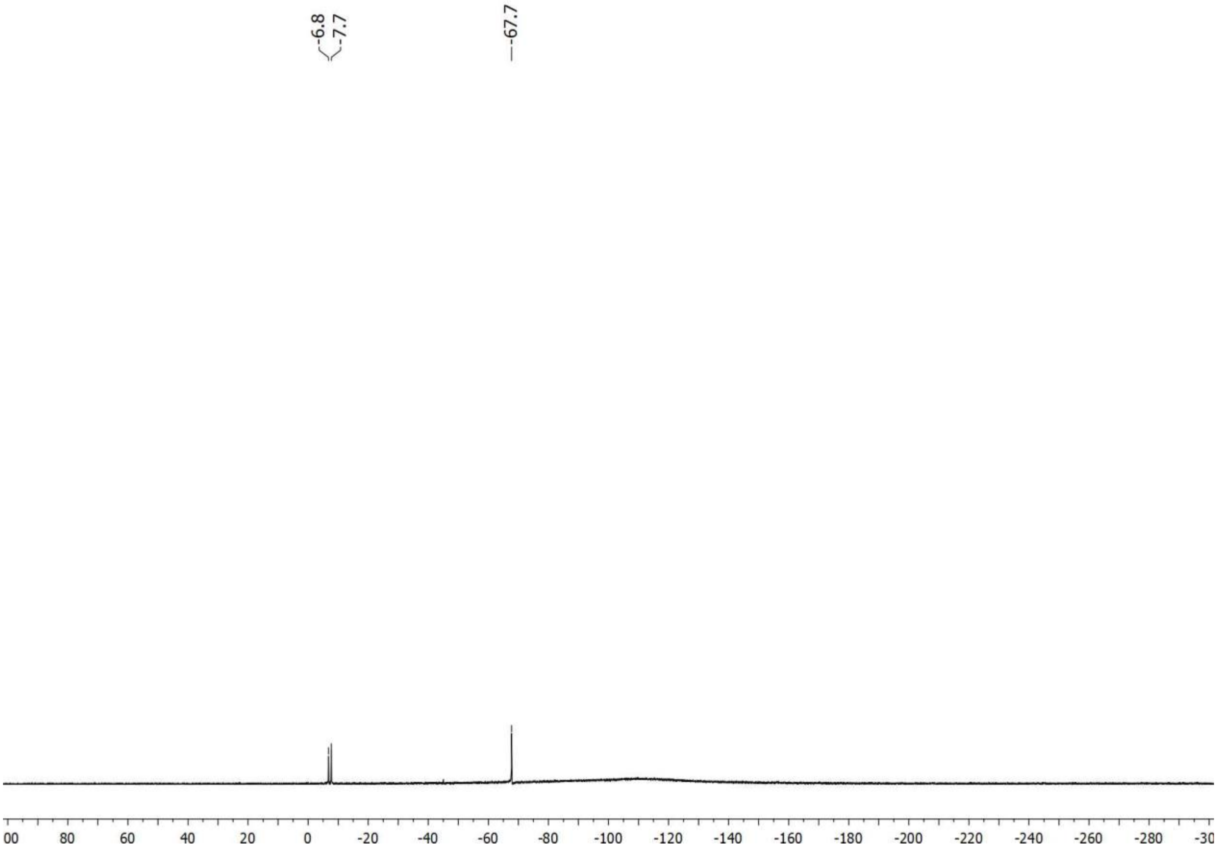

[[2-(Triisopropylsilyl)ethynyl][2-(trimethylsilyl)ethynyl]silyl]benzene (5ac-a)

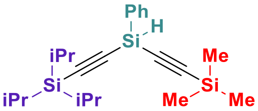

^{1}H NMR

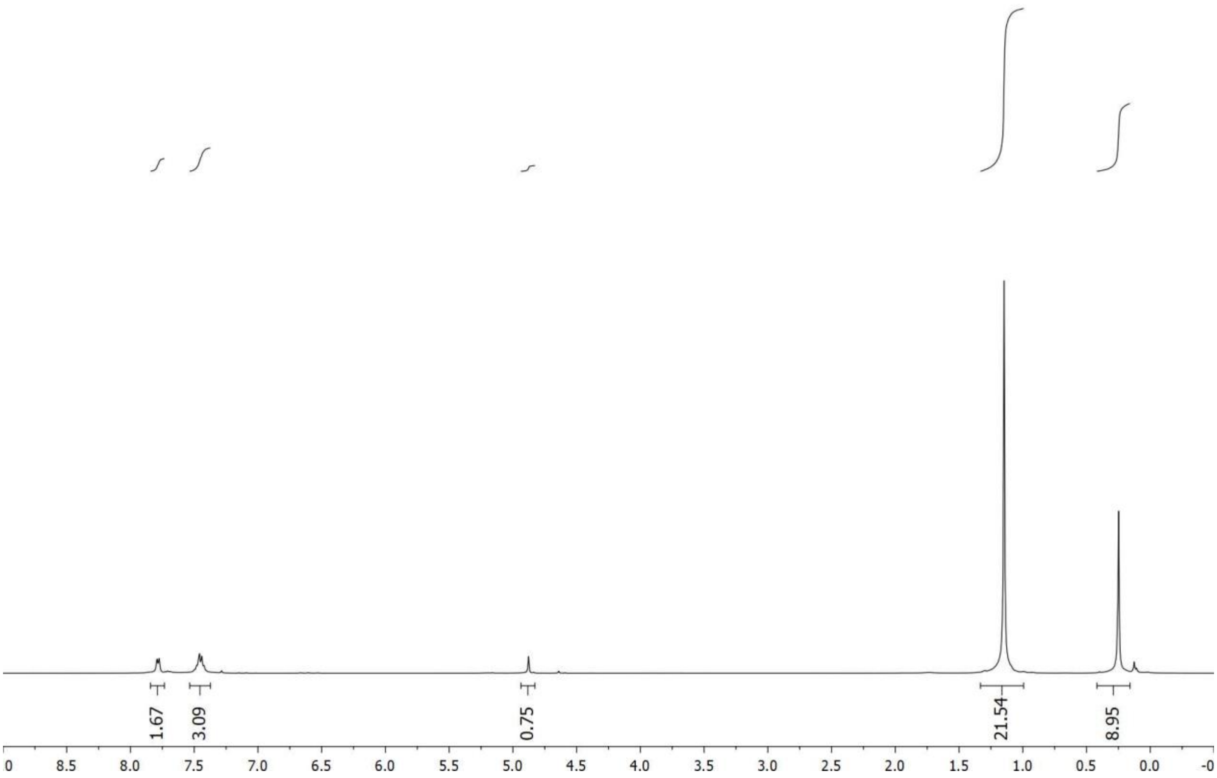

^{13}C NMR

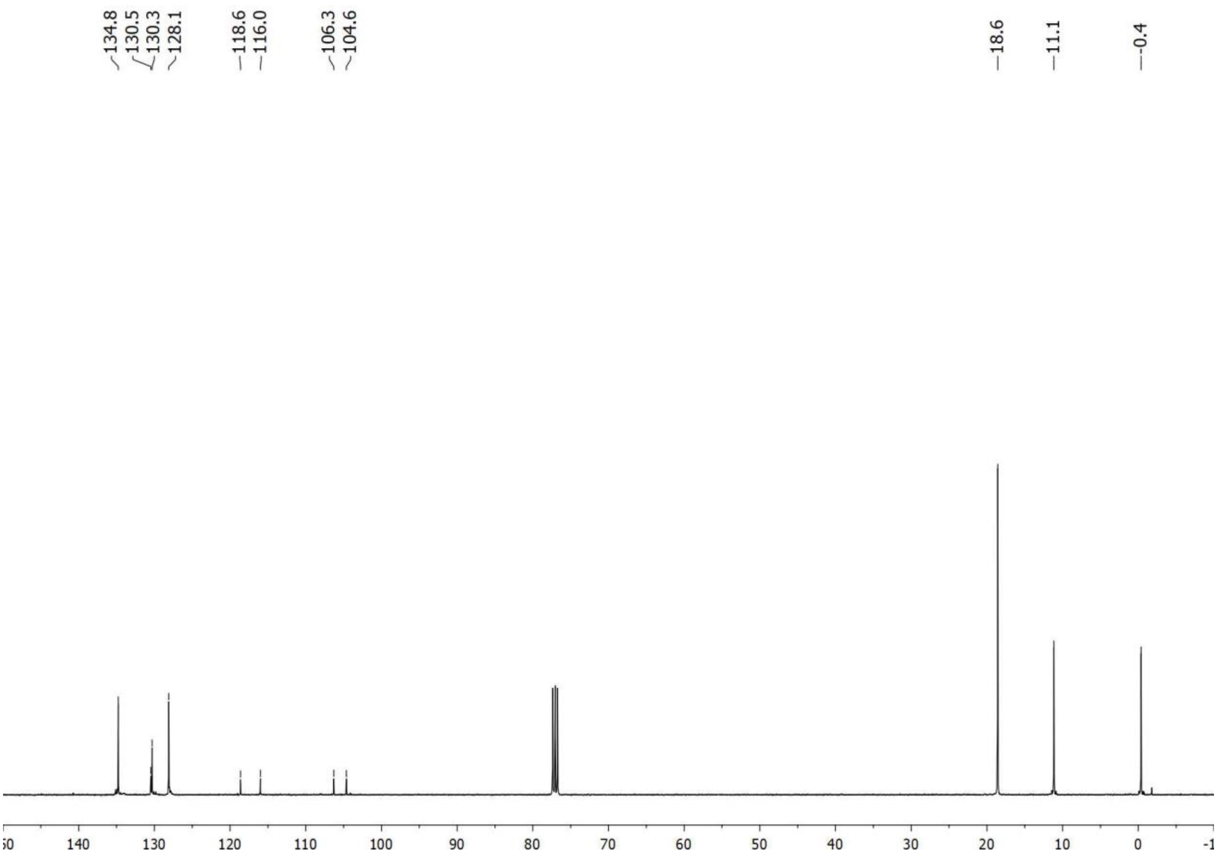

^{29}Si NMR

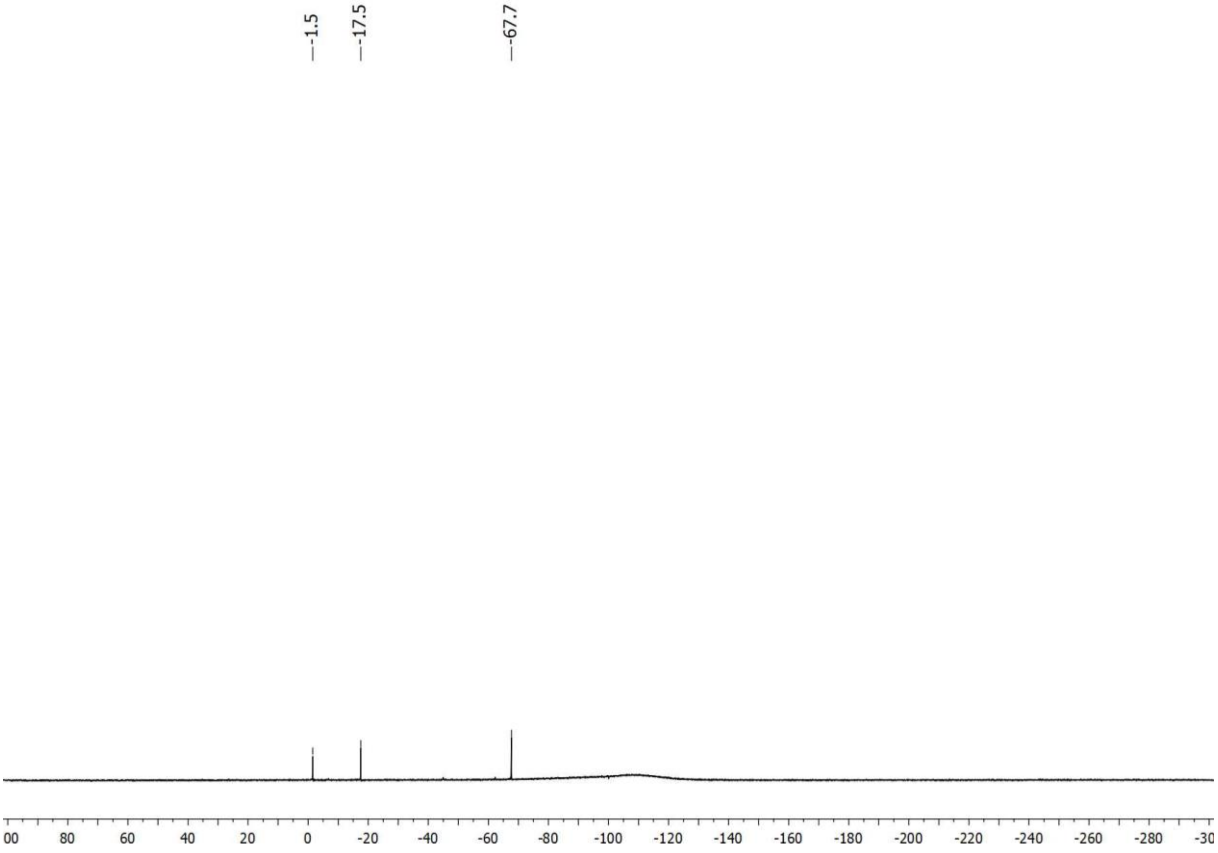

[[2-(Triethylsilyl)ethynyl][2-(triisopropylsilyl)ethynyl]silyl]benzene (5ac-b)

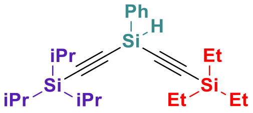

^{1}H NMR

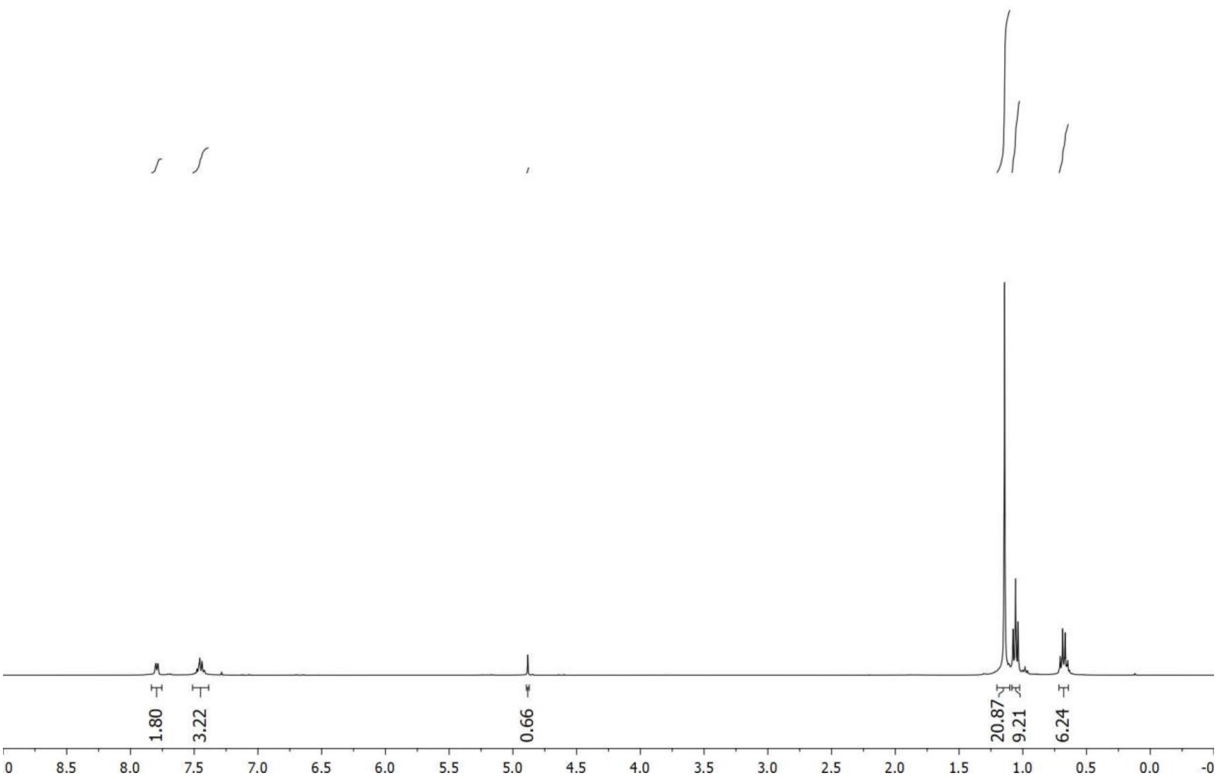

^{13}C NMR

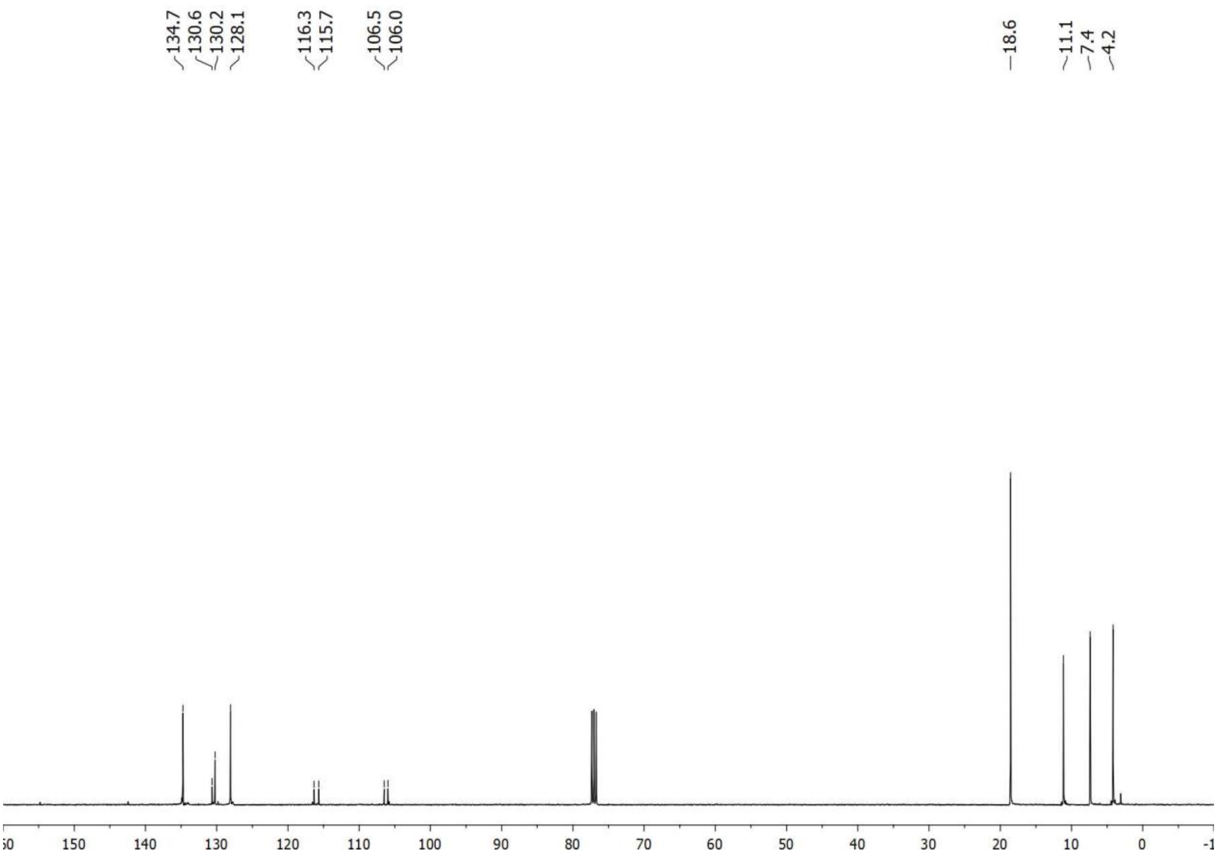

^{29}Si NMR

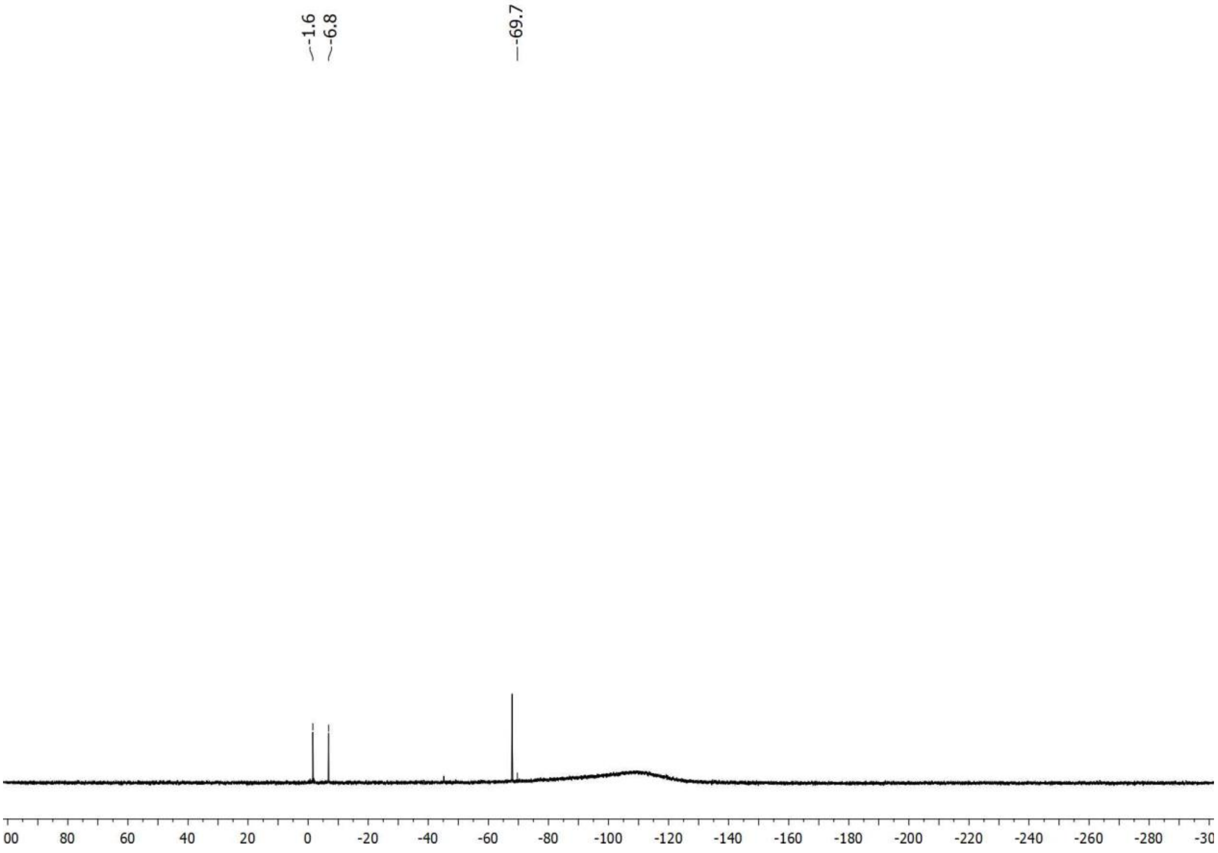

[[2-(Tert-butyl(dimethyl)silyl)ethynyl][2-(triisopropylsilyl)ethynyl]silyl]benzene (5ac-g)

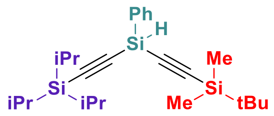

^{1}H NMR

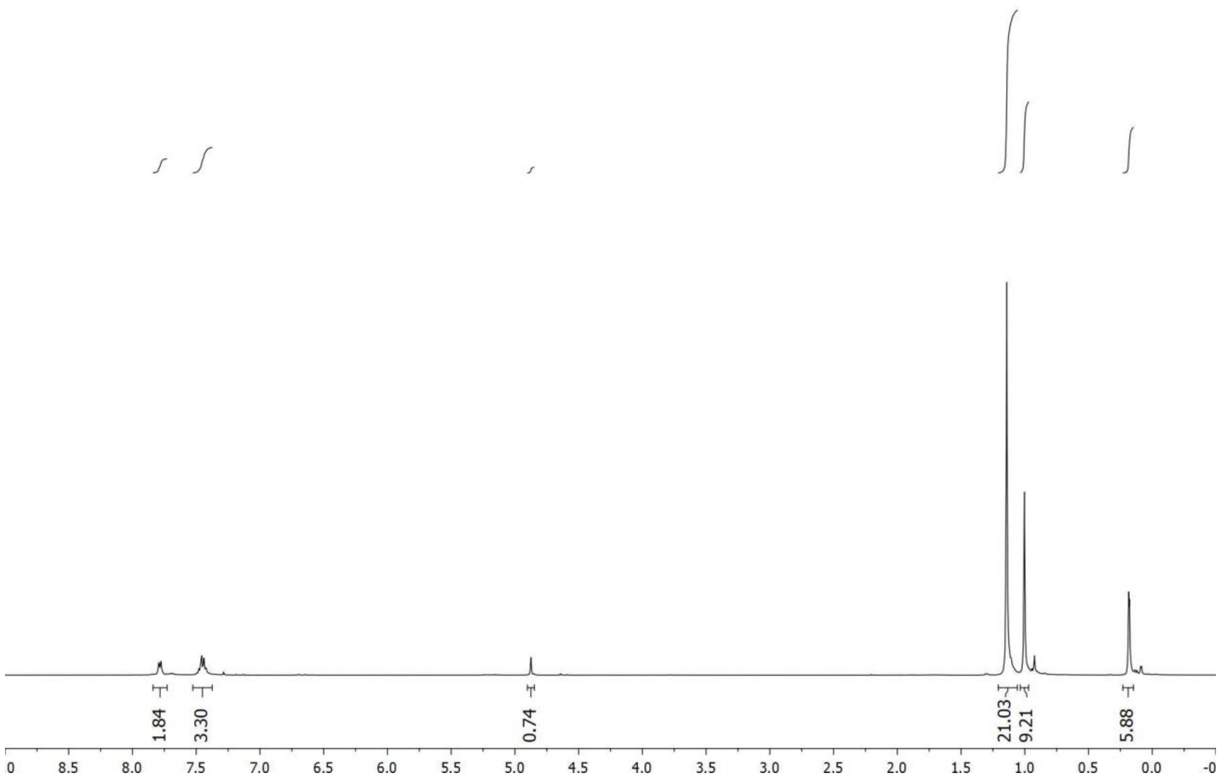

^{13}C NMR

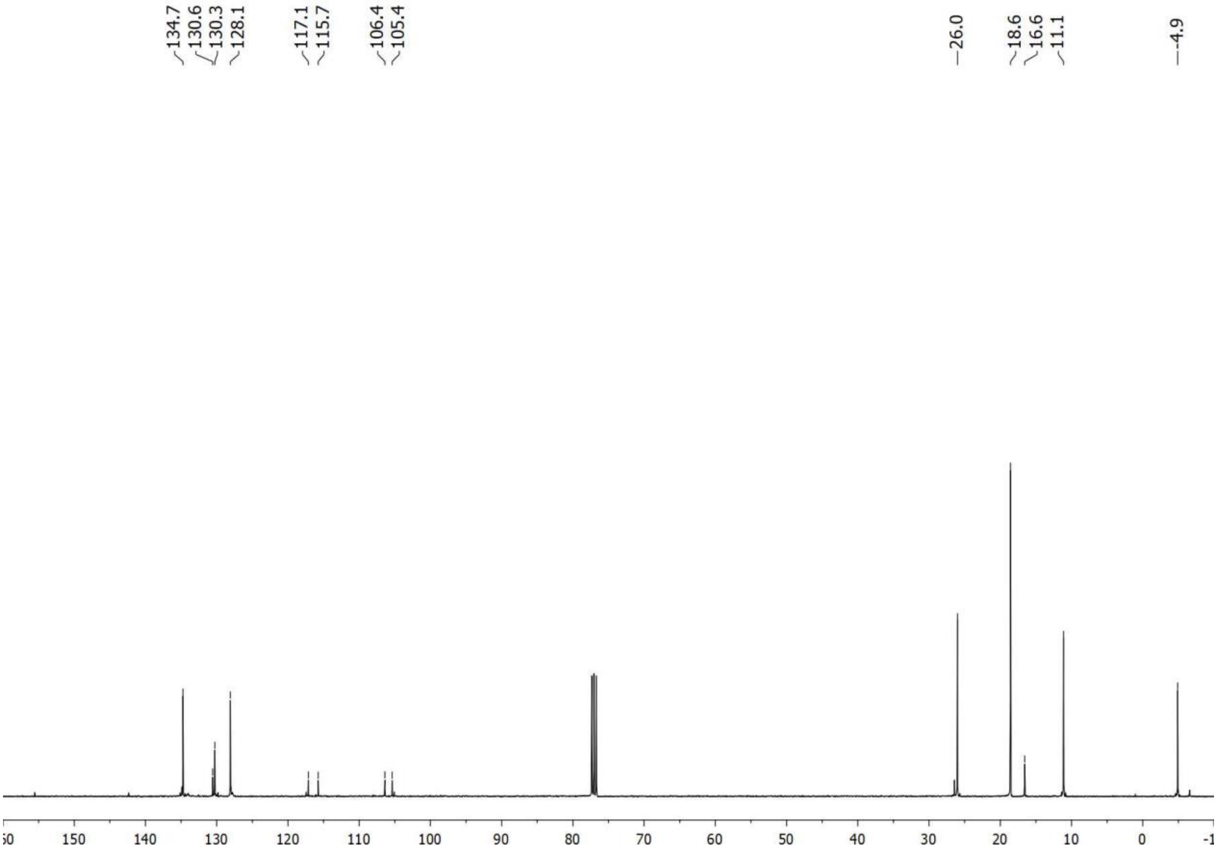

^{29}Si NMR

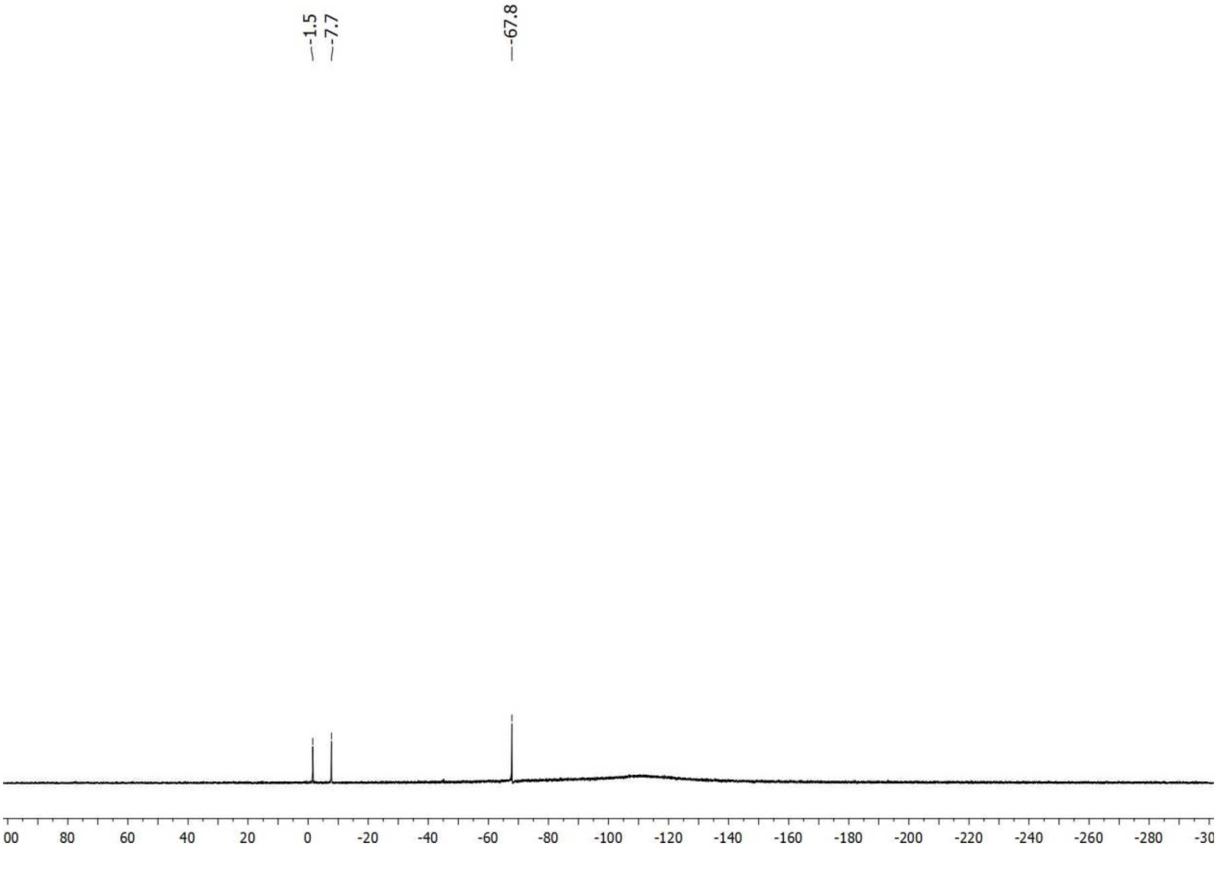

((Decyl(phenyl)silyl)ethynyl)triethylsilane (7ab-a)

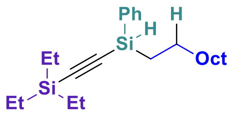

^{1}H NMR

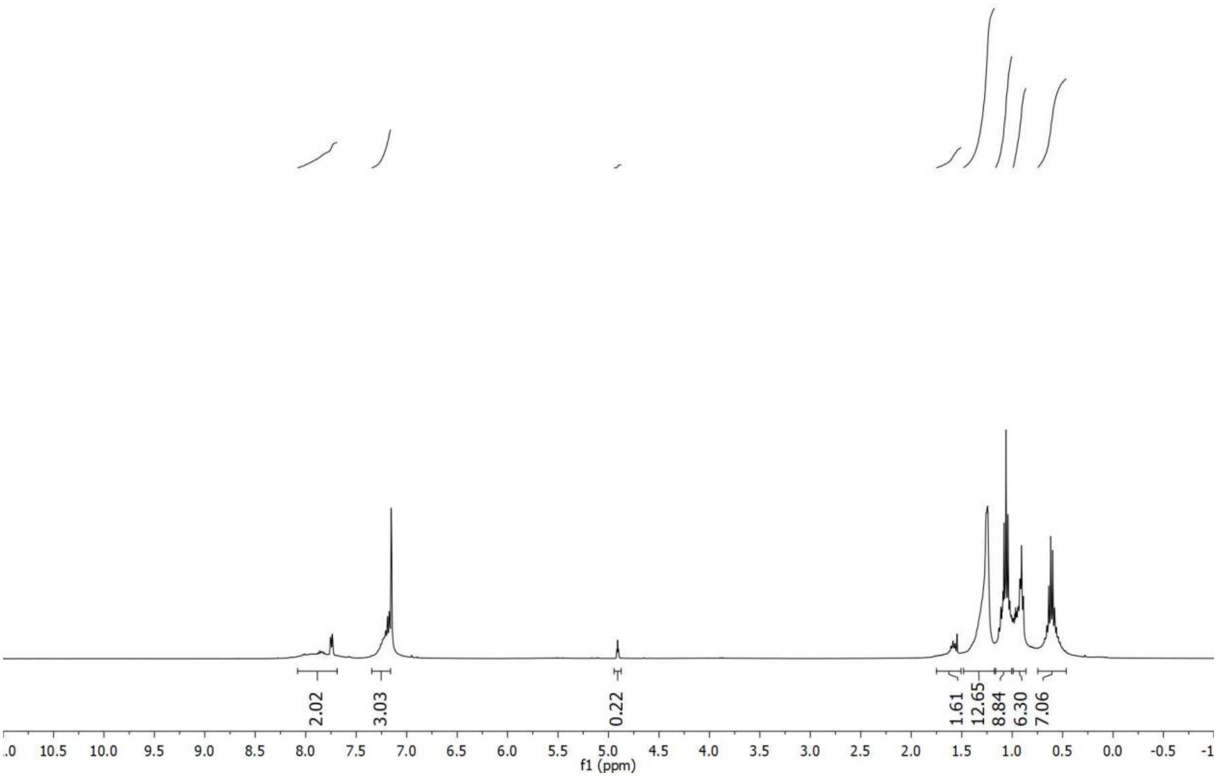

^{13}C NMR

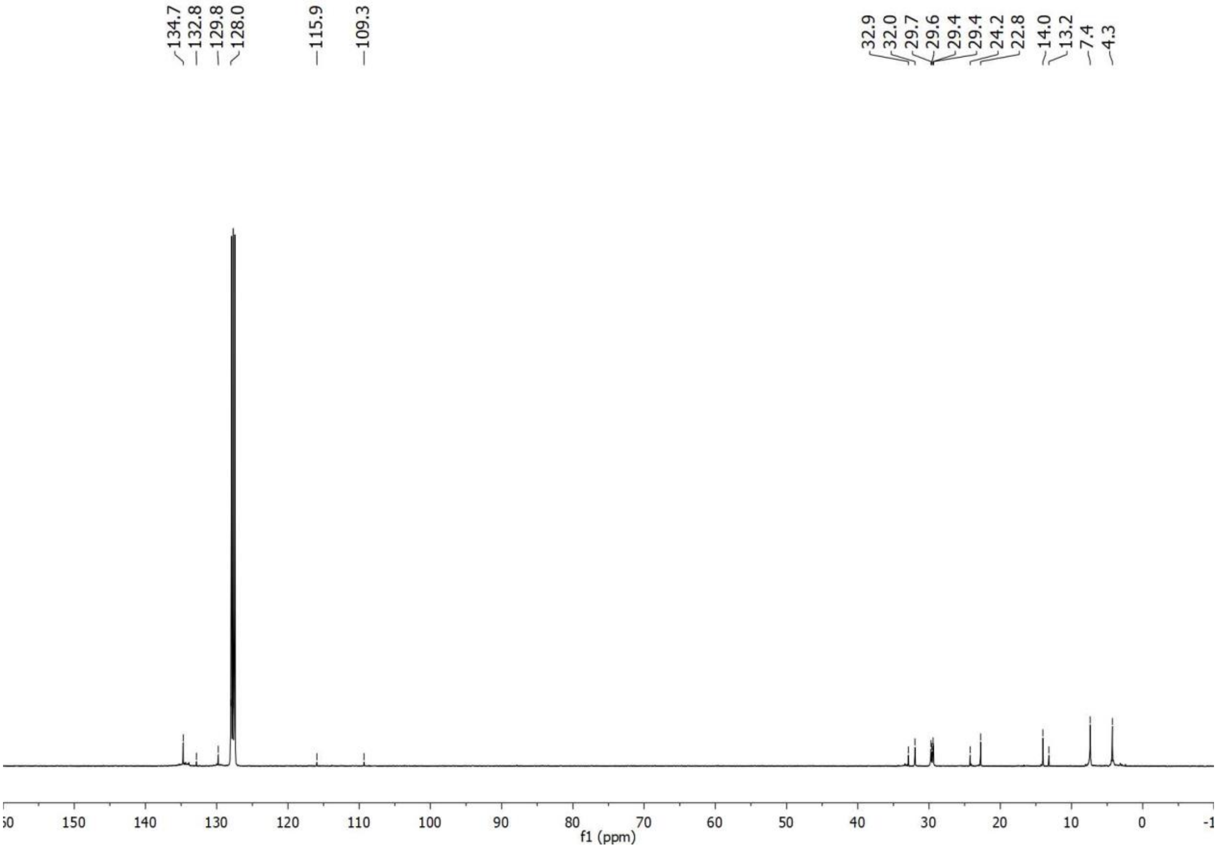

^{29}Si NMR

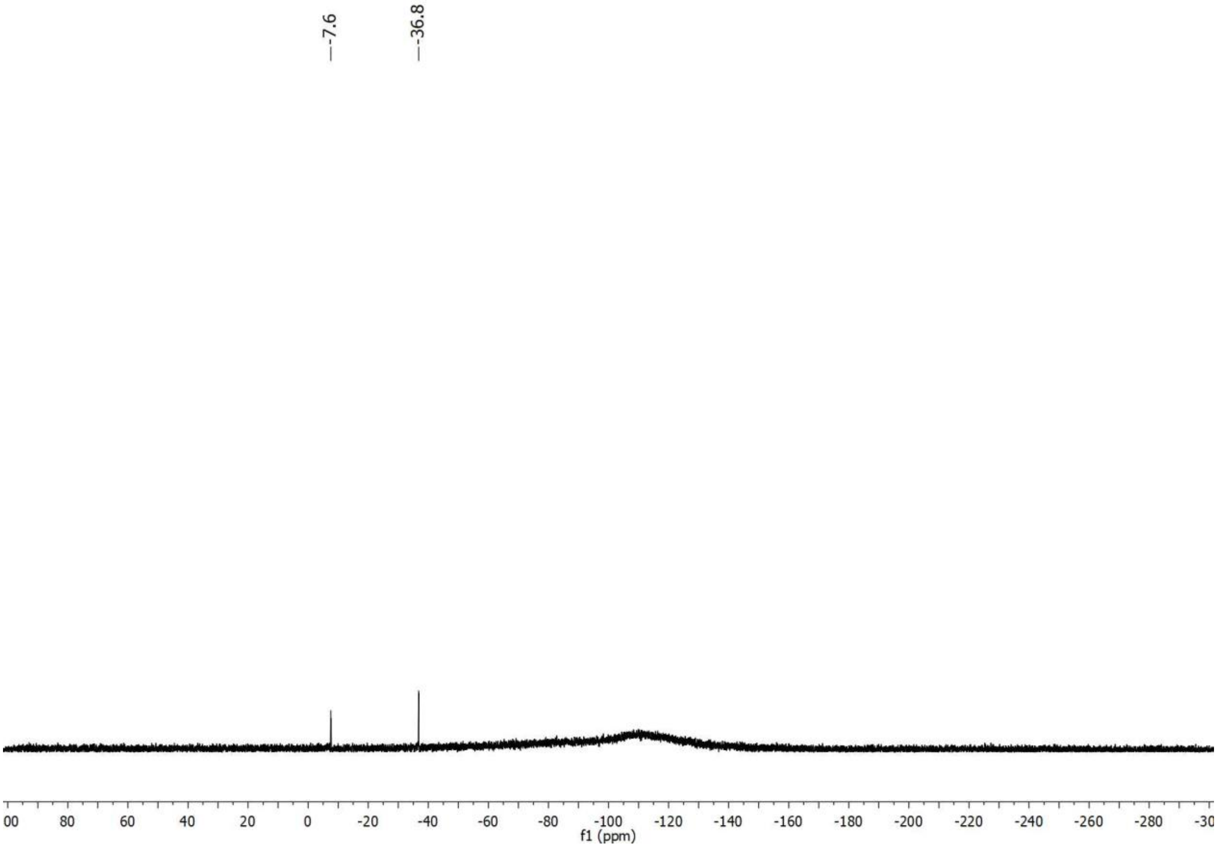

((Decyl(phenyl)silyl)ethynyl)triisopropylsilane (7ac-a)

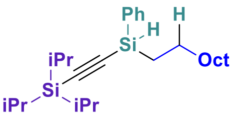

^{1}H NMR

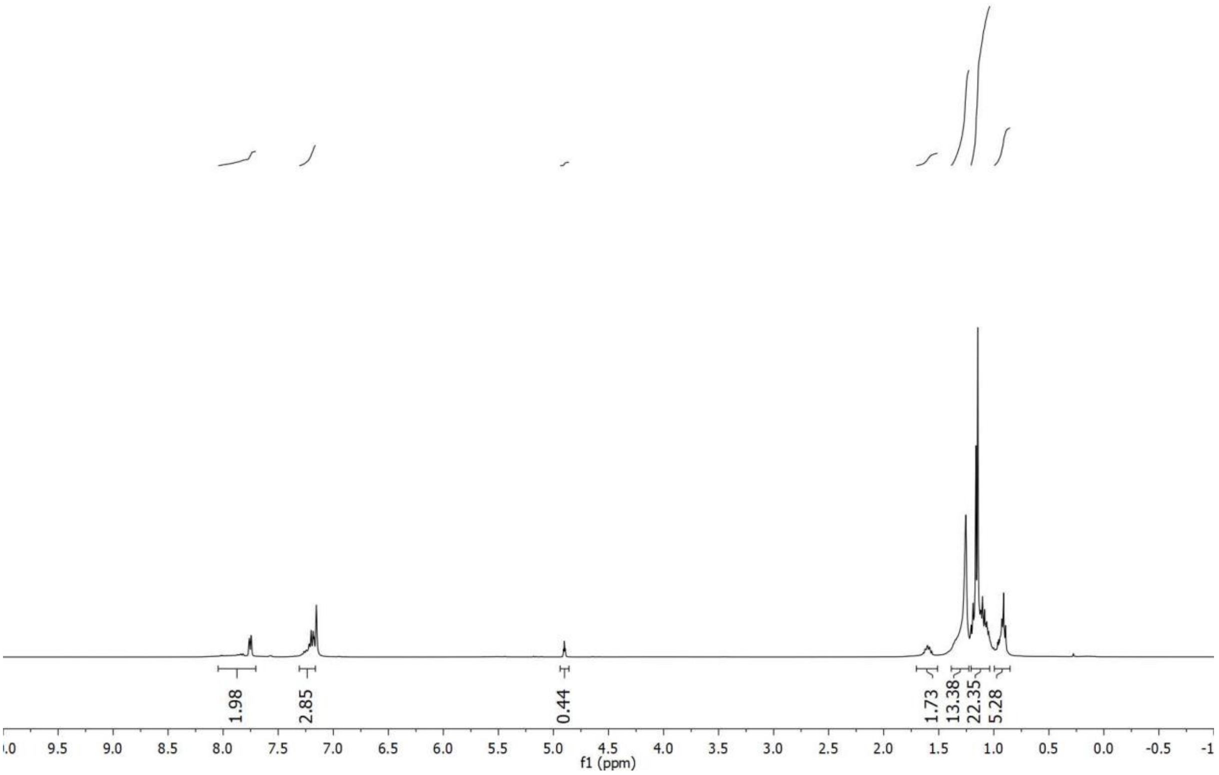

^{13}C NMR

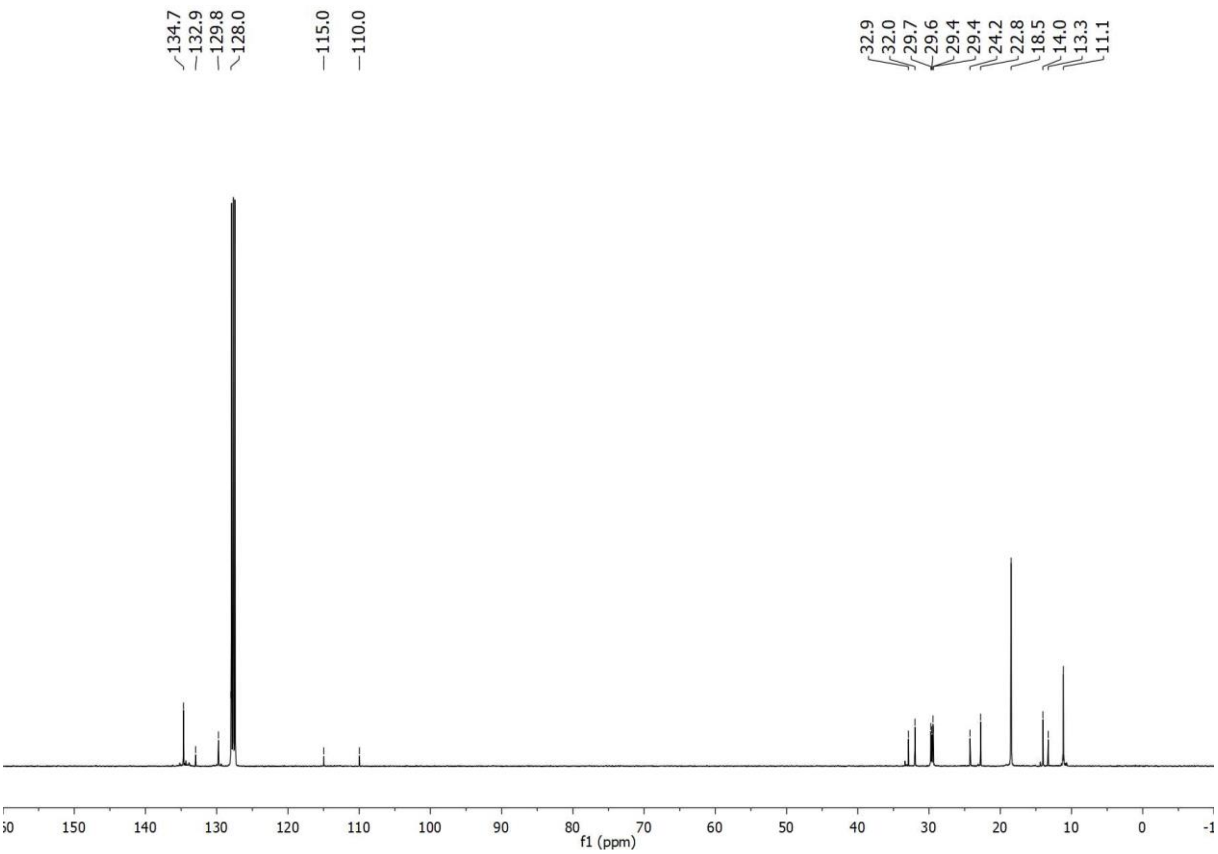

^{29}Si NMR

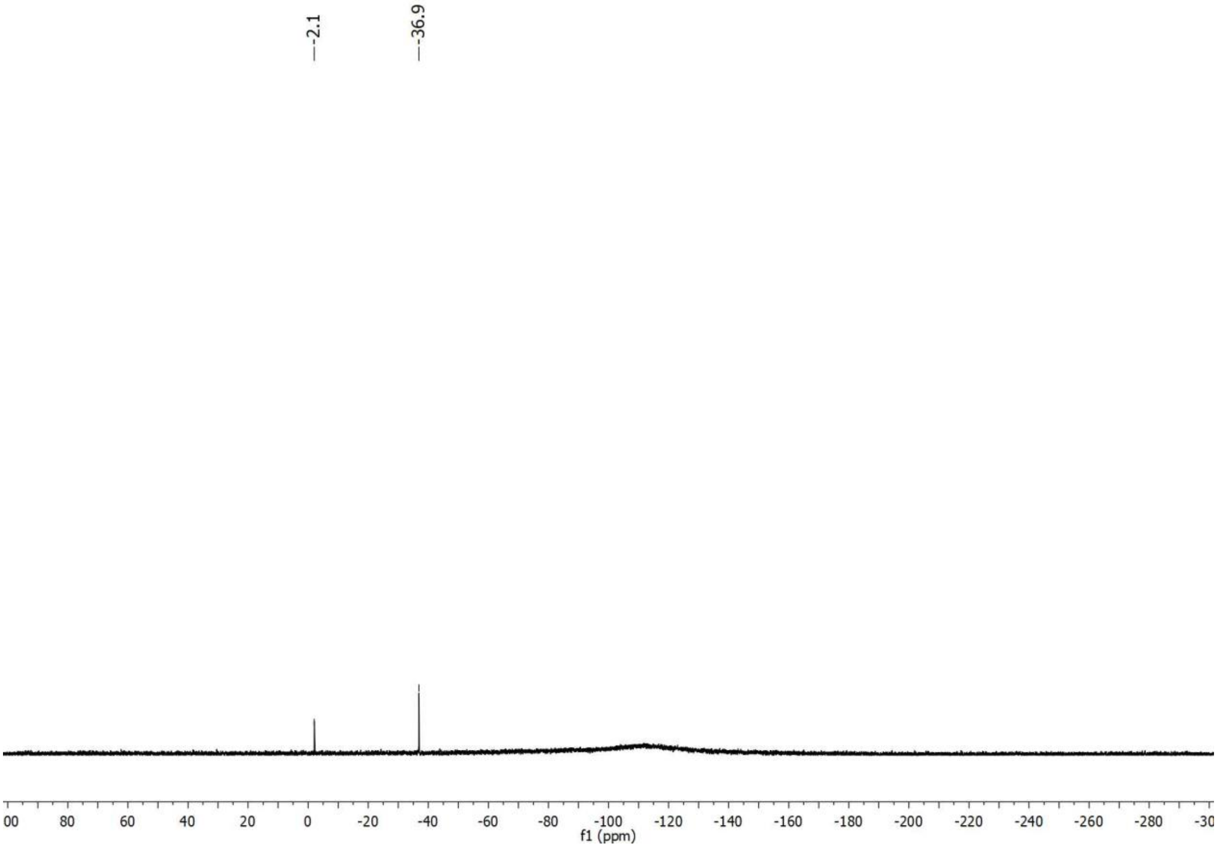

Tert-butyl((decyl(phenyl)silyl)ethynyl)dimethylsilane (7ag-a)

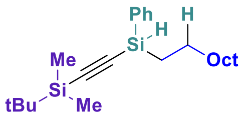

^{1}H NMR

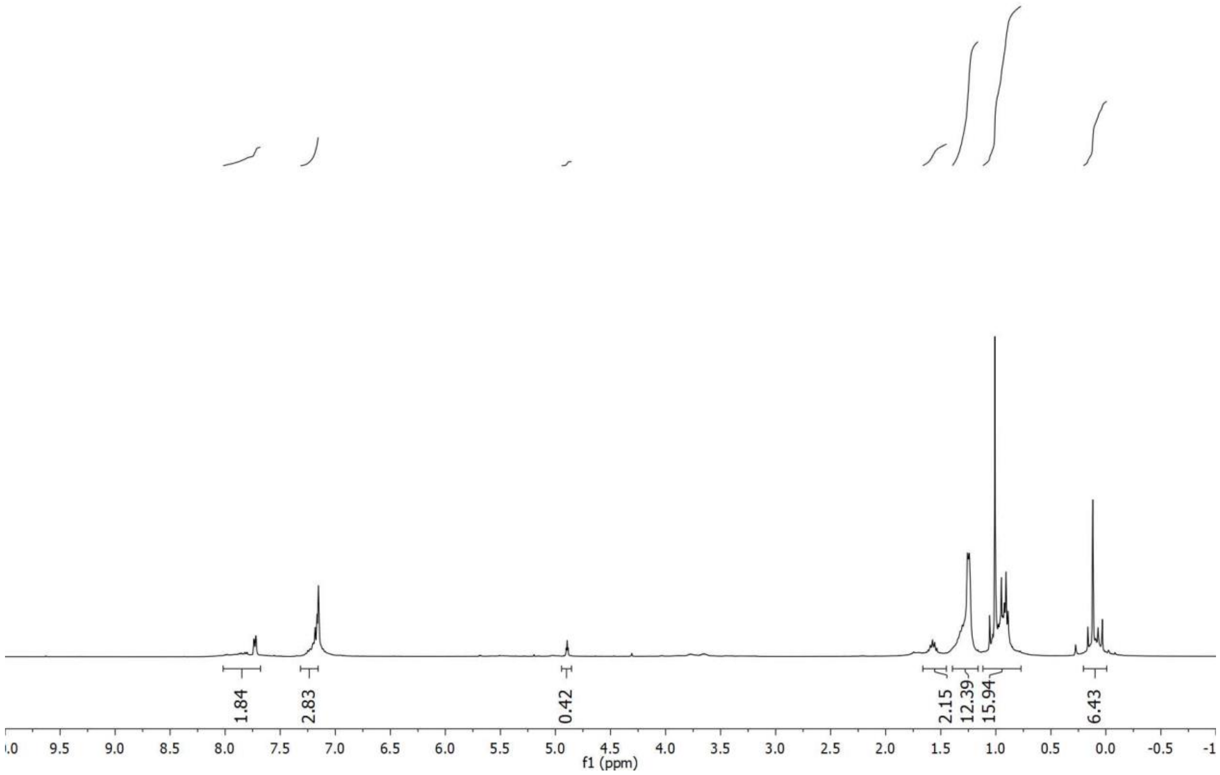

^{13}C NMR

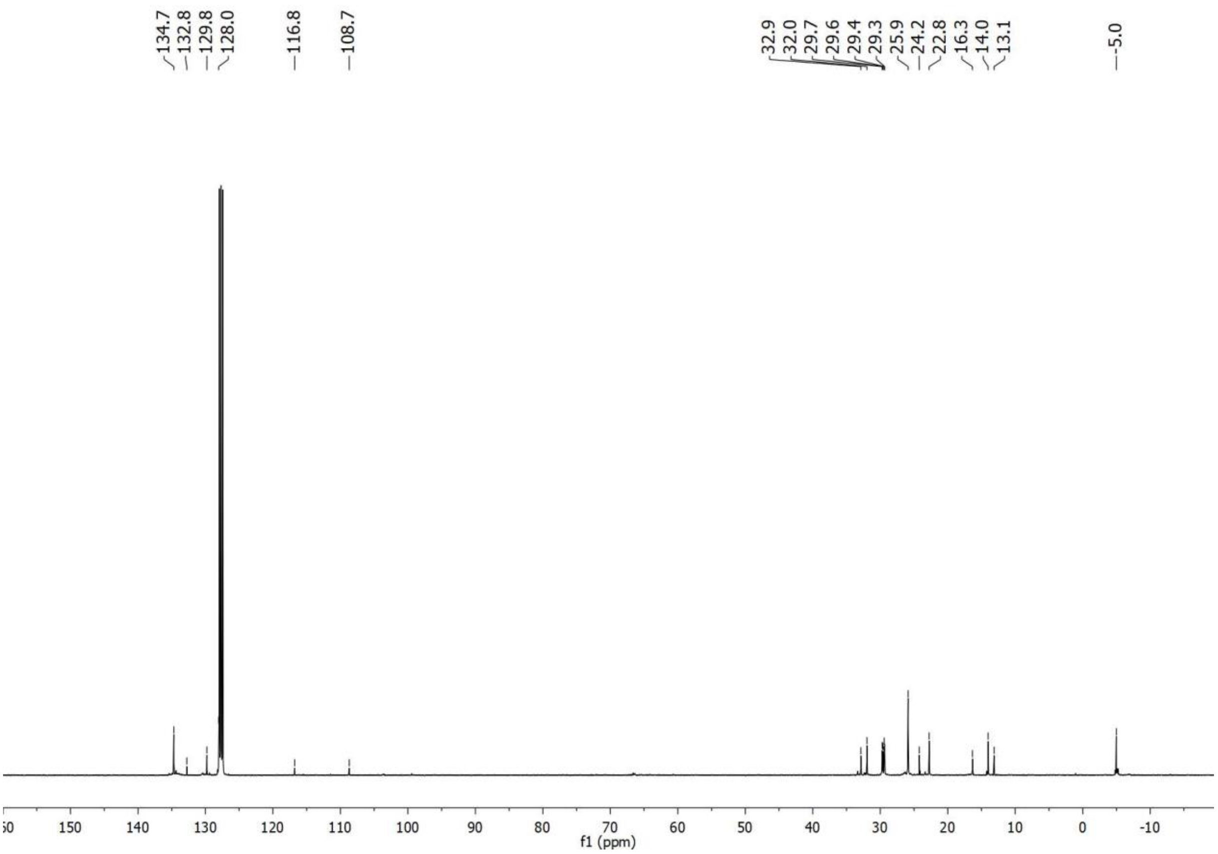

^{29}Si NMR

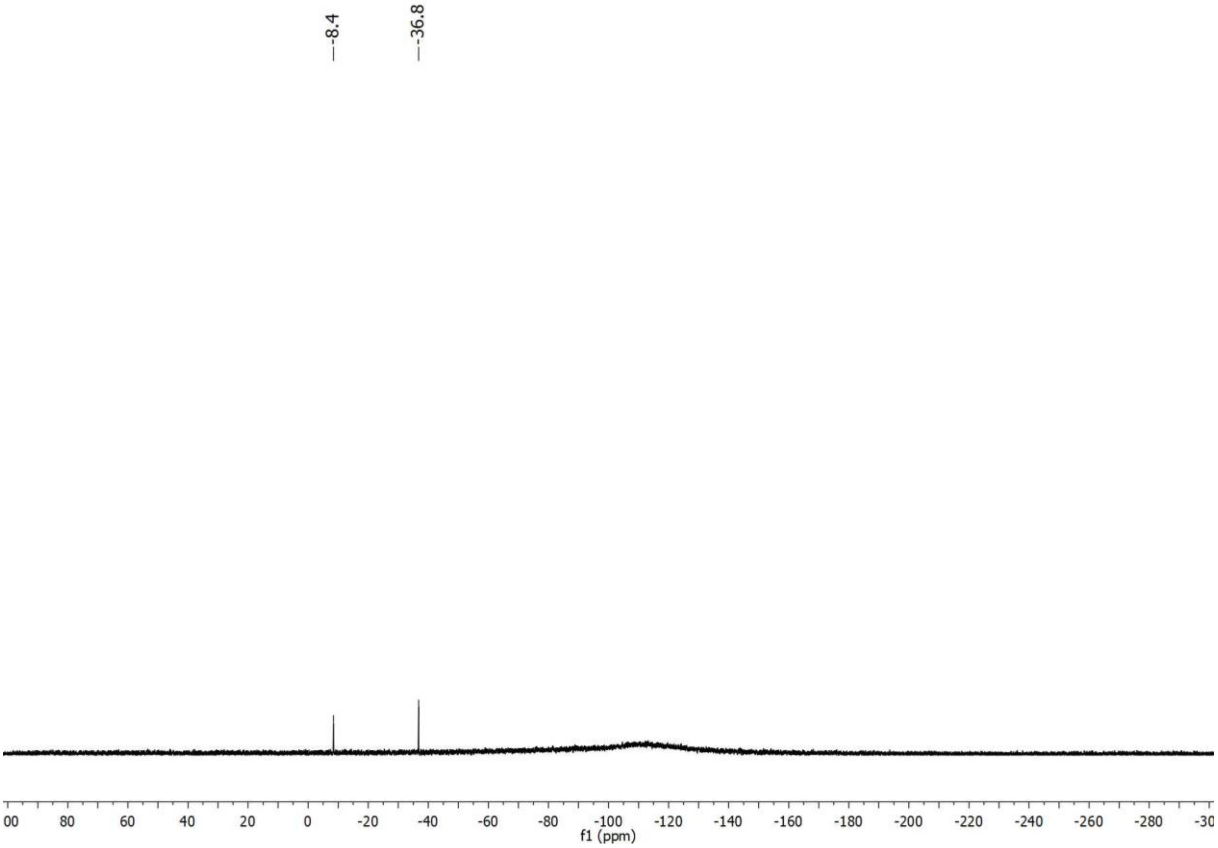

Triethyl(((3-(glycidoxy)propyl)(phenyl)silyl)ethynyl)silane (7ab-b)

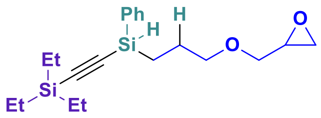

^{1}H NMR

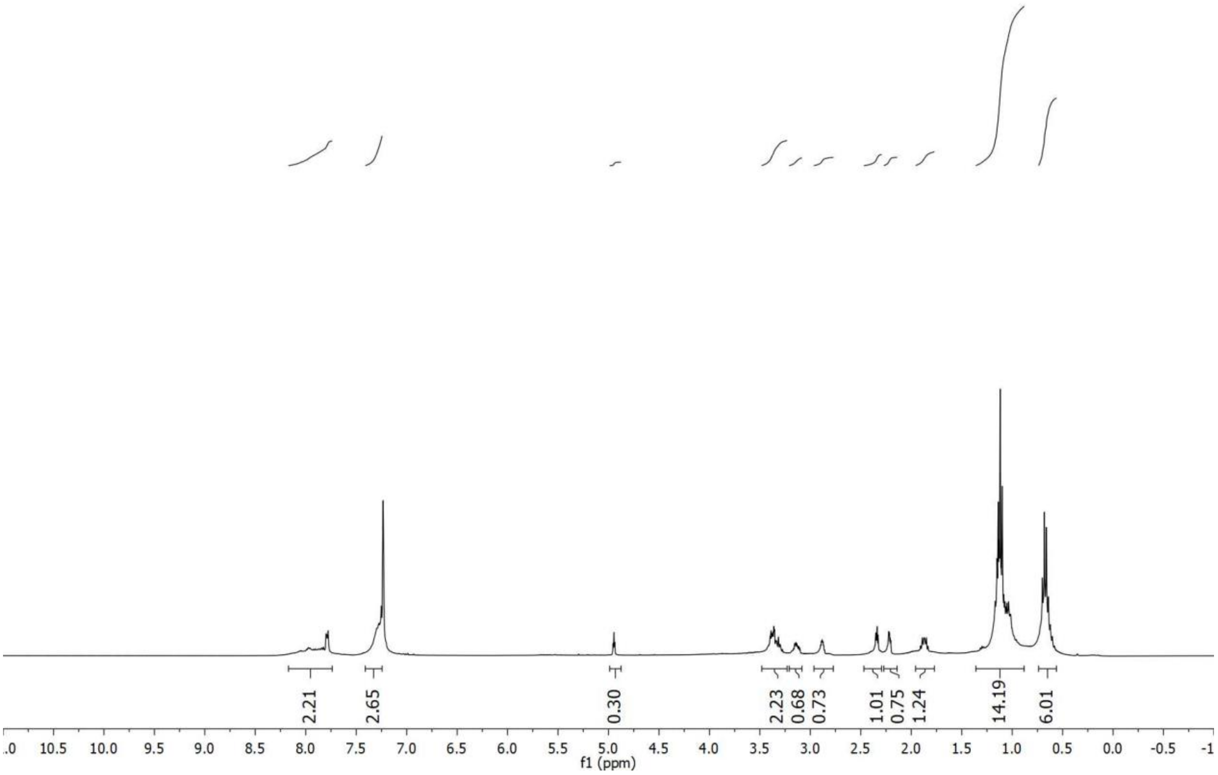

13C NMR

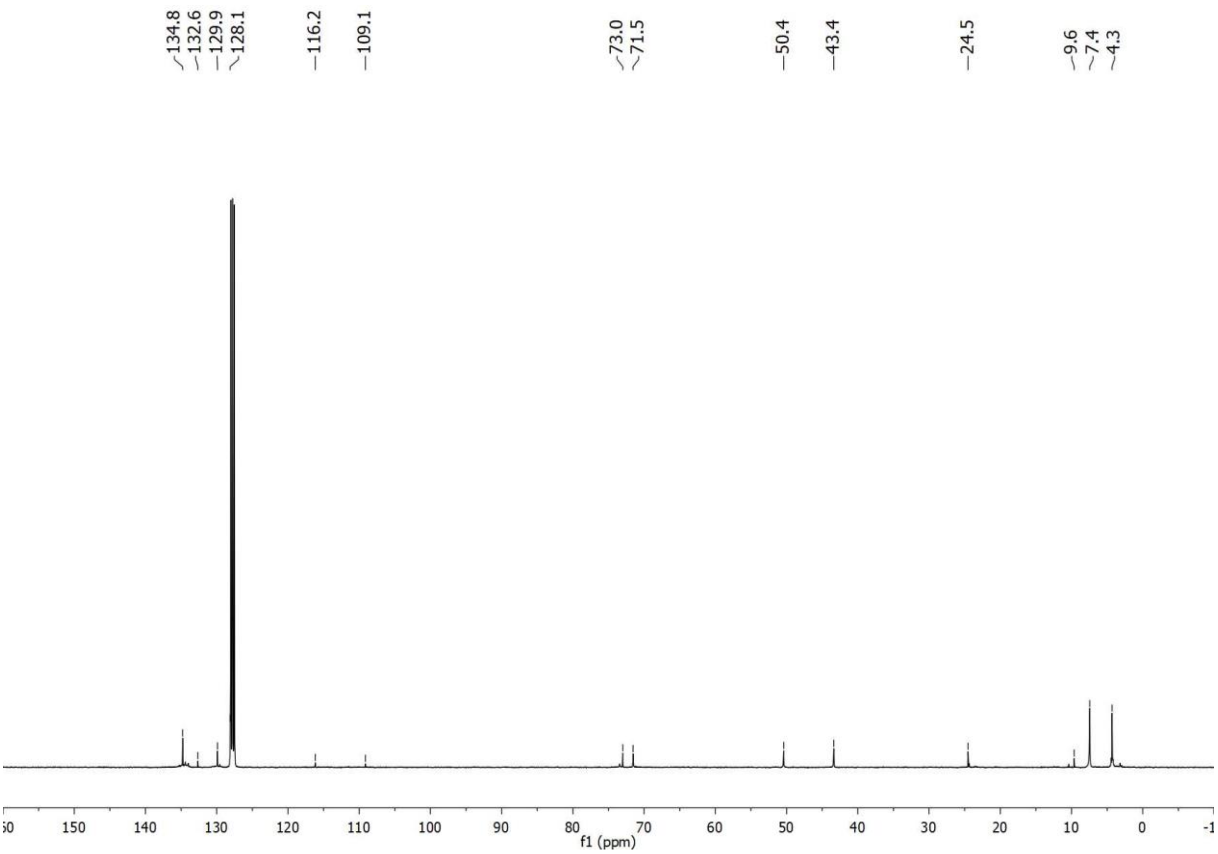

29Si NMR

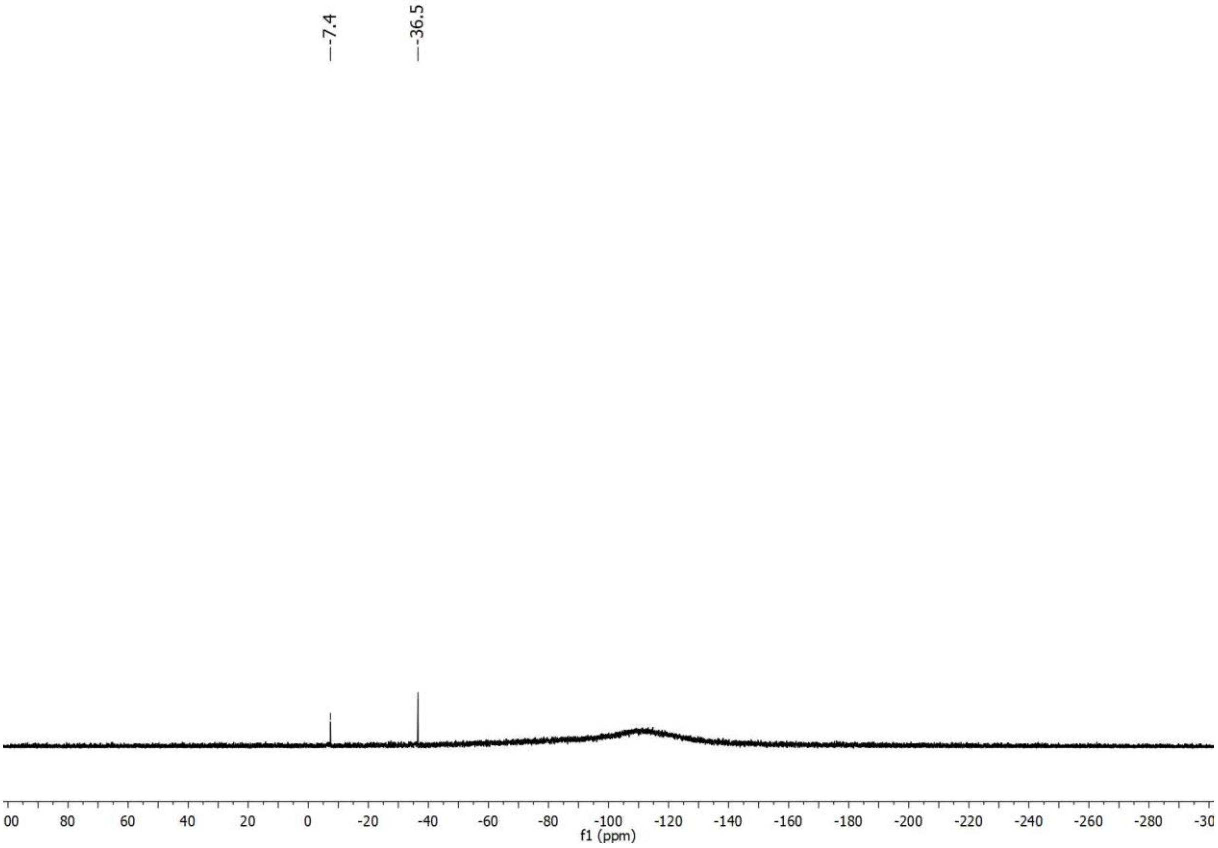

Triisopropyl(((3-(glycidoxy)propyl)(phenyl)silyl)ethynyl)silane (7ac-b)

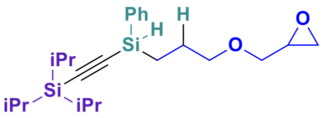

^{1}H NMR

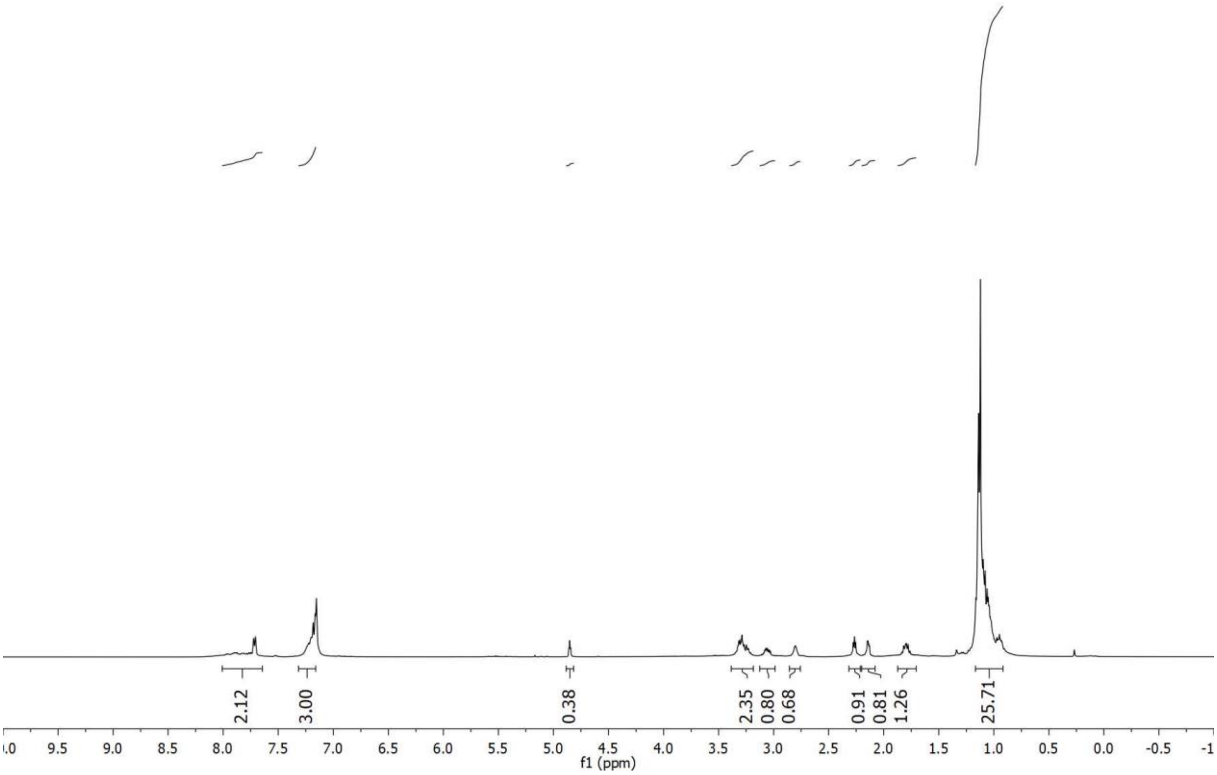

^{13}C NMR

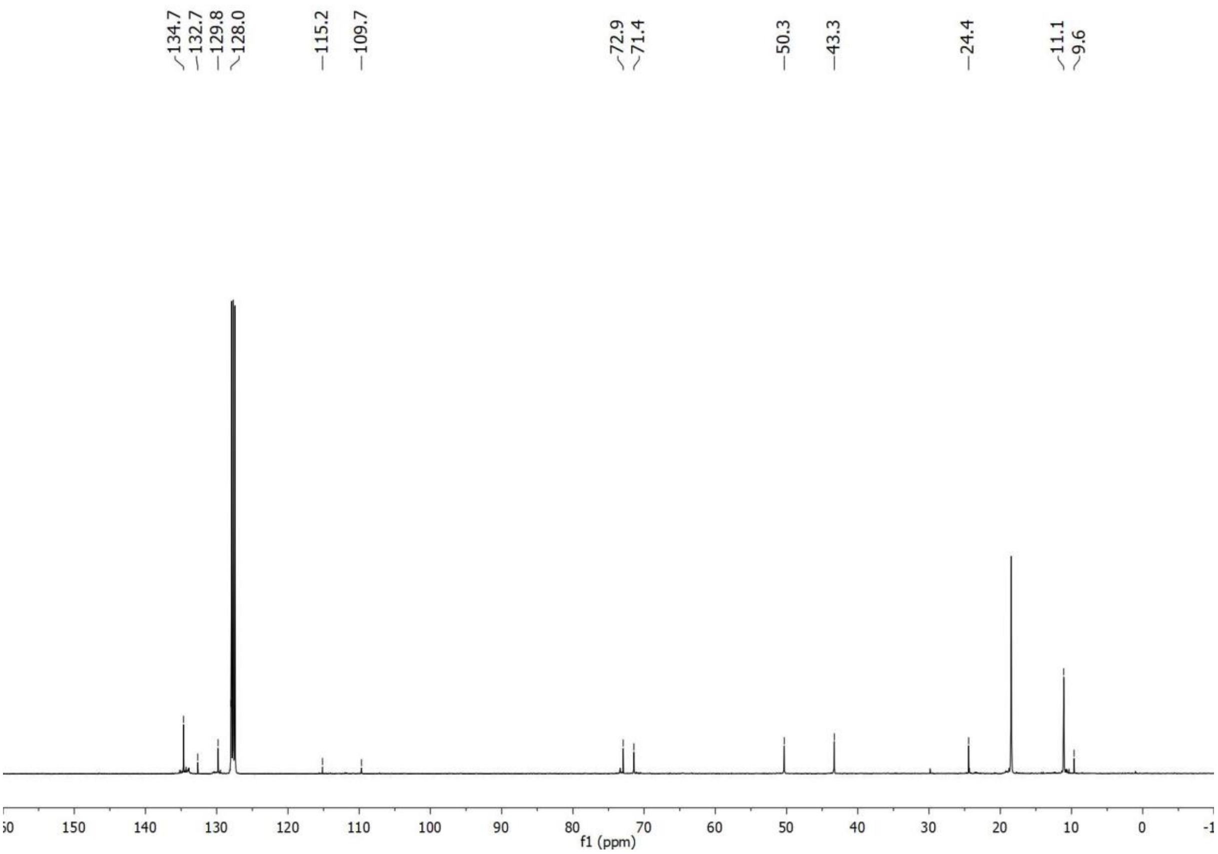

^{29}Si NMR

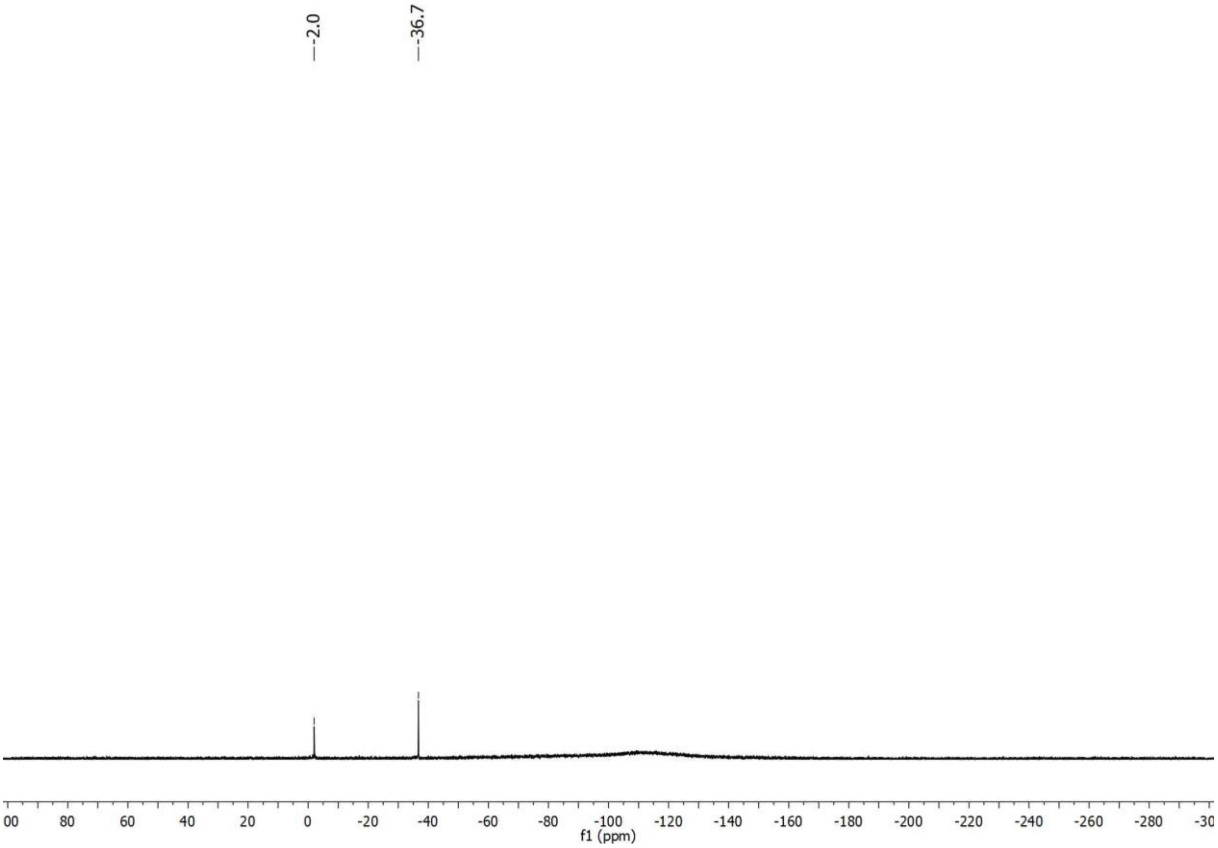

Tert-butyl(dimethyl)(((3-(glycidoxy)propyl)(phenyl)silyl)ethynyl)silane (7ag-b)

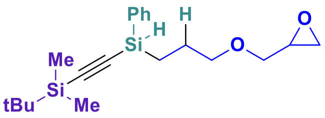

^{1}H NMR

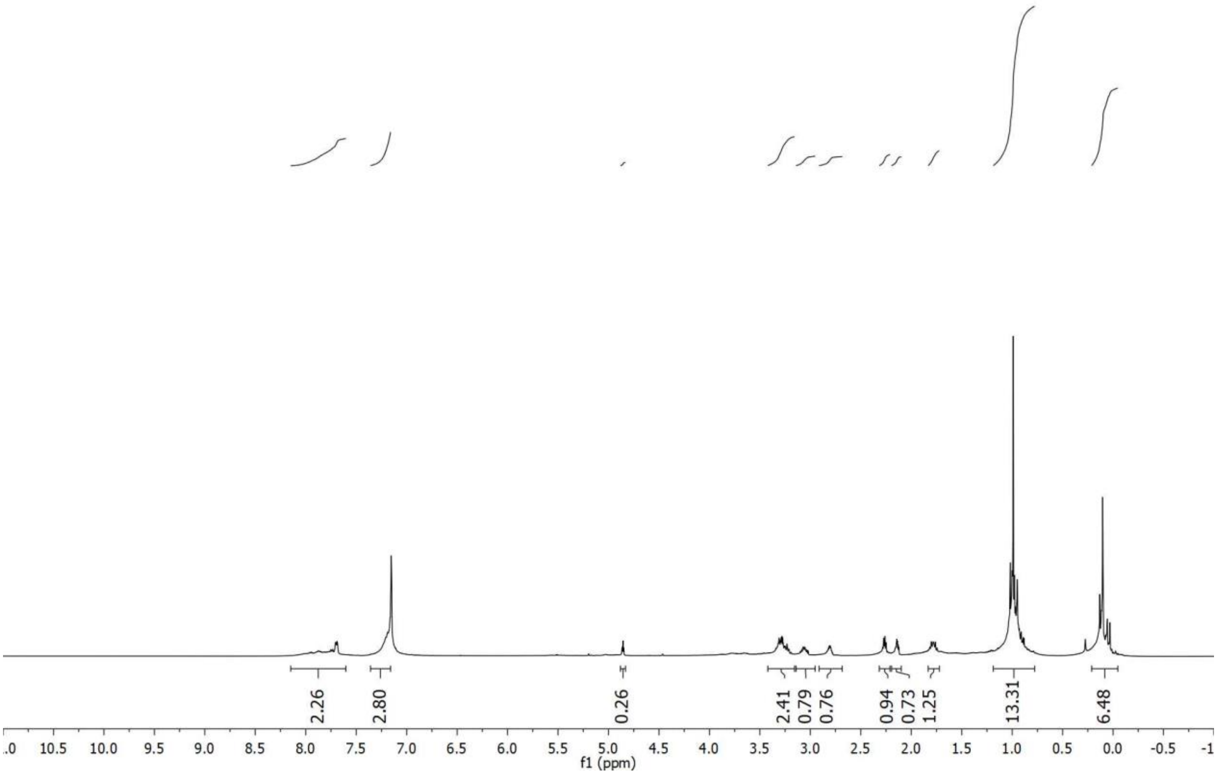

^{13}C NMR

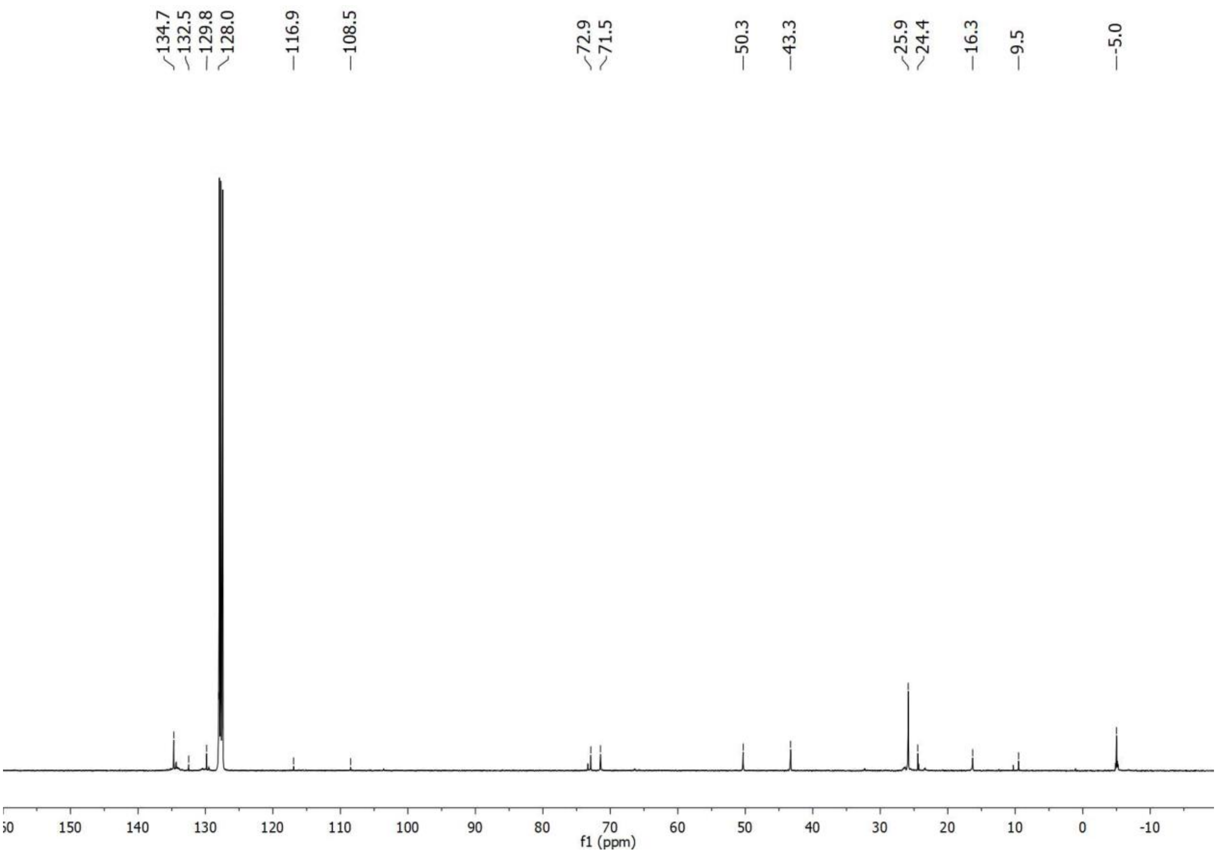

^{29}Si NMR

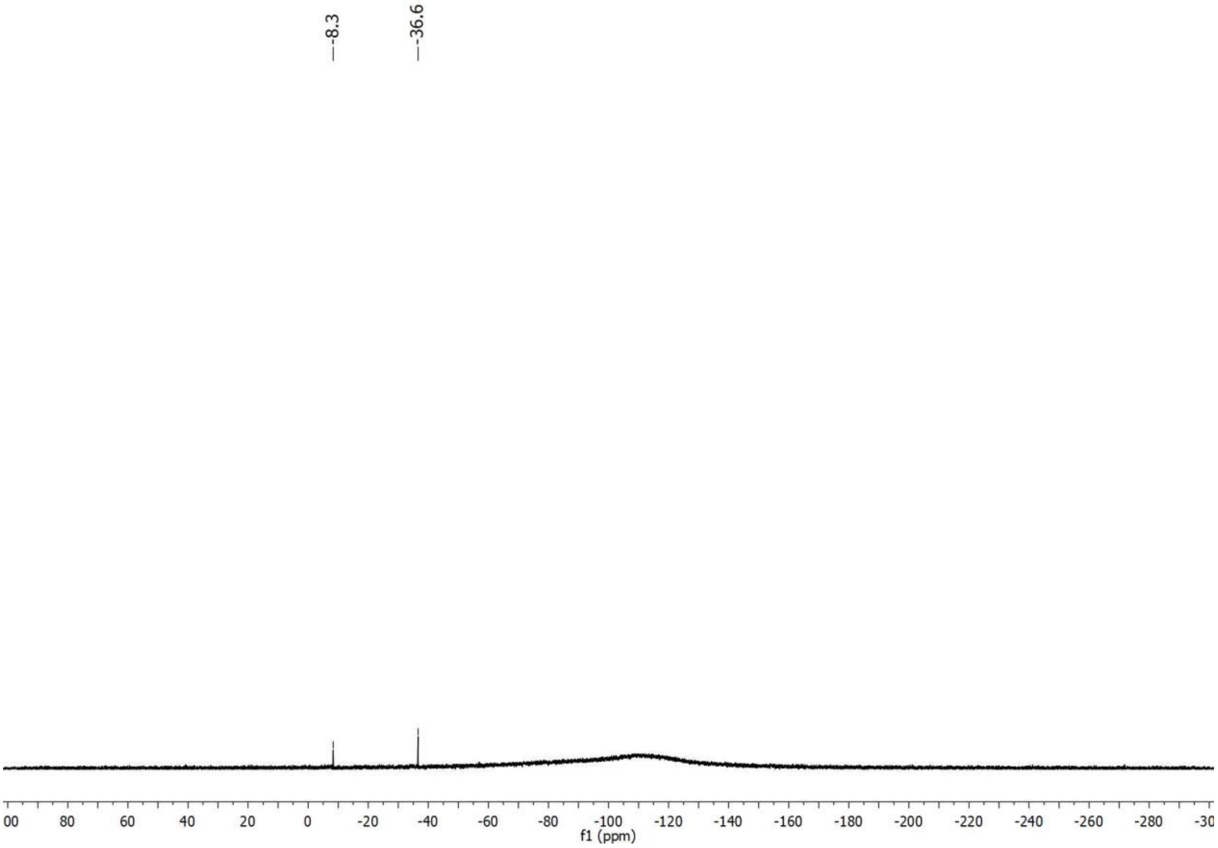

MECHANISTIC STUDIES

Supplement 1

***N*²,*N*⁴-bis(diisopropylphosphino)-6-methyl-1,3,5-triazine-2,4-diamine**

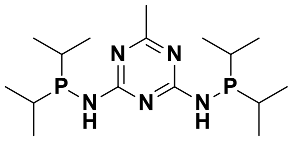

*N*²,*N*⁴-bis(diisopropylphosphino)-6-methyl-1,3,5-triazine-2,4-diamine was obtained as solid in 74% yield. The title compound was known in the literature and all spectroscopic data are in agreement.[7]

¹H NMR (400 MHz, C_6D_6) δ (ppm) = 0.83–1.01 (m, 24H), 1.71 (s_br, 4H), 2.38 (s, 3H), 5.45 (s_br, 2H).

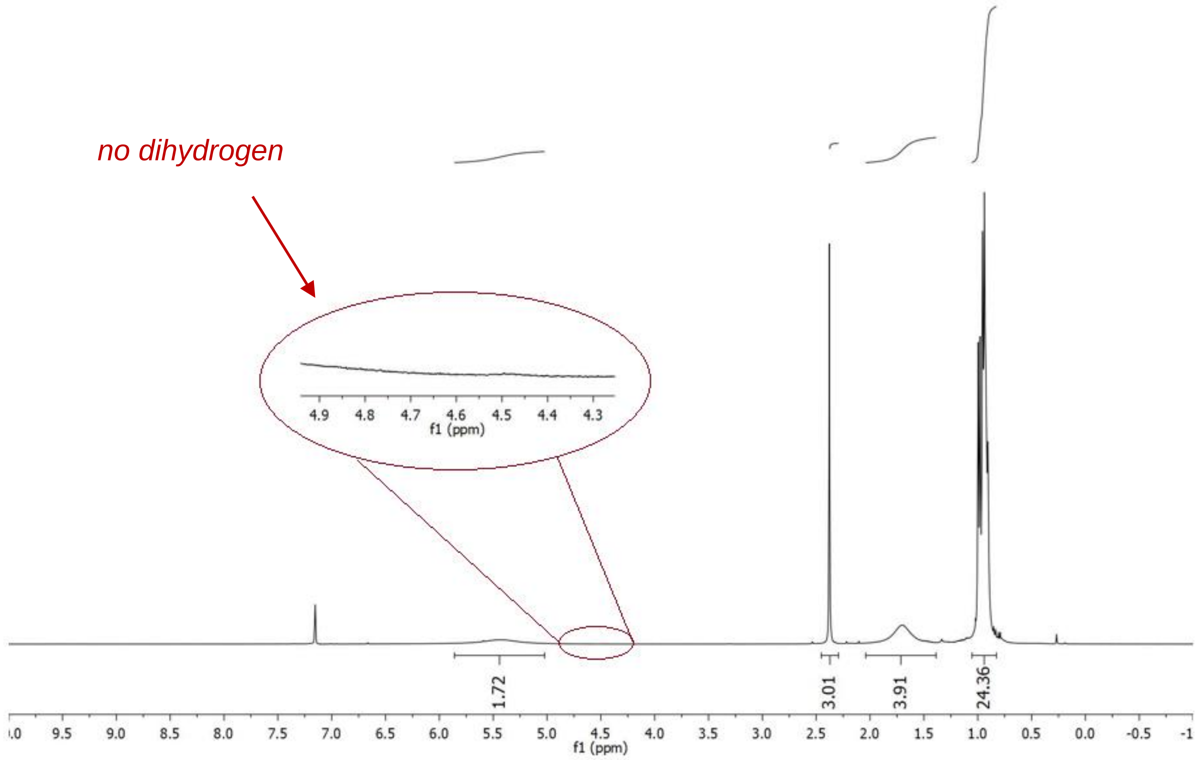

Supplement 2

Conditions: 1 eq. of **B**, 2.2 eq. of **1a**, THF-d_8, 50°C, 1h.

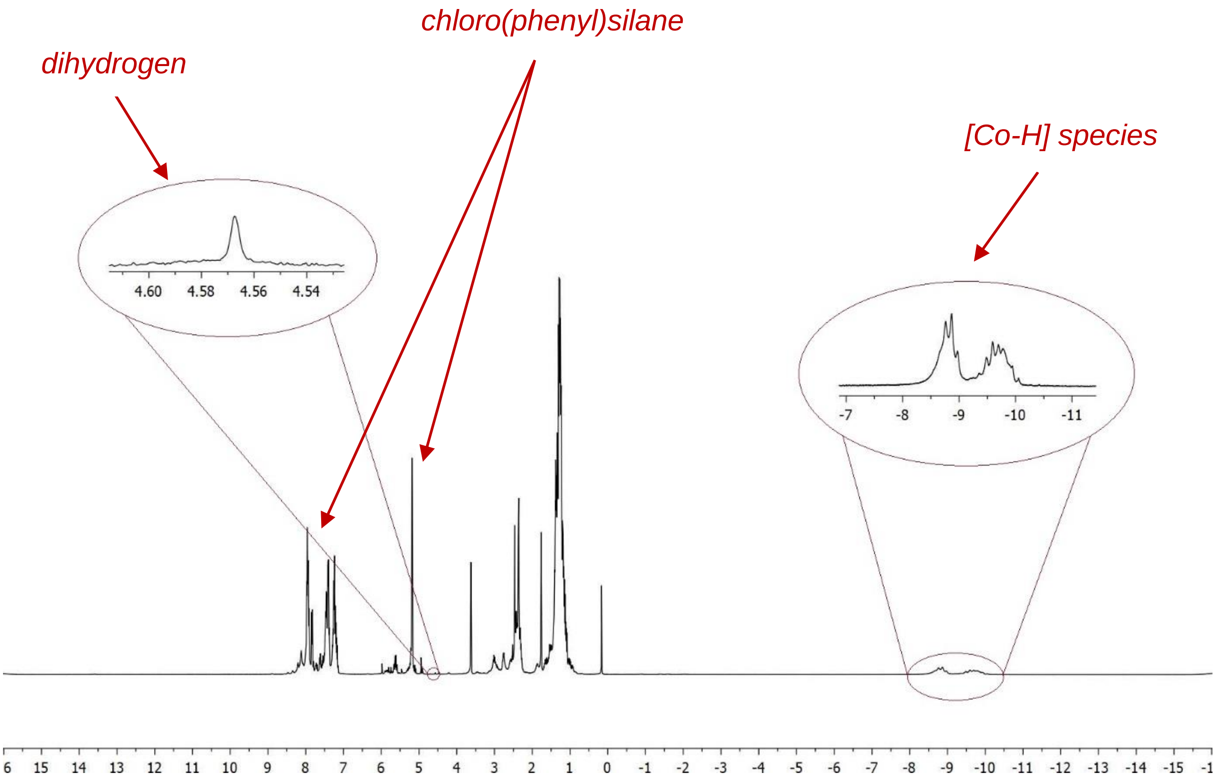

Supplement 3

Conditions: 1eq. of **B**, 10 eq. of **1a**, THF-d_8, 50°C, 1h.

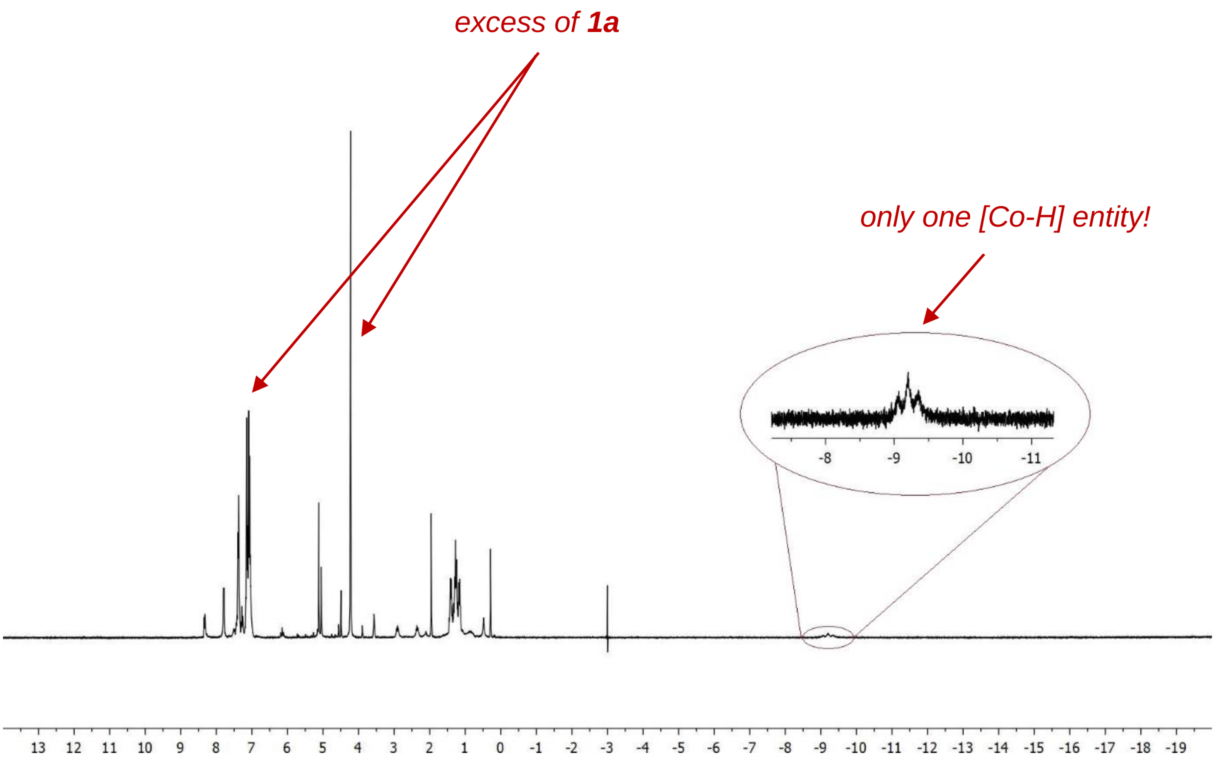

Supplement 4

Conditions: 1eq. of **B**, 10 eq. of **1a**, THF-d_8, 50°C, 1 h; then added 15 eq. of **2c**, rt.

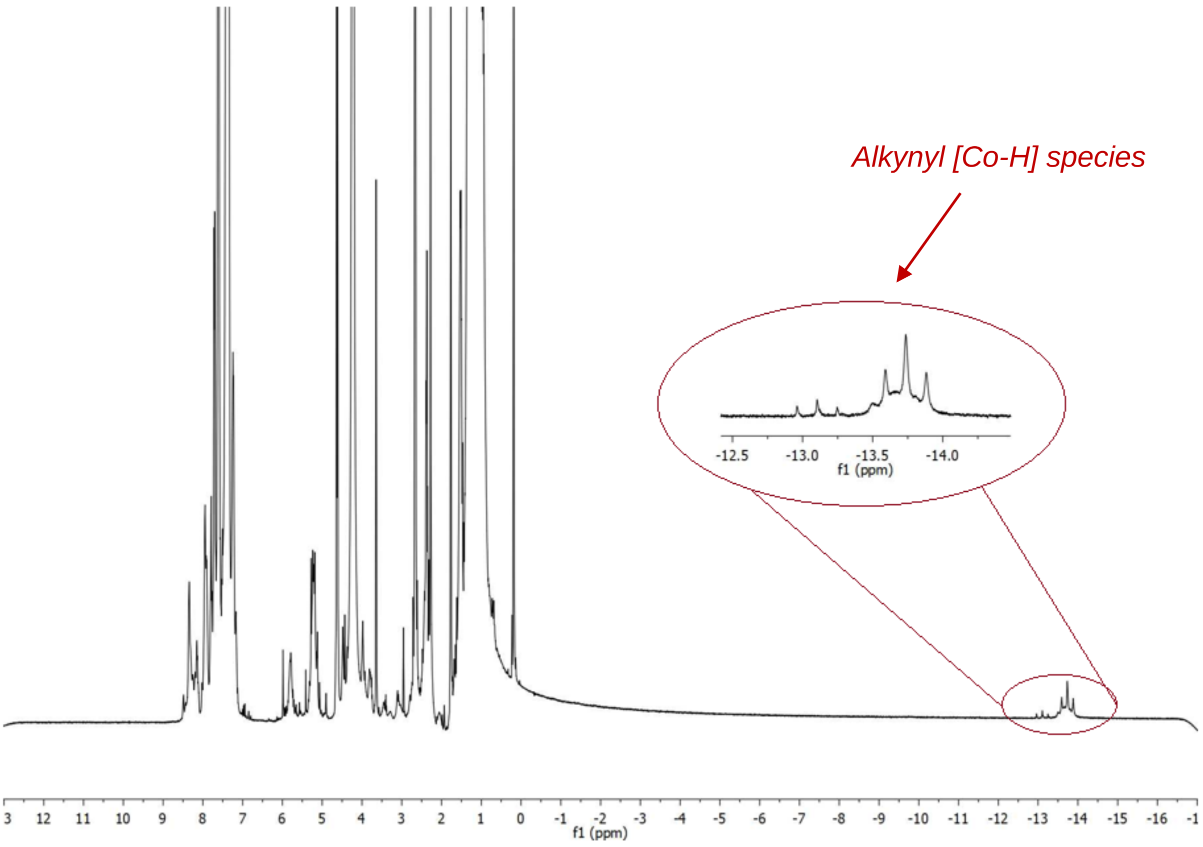

Supplement 5

The GC-MS analysis confirmed the conversion of chlorosilane to 1,3-diphenyldisiloxane, when a stoichiometric amount of H_2O was added

Characterization data for 1,3-diphenyldisiloxane

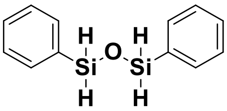

EI-MS m/z (rel. int.): 231 (26%, $[M+H]^+$), 227 (10), 207 (3), 183 (6), 181 (11), 151 (100), 107 (69), 78 (37), 74 (68), 55 (7), 53 (15), 51 (10).

REFERENCES

[1] N. Deibl, R. Kempe, *J. Am. Chem. Soc.* **2016**, *138*, 10786-10789.

[2] M. Skrodzki, S. Witomska, P. Pawluć, *Dalton Trans.* **2018**, *47*, 5948-5951.

[3] A. K. Dash, J. X. Wang, J. C. Berthet, M. Ephritikhine, M. S. Eisen, *J. Org. Chem.* **2020**, *604*, 83-98.

[4] A. K. Dash, J. Q. Wang, J. Wang, I. Gourevich, M. S. Eisen, *Journal of Nuclear Science and Technology* **2002**, *39*, 386-392.

[5] A. K. Dash, J. Q. Wang, M. S. Eisen, *Organometallics* **1999**, *18*, 4724-4741.

[6] Y. Ma, S.-J. Lou, G. Luo, Y. Luo, G. Zhan, M. Nishura, Y. Luo, Z. Hou, *Angew. Chem. Int. Ed.* **2018**, *57*, 15222-15226.

[7] S.S. Gawali, B. K. Pandia, C. Gunanathan, *Org. Lett.* **2019**, *21*, 3842–3847.
